# Hydroxymethylation hydroxylation of 1,3-diarylpropene through a catalytic diastereoselective Prins reaction: cyclization logic and access to brazilin core

**DOI:** 10.1007/s13659-024-00450-2

**Published:** 2024-05-14

**Authors:** Xin-Ting Hu, Qing-Yan Cheng, Yan-Ping Chen, Kun Li, Cai-Xian Yan, Dashan Li, Li-Dong Shao

**Affiliations:** 1 Yunnan Key Laboratory of Southern Medicinal Utilization, School of Chinese Materia Medica, Yunnan University of Chinese Medicine, Kunming, 650500 China; 2 Yunnan Precious Metals Laboratory, Kunming Institute of Precious Metals, Kunming, 650106 China

**Keywords:** Catalytic Prins reaction, Hydroxymethylation/hydroxylation, 1,3-Diarylpropene, Brazilin

## Abstract

A catalytic diastereoselective Prins reaction for hydroxymethylation and hydroxylation of 1,3-diarylpropene was successfully utilized to prepare various 1,3-dioxanes **7** in 14–88% yields. Take advantage of the synthetic intermediate **7h**, the key B/C rings in brazilin core could be constructed by the sequential of Friedel–Crafts/Ullmann-Ma rather than Ullmann-Ma/Friedel–Crafts reactions.

**Graphical Abstract:**

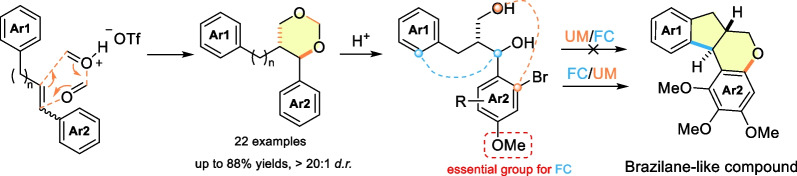

**Supplementary Information:**

The online version contains supplementary material available at 10.1007/s13659-024-00450-2.

## Introduction

**Scheme 1:**
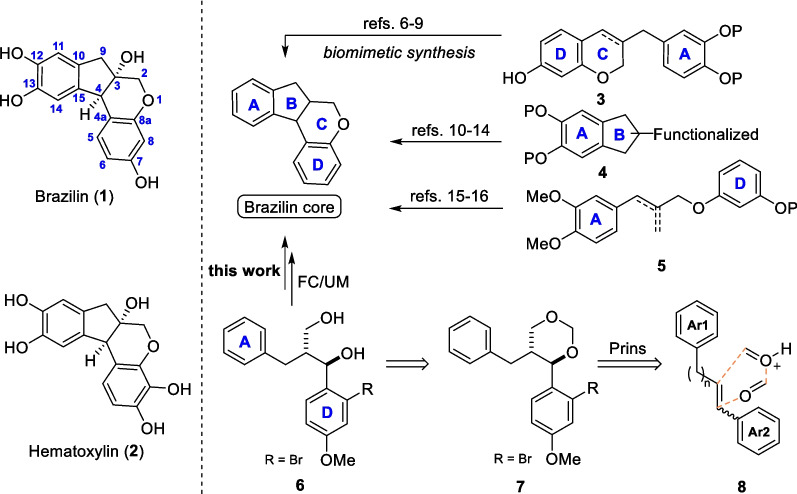
Synthetic routes to brazilin-type compounds

The brazilin family of natural products is a group of homoisoflavonoids with *oxo*-6/5/6/6 fused tetracyclic tetrahydroindeno[2,1-*c*]chromene core from the traditional Chinese medicine ‘Sumu’ (*Caesalpinia sappan L.*) [1], of which brazilin (**1**) possesses antitumor, hypoglycemic, anti-inflammatory, and hepatoprotective pharmacological activities [2–4], and hematoxylin (**2**) exhibits c-Src inhibitory activity and is an excellent tyrosine kinase inhibitor [5] (Scheme 1). Organic chemists have extensively studied the total synthesis of such biologically active molecules. Representative routes mainly rely on the biogenetic precursors **3**, [6–9] indane derivatives **4**, [10–14] phenylpropanoid derivatives **5**, [15, 16] and others [17, 18] as key intermediates. We propose that the generation of the tetracyclic brazilin core from 1,3-diol **6** through Ullmann-Ma (UM) and Friedel–Crafts (FC) reactions may present a new strategy for synthesizing this class of ring systems, and 1,3-dioxane **7** is an excellent precursor for the preparation of 1,3-diol **6** through the ring opening under the acid condition. Compound **7** can be prepared by the reaction of diarylpropene **8** with formaldehyde or paraformaldehyde (PF) via Prins reaction [19]. In this article, we report the synthesis of series 1,3-dioxane **7** through the TfOH-catalyzed diastereoselective Prins reaction of diarylpropene **8** with PF. We also explore the cyclization logic for the synthesis of brazilin core from 1,3-diol **6** via UM and FC reactions.

## Results and discussion

**Scheme 2:**
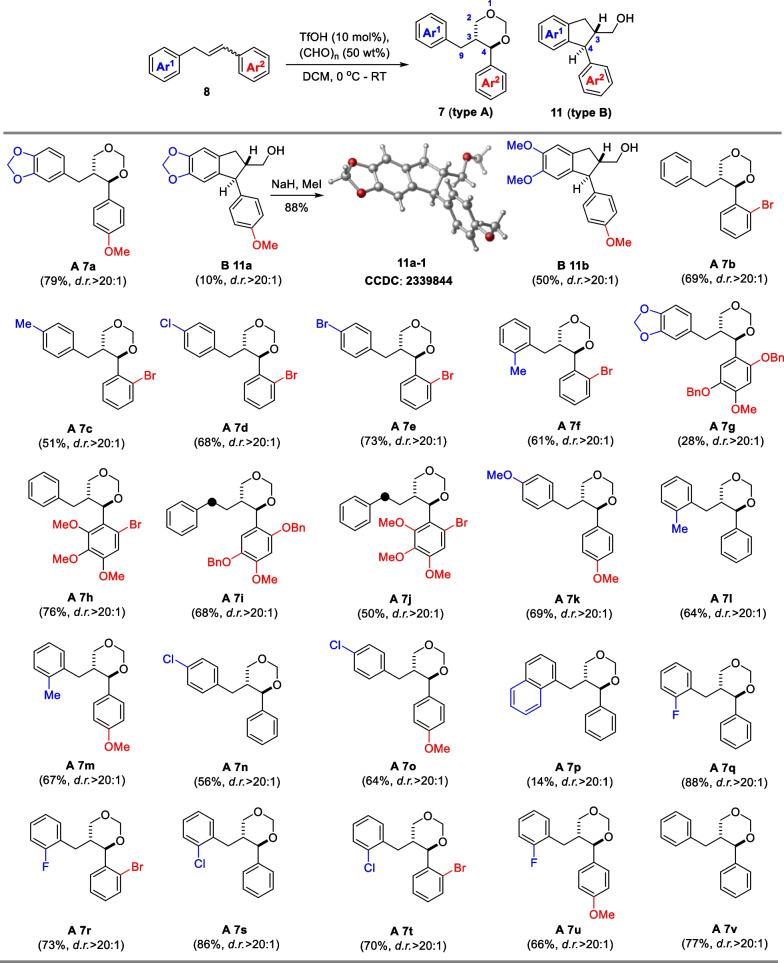
Synthesis of 1,3-dioxanes **7**

Recently, List et al. reported the synthesis of chiral 1,3-dioxanes through the imino-imidodiphosphate (iIDP)-catalyzed asymmetric Prins reaction of styrene with paraformaldehyde (PF) [20]. However, we found both iIDP and *N*-triflyl phosphoramides (NTPAs) [21] were unable to catalyze the Prins reaction of diarylpropene **8a** with PF (data not shown). Aiming at the preparation of 1,3-dioxane **7**, we screened the conditions for the Prins reaction of diarylpropene **8a** with formaldehyde or PF using Cu(OTf)_2_ [22] or TfOH (Table 1). Heating **8a** with formaldehyde or PF in the presence of Cu(OTf)_2_ (5 mol%) generated the target product **7a** in up to 36% yield, along with a small amount of ring-opening and subsequently FC cyclized products **11a** and **11a′**, while the reactions did not occurred at room temperature (Table 1, entries 1–4). Cu(BF_4_)_2_ gave a comparable yields of **7a** to Cu(OTf)_2_ (entry 5), and DCM was seemed the optimal choice of solvent (entries 6–11). Replacement of Cu(OTf)_2_ with TfOH, no reaction was detected at 0 °C (entry 12), but at room temperature, TfOH could achieve similar results to Cu(OTf)_2_ (entry 13). Further increasing the loading of TfOH to 10 mol% resulted in the desired product **7a** (79% yield) with excellent diastereoselective ratio (*d*.*r*.) > 20:1 and a small amount of **11a** (entry 14). A large coupling constant of 9.8 Hz (^3^*J*_H3–H4_) indicated the *trans*-configuration of C3,C4 stereochemistry in **7a**. The relative configuration of **11a** was determined to be *trans*- through X-ray single-crystal diffraction of its methylated derivative **11a-1** (Scheme 2).

**Table 1:** Optimizations of the diastereoselective Prins cycloaddition


Entry	Conditions	Yields (%)^a^ **7a**/**11a/11a′**
1	Cu(OTf)_2_ (5 mol%), (CHO)_n_ (50 wt%), DCM, RT	NR^b^
2	Cu(OTf)_2_ (5 mol%), 37% HCHO (500 mol%), DCM, RT	NR
3	Cu(OTf)_2_ (5 mol%), (CHO)_n_ (50 wt%), DCM, 60 °C	36/18/14
4	Cu(OTf)_2_ (5 mol%), 37% HCHO (500 mol%), DCM, 60 °C	30/11/8
5	Cu(BF_4_)_2_ (5 mol%), (CHO)_n_ (50 wt%), DCM, 60 °C	33/15/12
6	Cu(OTf)_2_ (5 mol%), (CHO)_n_ (50 wt%), DCE, 60 °C	NR
7	Cu(OTf)_2_ (5 mol%), (CHO)_n_ (50 wt%), CHCl_3_, 60 °C	NR
8	Cu(OTf)_2_ (5 mol%), (CHO)_n_ (50 wt%), THF, 80 °C	Decomposed
9	Cu(OTf)_2_ (5 mol%), (CHO)_n_ (50 wt%), MeCN, 60 °C	NR
10	Cu(OTf)_2_ (5 mol%), (CHO)_n_ (50 wt%), DMF, 100 °C	NR
11	Cu(OTf)_2_ (5 mol%), (CHO)_n_ (50 wt%), toluene, 100 °C	NR
12	TfOH (5 mol%), (CHO)_n_ (50 wt%), DCM, 0 °C	NR
13^c^	TfOH (5 mol%), (CHO)_n_ (50 wt%), DCM, 0 °C-RT	33/27/0
14^c^	TfOH (10 mol%), (CHO)_n_ (50 wt%), DCM, 0 °C-RT	79/10/0

All reactions were performed in freshly distilled solvents (2 mL) with **8a** (0.1 mmol) at indicated temperature for 24 h

^a^Isolated yield

^b^NR: no reaction

^c^The reaction was initially stirred at 0 °C for 4 h, then reacted at RT for another 20 h.

After obtaining the optimal conditions (as shown in Table 1, entry 14), we investigated the substrate scope for this reaction (Scheme 2). The results indicated that only **8a** and **8w** (*Z*/*E* mixture, Additional file 1: Scheme S1) could directly produce the indane-type product **11** (type B). Specifically, **8a** mainly led to **7a** and **8w** mainly generated **11b** under the optimal conditions, while all of the other substrates produced the 1,3-dioxane-type product **7** (type A) with excellent diastereoselectivity (d.r. > 20:1). The reaction exhibited a certain range of substrate adaptation. The Ar^1^ fragment tolerated with *ortho*- or *para*-substituted electron-donating groups (EDGs) giving **7a**, **11a**, **11b**, **7c**, **7e**–**g**, **7k**-**m**, **7p** in 10–79% yields, as well as *ortho*- or *para*-substituted electron-withdrawing groups (EWGs) delivering **7d**, **7n**, **7o**, **7q**–**u** in 14–88% yields (Scheme 2). However, the Ar^2^ fragment could only tolerate with the EDGs substitution, as the EWGs prevented the reaction from occurring (**8 × **in Additional file 1: Scheme S1). It is important to note that substrates **8i** and **8j** with four-carbon alkyl chain were equally capable of undergoing similar transformations (**7i** and **7j**).

**Scheme 3:**
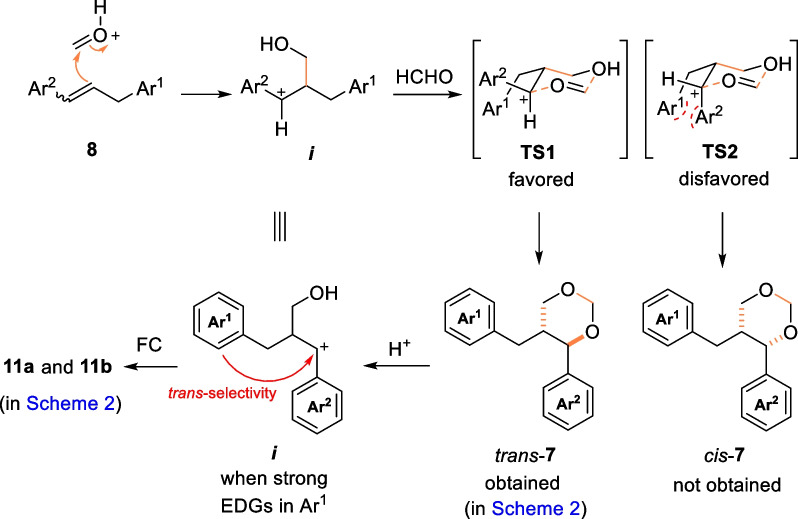
Proposed mechanism for the formation of **7** and **11**

The mechanism of the reaction was postulated in Scheme 3. Given that the reaction yielded highly diastereoselective products **7** and **11** from substrate **8**, it was hypothesized that the Prins reaction was a stepwise process [20]. Namely, the benzyl cation ***i*** was produced when **8** first underwent the Prins reaction with protonated formaldehyde. This step was significantly influenced by the electrical properties of Ar^2^ fragment, Ar^2^ with EDGs favoring the reaction and EWGs having the opposite effect. These results were consistent with those obtained in our experiments (Scheme 2). Then, by reacting with another molecular formaldehyde via the dominant transition state **TS1**, *trans*-**7** was produced, while *cis*-**7** resulting from the disfavored transition state **TS2** was not detected. Alternatively, both products **11a** and **11b** could be generated simultaneously through further protonation ring-opening/FC reactions of *trans*-**7a** and **7b**, as well as through the direct FC reaction of ***i*** (when strong EDGs were present in Ar^1^) [22, 23].

**Scheme 4:**
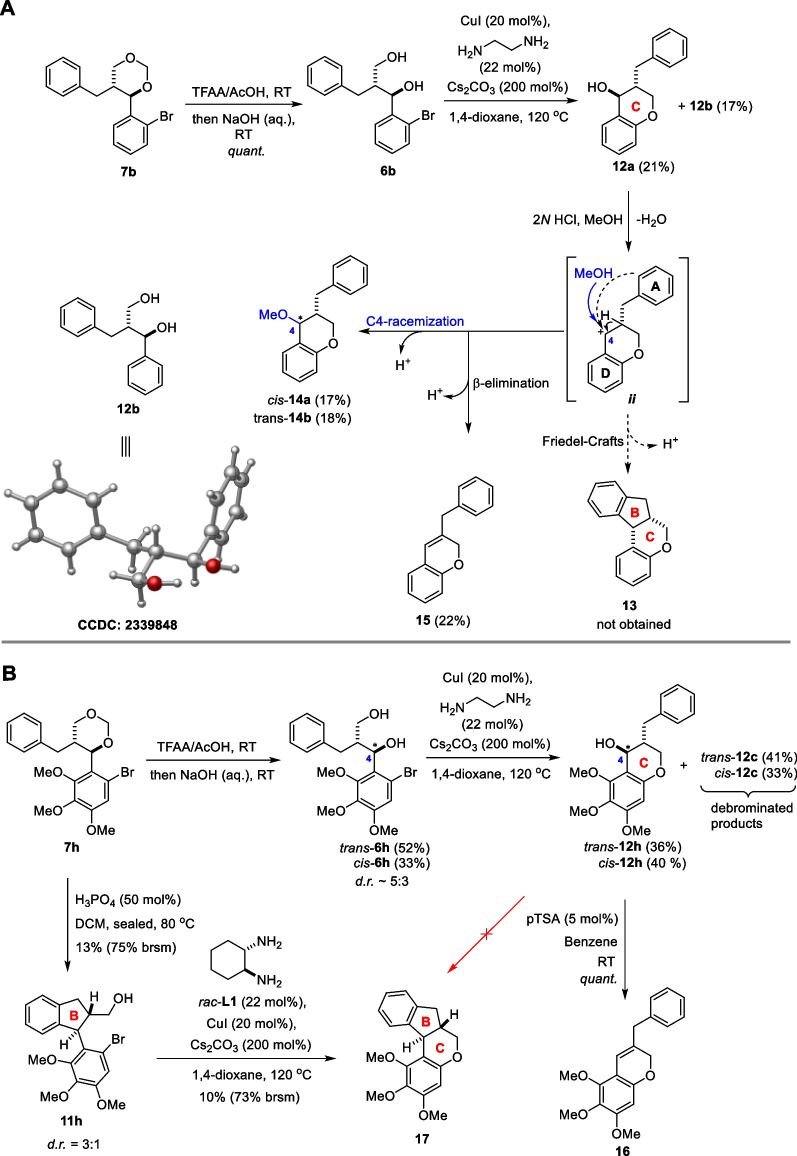
Construction of the brazilin core from **7b** and **7h**

To construct the braziline core, we used **7b** as a substrate (Scheme 4A). Under acidic conditions, **7b** underwent ring-opening to give 1,3-diol **6b** in quantitative yield. Subsequent UM reaction of **6b** produced cyclization product **12a**, which facilitated construction of the C-ring in the braziline core. A small amount of **12b** was also observed as the debromination product of **6b**. The X-ray single-crystal diffraction structure of **12b** confirmed its relative configuration to be *trans*, which in turn verified the *trans*-configuration of 1,3-dioxane **7**. However, treatment of **12a** with various acids did not lead to the expected FC cyclization. The use of Lewis acids (BF_3_·OEt_2_, AlCl_3_, Cu(OTf)_2_, etc.) caused the decomposition of **12a**, while Brönsted acids (HCl, pTSA, H_3_PO_4_, etc.) mainly produced the C4 racemized products **14a** and **14b** with a minor eliminated product **15**. We hypothesized that the reason for the unsuccessful FC reaction of **12a** may be attributed to the inert aryl rings A and D, which lack EDGs activation [17]. Specifically, aryl ring D cannot stabilize the benzylic cation ***ii***, while aryl ring A is difficult to capture ***ii*** to form cyclized product. To address this issue, we utilized **7h** as a substrate for further attempts, which contains three OMe groups on aryl ring D (Scheme 4B). Under acidic conditions, **7h** was similarly converted to ring-opening product **6h** but as a separable mixture of *trans*-**6h** and *cis*-**6h** in 85% yield (*d*.*r*. ~ 5:3). The subsequent UM reactions of *trans*-**6h** and *cis*-**6h** delivered the cyclized products *trans*-**12h** and *cis*-**12h** in 36% and 40% yield, along with debrominated products *trans*-**12c** and *cis*-**12c**, respectively. However, similar to **12a**, attempts to achieve FC cyclization of both *trans*-**12h** and *cis*-**12h** using different acid catalysts were unsuccessful, the eliminated product **16** was obtained as a major product. Inspired by the formation of **11a** in Scheme 2, we hypothesized whether the brazilin core could be constructed through the FC cyclization followed by UM ring closure from **7h**, although this strategy was failed using ‘inert’ **7b**. Namely, **7h** was converted to the cyclized product **11h** as a separable mixture (*d*.*r*. = 3:1) under the catalysis of H_3_PO_4_ in 13% yield (75% brsm). The UM reaction of **11h** successfully enabled the C ring closure, resulting in the final tetracyclic product **17** albeit in 10% yield (73% brsm). It is worth noting that **17** is a first example with the *trans*-fused B/C rings in brazilin core.

## Conclusions

In summary, a catalytic diastereoselective Prins reaction for hydroxymethylation and hydroxylation of 1,3-diarylpropene was successfully utilized to prepare various 1,3-dioxanes **7**. The construction of brazilin core was attempted using intermediates **7b** and **7h**. It was found that UM reaction smoothly achieved C-ring formation, but **7a** could not undergo FC cyclization to construct the B-ring due to lack of EDG activation on aryl ring A. However, **7h** containing the electron-rich aryl ring D was advantageous for the construction of the B-ring using FC reaction. This finding presents an alternative approach to synthesizing the brazilin core and provides insight into constructing B/C rings in similar tetracyclic structures.

## Experimental section

### General information

Unless otherwise noted, all reactions were conducted in oven-dried round-bottom flasks under an argon atmosphere. Solvents were dried and freshly distilled from Na (THF and 1,4-dioxane) under an argon atmosphere. All reagents were from commercial sources without further purification unless otherwise noted. The silica gel (200–300 mesh, Qingdao Marine Chemical Inc., Qingdao, China) was used for column chromatography. Thin layer chromatography (TLC) was carried out on GF plates (0.25 mm layer thickness, Qingdao Marine Chemical Inc.) and was visualized by ultraviolet light (254 nm, if applicable) and phosphomolybdic acid (50 g/L) in EtOH following heating as developing agents. Unless otherwise noted, yields reported were for isolated spectroscopically pure compounds.

^1^H, ^13^C, and ^19^F NMR spectra were recorded on ADVANCE III AM-400 MHz, ADVANCE III AM-500 MHz and ADVANCE III 600 MHz spectrometers (Bruker) at ambient temperature. The residue solvent protons (^1^H) or the solvent carbons (^13^C) were used as internal standards. ^1^H NMR data are presented as chemical shifts in parts per million downfield from tetramethylsilane [multiplicity, coupling constant (hertz), integration]. Chemical shifts (*δ*) are given in parts per million with reference to solvent signals [^1^H NMR: CDCl_3_ (7.26); ^13^C NMR: CDCl_3_ (77.16)]. The following abbreviations are used in reporting NMR data: s, singlet; brs, broad singlet; d, doublet; t, triplet; q, quartet; dd, doublet of doublets; ddd, doublet of doublet of doublets; dt, doublet of triplets; td, triplet of doublets; m, multiplet.

### General procedure for preparation of 1,3-diarylpropenes 8a–8x

According to the literatures [24, 25], 1,3-diarylpropenes **8a–8 × **were synthesized through Wittig reaction from commercially available benzaldehydes and the corresponding phosphonium salts. To a suspension of phosphonium salts (1.1 equiv) in THF (0.3 M) was added dropwise LiHMDS (1 M in THF, 1.1 equiv) at 0 °C, and the resulting mixture was stirred at 0 °C until a clear red solution formed (∼ 30 min); the reaction mixture was then placed in a − 78 °C cold bath. To this solution was added a THF (0.35 M) solution of benzaldehydes (1.0 equiv) over 5 min, and the resulting mixture was warmed to room temperature and stirred for 12 h. After consumption of the starting materials, the reaction was quenched by adding water at 0 °C, and the mixture was extracted with ethyl acetate. The organic layer was dried over Na_2_SO_4_ and evaporated under vacuum to give the crude product that was purified by flash column chromatography on silica gel (petroleum ether/dichloromethane, 1:0–2:1, v/v) to afford **8a–8x** (for details about the structures, overall yields, and *Z:E* ratios, see Additional file 1: Scheme S1).

*5-(3-(4-methoxyphenyl)allyl)benzo[d][1,3]dioxole (****8a,**** 1:5 Z:E)*. yellow wax (477.0 mg, 87% yield); *E* isomer: ^1^H NMR (400 MHz, CDCl_3_) *δ* 7.31 (d, *J* = 8.7 Hz, 2H), 6.85 (d, *J* = 8.7 Hz, 2H), 6.77 (d, *J* = 7.9 Hz, 1H), 6.75 (d, *J* = 1.2 Hz, 1H), 6.71 (d, *J* = 7.8 Hz, 1H), 6.40 (d, *J* = 15.7 Hz, 1H), 6.19 (dt, *J* = 15.7, 6.9 Hz, 1H), 5.93 (s, 2H), 3.81 (s, 3H), 3.45 (d, *J* = 6.8 Hz, 2H).; HRMS (ESI): *m*/*z* [M – H]^–^ calcd for C_17_H_15_O_3_^–^ 267.1027; found: 267.1025.

*1-bromo-2-(3-phenylprop-1-en-1-yl)benzene (****8b****, 3:1 Z:E)*. colorless oil (51.3 mg, 75% yield); *Z* isomer: ^1^H NMR (600 MHz, CDCl_3_) *δ* 7.60 (dd, *J* = 8.0, 0.8 Hz, 1H), 7.31 (dd, *J* = 4.0, 2.4 Hz, 2H), 7.29 (s, 1H), 7.28 (d, *J* = 3.5 Hz, 1H), 7.21 (d, *J* = 6.5 Hz, 2H), 7.19 (s, 1H), 7.12 (td, *J* = 7.8, 1.6 Hz, 1H), 6.61 (d, *J* = 11.3 Hz, 1H), 5.96 (dt, *J* = 11.3, 7.6 Hz, 1H), 3.52 (d, *J* = 7.5 Hz, 2H); HRMS (ESI): *m*/*z* [M – H]^–^ calcd for C_15_H_12_Br^–^ 271.0128; found: 271.0125.

*1-bromo-2-(3-(p-tolyl)prop-1-en-1-yl)benzene (****8c****, 5:4 Z:E)*. colorless oil (85.5 mg, 78% yield); *Z* isomer: ^1^H NMR (500 MHz, CDCl_3_) *δ* 7.60 (d, *J* = 7.8 Hz, 1H), 7.32 (d, *J* = 7.4 Hz, 1H), 7.24 (overlapped, 1H), 7.12 (s, 1H), 7.10 (d, *J* = 3.6 Hz, 3H), 7.08 (d, *J* = 3.9 Hz, 1H), 6.60 (d, *J* = 11.2 Hz, 1H), 5.95 (dt, *J* = 11.3, 7.7 Hz, 1H), 3.48 (d, *J* = 7.5 Hz, 2H), 2.32 (s, 3H); HRMS (ESI): *m*/*z* [M – H]^–^ calcd for C_16_H_14_Br^–^ 285.0284; found: 285.0283.

*1-bromo-2-(3-(4-chlorophenyl)prop-1-en-1-yl)benzene (****8d****, 5:2 Z:E)*. colorless oil (93.5 mg, 79% yield); *Z* isomer: ^1^H NMR (500 MHz, CDCl_3_) *δ* 7.60 (d, *J* = 8.0 Hz, 1H), 7.27 (s, 1H), 7.25 (d, *J* = 3.0 Hz, 2H), 7.18 (d, *J* = 8.4 Hz, 1H), 7.14 (d, *J* = 4.9 Hz, 1H), 7.12 (s, 1H), 7.10 (s, 1H), 6.62 (d, *J* = 11.3 Hz, 1H), 5.91 (dt, *J* = 11.3, 7.6 Hz, 1H), 3.47 (d, *J* = 7.6 Hz, 2H); HRMS (ESI): *m*/*z* [M – H]^–^ calcd for C_15_H_11_ClBr^–^ 304.9738; found: 304.9736.

*1-bromo-2-(3-(4-bromophenyl)prop-1-en-1-yl)benzene (****8e****, 5:2 Z:E)*. colorless oil (86.4 mg, 72% yield); *Z* isomer: ^1^H NMR (500 MHz, CDCl_3_) *δ* 7.61 (d, *J* = 7.9 Hz, 1H), 7.41 (d, *J* = 8.4 Hz, 2H), 7.28 (s, 1H), 7.27 (s, 1H), 7.15 (d, *J* = 4.0 Hz, 1H), 7.07 (d, *J* = 8.4 Hz, 2H), 6.63 (d, *J* = 11.3 Hz, 1H), 5.91 (dt, *J* = 11.3, 7.6 Hz, 1H), 3.46 (d, *J* = 7.6 Hz, 2H); HRMS (ESI): *m*/*z* [M – H]^–^ calcd for C_15_H_11_Br_2_^–^ 348.9233; found: 348.9231.

*1-bromo-2-(3-(o-tolyl)prop-1-en-1-yl)benzene (****8f****, 2:1 Z:E)*. colorless oil (72.2 mg, 64% yield); *Z* isomer: ^1^H NMR (500 MHz, CDCl_3_) *δ* 7.61 (d, *J* = 8.0 Hz, 1H), 7.32 (d, *J* = 7.4 Hz, 1H), 7.28 (d, *J* = 7.3 Hz, 1H), 7.19 (d, *J* = 7.5 Hz, 2H), 7.17 (d, *J* = 2.6 Hz, 1H), 7.14 (s, 2H), 6.63 (d, *J* = 11.3 Hz, 1H), 5.91 (dt, *J* = 11.3, 7.5 Hz, 1H), 3.49 (d, *J* = 7.4 Hz, 2H), 2.18 (s, 3H); HRMS (ESI): *m*/*z* [M – H]^–^ calcd for C_16_H_14_Br^–^ 285.0284; found: 285.0285.

*5-(3-(2,5-bis(benzyloxy)-4-methoxyphenyl)allyl)benzo[d][1,3]dioxole (****8g****, 3:5 Z:E)*. yellow wax (75 mg, 65% yield); *E* isomer: ^1^H NMR (500 MHz, CDCl_3_) *δ* 7.43 (s, 2H), 7.42 (s, 1H), 7.40 (s, 1H), 7.38 (d, *J* = 2.0 Hz, 1H), 7.36 (t, *J* = 1.7 Hz, 1H), 7.34 (s, 2H), 7.31 (s, 1H), 7.30 (s, 1H), 7.03 (s, 1H), 6.74 (d, *J* = 4.4 Hz, 1H), 6.72 (dd, *J* = 4.1, 2.6 Hz, 2H), 6.70–6.66 (m, 1H), 6.54 (s, 1H), 6.10 (dt, *J* = 15.8, 6.9 Hz, 1H), 5.93 (s, 2H), 5.07 (s, 2H), 5.04 (s, 2H), 3.83 (s, 3H), 3.43 (d, *J* = 6.8 Hz, 2H); HRMS (ESI): *m*/*z* [M – H]^–^ calcd for C_31_H_27_O_5_^–^ 479.1864; found: 479.1861.

*1-bromo-3,4,5-trimethoxy-2-(3-phenylprop-1-en-1-yl)benzene (****8h****, 3:2 Z:E)*. colorless oil (314.6 mg, 70% yield); *Z* isomer: ^1^H NMR (500 MHz, CDCl_3_) *δ* 7.31 (s, 1H), 7.29 (d, *J* = 1.7 Hz, 1H), 7.28 (s, 1H), 7.27 (s, 1H), 7.19 (s, 1H), 6.95 (s, 1H), 6.27 (dt, *J* = 11.0, 1.7 Hz, 1H), 5.97 (dt, *J* = 11.0, 7.2 Hz, 1H), 3.88 (s, 3H), 3.86 (s, 3H), 3.78 (s, 3H), 3.32 (dd, *J* = 7.2, 1.2 Hz, 2H); HRMS (ESI): *m*/*z* [M – H]^–^ calcd for C_18_H_18_O_3_Br^–^ 361.0445; found: 361.0444.

*(((2-methoxy-5-(4-phenylbut-1-en-1-yl)-1,4-phenylene)bis(oxy))bis(methylene)) dibenzene (****8i****, 2:3 Z:E)*. yellow wax (47.7 mg, 65% yield); *E* isomer: ^1^H NMR (500 MHz, CDCl_3_) *δ* 7.46 (s, 1H), 7.41 (s, 2H), 7.37 (s, 3H), 7.35 (s, 1H), 7.28 (s, 2H), 7.27 (s, 1H), 7.25 (s, 1H), 7.21 (d, *J* = 7.1 Hz, 3H), 7.14 (d, *J* = 7.1 Hz, 1H), 7.01 (s, 1H), 6.69 (d, *J* = 16.0 Hz, 1H), 6.53 (s, 1H), 6.04 (dt, *J* = 15.9, 6.9 Hz, 1H), 5.09 (s, 2H), 5.02 (s, 2H), 3.82 (s, 3H), 2.78–2.73 (m, 2H), 2.51 (dd, *J* = 14.6, 6.8 Hz, 2H); HRMS (ESI): *m*/*z* [M – H]^–^ calcd for C_31_H_29_O_3_^–^ 449.2122; found: 449.2125.

*1-bromo-3,4,5-trimethoxy-2-(4-phenylbut-1-en-1-yl)benzene (****8j****, 1:1 Z:E)*. colorless oil (80.3 mg, 55% yield); *Z/E* mixture (1:1): ^1^H NMR (500 MHz, CDCl_3_) *δ* 7.29 (t, *J* = 7.4 Hz, 2H), 7.25 (overlapped, 4H), 7.19 (d, *J* = 7.2 Hz, 1H), 7.16 (d, *J* = 7.0 Hz, 3H), 6.90 (d, *J* = 7.4 Hz, 2H), 6.39–6.36 (overlapped, 2H), 6.17 (dt, *J* = 11.1, 1.5 Hz, 1H), 5.84 (dt, *J* = 11.1, 7.2 Hz, 1H), 3.86 (s, 3H), 3.85 (overlapped, 6H), 3.84 (s, 3H), 3.72 (s, 3H), 3.71 (s, 3H), 2.87 – 2.79 (m, 2H), 2.75–2.67 (m, 2H), 2.62–2.53 (m, 2H), 2.32–2.25 (m, 2H). HRMS (ESI): *m*/*z* [M – H]^–^ calcd for C_19_H_20_O_3_Br^–^ 375.0601; found: 375.0603.

*4,4'-(prop-1-ene-1,3-diyl)bis(methoxybenzene) (****8k****, 3:5 Z:E)*. white wax (66.6 mg, 69% yield); *Z* isomer: ^1^H NMR (500 MHz, CDCl_3_) *δ* 7.47–7.44 (m, 1H), 7.30 (t, *J* = 2.9 Hz, 2H), 7.16 (dd, *J* = 5.0, 2.9 Hz, 2H), 6.91 (dd, *J* = 5.6, 3.2 Hz, 1H), 6.88 (d, *J* = 2.1 Hz, 1H), 6.83 (d, *J* = 2.1 Hz, 1H), 6.50 (d, *J* = 11.5 Hz, 1H), 5.75 (dt, *J* = 11.5, 7.5 Hz, 1H), 3.82 (s, 3H), 3.80 (s, 3H), 3.62 (dd, *J* = 7.5, 1.5 Hz, 2H); HRMS (ESI): *m*/*z* [M – H]^–^ calcd for C_17_H_17_O_2_^–^ 253.1234; found: 253.1231.

*1-methyl-2-(3-phenylallyl)benzene (****8l****, 2:1 Z:E)*. colorless oil (64 mg, 60% yield); *Z* isomer: ^1^H NMR (500 MHz, CDCl_3_) *δ* 7.35 (s, 1H), 7.34 (s, 2H), 7.33 (s, 2H), 7.20 (d, *J* = 1.9 Hz, 1H), 7.17 (d, *J* = 2.7 Hz, 1H), 7.16 (d, *J* = 2.9 Hz, 1H), 7.15 (s, 1H), 6.60 (d, *J* = 11.5 Hz, 1H), 5.79 (dt, *J* = 11.5, 7.3 Hz, 1H), 3.64 (dd, *J* = 7.3, 1.5 Hz, 2H), 2.22 (s, 3H); HRMS (ESI): *m*/*z* [M – H]^–^ calcd for C_16_H_15_^–^ 207.1179; found: 207.1175.

*1-(3-(4-methoxyphenyl)allyl)-2-methylbenzene (****8m****, 2:3 Z:E)*. colorless oil (84.9 mg, 71% yield); *E* isomer: ^1^H NMR (500 MHz, CDCl_3_) *δ* 7.28 (s, 1H), 7.27 (s, 1H), 7.17 (d, *J* = 4.8 Hz, 2H), 7.15 (d, *J* = 2.2 Hz, 1H), 7.15 (s, 1H), 6.85–6.80 (m, 2H), 6.32 (d, *J* = 15.8 Hz, 1H), 6.19 (dt, *J* = 15.8, 6.5 Hz, 1H), 3.80 (s, 3H), 3.51 (d, *J* = 6.5 Hz, 2H), 2.34 (s, 3H); HRMS (ESI): *m*/*z* [M – H]^–^ calcd for C_17_H_17_O^–^ 237.1285; found: 237.1288.

*1-chloro-4-(3-phenylallyl)benzene (****8n****, 2:1 Z:E)*. colorless oil (131.3 mg, 77% yield); *Z* isomer: ^1^H NMR (500 MHz, CDCl_3_) *δ* 7.45 (d, *J* = 8.6 Hz, 1H), 7.35 (d, *J* = 3.3 Hz, 2H), 7.34 (d, *J* = 1.8 Hz, 1H), 7.28–7.27 (overlapped, 2H), 7.16 (d, *J* = 3.1 Hz, 1H), 7.14 (s, 1H), 7.13 (d, *J* = 2.6 Hz, 1H), 6.61 (d, *J* = 11.5 Hz, 1H), 5.81 (dt, *J* = 11.5, 7.5 Hz, 1H), 3.46 (d, *J* = 5.5 Hz, 2H); HRMS (ESI): *m*/*z* [M – H]^–^ calcd for C_15_H_12_Cl^–^ 227.0633; found: 227.0630.

*1-chloro-4-(3-(4-methoxyphenyl)allyl)benzene (****8o****, 4:5 Z:E)*. white wax (86 mg, 28% yield); *E* isomer: ^1^H NMR (500 MHz, CDCl_3_) *δ* 7.43 (d, *J* = 8.6 Hz, 1H), 7.31 (d, *J* = 2.7 Hz, 1H), 7.29 (d, *J* = 1.7 Hz, 1H), 7.27 (d, *J* = 1.9 Hz, 1H), 7.17 (d, *J* = 7.9 Hz, 1H), 6.92 (d, *J* = 8.8 Hz, 1H), 6.88 (d, *J* = 8.7 Hz, 1H), 6.84 (d, *J* = 8.8 Hz, 1H), 6.38 (d, *J* = 15.7 Hz, 1H), 6.16 (dt, *J* = 15.7, 6.9 Hz, 1H), 3.84 (s, 3H), 3.63 (d, *J* = 7.4 Hz, 2H); HRMS (ESI): *m*/*z* [M – H]^–^ calcd for C_16_H_14_ClO^–^ 257.0739; found: 257.0740.

*1-(3-phenylallyl)naphthalene (****8p****, 2:1 Z:E)*. yellow oil (58.7 mg, 53% yield); *Z* isomer: ^1^H NMR (500 MHz, CDCl_3_) *δ* 7.90 (d, *J* = 9.0 Hz, 1H), 7.88 (dd, *J* = 6.5, 2.9 Hz, 1H), 7.50 – 7.48 (m, 1H), 7.48 (s, 1H), 7.48–7.46 (m, 1H), 7.43 (d, *J* = 3.4 Hz, 1H), 7.42 (s, 1H), 7.39 (d, *J* = 1.6 Hz, 2H), 7.38 (s, 1H), 7.30 (t, *J* = 2.0 Hz, 1H), 7.28 (s, 1H), 6.65 (d, *J* = 11.5 Hz, 1H), 5.95 (dt, *J* = 11.5, 7.2 Hz, 1H), 4.12 (dd, *J* = 7.2, 1.7 Hz, 2H); HRMS (ESI): *m*/*z* [M – H]^–^ calcd for C_19_H_15_^–^ 243.1179; found: 243.1178.

*1-fluoro-2-(3-phenylallyl)benzene (****8q****, 3:2 Z:E)*. colorless oil (48.9 mg, 59% yield); *Z* isomer: ^1^H NMR (500 MHz, CDCl_3_) *δ* 7.37 (s, 1H), 7.35 (s, 2H), 7.34 (s, 1H), 7.23 (d, *J* = 7.2 Hz, 2H), 7.21 (s, 1H), 7.10 (dd, *J* = 4.5, 3.0 Hz, 1H), 7.08 (d, *J* = 7.5 Hz, 1H), 6.62 (d, *J* = 11.5 Hz, 1H), 5.83 (dt, *J* = 11.5, 7.5 Hz, 1H), 3.70 (d, *J* = 7.4 Hz, 2H); HRMS (ESI): *m*/*z* [M – H]^–^ calcd for C_15_H_12_F^–^ 211.0929; found: 211.0925.

*1-bromo-2-(3-(2-fluorophenyl)prop-1-en-1-yl)benzene (****8r****, 5:1 Z:E)*. colorless oil (36.8 mg, 32% yield); *Z* isomer: ^1^H NMR (500 MHz, CDCl_3_) *δ* 7.61 (dd, *J* = 8.0, 0.9 Hz, 1H), 7.33 (dd, *J* = 7.6, 1.7 Hz, 1H), 7.31–7.28 (m, 1H), 7.20 (s, 1H), 7.19 (s, 1H), 7.15 (dd, *J* = 7.6, 1.7 Hz, 1H), 7.10–7.07 (m, 1H), 7.02 (dd, *J* = 13.4, 4.8 Hz, 1H), 6.63 (d, *J* = 11.3 Hz, 1H), 5.94 (dt, *J* = 11.3, 7.5 Hz, 1H), 3.54 (d, *J* = 7.6 Hz, 2H); HRMS (ESI): *m*/*z* [M – H]^–^ calcd for C_15_H_12_FBr^–^ 290.0112; found: 290.0110.

*1-chloro-2-(3-phenylallyl)benzene (****8s****, 1:1 Z:E)*. colorless oil (56.6 mg, 65% yield); *Z*/*E* mixture (1:1): ^1^H NMR (400 MHz, CDCl_3_) *δ* 7.40–7.31 (overlapped, 6H), 7.30 (s, 2H), 7.28 (s, 2H), 7.26 (overlapped, 1H), 7.24 (s, 2H), 7.18 (overlapped, 5H), 6.63 (d, *J* = 11.5 Hz, 1H), 6.44 (d, *J* = 15.5 Hz, 1H), 6.39–6.27 (m, 1H), 5.86–5.73 (m, 1H), 3.76 (d, *J* = 7.3 Hz, 2H), 3.65 (d, *J* = 6.3 Hz, 2H); HRMS (ESI): *m*/*z* [M – H]^–^ calcd for C_15_H_12_Cl^–^ 227.0633; found: 227.0631.

*1-bromo-2-(3-(2-chlorophenyl)prop-1-en-1-yl)benzene (****8t****, 5:1 Z:E)*. colorless oil (49.8 mg, 54% yield); *Z* isomer: ^1^H NMR (500 MHz, CDCl_3_) *δ* 7.64–7.58 (m, 1H), 7.35 (dd, *J* = 7.8, 1.2 Hz, 1H), 7.30 (d, *J* = 2.3 Hz, 1H), 7.24 (dd, *J* = 7.5, 2.0 Hz, 1H), 7.21 (dd, *J* = 7.2, 1.3 Hz, 1H), 7.18 (dd, *J* = 4.3, 1.9 Hz, 1H), 7.15 (t, *J* = 1.9 Hz, 1H), 7.14 (s, 1H), 6.66 (d, *J* = 11.3 Hz, 1H), 5.94 (dt, *J* = 11.3, 7.5 Hz, 1H), 3.62 (dd, *J* = 7.5, 1.3 Hz, 2H); HRMS (ESI): *m*/*z* [M – H]^–^ calcd for C_15_H_11_ClBr^–^ 304.9738; found: 304.9739.

*1-fluoro-2-(3-(4-methoxyphenyl)allyl)benzene (****8u****, 4:5 Z:E)*. colorless oil (63.5 mg, 67% yield); *E* isomer: ^1^H NMR (500 MHz, CDCl_3_) *δ* 7.29–7.26 (m, 2H), 7.20 (ddd, *J* = 7.3, 6.2, 1.6 Hz, 2H), 7.09 (dd, *J* = 7.5, 1.3 Hz, 2H), 6.84 (dd, *J* = 8.7, 1.8 Hz, 2H), 6.41 (d, *J* = 15.7 Hz, 1H), 6.20 (dtd, *J* = 15.7, 6.9, 1.8 Hz, 1H), 3.80 (d, *J* = 1.2 Hz, 3H), 3.55 (d, *J* = 6.8 Hz, 2H); HRMS (ESI): *m*/*z* [M – H]^–^ calcd for C_16_H_14_OF^–^ 241.1034; found: 241.1036.

*prop-1-ene-1,3-diyldibenzene (****8v****, 5:3 Z:E)*. yellow oil (77.9 mg, 80% yield); *Z* isomer: ^1^H NMR (500 MHz, CDCl_3_) *δ* 7.38 (s, 1H), 7.36 (s, 2H), 7.34 (s, 2H), 7.32 (d, *J* = 3.3 Hz, 2H), 7.24 (s, 2H), 7.22 (d, *J* = 1.7 Hz, 1H), 6.60 (d, *J* = 11.5 Hz, 1H), 5.87 (dt, *J* = 11.5, 7.5 Hz, 1H), 3.69 (d, *J* = 7.5 Hz, 2H); HRMS (ESI): *m*/*z* [M – H]^–^ calcd for C_15_H_13_^–^ 193.1023; found: 193.1022.

*1,2-dimethoxy-4-(3-(4-methoxyphenyl)allyl)benzene (****8w****, 1:2 Z:E)*. white wax (87.8 mg, 72% yield); *E* isomer: ^1^H NMR (500 MHz, CDCl_3_) *δ* 7.30 (d, *J* = 8.7 Hz, 2H), 6.84 (d, *J* = 8.8 Hz, 2H), 6.81 (s, 1H), 6.79 (t, *J* = 2.1 Hz, 1H), 6.76 (d, *J* = 1.7 Hz, 1H), 6.39 (d, *J* = 15.7 Hz, 1H), 6.20 (dt, *J* = 15.7, 6.8 Hz, 1H), 3.87 (s, 3H), 3.87 (s, 3H), 3.80 (s, 3H), 3.47 (d, *J* = 6.7 Hz, 2H); HRMS (ESI): *m*/*z* [M – H]^–^ calcd for C_18_H_19_O_3_^–^ 283.1340, found: 283.1344.

*2-bromo-3-(3-(3-methoxyphenyl)prop-1-en-1-yl)pyridine (****8x****, 5:2 Z:E)*. colorless oil (76.2 mg, 66% yield); *Z* isomer: ^1^H NMR (600 MHz, CDCl_3_) *δ* 8.28 (dd, *J* = 4.5, 1.5 Hz, 1H), 7.59 (dd, *J* = 7.4, 1.3 Hz, 1H), 7.25 (dd, *J* = 7.5, 4.8 Hz, 1H), 7.10 (d, *J* = 8.5 Hz, 2H), 6.85 (d, *J* = 8.5 Hz, 2H), 6.55 (d, *J* = 11.4 Hz, 1H), 6.05 (dt, *J* = 11.3, 7.7 Hz, 1H), 3.79 (s, 3H), 3.44 (d, *J* = 7.6 Hz, 2H); HRMS (ESI): *m*/*z* [M + H]^+^ calcd for C_15_H_15_ONBr^+^ 304.0332, found: 304.0336.

### General procedure for Prins cyclization of 1,3-dioxanes 7

A reaction tube charged with a solution of (CHO)n (50 wt%), TfOH (10 mol%) in freshly distilled DCM (0.1 M) were stirred for 20 min at room temperature. Then the reaction mixture was cooled to 0 ℃ for 5 min, and was added of **8** (0.1 mmol, 1.0 equiv) in the DCM (0.5 M). The reaction was stirred at 0 ℃ for 4 h. Then the reaction mixture was warmed to room temperature for 20 h. The reaction was quenched with NaHCO_3_ saturated aqueous solution, and was extracted with dichloromethane. The combined organic layer was dried over Na_2_SO_4_, filtered, and concentrated to give the crude product that was further purified by flash column chromatography on silica gel (petroleum ether/ethyl acetate, 25:1–2:1, v/v) to afford **7a–7v**, **11a**, **11a′** and **11b**.

*5-((trans-4-(4-methoxyphenyl)-1,3-dioxan-5-yl)methyl)benzo[d][1,3]dioxole (****7a****).* yellow solid (25.9 mg, 79% yield): mp 112.4−113.0 °C; ^1^H NMR (400 MHz, CDCl_3_) *δ* 7.35 (d, *J* = 8.5 Hz, 2H), 6.93 (d, *J* = 8.4 Hz, 2H), 6.66 (d, *J* = 7.8 Hz, 1H), 6.44 (s, 1H), 6.41 (d, *J* = 8.0 Hz, 1H), 5.89 (s, 2H), 5.15 (d, *J* = 6.2 Hz, 1H), 4.80 (d, *J* = 6.3 Hz, 1H), 4.21 (d, *J* = 9.9 Hz, 1H), 3.99 (dd, *J* = 11.4, 4.3 Hz, 1H), 3.83 (s, 3H), 3.43 (t, *J* = 11.1 Hz, 1H), 2.38 (dd, *J* = 13.8, 3.6 Hz, 1H), 2.35–2.23 (m, 1H), 2.00 (dd, *J* = 13.7, 10.8 Hz, 1H); ^13^C NMR (100 MHz, CDCl_3_) *δ* 159.8, 147.7, 146.0, 132.2, 131.5, 129.0, 129.0, 121.6, 114.1, 114.1, 109.1, 108.2, 101.0, 94.3, 84.5, 71.5, 55.4, 42.8, 34.4; HRMS (ESI): *m*/*z* [M + H]^+^ calcd for C_19_H_21_O_5_^+^ 329.1384, found: 329.1383.

*(trans-5-(4-methoxyphenyl)-6,7-dihydro-5H-indeno[5,6-d][1,3]dioxol-6-yl)methanol (****11a****).* yellow solid (3.0 mg, 10% yield): mp 167.1–169.1 ℃; ^1^H NMR (400 MHz, CDCl_3_) *δ* 7.09 (d, *J* = 8.6 Hz, 2H), 6.85 (d, *J* = 8.6 Hz, 2H), 6.72 (s, 1H), 6.34 (s, 1H), 5.89 (dd, *J* = 8.0, 1.0 Hz, 2H), 3.98 (d, *J* = 8.1 Hz, 1H), 3.83–3.76 (overlapped, 4H), 3.71 (dd, *J* = 10.6, 6.7 Hz, 1H), 3.08 (dd, *J* = 15.4, 8.0 Hz, 1H), 2.73 (dd, *J* = 15.4, 8.3 Hz, 1H), 2.65–2.54 (m, 1H); ^13^C NMR (100 MHz, CDCl_3_) *δ* 158.5, 147.0, 146.8, 139.3, 136.7, 135.4, 129.4, 129.4, 114.1, 114.1, 105.7, 105.0, 101.0, 65.3, 55.4, 53.5, 53.2, 35.1; HRMS (ESI): *m*/*z* [M + H]^+^ calcd for C_18_H_19_O_4_^+^ 299.1278, found: 299.1278.

*(cis-5-(4-methoxyphenyl)-6,7-dihydro-5H-indeno[5,6-d][1,3]dioxol-6-yl)methanol (****11a′****).* The product was obtained under the conditions in Table 1***, entry 3***, as a yellow solid (4.2 mg, 14% yield): mp 98.5–100.1 ℃; ^1^H NMR (400 MHz, CDCl_3_) *δ* 7.05 (dd, *J* = 8.5, 4.3 Hz, 2H), 6.83 (d, *J* = 8.5 Hz, 2H), 6.69 (s, 1H), 6.33 (s, 1H), 5.89 (d, *J* = 7.3 Hz, 2H), 4.66 (s, 1H), 3.95 (t, *J* = 7.6 Hz, 1H), 3.79 (s, 3H), 3.70–3.52 (m, 2H), 3.03 (ddd, *J* = 14.9, 7.7, 2.4 Hz, 1H), 2.74–2.56 (overlapped, 2H); ^13^C NMR (100 MHz, CDCl_3_) *δ* 158.4, 146.9, 146.8, 139.2, 136.6, 135.7, 129.4, 129.4, 114.0, 114.0, 105.7, 105.0, 101.0, 69.9, 69.7, 55.4, 53.2, 51.1, 51.0, 35.6; HRMS (ESI): *m*/*z* [M + H]^+^ calcd for C_18_H_19_O_4_^+^: 299.1278; found: 299.1276.

*((trans)-5,6-dimethoxy-1-(4-methoxyphenyl)-2,3-dihydro-1H-inden-2-yl)methanol (****11b****).* yellow wax (15.7 mg, 50% yield); ^1^H NMR (400 MHz, CDCl_3_) *δ* 7.10 (d, *J* = 8.6 Hz, 2H), 6.86 (d, *J* = 8.7 Hz, 2H), 6.80 (s, 1H), 6.41 (s, 1H), 4.04 (d, *J* = 8.0 Hz, 1H), 3.88 (s, 3H), 3.83–3.78 (overlapped, 4H), 3.75–3.70 (overlapped, 4H), 3.12 (dd, *J* = 15.4, 8.1 Hz, 1H), 2.77 (dd, *J* = 15.4, 8.1 Hz, 1H), 2.64–2.49 (m, 1H); ^13^C NMR (100 MHz, CDCl_3_) *δ* 158.4, 148.5, 148.4, 137.9, 136.9, 134.4, 129.4, 129.4, 114.1, 114.1, 108.2, 107.6, 65.4, 56.2, 56.2, 55.4, 53.5, 53.5, 35.1; HRMS (ESI): *m*/*z* [M + H]^+^ calcd for C_19_H_23_O_4_^+^ 315.1591, found: 315.1591.

*trans-5-benzyl-4-(2-bromophenyl)-1,3-dioxane (****7b****).* yellow oil (22.9 mg, 69% yield); ^1^H NMR (500 MHz, CDCl_3_) *δ* 7.63 (dd, *J* = 7.8, 1.5 Hz, 1H), 7.58 (dd, *J* = 8.0, 0.9 Hz, 1H), 7.41 (dd, *J* = 11.0, 4.1 Hz, 1H), 7.20 (td, *J* = 6.7, 2.8 Hz, 3H), 7.15 (t, *J* = 7.3 Hz, 1H), 6.97 (d, *J* = 7.2 Hz, 2H), 5.18 (d, *J* = 6.3 Hz, 1H), 4.96 (d, *J* = 9.9 Hz, 1H), 4.87 (d, *J* = 6.3 Hz, 1H), 3.98 (dd, *J* = 11.5, 4.2 Hz, 1H), 3.54 (t, *J* = 11.1 Hz, 1H), 2.48 (dd, *J* = 13.7, 3.4 Hz, 1H), 2.44–2.35 (m, 1H), 2.28 (dd, *J* = 13.7, 11.0 Hz, 1H); ^13^C NMR (125 MHz, CDCl_3_) *δ* 138.9, 138.4, 132.7, 129.9, 129.2, 128.7, 128.7,128.5, 128.5, 128.3, 126.4, 124.3, 94.4, 82.4, 71.4, 43.8, 34.1; HRMS (ESI): *m*/*z* [M + NH_4_]^+^ calcd for C_17_H_21_ O_2_NBr^+^ 350.0750, found: 350.0751.

*trans-4-(2-bromophenyl)-5-(4-methylbenzyl)-1,3-dioxane (****7c****).* colorless oil (17.7 mg, 51% yield); ^1^H NMR (500 MHz, CDCl_3_) *δ* 7.62 (dd, *J* = 7.8, 1.6 Hz, 1H), 7.58 (dd, *J* = 8.0, 1.1 Hz, 1H), 7.43–7.39 (m, 1H), 7.20 (td, *J* = 7.9, 1.7 Hz, 1H), 7.02 (d, *J* = 7.8 Hz, 2H), 6.85 (d, *J* = 7.9 Hz, 2H), 5.17 (d, *J* = 6.3 Hz, 1H), 4.94 (d, *J* = 9.9 Hz, 1H), 4.86 (d, *J* = 6.3 Hz, 1H), 3.98 (dd, *J* = 11.5, 4.2 Hz, 1H), 3.52 (t, *J* = 11.1 Hz, 1H), 2.44 (dd, *J* = 13.7, 3.4 Hz, 1H), 2.41–2.31 (m, 1H), 2.28 (s, 3H), 2.24 (dd, *J* = 13.7, 11.0 Hz, 1H); ^13^C NMR (125 MHz, CDCl_3_) *δ* 139.0, 135.9, 135.2, 132.8, 129.9, 129.2, 129.2, 129.2, 128.6, 128.6, 128.3, 124.4, 94.4, 82.5, 71.5, 43.8, 33.7, 21.1; HRMS (ESI): *m*/*z* [M – H]^–^ calcd for C_18_H_19_O_2_ClBr^–^ 381.0262, found: 381.0265.

*trans-4-(2-bromophenyl)-5-(4-chlorobenzyl)-1,3-dioxane (****7d****).* colorless oil (24.8 mg, 68% yield); ^1^H NMR (500 MHz, CDCl_3_) *δ* 7.64 – 7.57 (m, 2H), 7.42 (t, *J* = 7.5 Hz, 1H), 7.23 (dd, *J* = 7.8, 1.6 Hz, 1H), 7.21–7.17 (m, 2H), 6.91 (d, *J* = 8.3 Hz, 2H), 5.20 (d, *J* = 6.3 Hz, 1H), 4.96 (d, *J* = 9.7 Hz, 1H), 4.89 (d, *J* = 6.3 Hz, 1H), 3.98 (dd, *J* = 11.5, 4.1 Hz, 1H), 3.54 (t, *J* = 11.0 Hz, 1H), 2.46 (dd, *J* = 13.5, 3.4 Hz, 1H), 2.43–2.33 (m, 1H), 2.29 (dd, *J* = 13.4, 10.7 Hz, 1H); ^13^C NMR (125 MHz, CDCl_3_) *δ* 138.8, 136.9, 132.8, 132.2, 130.0, 130.0, 130.0, 129.2, 128.7, 128.7, 128.3, 124.2, 94.4, 82.36, 71.3, 43.8, 33.5; HRMS (ESI): *m*/*z* [M – H]^–^ calcd for C_17_H_15_O_2_ClBr^–^ 364.9949, found: 364.9948.

*trans-5-(4-bromobenzyl)-4-(2-bromophenyl)-1,3-dioxane (****7e****).* white solid (30 mg, 73% yield): mp 90.9–91.5 °C; ^1^H NMR (500 MHz, CDCl_3_) *δ* 7.58 (ddd, *J* = 13.2, 7.9, 1.3 Hz, 2H), 7.42 – 7.38 (m, 1H), 7.34–7.29 (m, 2H), 7.20 (td, *J* = 7.8, 1.7 Hz, 1H), 6.83 (d, *J* = 8.3 Hz, 2H), 5.17 (d, *J* = 6.3 Hz, 1H), 4.93 (d, *J* = 9.8 Hz, 1H), 4.86 (d, *J* = 6.3 Hz, 1H), 3.95 (dd, *J* = 11.5, 4.1 Hz, 1H), 3.51 (t, *J* = 11.0 Hz, 1H), 2.42 (dd, *J* = 13.5, 3.5 Hz, 1H), 2.33 (ddd, *J* = 13.8, 10.2, 5.0 Hz, 1H), 2.25 (dd, *J* = 13.5, 10.7 Hz, 1H); ^13^C NMR (125 MHz, CDCl_3_) *δ* 138.8, 137.4, 132.8, 131.6, 131.6, 130.4, 130.4, 130.0, 129.2, 128.3, 124.2, 120.2, 94.4, 82.4, 71.2, 43.7, 33.6; HRMS (ESI): *m*/*z* [M + Na]^+^ calcd for C_17_H_15_O_2_Br_2_Na^+^ 432.9409, found: 423.9607.

*trans-4-(2-bromophenyl)-5-(2-methylbenzyl)-1,3-dioxane (****7f****).* yellow oil (21.2 mg, 61% yield); ^1^H NMR (500 MHz, CDCl_3_) *δ* 7.61 (ddd, *J* = 20.9, 7.9, 1.4 Hz, 2H), 7.44–7.39 (m, 1H), 7.23–7.19 (m, 1H), 7.09–7.02 (m, 3H), 6.93–6.89 (m, 1H), 5.19 (d, *J* = 6.3 Hz, 1H), 5.01–4.96 (m, 1H), 4.89 (d, *J* = 6.3 Hz, 1H), 4.01 (dd, *J* = 11.6, 3.1 Hz, 1H), 3.63–3.55 (m, 1H), 2.44 (dd, *J* = 12.9, 7.1 Hz, 1H), 2.30 (overlapped, 2H), 1.96 (s, 3H); ^13^C NMR (125 MHz, CDCl_3_) *δ* 139.1, 136.6, 136.0, 132.7, 130.6, 129.9, 129.6, 129.3, 128.2, 126.6, 125.9, 124.2, 94.4, 82.5, 71.6, 42.8, 31.5, 19.1; HRMS (ESI): *m*/*z* [M – H]^–^ calcd for C_18_H_18_O_2_Br^–^ 345.0496, found: 345.0494.

*5-((trans-4-(2,5-bis(benzyloxy)-4-methoxyphenyl)-1,3-dioxan-5-yl)methyl)benzo [d][1,3]dioxole (****7g****).* colorless oil (9.5 mg, 28% yield); ^1^H NMR (500 MHz, CDCl_3_) *δ* 7.49–7.29 (overlapped, 10H), 7.08 (s, 1H), 6.62 (d, *J* = 7.8 Hz, 1H), 6.56 (s, 1H), 6.32 (s, 1H), 6.30 (d, *J* = 7.9 Hz, 1H), 5.89 (dd, *J* = 4.3, 1.3 Hz, 2H), 5.18 (d, *J* = 12.1 Hz, 1H), 5.13 (s, 1H), 5.10 (d, *J* = 12.3 Hz, 1H), 5.06 (s, 2H), 4.81 (d, *J* = 10.0 Hz, 1H), 4.77 (d, *J* = 6.2 Hz, 1H), 3.91 (dd, *J* = 11.3, 4.2 Hz, 1H), 3.85 (s, 3H), 3.38 (t, *J* = 11.2 Hz, 1H), 2.29 (dd, *J* = 14.0, 3.8 Hz, 1H), 2.19–2.07 (m, 1H), 1.97 (dd, *J* = 14.0, 10.8 Hz, 1H); ^13^C NMR (125 MHz, CDCl_3_) *δ* 151.2, 150.5, 147.7, 146.0, 143.3, 137.5, 137.3, 132.5, 128.8, 128.8, 128.7, 128.7, 128.2, 128.0, 127.7, 127.7, 127.6, 127.6, 121.6, 120.3, 114.6, 109.2, 108.1, 100.9, 100.2, 99.7, 94.4, 72.1, 72.0, 71.6, 56.4, 43.1, 34.1; HRMS (ESI): *m*/*z* [M + H]^+^ calcd for C_33_H_33_O_7_^+^ 541.2221, found: 541.2221.

*trans-5-benzyl-4-(6-bromo-2,3,4-trimethoxyphenyl)-1,3-dioxane (****7h****).* yellow oil (32.1 mg, 76% yield); ^1^H NMR (500 MHz, CDCl_3_) *δ* 7.18 (t, *J* = 7.3 Hz, 2H), 7.11 (t, *J* = 7.3 Hz, 1H), 7.01 (d, *J* = 7.1 Hz, 2H), 6.89 (s, 1H), 5.19 (d, *J* = 6.1 Hz, 1H), 4.92 (d, *J* = 10.1 Hz, 1H), 4.82 (d, *J* = 6.2 Hz, 1H), 4.01 (dd, *J* = 11.3, 4.4 Hz, 1H), 3.94 (s, 3H), 3.85 (s, 3H), 3.83 (s, 3H), 3.44 (t, *J* = 11.1 Hz, 1H), 3.21 (s, 1H), 2.40 (dd, *J* = 14.0, 4.6 Hz, 1H), 2.18 (dd, *J* = 13.9, 9.9 Hz, 1H). ^13^C NMR (125 MHz, CDCl_3_) *δ* 154.4, 154.4, 154.0, 143.0, 138.9, 128.7, 128.7, 128.4, 128.4, 126.1, 124.4, 112.5, 94.6, 83.9, 72.2, 62.1, 60.9, 56.3, 39.1, 34.9; HRMS (ESI): *m*/*z* [M + H]^+^ calcd for C_20_H_24_O_5_Br^+^ 423.0786, found: 423.0786.

*trans-4-(2,5-bis(benzyloxy)-4-methoxyphenyl)-5-phenethyl-1,3-dioxane (****7i****).* yellow wax (34.7 mg, 68% yield); ^1^H NMR (500 MHz, CDCl_3_)* δ* 7.45 (d, *J* = 7.4 Hz, 2H), 7.39–7.27 (m, 8H), 7.20 (t, *J* = 7.4 Hz, 2H), 7.13 (t, *J* = 7.3 Hz, 1H), 6.99 (s, 1H), 6.95 (d, *J* = 7.3 Hz, 2H), 6.55 (s, 1H), 5.14 (d, *J* = 6.2 Hz, 1H), 5.11–4.98 (m, 4H), 4.82–4.75 (m, 2H), 4.23 (dd, *J* = 11.3, 4.4 Hz, 1H), 3.83 (s, 3H), 3.46 (t, *J* = 11.1 Hz, 1H), 2.45–2.35 (m, 1H), 2.21 (ddd, *J* = 13.9, 10.0, 6.6 Hz, 1H), 2.04–1.94 (m, 1H), 1.41–1.31 (m, 1H), 1.25–1.14 (m, 1H); ^13^C NMR (125 MHz, CDCl_3_) *δ* 151.2, 150.4, 143.3, 141.9, 137.5, 137.3, 128.7, 128.7, 128.6, 128.6, 128.4, 128.4, 128.3, 128.3, 128.1, 127.9, 127.8, 127.8, 127.6, 127.6, 126.0, 120.5, 114.6, 99.7, 94.4, 75.5, 72.0, 72.0, 71.9, 56.4, 40.8, 32.8, 29.3; HRMS (ESI): *m*/*z* [M + H]^+^ calcd for C_33_H_35_O_5_^+^ 511.2478, found: 511.2478.

*trans-4-(6-bromo-2,3,4-trimethoxyphenyl)-5-phenethyl-1,3-dioxane (****7j****)*. colorless oil (22.9 mg, 50% yield); ^1^H NMR (600 MHz, CDCl_3_) *δ* 7.21 (t, *J* = 7.5 Hz, 2H), 7.13 (t, *J* = 7.4 Hz, 1H), 7.03 (d, *J* = 7.3 Hz, 2H), 6.89 (s, 1H), 5.19 (d, *J* = 6.1 Hz, 1H), 4.87 (d, *J* = 10.2 Hz, 1H), 4.81 (d, *J* = 6.2 Hz, 1H), 4.28 (dd, *J* = 11.2, 4.5 Hz, 1H), 3.86 (s, 3H), 3.85 (s, 3H), 3.84 (s, 3H), 3.45 (t, *J* = 11.1 Hz, 1H), 2.94–2.83 (m, 1H), 2.49 (ddd, *J* = 14.1, 10.4, 5.6 Hz, 1H), 2.30 (ddd, *J* = 13.8, 10.2, 6.6 Hz, 1H), 1.42–1.32 (m, 1H), 1.34–1.26 (m, 1H); ^13^C NMR (150 MHz, CDCl_3_) *δ* 154.6, 154.5, 154.0, 142.9, 142.0, 128.5, 128.5, 128.3, 128.3, 126.0, 124.6, 111.9, 94.5, 84.5, 72.2, 61.9, 60.9, 56.3, 37.2, 32.9, 30.1; HRMS (ESI): *m*/*z* [M + H]^+^ calcd for C_21_H_26_O_5_Br^+^ 437.0958, found: 437.0955.

*trans-5-(4-methoxybenzyl)-4-(4-methoxyphenyl)-1,3-dioxane (****7k****).* white solid (21.7 mg, 69% yield): mp 113.6–115.6 °C; ^1^H NMR (500 MHz, CDCl_3_) *δ* 7.40 – 7.33 (m, 2H), 6.94 (d, *J* = 8.7 Hz, 2H), 6.88 (d, *J* = 8.6 Hz, 2H), 6.76 (d, *J* = 8.6 Hz, 2H), 5.16 (d, *J* = 6.3 Hz, 1H), 4.81 (d, *J* = 6.3 Hz, 1H), 4.23 (d, *J* = 10.0 Hz, 1H), 3.98 (dd, *J* = 11.5, 4.3 Hz, 1H), 3.83 (s, 3H), 3.76 (s, 3H), 3.44 (t, *J* = 11.2 Hz, 1H), 2.42 (dd, *J* = 13.9, 3.6 Hz, 1H), 2.37–2.26 (m, 1H), 2.03 (dd, *J* = 13.9, 10.8 Hz, 1H); ^13^C NMR (125 MHz, CDCl_3_) *δ* 159.9, 158.2, 131.7, 130.5, 129.7, 129.7, 129.0, 129.0, 114.2, 114.2, 114.0, 114.0, 94.3, 84.6, 71.6, 55.5, 55.4, 42.8, 33.8; HRMS (ESI): *m*/*z* [M + Na]^+^ calcd for C_19_H_22_O_4_Na^+^ 337.1410, found: 337.1411.

*trans-5-(2-methylbenzyl)-4-phenyl-1,3-dioxane (****7l****).* white wax (17.1 mg, 64% yield); ^1^H NMR (500 MHz, CDCl_3_) *δ* 7.47 (d, *J* = 7.0 Hz, 2H), 7.41 (t, *J* = 7.2 Hz, 2H), 7.39–7.34 (m, 1H), 7.09–7.03 (overlapped, 3H), 6.94–6.89 (m, 1H), 5.20 (d, *J* = 6.2 Hz, 1H), 4.87 (d, *J* = 6.2 Hz, 1H), 4.33 (d, *J* = 9.9 Hz, 1H), 3.99 (dd, *J* = 11.4, 4.4 Hz, 1H), 3.53 (t, *J* = 11.1 Hz, 1H), 2.46 (dd, *J* = 13.9, 3.2 Hz, 1H), 2.38–2.25 (m, 1H), 2.12 (dd, *J* = 13.8, 11.5 Hz, 1H), 1.97 (s, 3H); ^13^C NMR (125 MHz, CDCl_3_) *δ* 139.4, 136.6, 136.1, 130.6, 129.7, 128.7, 128.7, 128.7, 127.8, 127.8, 126.5, 125.9, 94.3, 85.3, 71.7, 41.6, 32.0, 19.1; HRMS (ESI): *m*/*z* [M + NH_4_]^+^ calcd for C_18_H_24_O_2_N^+^ 286.1802, found: 286.1806.

*trans-4-(4-methoxyphenyl)-5-(2-methylbenzyl)-1,3-dioxane (****7 m****).* colorless oil (20.0 mg, 67% yield); ^1^H NMR (500 MHz, CDCl_3_) *δ* 7.38 (d, *J* = 8.6 Hz, 2H), 7.08–7.02 (overlapped, 3H), 6.93 (d, *J* = 8.6 Hz, 2H), 6.91 (d, *J* = 6.1 Hz, 1H), 5.17 (d, *J* = 6.2 Hz, 1H), 4.84 (d, *J* = 6.2 Hz, 1H), 4.26 (d, *J* = 9.9 Hz, 1H), 3.96 (dd, *J* = 11.5, 4.3 Hz, 1H), 3.83 (s, 3H), 3.51–3.47 (m, 1H), 2.45 (dd, *J* = 13.9, 3.2 Hz, 1H), 2.35–2.25 (m, 1H), 2.09 (dd, *J* = 13.8, 11.5 Hz, 1H), 2.00 (s, 3H).; ^13^C NMR (125 MHz, CDCl_3_) *δ* 159.9, 136.8, 136.1, 131.70, 130.6, 129.7, 129.0, 129.0, 126.5, 125.9, 114.1, 114.1, 94.3, 84.9, 71.8, 55.5, 41.6, 32.1, 19.3; HRMS (ESI): *m*/*z* [M + K]^+^ calcd for C_19_H_22_O_3_K^+^ 337.1201, found: 337.1200.

*trans-5-(4-chlorobenzyl)-4-phenyl-1,3-dioxane (****7n****).* white solid (16.1 mg, 56% yield): mp 100.7–102.0 °C; ^1^H NMR (500 MHz, CDCl_3_) *δ* 7.45–7.33 (overlapped, 5H), 7.18 (d, *J* = 8.4 Hz, 2H), 6.88 (d, *J* = 8.3 Hz, 2H), 5.18 (d, *J* = 6.3 Hz, 1H), 4.83 (d, *J* = 6.3 Hz, 1H), 4.28 (d, *J* = 9.9 Hz, 1H), 3.95 (dd, *J* = 11.5, 4.3 Hz, 1H), 3.45 (t, *J* = 11.1 Hz, 1H), 2.43 (dd, *J* = 13.9, 3.7 Hz, 1H), 2.34 (dt, *J* = 10.0, 4.0 Hz, 1H), 2.09 (dd, *J* = 13.9, 10.8 Hz, 1H); ^13^C NMR (125 MHz, CDCl_3_) *δ* 139.2, 136.9, 132.2, 130.1, 130.1, 128.8, 128.8, 128.8, 128.7, 128.7, 127.8, 127.8, 94.3, 85.0, 71.3, 42.6, 34.0; HRMS (ESI): *m*/*z* [M + K]^+^ calcd for C_17_H_17_O_2_ClK^+^ 327.0549, found: 327.0547.

*trans-5-(4-chlorobenzyl)-4-(4-methoxyphenyl)-1,3-dioxane (****7o****).* white solid (20.3 mg, 64% yield): mp 123.5 − 126.9 °C; ^1^H NMR (500 MHz, CDCl_3_) *δ* 7.34 (d, *J* = 8.7 Hz, 2H), 7.18 (d, *J* = 8.4 Hz, 2H), 6.93 (d, *J* = 8.7 Hz, 2H), 6.88 (d, *J* = 8.3 Hz, 2H), 5.15 (d, *J* = 6.2 Hz, 1H), 4.81 (d, *J* = 6.3 Hz, 1H), 4.22 (d, *J* = 9.9 Hz, 1H), 3.95 (dd, *J* = 11.4, 4.4 Hz, 1H), 3.83 (s, 3H), 3.44 (t, *J* = 11.1 Hz, 1H), 2.43 (dd, *J* = 13.9, 3.7 Hz, 1H), 2.31 (ddt, *J* = 10.6, 8.3, 5.2 Hz, 1H), 2.07 (dd, *J* = 13.9, 10.7 Hz, 1H); ^13^C NMR (125 MHz, CDCl_3_) δ 160.0, 137.0, 132.2, 131.4, 130.1, 130.1, 129.0, 129.0, 128.7, 128.7, 114.2, 114.2, 94.3, 84.5, 71.4, 55.5, 42.69, 34.1; HRMS (ESI): *m*/*z* [M – H]^–^ calcd for C_18_H_18_O_3_Cl^–^ 317.0950, found: 317.0951.

*trans-5-(naphthalen-1-ylmethyl)-4-phenyl-1,3-dioxane (****7p****).* yellow wax (4.2 mg, 14% yield); ^1^H NMR (400 MHz, CDCl_3_) *δ* 7.81 (d, *J* = 8.1 Hz, 1H), 7.69 (d, *J* = 8.2 Hz, 1H), 7.56 (dd, *J* = 8.1, 1.3 Hz, 2H), 7.52 – 7.47 (m, 2H), 7.45 (dt, *J* = 5.0, 1.8 Hz, 1H), 7.41 (dd, *J* = 8.2, 1.3 Hz, 1H), 7.32 (ddd, *J* = 8.9, 6.8, 1.9 Hz, 2H), 7.26 (d, *J* = 8.3 Hz, 1H), 7.13 (d, *J* = 6.9 Hz, 1H), 5.19 (d, *J* = 6.2 Hz, 1H), 4.88 (d, *J* = 6.2 Hz, 1H), 4.42 (d, *J* = 9.4 Hz, 1H), 3.90 (dd, *J* = 11.3, 3.8 Hz, 1H), 3.61–3.52 (m, 1H), 3.01 (d, *J* = 11.3 Hz, 1H), 2.56–2.41 (overlapped, 2H); ^13^C NMR (100 MHz, CDCl_3_) *δ* 139.5, 134.5, 134.1, 131.7, 128.9, 128.9, 128.8, 128.8, 128.0, 128.0, 127.4, 126.9, 126.0, 125.7, 125.2, 123.7, 94.2, 85.2, 71.9, 41.9, 31.8; HRMS (ESI): *m*/*z* [M + NH_4_]^+^ calcd for C_21_H_24_O_2_N^+^ 322.1802, found: 322.1805.

*trans-5-(2-fluorobenzyl)-4-phenyl-1,3-dioxane (****7q****).* colorless oil (23.9 mg, 88% yield); ^1^H NMR (500 MHz, CDCl_3_) *δ* 7.45 (d, *J* = 7.0 Hz, 2H), 7.40 (t, *J* = 7.3 Hz, 2H), 7.38–7.33 (m, 1H), 7.14 (td, *J* = 7.4, 1.8 Hz, 1H), 7.01–6.88 (overlapped, 3H), 5.18 (d, *J* = 6.2 Hz, 1H), 4.85 (d, *J* = 6.2 Hz, 1H), 4.32 (d, *J* = 9.8 Hz, 1H), 3.98 (dd, *J* = 11.5, 4.2 Hz, 1H), 3.53 (t, *J* = 11.2 Hz, 1H), 2.42 (overlapped, 2H), 2.27 (dd, *J* = 13.8, 11.0 Hz, 1H); ^13^C NMR (125 MHz, CDCl_3_) *δ* 162.1, 160.1, 139.1, 131.0, 131.0, 128.7, 128.7, 128.2, 128.2, 127.9, 125.5, 125.4, 124.1, 124.1, 115.5, 115.3, 94.3, 85.2, 71.3, 41.7, 27.5, 27.5; ^19^F NMR (376 MHz, CDCl_3_) *δ* -117.6; HRMS (ESI): *m*/*z* [M + NH_4_]^+^ calcd for C_17_H_21_O_2_NF^+^ 290.1551, found: 290.1551.

*trans-4-(2-bromophenyl)-5-(2-fluorobenzyl)-1,3-dioxane (****7r****).* yellow oil (25.6 mg, 73% yield); ^1^H NMR (500 MHz, CDCl_3_) *δ* 7.59 (ddd, *J* = 16.4, 7.9, 1.3 Hz, 2H), 7.40 (t, *J* = 7.1 Hz, 1H), 7.20 (td, *J* = 7.9, 1.7 Hz, 1H), 7.16–7.10 (m, 1H), 6.99–6.90 (m, 3H), 5.17 (d, *J* = 6.3 Hz, 1H), 4.96 (d, *J* = 9.3 Hz, 1H), 4.88 (d, *J* = 6.3 Hz, 1H), 3.98 (dd, *J* = 11.4, 3.1 Hz, 1H), 3.59 (dd, *J* = 13.8, 7.6 Hz, 1H), 2.41 (overlapped, 3H); ^13^C NMR (125 MHz, CDCl_3_) *δ* 162.3, 159.9, 138.6, 132.7, 131.0, 131.0 130.0, 129.3, 128.3, 128.2, 125.5, 125.4, 124.4, 124.1, 124.1, 115.5, 115.3, 94.4, 82.4, 71.2, 42.7, 27.3; ^19^F NMR (376 MHz, CDCl_3_) *δ*-117.7; HRMS (ESI): *m*/*z* [M + Na]^+^ calcd for C_17_H_16_O_2_FBrNa^+^ 373.0210, found: 373.0210.

*trans-5-(2-chlorobenzyl)-4-phenyl-1,3-dioxane (****7s****).* white solid (24.8 mg, 86% yield): mp 61.6–62.0 °C; ^1^H NMR (500 MHz, CDCl_3_) *δ* 7.46 (d, *J* = 7.0 Hz, 2H), 7.39 (t, *J* = 7.3 Hz, 2H), 7.36 (d, *J* = 7.2 Hz, 1H), 7.27 (dd, *J* = 5.7, 3.5 Hz, 1H), 7.11 – 7.07 (m, 2H), 6.94 (dd, *J* = 5.7, 3.6 Hz, 1H), 5.19 (d, *J* = 6.2 Hz, 1H), 4.86 (d, *J* = 6.2 Hz, 1H), 4.33 (d, *J* = 9.6 Hz, 1H), 3.96 (dd, *J* = 11.4, 4.0 Hz, 1H), 3.57 (t, *J* = 11.0 Hz, 1H), 2.56–2.46 (overlapped, 2H), 2.35 (dd, *J* = 14.5, 11.7 Hz, 1H); ^13^C NMR (125 MHz, CDCl_3_) *δ* 139.0, 136.4, 134.1, 130.9, 129.8, 128.7, 128.7, 128.7, 127.9, 127.9, 127.9, 126.8, 94.3, 85.3, 71.3, 41.4, 32.1; HRMS (ESI): *m*/*z* [M + H]^+^ calcd for C_17_H_18_O_2_Cl^+^ 289.0990, found: 289.0990.

*trans-4-(2-bromophenyl)-5-(2-chlorobenzyl)-1,3-dioxane (****7t****).* yellow oil (25.6 mg, 70% yield); ^1^H NMR (500 MHz, CDCl_3_) *δ* 7.62 (dd, *J* = 7.8, 1.6 Hz, 1H), 7.58 (dd, *J* = 8.0, 1.1 Hz, 1H), 7.40–7.36 (m, 1H), 7.27 (dd, *J* = 3.2, 2.3 Hz, 1H), 7.21 – 7.17 (m, 1H), 7.12–7.06 (m, 2H), 6.97–6.94 (m, 1H), 5.18 (d, *J* = 6.3 Hz, 1H), 4.99 (d, *J* = 9.3 Hz, 1H), 4.89 (d, *J* = 6.3 Hz, 1H), 3.97 (dd, *J* = 11.3, 3.1 Hz, 1H), 3.66–3.58 (m, 1H), 2.58–2.43 (overlapped, 3H); ^13^C NMR (125 MHz, CDCl_3_) *δ* 138.6, 136.3, 134.0, 132.6, 131.0, 130.0, 129.8, 129.5, 128.2, 128.0, 126.7, 124.3, 94.4, 82.4, 71.3, 42.2, 31.8; HRMS (ESI): *m*/*z* [M + H]^+^ calcd for C_17_H_17_O_2_ClBr^+^ 367.0095, found: 367.0095.

*trans-5-(2-fluorobenzyl)-4-(4-methoxyphenyl)-1,3-dioxane (****7u****).* yellow oil (19.9 mg, 66% yield); ^1^H NMR (500 MHz, CDCl_3_) *δ* 7.37 (d, *J* = 8.7 Hz, 2H), 7.17–7.11 (m, 1H), 7.00–6.96 (m, 1H), 6.96–6.91 (overlapped, 4H), 5.16 (d, *J* = 6.2 Hz, 1H), 4.83 (d, *J* = 6.2 Hz, 1H), 4.26 (d, *J* = 9.8 Hz, 1H), 3.97 (dd, *J* = 11.4, 4.2 Hz, 1H), 3.83 (s, 3H), 3.51 (t, *J* = 11.1 Hz, 1H), 2.40 (overlapped, 2H), 2.23 (dd, *J* = 14.2, 11.3 Hz, 1H); ^13^C NMR (125 MHz, CDCl_3_) *δ* 162.1, 160.1, 159.9, 131.4, 131.0, 131.0, 129.1, 128.2, 128.1, 125.6, 125.5, 124.1, 124.1, 115.5, 115.3, 114.2, 94.3, 84.7, 71.4, 55.4, 41.7, 27.6; ^19^F NMR (376 MHz, CDCl_3_) *δ* − 117.5; HRMS (ESI): *m*/*z* [M + H]^+^ calcd for C_18_H_20_O_3_F^+^ 303.1391; found: 303.1391.

*trans-5-benzyl-4-phenyl-1,3-dioxane (****7v****).* white wax (19.4 mg, 77% yield); ^1^H NMR (500 MHz, CDCl_3_) *δ* 7.49–7.44 (m, 2H), 7.42 (t, *J* = 7.4 Hz, 2H), 7.37 (dd, *J* = 11.4, 4.2 Hz, 1H), 7.22 (t, *J* = 7.3 Hz, 2H), 7.16 (t, *J* = 7.3 Hz, 1H), 6.97 (d, *J* = 7.2 Hz, 2H), 5.19 (d, *J* = 6.2 Hz, 1H), 4.84 (d, *J* = 6.3 Hz, 1H), 4.31 (d, *J* = 9.9 Hz, 1H), 3.99 (dd, *J* = 11.5, 4.4 Hz, 1H), 3.48 (t, *J* = 11.2 Hz, 1H), 2.49 (dd, *J* = 13.8, 3.6 Hz, 1H), 2.44–2.33 (m, 1H), 2.11 (dd, *J* = 13.8, 10.9 Hz, 1H); ^13^C NMR (125 MHz, CDCl_3_) *δ* 139.3, 138.4, 128.8, 128.8, 128.8, 128.8, 128.7, 128.5, 128.5, 127.8, 127.8, 126.4, 94.3, 85.1, 71.5, 42.6, 34.6; HRMS (ESI): *m*/*z* [M – H]^–^ calcd for C_17_H_17_O_2_^–^ 253.1234, found: 235.1235.

### Procedure for preparation of 11a-1

To a solution of **11a** (20 mg, 0.067 mmol) in dry DMF (0.67 mL) was added NaH (4 mg, 0.101 mmol) at 0 ℃ and stirred for 30 min. The mixture was then allowed to warm up to room temperature, followed by the addition of MeI (4 mg, 0.101 mmol). The reaction mixture was stirred for 15 h. The reaction diluted with water and extracted with ethyl acetate. The organic layer was dried over Na_2_SO_4_ and evaporated under vacuum to give the crude product that was purified by flash column chromatography on silica gel (petroleum ether/ethyl acetate, 10:2, v/v) to afford **11a-1**.

*trans-6-(methoxymethyl)-5-(4-methoxyphenyl)-6,7-dihydro-5H-indeno[5,6-d][1,3]dioxole (****11a-1****).* white solid (18.5 mg, 88% yield): mp 156.7–157.5 ℃; ^1^H NMR (400 MHz, CDCl_3_) *δ* 7.08 (d, *J* = 8.6 Hz, 2H), 6.85 (d, *J* = 8.6 Hz, 2H), 6.71 (s, 1H), 6.34 (s, 1H), 5.89 (d, *J* = 7.6 Hz, 2H), 3.95 (d, *J* = 8.0 Hz, 1H), 3.80 (s, 3H), 3.49 (dd, *J* = 9.2, 5.1 Hz, 1H), 3.42 (t, *J* = 8.3 Hz, 1H), 3.33 (s, 3H), 3.07 (dd, *J* = 15.4, 7.9 Hz, 1H), 2.73 (dd, *J* = 15.4, 8.1 Hz, 1H), 2.68–2.55 (m, 1H); ^13^C NMR (100 MHz, CDCl_3_) *δ* 158.4, 146.9, 146.7, 139.2, 136.7, 135.8, 129.4, 129.4, 114.0, 114.0, 105.7, 105.0, 101.0, 75.0, 59.0, 55.4, 53.1, 50.9, 35.6; HRMS (ESI): *m*/*z* [M + H]^+^ calcd for C_19_H_21_O_4_^+^ 313.1434, found: 313.1434.

### Procedure for preparation of 6b, *trans*-6h, and *cis*-6h

Compound **7b** or **7h** (0.33 mmol, 1.0 equiv) was treated at 0 °C with glacial acetic acid added dropwise (5.0 equiv) and trifluoroacetic anhydride added dropwise (5.0 equiv). The reaction mixture was allowed to warm to room temperature and stirred for 2 h. The reaction was stopped by adding a cold saturated aqueous solution of NaHCO_3_ and then extracted with dichloromethane. The combined organic layer was dried over Na_2_SO_4_, filtered, and concentrated to obtain the crude product. The crude product was dissolved in a 1*N* solution of NaOH (MeOH/H_2_O = 9:1, 0.2 M) and stirred for 40 min. After consumption of the starting materials, the reaction was quenched by adding water at 0 °C, and the mixture was extracted with ethyl acetate. The reaction mixture was then purified by flash chromatography (petroleum ether/dichloromethane/ethyl acetate, 20:10:2, v/v) to yield **6b**, *trans*-**6h**, and *cis*-**6h**.

*trans-2-benzyl-1-(2-bromophenyl)propane-1,3-diol* (***6b***). light yellow oil (99.2 mg, quantitative yield); ^1^H NMR (400 MHz, CDCl_3_) *δ* 7.65 (dd, *J* = 7.8, 1.1 Hz, 1H), 7.51 (d, *J* = 8.0 Hz, 1H), 7.37 (t, *J* = 7.5 Hz, 1H), 7.31–7.17 (overlapped, 5H), 7.14 (td, *J* = 7.8, 1.4 Hz, 1H), 5.17 (t, *J* = 4.1 Hz, 1H), 3.71 (d, *J* = 11.1 Hz, 1H), 3.53 (d, *J* = 11.0 Hz, 1H), 3.48 (d, *J* = 5.1 Hz, 1H), 2.93 (qd, *J* = 13.6, 7.8 Hz, 2H), 2.50 (s, 1H), 2.24–2.15 (m, 1H); ^13^C NMR (100 MHz, CDCl_3_) *δ* 142.5, 140.1, 133.0, 129.4, 129.4, 129.1, 128.5, 128.5 128.1, 127.7, 126.3, 122.2, 76.8, 62.3, 45.9, 35.2; HRMS (ESI): *m*/*z* [M + Cl]^–^ calcd for C_16_H_17_O_2_ClBr^–^ 335.0106, found: 335.0104.

*trans-2-benzyl-1-(6-bromo-2,3,4-trimethoxyphenyl)propane-1,3-diol* (*trans*-***6h***). yellow oil (51 mg, 52% yield); ^1^H NMR (400 MHz, CDCl_3_) *δ* 7.20 (t, *J* = 7.4 Hz, 2H), 7.13 (t, *J* = 7.3 Hz, 1H), 7.02 (d, *J* = 7.2 Hz, 2H), 6.87 (s, 1H), 5.16 (t, *J* = 8.6 Hz, 1H), 4.01 (s, 3H), 3.89 (overlapped, 1H), 3.85 (s, 3H), 3.82 (s, 3H), 3.76 (d, *J* = 9.9 Hz, 1H), 3.71 (dd, *J* = 11.1, 5.6 Hz, 1H), 2.92 (s, 1H), 2.55–2.36 (overlapped, 3H); ^13^C NMR (100 MHz, CDCl_3_) *δ* 153.7, 153.0, 141.9, 140.3, 129.0, 129.0, 128.4, 128.4, 126.9, 126.1, 117.8, 112.1, 78.0, 64.8, 61.9, 60.9, 56.3, 48.5, 34.4; HRMS (ESI): *m*/*z* [M + Na]^+^ calcd for C_19_H_23_ O_5_BrNa^+^ 433.0621, found: 433.0625.

*cis-2-benzyl-1-(6-bromo-2,3,4-trimethoxyphenyl)propane-1,3-diol* (cis-***6h***). yellow oil (32.5 mg, 33% yield); ^1^H NMR (400 MHz, CDCl_3_) *δ* 7.32–7.22 (overlapped, 4H), 7.19 (t, *J* = 6.9 Hz, 1H), 6.88 (s, 1H), 5.12 (t, *J* = 8.8 Hz, 1H), 4.02 (s, 3H), 3.85 (s, 3H), 3.83 (overlapped, 4H), 3.43 (s, 2H), 3.10 (dd, *J* = 13.4, 3.2 Hz, 1H), 2.92–2.78 (m, 1H), 2.34–2.23 (m, 1H), 1.89 (s, 1H); ^13^C NMR (100 MHz, CDCl_3_) *δ* 153.4, 152.8, 141.8, 140.7, 129.5, 129.5, 128.5, 128.5, 126.6, 126.0, 116.5, 112.4, 76.1, 62.2, 62.0, 60.9, 56.3, 48.8, 32.8; HRMS (ESI): *m*/*z* [M + Na]^+^ calcd for C_19_H_23_O_5_BrNa^+^ 433.0621, found: 433.0623.

### Procedure for preparation of 12a and 12b through UM reaction

A Schlenk tube was equipped with a magnetic stirring bar, and loaded with Cs_2_CO_3_ (200 mol %), CuI (20 mol %), ethane-1,2-diamine (22 mol %), **6b** (1.0 equiv) and 1,4-dioxane (0.1 M) under air. The tube was sealed, evacuated, and refilled with argon. The reaction mixture was stirred at 120 °C for 12 h. Afterward, The mixture was filtered and the solid was washed with ethyl acetate, and the filtrates were concentrated under reduced pressure. The residue was purified by silica gel chromatography (petroleum ether/dichloromethane/ethyl acetate, 60:10:2—10:10:2 v/v) to give the product **12a** and **12b**.

*trans-3-benzylchroman-4-ol* (***12a***). white solid (4.8 mg, 21% yield, 34% brsm): mp 125.6 − 128.2 °C; ^1^H NMR (400 MHz, CDCl_3_) *δ* 7.36–7.31 (overlapped, 2H), 7.26 (overlapped, 3H), 7.24–7.18 (overlapped, 2H), 6.89 (t, *J* = 7.4 Hz, 1H), 6.85 (d, *J* = 8.6 Hz, 1H), 4.52 (d, *J* = 2.9 Hz, 1H), 4.11 (d, *J* = 2.8 Hz, 1H), 4.09 (s, 1H), 2.89 (dd, *J* = 13.6, 8.4 Hz, 1H), 2.73–2.62 (m, 1H), 2.39–2.27 (m, 1H), 1.68 (s, 1H); ^13^C NMR (100 MHz, CDCl_3_) *δ* 154.5, 139.3, 130.3, 130.1, 129.3, 129.3, 128.7, 128.7, 126.5, 124.3, 120.7, 117.1, 65.1, 65.1, 40.1, 33.0; HRMS (ESI): *m*/*z* [M – H]^–^ calcd for C_16_H_15_O_2_^–^ 239.1078; found: 239.1075.

*trans-2-benzyl-1-phenylpropane-1,3-diol* (***12b***). white solid (3.9 mg, 17% yield, 20% brsm): mp 66.3 − 69.8 °C; ^1^H NMR (400 MHz, CDCl_3_) *δ* 7.37 (s, 2H), 7.36 (s, 2H), 7.32–7.27 (m, 1H), 7.25 (d, *J* = 7.5 Hz, 2H), 7.18 (t, *J* = 7.3 Hz, 1H), 7.13 (d, *J* = 7.4 Hz, 2H), 4.76 (d, *J* = 6.1 Hz, 1H), 3.75 (dd, *J* = 11.0, 2.1 Hz, 1H), 3.56 (dd, *J* = 11.0, 5.5 Hz, 1H), 3.18 (s, 1H), 2.71 (overlapped, 2H), 2.59 (dd, *J* = 13.8, 9.5 Hz, 1H), 2.10 (ddd, *J* = 8.9, 5.9, 2.9 Hz, 1H); ^13^C NMR (100 MHz, CDCl_3_) *δ* 143.5, 140.2, 129.2, 129.2, 128.6, 128.6, 128.5, 128.5, 127.8, 126.4, 126.4, 126.2, 78.1, 63.3, 48.5, 34.9; HRMS (ESI): *m*/*z* [M + Cl]^–^ calcd for C_16_H_18_O_2_Cl^–^ 277.1001, found: 277.1001.

### Procedure for preparation of 14a, 14b and 15

A solution of **12a** (1.0 equiv) in 17% HCl–MeOH (1:1, 0.05 M) was heated at 90 °C for 1 h. After being cooled to room temperature, the reaction mixture was concentrated under reduced pressure to give the crude residue, diluted with dichloromethane and water, and the layers were separated. The water layer was extracted with dichloromethane. The combined organic layers were dried over Na_2_SO_4_ and concentrated in vacuo to give the residue which was purified by silica gel column chromatography (petroleum ether/dichloromethane, 8:1–0:1,v/v) to give **14a**, **14b** and **15**.

*cis-3-benzyl-4-methoxychromane* (***14a***). yellow oil (2.1 mg, 16.5% yield); ^1^H NMR (400 MHz, CDCl_3_) *δ* 7.33 (overlapped, 2H), 7.22 (overlapped, 4H), 7.13–7.09 (m, 1H), 6.85 (d, *J* = 7.7 Hz, 2H), 4.20 (t, *J* = 10.7 Hz, 1H), 4.09 (ddd, *J* = 10.6, 3.9, 0.7 Hz, 1H), 3.92 (d, *J* = 2.9 Hz, 1H), 3.38 (s, 3H), 2.86 (dd, *J* = 13.5, 8.4 Hz, 1H), 2.66 (dd, *J* = 13.5, 7.2 Hz, 1H), 2.35 (tdd, *J* = 11.1, 7.1, 3.7 Hz, 1H); ^13^C NMR (100 MHz, CDCl_3_) *δ* 154.6, 139.6, 130.7, 130.0, 129.3, 129.3, 128.6, 128.6, 126.3, 121.2, 119.4, 117.0, 73.7, 65.8, 56.1, 39.3, 32.9; HRMS (ESI): *m*/*z* [M – H]^–^ calcd for C_17_H_17_O_2_^–^ 253.1234; found: 253.1236.

*trans-3-benzyl-4-methoxychromane* (***14b***). yellow oil (2.3 mg, 18% yield); ^1^H NMR (400 MHz, CDCl_3_) *δ* 7.34–7.27 (overlapped, 2H), 7.26 (overlapped, 1H), 7.25–7.20 (overlapped, 2H), 7.17 (d, *J* = 7.6 Hz, 2H), 6.94 (d, *J* = 7.4 Hz, 1H), 6.90 (d, *J* = 8.5 Hz, 1H), 4.26 (dd, *J* = 10.9, 2.2 Hz, 1H), 4.01 (d, *J* = 10.8 Hz, 1H), 3.96 (s, 1H), 3.36 (s, 3H), 2.58 (d, *J* = 2.5 Hz, 1H), 2.56 (s, 1H), 2.38–2.28 (m, 1H); ^13^C NMR (100 MHz, CDCl_3_) *δ* 154.6, 139.5, 131.8, 129.2, 128.6, 129.2, 128.6, 126.4, 120.3, 119.9, 117.1, 75.9, 64.5, 55.8, 38.1, 34.7; HRMS (ESI): *m*/*z* [M – H]^–^ calcd for C_17_H_17_O_2_^–^ 253.1234; found: 253.1232.

*3-benzyl-2H-chromene* (***15***). colorless oil (2.4 mg, 21.6% yield); ^1^H NMR (400 MHz, CDCl_3_) *δ* 7.32 (t, *J* = 7.3 Hz, 2H), 7.24 (overlapped, 3H), 7.06 (td, *J* = 7.8, 1.6 Hz, 1H), 6.93 (dd, *J* = 7.4, 1.5 Hz, 1H), 6.84 (t, *J* = 7.4 Hz, 1H), 6.75 (d, *J* = 8.0 Hz, 1H), 6.16 (s, 1H), 4.65 (s, 2H), 3.43 (s, 2H); ^13^C NMR (100 MHz, CDCl_3_) *δ* 153.0, 137.6, 134.1, 129.1, 129.1,128.7, 128.7, 128.6, 126.8, 126.3, 121.4, 120.5, 115.5, 68.2, 40.0; HRMS (ESI): *m*/*z* [M + Br]^–^ calcd for C_16_H_14_OBr^–^ 301.0234; found: 301.0235.

### Procedure for preparation of *trans*-12h, *cis*-12h, *trans*-12c and *cis*-12c

*Trans***-12h,** *cis***-12h, ***trans***-12c** and *cis***-12c** were prepared using the same protocol as the preparation of **12a** in *trans*-**6h**, and *cis*-**6h**. The residue was purified by silica gel chromatography (petroleum ether/ethyl acetate, 5:1–2:1, v/v) to give the product.

*trans-3-benzyl-5,6,7-trimethoxychroman-4-ol* (*trans-****12h***). colorless oil (6 mg, 36% yield); ^1^H NMR (400 MHz, CDCl_3_) *δ* 7.36–7.20 (overlapped, 5H), 6.17 (s, 1H), 4.70 (s, 1H), 3.99 (d, *J* = 7.8 Hz, 2H), 3.96 (s, 3H), 3.80 (s, 3H), 3.78 (s, 3H), 2.97 (dd, *J* = 13.8, 7.6 Hz, 1H), 2.68 (dd, *J* = 13.8, 7.9 Hz, 1H), 2.28–2.18 (m, 1H), 2.15 (s, 1H); ^13^C NMR (100 MHz, CDCl_3_) *δ* 154.6, 152.0, 150.9, 139.6, 135.4, 129.2, 129.2, 128.7, 128.7, 126.4, 110.6, 95.7, 65.1, 61.4, 61.1, 60.6, 56.0, 40.2, 33.1; HRMS (ESI): *m*/*z* [M + NH_4_]^+^ calcd for C_19_H_26_O_5_N^+^ 348.1805; found: 348.1806.

*cis-3-benzyl-5,6,7-trimethoxychroman-4-ole* (*cis-****12h***). colorless oil (13.1 mg, 40% yield); ^1^H NMR (400 MHz, CDCl_3_) *δ* 7.31 (t, *J* = 7.4 Hz, 2H), 7.24 (d, *J* = 7.2 Hz, 1H), 7.20 (d, *J* = 7.7 Hz, 2H), 6.25 (s, 1H), 4.61 (s, 1H), 4.11 (dd, *J* = 11.0, 2.0 Hz, 1H), 3.99 (s, 3H), 3.93 (dd, *J* = 10.9, 2.4 Hz, 1H), 3.84 (s, 3H), 3.81 (s, 3H), 2.65 (dd, *J* = 13.7, 6.9 Hz, 1H), 2.54 (overlapped, 2H), 2.22 (ddd, *J* = 9.2, 6.6, 2.9 Hz, 1H); ^13^C NMR (100 MHz, CDCl_3_) *δ* 154.4, 152.5, 150.6, 139.4, 135.6, 129.2, 129.2. 128.5, 128.5, 126.3, 109.1, 95.9, 64.4, 63.1, 61.3, 61.0, 55.89, 40.5, 34.6; HRMS (ESI): *m*/*z* [M + NH_4_]^+^ calcd for C_19_H_26_O_5_N^+^ 348.1805; found: 348.1803.

*trans-2-benzyl-1-(2,3,4-trimethoxyphenyl)propane-1,3-diol (trans-****12c****).* yellow oil (6.8 mg, 41% yield); ^1^H NMR (400 MHz, CDCl_3_)* δ* 7.26 (overlapped, 2H), 7.20–7.11 (m, 4H), 6.70 (d, *J* = 8.6 Hz, 1H), 4.96 (t, *J* = 4.8 Hz, 1H), 3.86 (s, 3H), 3.85 (s, 3H), 3.83 (s, 3H), 3.78 (d, *J* = 11.1 Hz, 1H), 3.57 (d, *J* = 11.1 Hz, 1H), 3.04 (d, *J* = 4.9 Hz, 1H), 2.78 (s, 1H), 2.74 (dd, *J* = 13.9, 5.9 Hz, 1H), 2.60 (dd, *J* = 13.8, 9.3 Hz, 1H), 2.20 (ddd, *J* = 8.7, 5.8, 2.8 Hz, 1H); ^13^C NMR (100 MHz, CDCl_3_) *δ* 153.3, 151.0, 142.0, 140.4, 129.2, 129.2, 128.8, 128.5, 128.5, 126.1, 121.8, 107.3, 73.6, 63.7, 61.1, 60.9, 56.1, 47.4, 34.9; HRMS (ESI): *m*/*z* [M + Na]^+^ calcd for C_19_H_24_O_5_Na^+^ 355.1516; found: 355.1515.

*cis-2-benzyl-1-(2,3,4-trimethoxyphenyl)propane-1,3-diol (cis-****12c****).* yellow oil (10.9 mg, 33% yield); ^1^H NMR (400 MHz, CDCl_3_) *δ* 7.23 (d, *J* = 7.4 Hz, 2H), 7.15 (t, *J* = 9.0 Hz, 4H), 6.71 (d, *J* = 8.7 Hz, 1H), 5.19 (d, *J* = 5.2 Hz, 1H), 3.93 (s, 3H), 3.87 (s, 6H), 3.56 (dd, *J* = 11.2, 2.6 Hz, 1H), 3.51 (dd, *J* = 11.2, 4.1 Hz, 1H), 2.92 (s, 1H), 2.88 (dd, *J* = 13.7, 3.6 Hz, 1H), 2.74 (dd, *J* = 13.5, 11.1 Hz, 1H), 2.31 (s, 1H), 2.10–2.01 (m, 1H); ^13^C NMR (100 MHz, CDCl_3_) *δ* 153.1, 150.6, 141.9, 141.1, 129.3, 129.3, 128.5, 128.4, 128.4, 125.9, 121.8, 107.4, 72.3, 63.1, 61.3, 60.9, 56.1, 48.7, 31.4; HRMS (ESI): *m*/*z* [M + Na]^+^ calcd for C_19_H_24_O_5_Na^+^ 355.1516; found: 355.1512.

### Procedure for preparation of 16

To a solution of *trans***-12h** or *cis***-12h** (1.0 equiv) in dry benzene (0.05 M), then pTSA (5 mol%) was added at 0 ℃. The reaction was sealed and stirred at room temperature for 2 h. The reaction was quenched with NaHCO_3_ saturated aqueous solution, and was extracted with ethyl acetate. The combined organic layer was dried over Na_2_SO_4_, filtered, and concentrated to give the crude product that was further purified by flash column chromatography on silica gel (petroleum ether/dichloromethane/ethyl acetate/ethyl acetate, 80:10:2, v/v) to afford **16**.

*3-benzyl-5,6,7-trimethoxy-2H-chromene* (***16***). colorless oil (1.1 mg, quantitative yield); ^1^H NMR (400 MHz, CDCl_3_)* δ* 7.30 (d, *J* = 6.9 Hz, 2H), 7.24 (d, *J* = 6.3 Hz, 3H), 6.45 (s, 1H), 6.19 (s, 1H), 4.53 (s, 2H), 3.88 (s, 3H), 3.80 (s, 6H), 3.46 (s, 2H); ^13^C NMR (100 MHz, CDCl_3_) *δ* 153.2, 149.1, 149.4, 137.9, 130.5, 128.8, 128.8, 128.6, 128.6, 126.6, 115.3, 109.5, 95.9, 67.8, 61.5, 61.1, 56.0, 40.3, 32.0; HRMS (ESI): *m*/*z* [M + H]^+^ calcd for C_19_H_21_O_4_^+^ 313.1434; found: 313.1435.

### Procedure for preparation of 11h

The contents of a reaction tube charged with a solution of **7h** (1.0 equiv), H_3_PO_4_ (50 mol%) in freshly distilled dichloromethane (0.05 M) were stirred for 12 h at 80 ℃. The reaction was quenched with NaHCO_3_ saturated aqueous solution, and was extracted with dichloromethane. The combined organic layer was dried over Na_2_SO_4_, filtered, and concentrated to give the crude product that was further purified by flash column chromatography on silica gel (petroleum ether/dichloromethane/ethyl acetate, 10:10:2, v/v) to afford **11h**.

*(trans-1-(6-bromo-2,3,4-trimethoxyphenyl)-2,3-dihydro-1H-inden-2-yl)methanol (****11h****)*. colorless oil (24.2 mg, 13% yield, 75% brsm); *trans-*isomer: ^1^H NMR (400 MHz, CDCl_3_) *δ* 7.23 (t, *J* = 6.8 Hz, 1H), 7.13 (t, *J* = 7.4 Hz, 1H), 7.07 (t, *J* = 7.4 Hz, 1H), 6.91 (s, 1H), 6.88 (d, *J* = 4.0 Hz, 1H), 4.73 (d, *J* = 7.5 Hz, 1H), 3.86 (s, 3H), 3.83–3.78 (overlapped, 1H), 3.75 (overlapped, 4H), 3.32 (dd, *J* = 15.7, 8.8 Hz, 1H), 3.08 (s, 3H), 2.99 (dd, *J* = 15.0, 7.2 Hz, 1H), 2.88 (dd, *J* = 16.0, 7.5 Hz, 1H), 1.61 (s, 1H); ^13^C NMR (100 MHz, CDCl_3_) *δ* 153.6, 153.0, 146.4, 142.8, 142.6, 130.5, 126.5, 126.2, 124.5, 123.5, 118.8, 111.0, 66.4, 60.6, 60.1, 56.2, 52.4, 49.0, 35.9; HRMS (ESI): *m*/*z* [M + H]^+^ calcd for C_19_H_20_O_4_Br^+^ 393.0696; found: 393.0693.

### Procedure for preparation of 17

A Schlenk tube was equipped with a magnetic stirring bar, and loaded with Cs_2_CO_3_ (200 mol%), CuI (20 mol %), *rac*-cyclohexane-1,2-diamine (22 mol %), **11h** (1.0 equiv) and 1,4-dioxane (0.1 M) under air. The tube was sealed, evacuated, and refilled with argon. The reaction mixture was stirred at 120 °C for 12 h. Afterward, the mixture was filtered and the solid was washed with ethyl acetate, and the filtrates were concentrated under reduced pressure. The residue was purified by silica gel chromatography (petroleum ether/dichloromethane/ethyl acetate/ethyl acetate, 80:10:2, v/v) to give the product **17**.

*trans-1,2,3-trimethoxy-6,6a,7,11b-tetrahydroindeno[2,1-c]chromene (****17****)*. yellow oil (3.9 mg, 10% yield, 73% brsm); ^1^H NMR (400 MHz, CDCl_3_) *δ* 8.19 (d, *J* = 7.3 Hz, 1H), 7.28 (d, *J* = 7.1 Hz, 1H), 7.24–7.15 (overlapped, 2H), 6.27 (s, 1H), 4.46 (dd, *J* = 9.9, 3.4 Hz, 1H), 4.24 (t, *J* = 10.5 Hz, 1H), 3.97 (d, *J* = 11.8 Hz, 1H), 3.89 (s, 3H), 3.84 (s, 3H), 3.82 (s, 3H), 2.92 (dd, *J* = 13.6, 5.5 Hz, 1H), 2.63 (t, *J* = 12.8 Hz, 1H), 2.54 (td, *J* = 12.0, 5.8 Hz, 1H); ^13^C NMR (100 MHz, CDCl_3_) *δ* 152.7, 152.5, 151.3, 144.3, 143.9, 136.3, 126.6, 126.5, 126.3, 124.6, 111.4, 97.1, 70.2, 61.5, 60.6, 56.0, 47.5, 46.8, 33.1; HRMS (ESI): *m*/*z* [M + H]^+^ calcd for C_19_H_21_O_4_^+^ 313.1434; found: 313.1432.

### Supplementary Information

**Additional file 1: Scheme S1.** Copies of NMR spectra for all synthetic compounds, and X-ray crystallography data of compounds **11a-1** and **12b**.

## Data Availability

All data generated or analyzed during this study are available in this published article and its Additional files.
