# Supplementary material for: Hydroxymethylation hydroxylation of 1,3-diarylpropene through a catalytic diastereoselective Prins reaction: cyclization logic and access to brazilin core
Source: Nat Prod Bioprospect. 2024 May 14;14(1):29. doi: 10.1007/s13659-024-00450-2 (PMC11091033; doi:10.1007/s13659-024-00450-2)
Supplement: Supplementary file 1 — Additional file 1: Scheme S1. Copies of NMR spectra for all synthetic compounds, and X-ray crystallography data of compounds 11a-1 and 12b. [file 13659_2024_450_MOESM1_ESM.pdf]

Supporting information for

Hydroxymethylation Hydroxylation of 1,3-Diarylpropene Through a Catalytic Diastereoselective Prins Reaction: Cyclization Logic and Access to Brazilin Core

Xin-Ting Hu,[†,§] Qing-Yan Cheng,[†,§] Yan-Ping Chen,[†] Kun Li,[†] Cai-Xian Yan,[¶] Dashan Li,[†] and Li-Dong Shao,*[,†]

[†] *Yunnan Key Laboratory of Southern Medicinal Utilization, School of Chinese Materia Medica, Yunnan University of Chinese Medicine, Kunming, 650500, China.*

[¶] *Yunnan Precious Metals Laboratory, Kunming Institute of Precious Metals, Kunming, 650106, China.*

[§] These authors contributed equally.

* Corresponding author. Email: L.-D Shao (shaolidong@ynucm.edu.cn).

Table of Contents

Scheme S1. Structures of 8a-8x. S1

NMR spectra copies for all synthetic compounds......S2

X-ray crystallography data of compounds 11a-1 and 12b. S68

Scheme S1. Structures of 8a-8x.

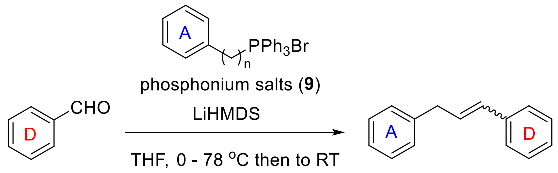

10 27 - 80% **8**

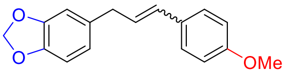

8a
Z : *E* ~ 1 : 5
87%

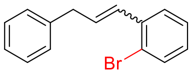

8b
Z : *E* ~ 3 : 1
75%

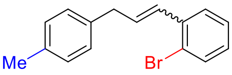

8c
Z : *E* ~ 5 : 4
78%

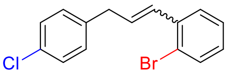

8d
Z : *E* ~ 5 : 2
79%

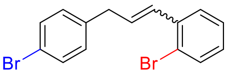

8e
Z : *E* ~ 5 : 2
72%

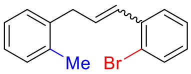

8f
Z : *E* ~ 2 : 1
64%

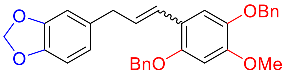

8g
Z : *E* ~ 3 : 5
65%

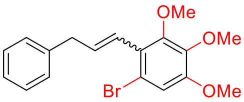

8h
Z : *E* ~ 3 : 2
70%

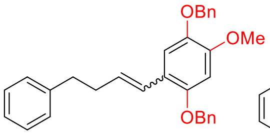

8i
Z : *E* ~ 2 : 3
65%

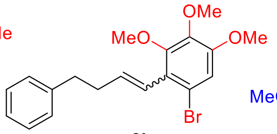

8j
Z : *E* ~ 1 : 1
55%

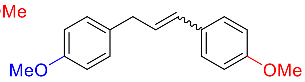

8k
Z : *E* ~ 3 : 5
69%

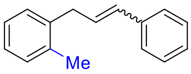

8l
Z : *E* ~ 2 : 1
60%

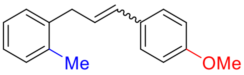

8m
Z : *E* ~ 2 : 3
70%

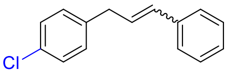

8n
Z : *E* ~ 2 : 1
77%

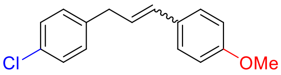

8o
Z : *E* ~ 4: 5
28%

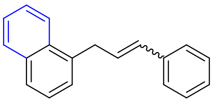

8p
Z : *E* ~ 2 : 1
53%

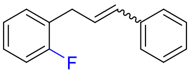

8q
Z : *E* ~ 3 : 2
59%

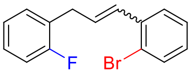

8r
Z : *E* ~ 5 : 1
32%

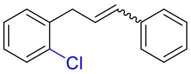

8s
Z : *E* ~ 1 : 1
65%

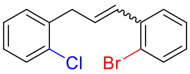

8t
Z : *E* ~ 5 : 1
54%

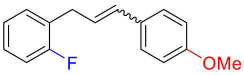

8u
Z : *E* ~ 4 : 5
67%

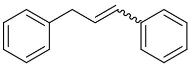

8v
Z : *E* ~ 5 : 3
80%

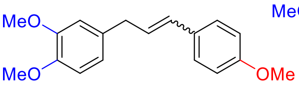

8w
Z : *E* ~ 1 : 2
72%

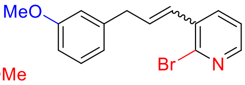

8x
Z : *E* ~ 5 : 2
66%
unsuccessful case

NMR spectra copies for all synthetic compounds.

NMR spectra of 8a-8x

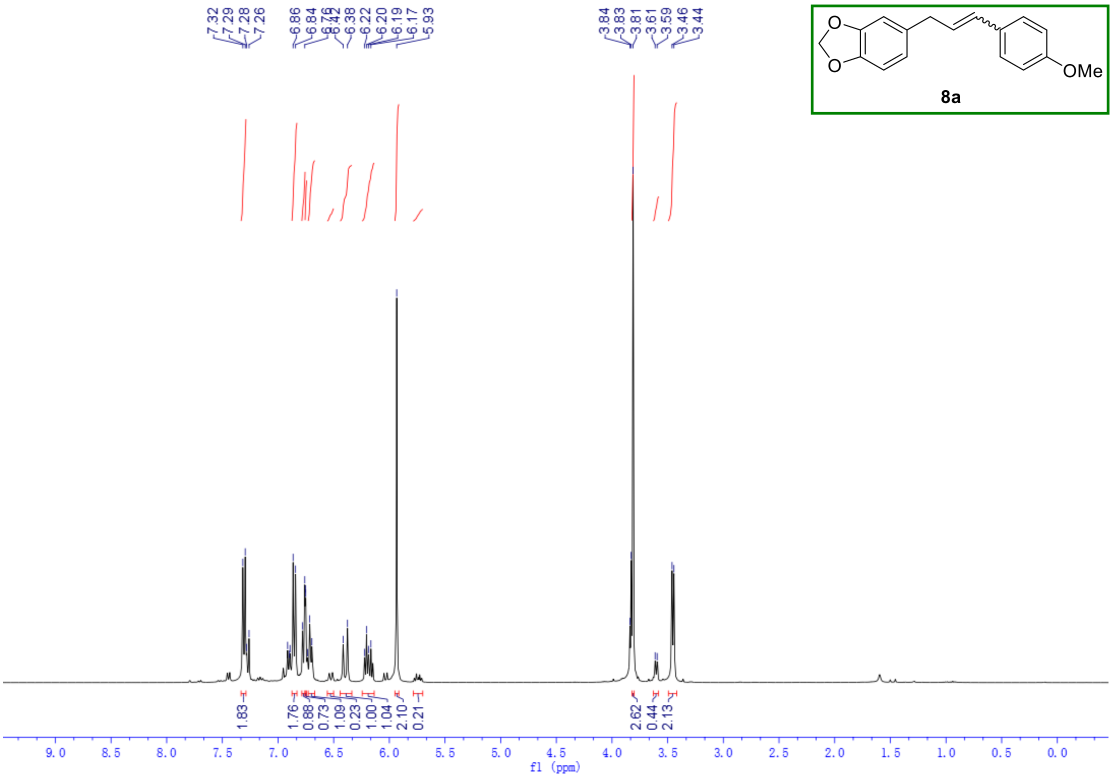

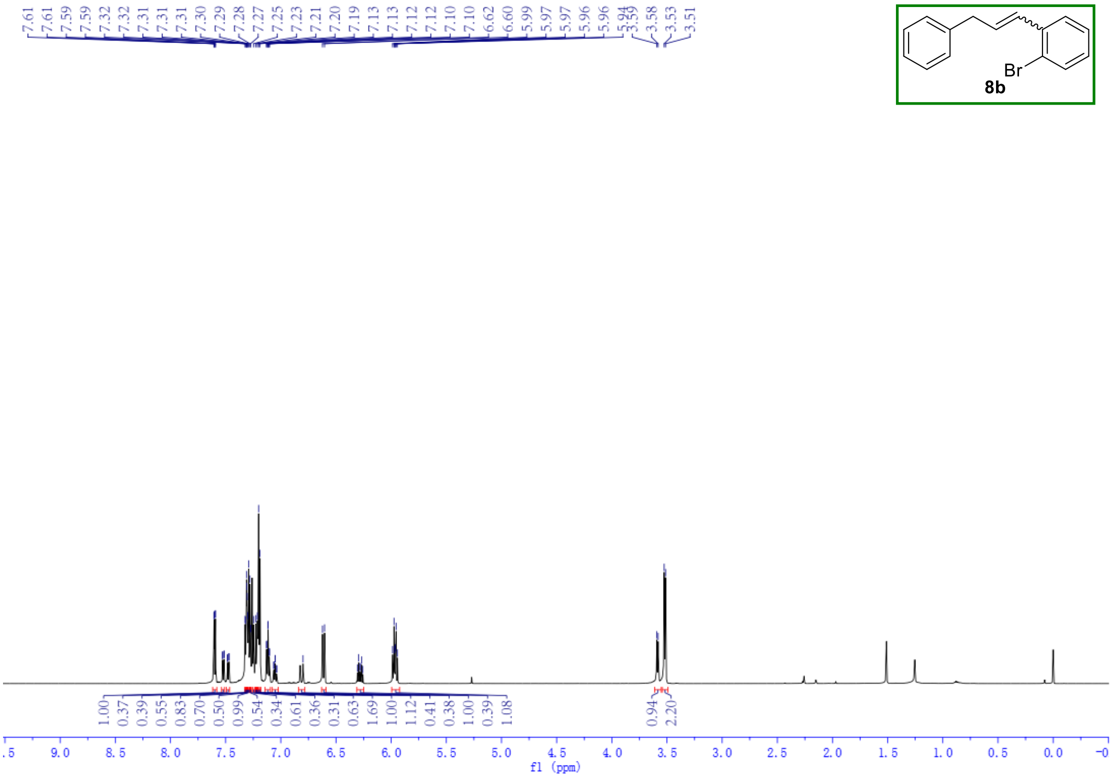

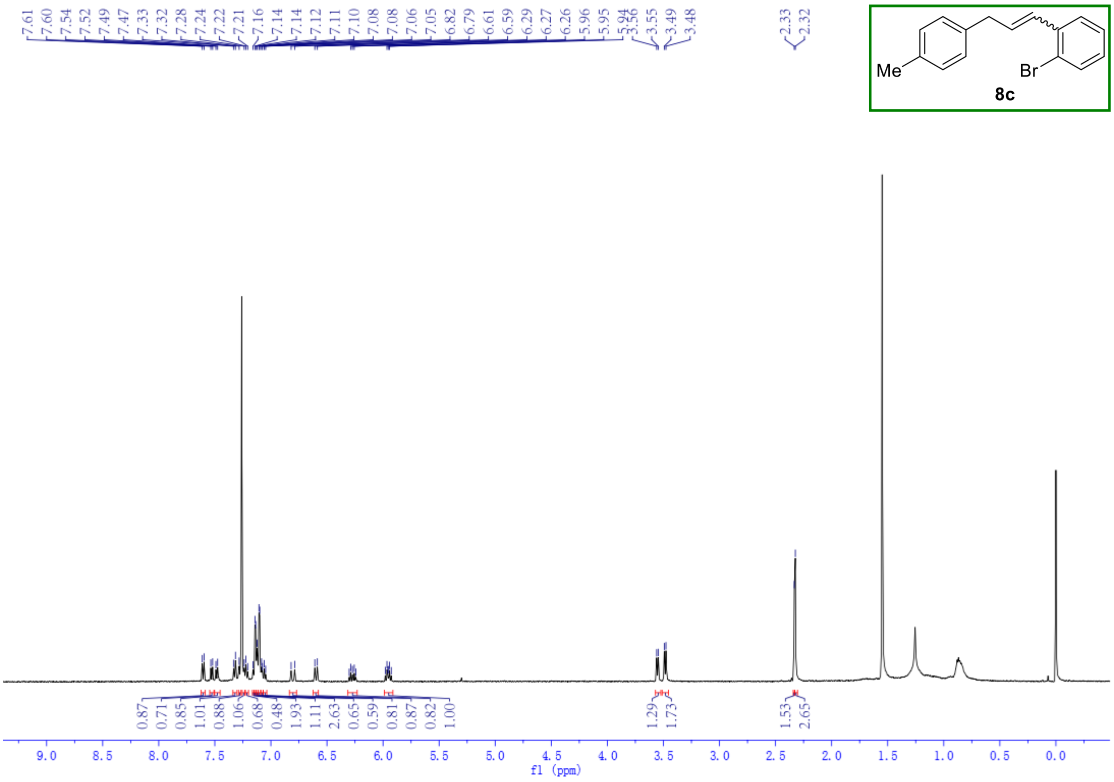
Me
Br
8c
f1 (ppm)

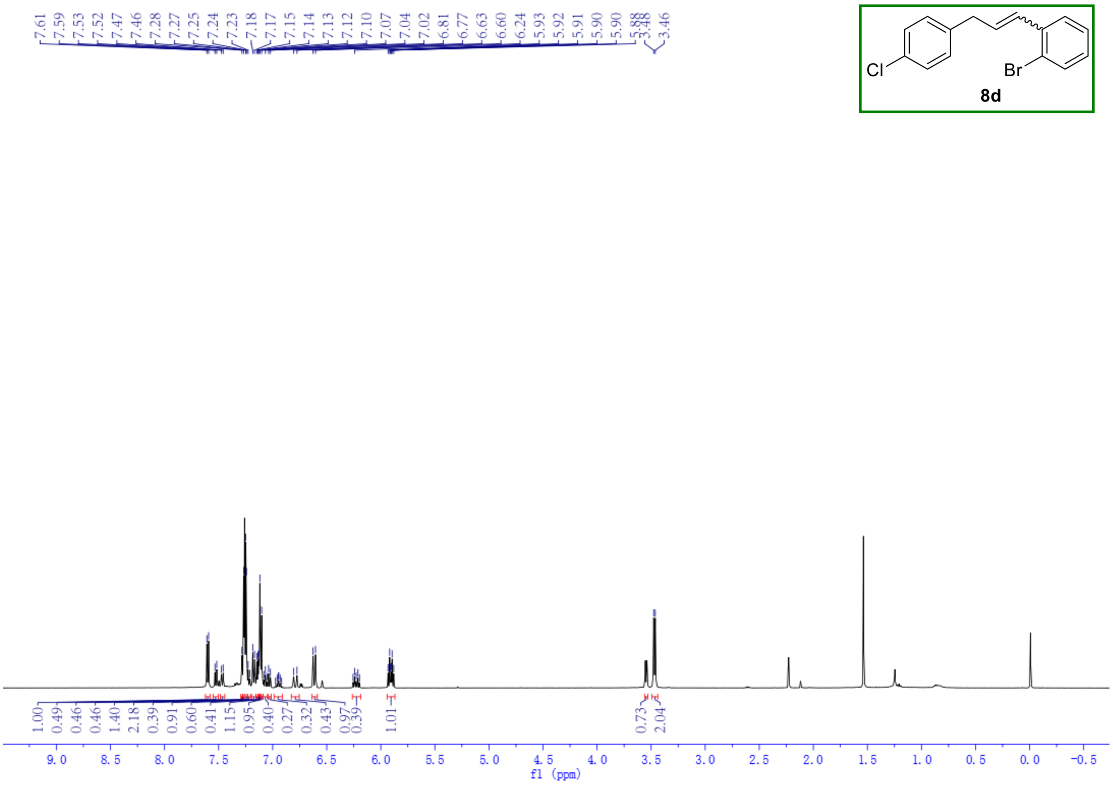
Cl
Br
8d
f1 (ppm)

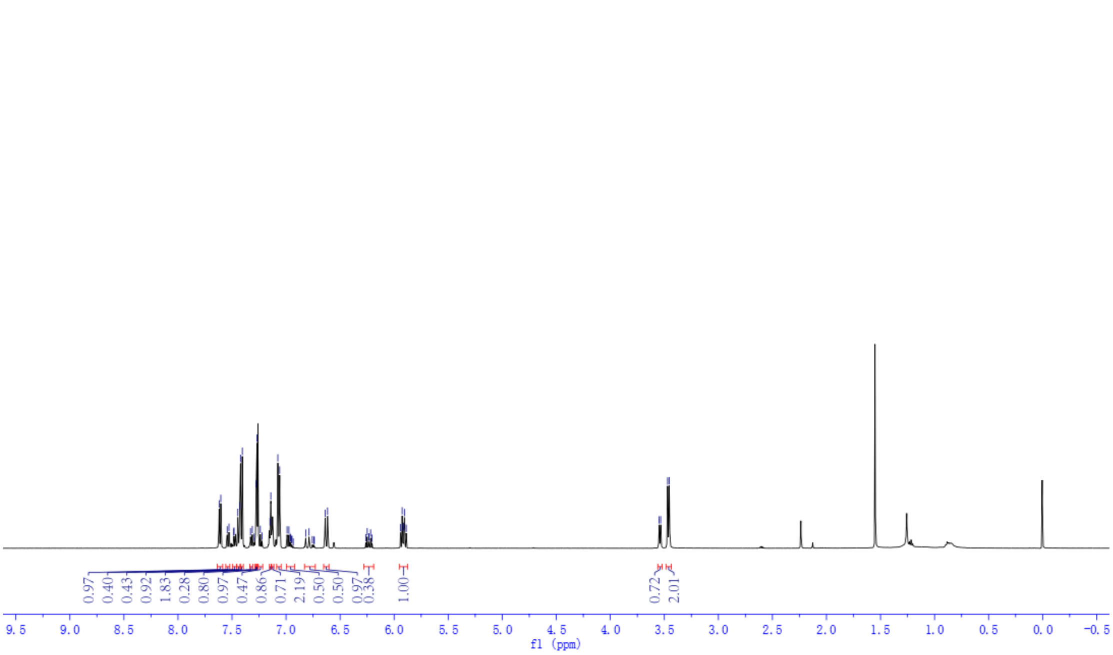

7.62 7.60 7.54 7.53 7.48 7.48 7.45 7.43 7.42 7.40 7.33 7.31 7.28 7.27 7.24 7.15 7.14 7.08 7.06 6.99 6.97 6.79 6.64 6.62 6.25 6.22 5.94 5.93 5.92 5.91 5.90 5.89 3.55 3.53 3.47 3.45
0.97 0.40 0.43 0.92 1.83 0.28 0.80 0.97 0.47 0.86 0.71 2.19 0.50 0.50 0.97 0.38 1.00 0.72 2.01
9.5 9.0 8.5 8.0 7.5 7.0 6.5 6.0 5.5 5.0 4.5 4.0 3.5 3.0 2.5 2.0 1.5 1.0 0.5 0.0 -0.5
f1 (ppm)

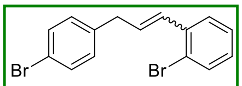

Br
Br

8e

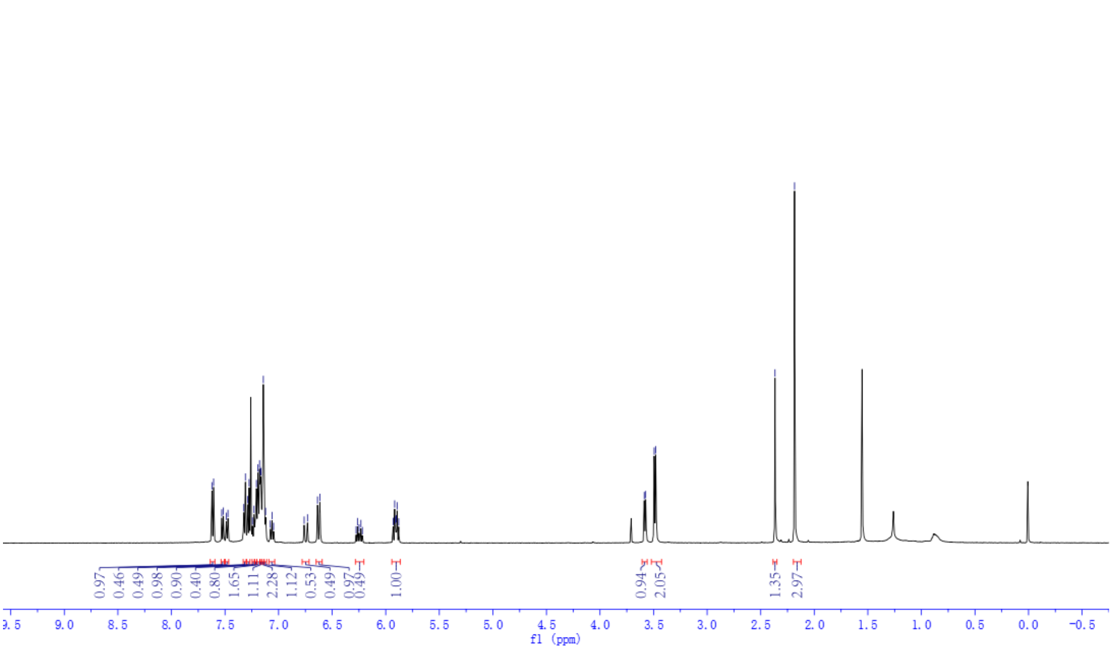

7.62 7.61 7.53 7.51 7.49 7.47 7.32 7.31 7.29 7.27 7.24 7.23 7.21 7.19 7.18 7.17 7.17 7.14 7.12 7.06 6.76 6.73 6.64 6.61 6.26 5.93 5.92 5.91 5.90 5.89 5.88 3.59 3.57 3.49 3.48 2.37 2.18
0.97 0.46 0.49 0.98 0.90 0.40 0.80 1.65 1.11 2.28 1.12 0.53 0.49 0.97 0.49 1.00 0.94 2.05 1.35 2.97
9.5 9.0 8.5 8.0 7.5 7.0 6.5 6.0 5.5 5.0 4.5 4.0 3.5 3.0 2.5 2.0 1.5 1.0 0.5 0.0 -0.5
f1 (ppm)

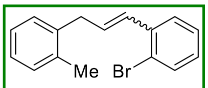

Me
Br

8f

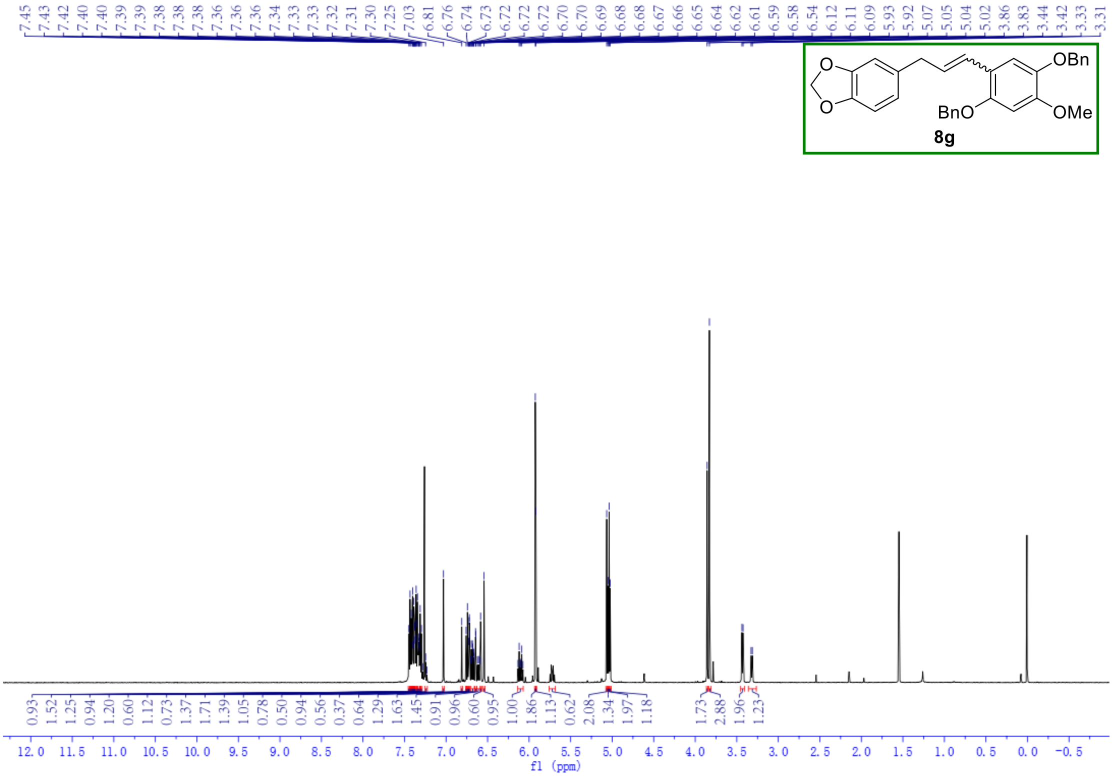
7.45 7.43 7.42 7.40 7.40 7.39 7.39 7.38 7.38 7.37 7.36 7.36 7.36 7.34 7.33 7.33 7.32 7.31 7.30 7.25 7.03 6.81 6.76 6.74 6.73 6.72 6.72 6.72 6.70 6.70 6.69 6.68 6.68 6.67 6.66 6.65 6.64 6.62 6.61 6.59 6.58 6.54 6.12 6.11 6.09 5.93 5.92 5.07 5.05 5.04 5.02 3.86 3.83 3.44 3.42 3.33 3.31
OBn
BnO
OMe
8g
0.93 1.52 1.25 0.94 1.20 0.60 1.12 0.73 1.37 1.71 1.39 1.05 0.78 0.50 0.94 0.56 0.37 0.64 1.29 1.63 1.45 0.91 0.96 0.60 0.95 1.00 1.86 1.13 0.62 2.08 1.34 1.97 1.18 1.73 2.88 1.96 1.23
12.0 11.5 11.0 10.5 10.0 9.5 9.0 8.5 8.0 7.5 7.0 6.5 6.0 5.5 5.0 4.5 4.0 3.5 3.0 2.5 2.0 1.5 1.0 0.5 0.0 -0.5
f1 (ppm)

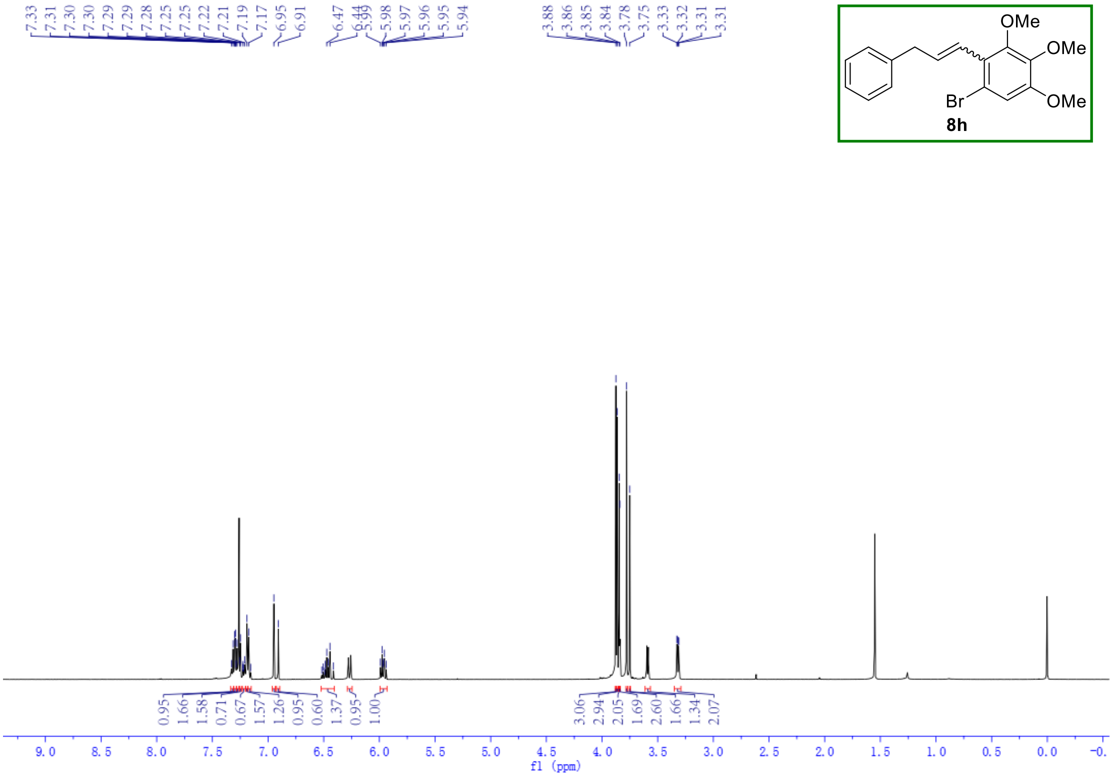
7.33 7.31 7.30 7.30 7.29 7.29 7.28 7.25 7.25 7.22 7.21 7.19 7.17 6.95 6.91 6.47 6.44 5.99 5.98 5.97 5.96 5.95 5.94 3.88 3.86 3.85 3.84 3.78 3.75 3.33 3.32 3.31 3.31
OMe
OMe
Br
OMe
8h
0.95 1.66 1.58 0.71 0.67 1.57 1.26 0.95 0.60 1.37 0.95 1.00 3.06 2.94 2.05 1.69 2.60 1.66 1.34 2.07
9.0 8.5 8.0 7.5 7.0 6.5 6.0 5.5 5.0 4.5 4.0 3.5 3.0 2.5 2.0 1.5 1.0 0.5 0.0 -0.
f1 (ppm)

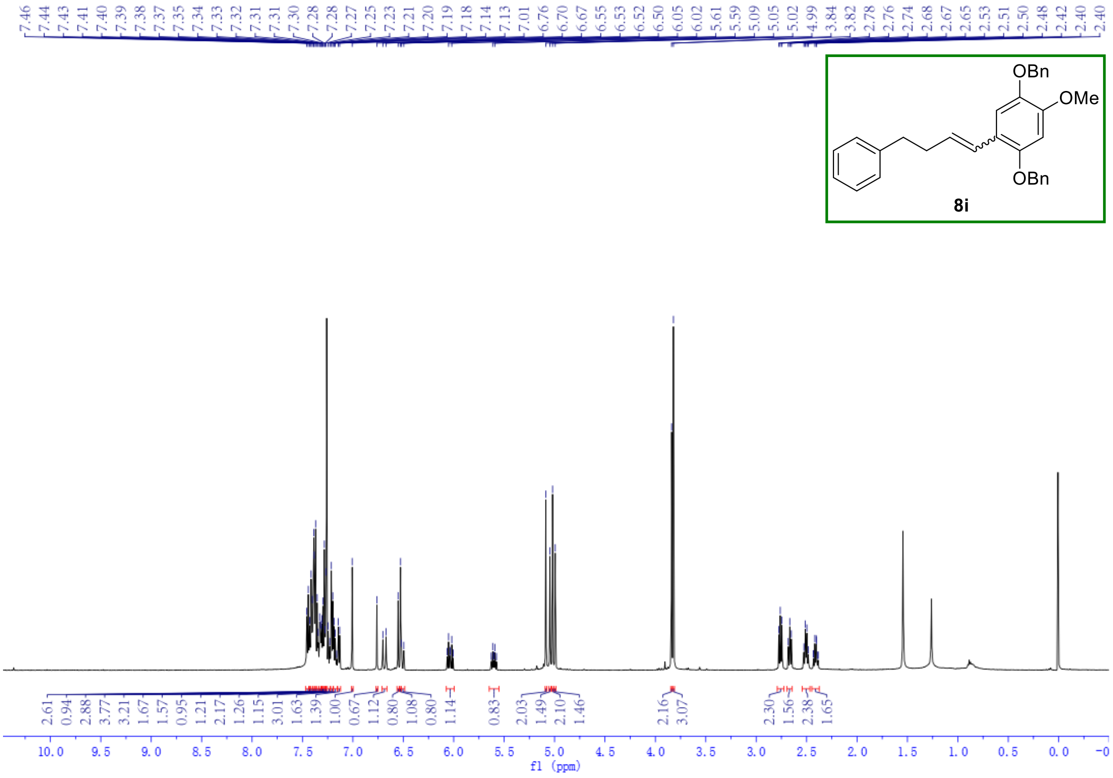
OBn
OMe
OBn
8i
f1 (ppm)

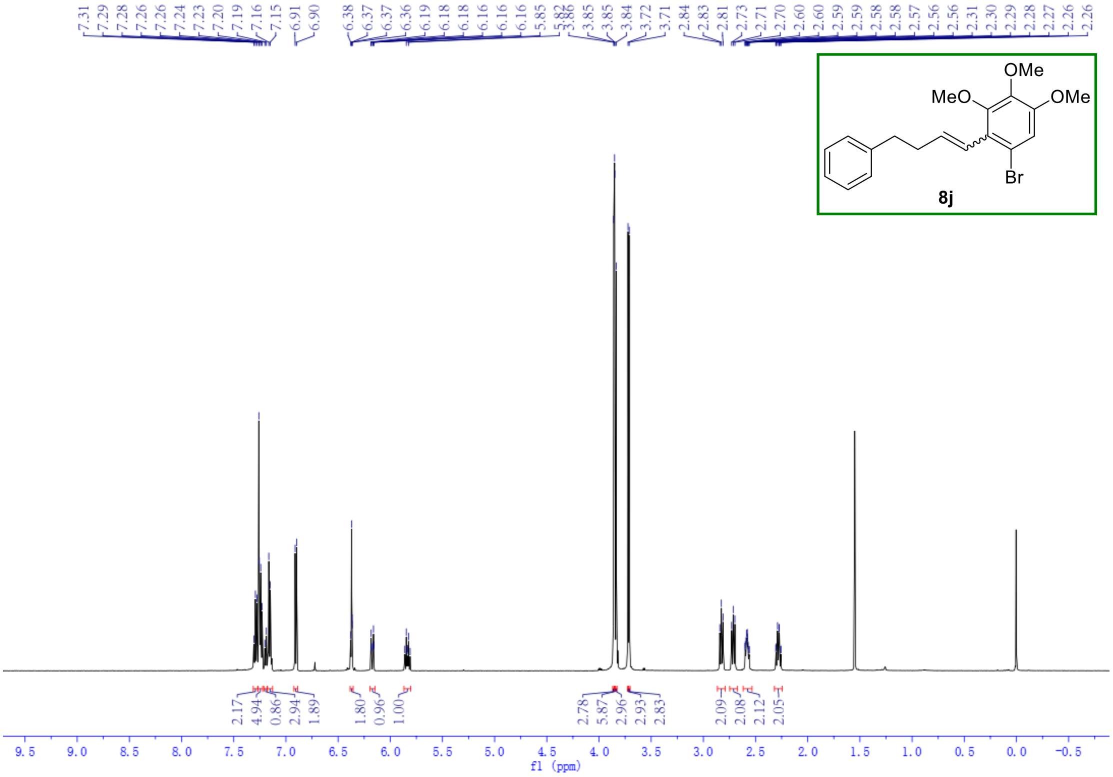
OMe
MeO
OMe
Br
8j
f1 (ppm)

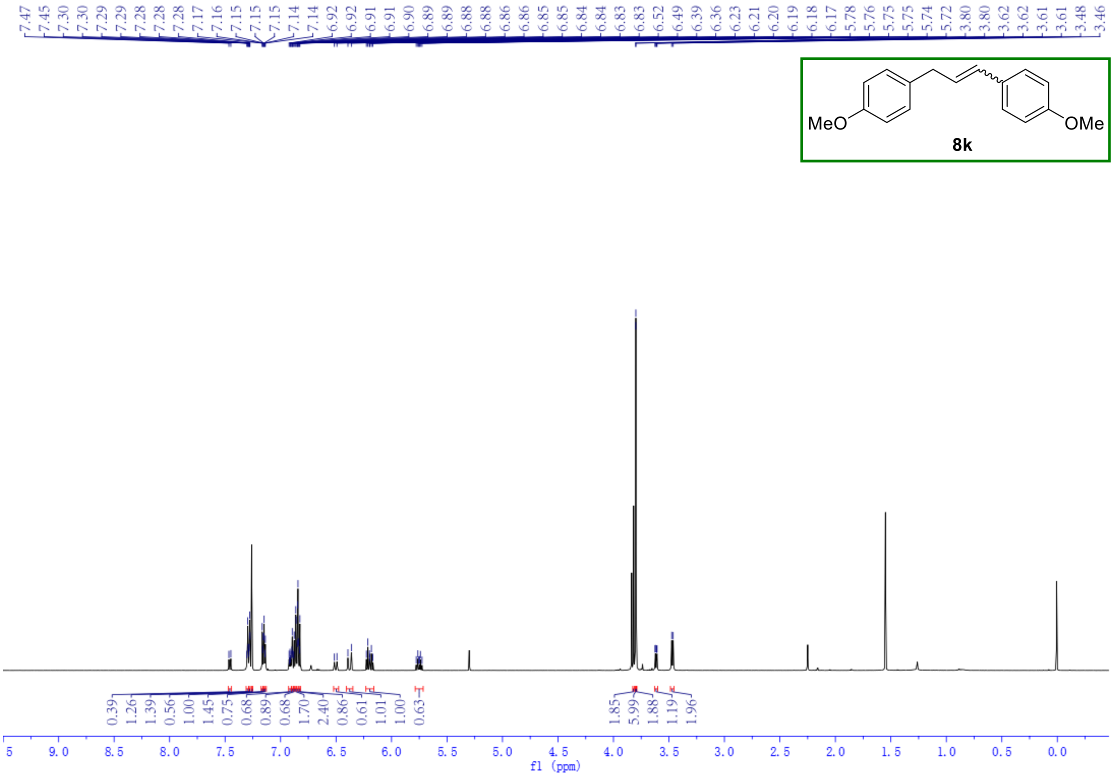
MeO
OMe
8k
f1 (ppm)

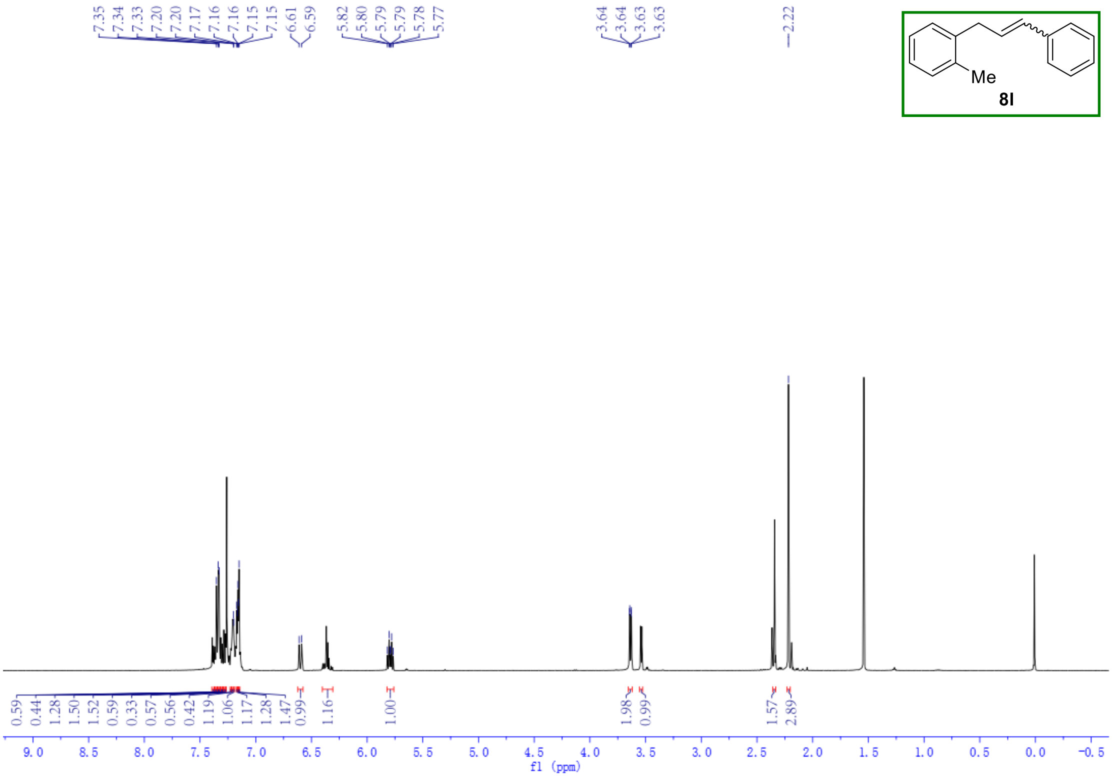
Me
8l
f1 (ppm)

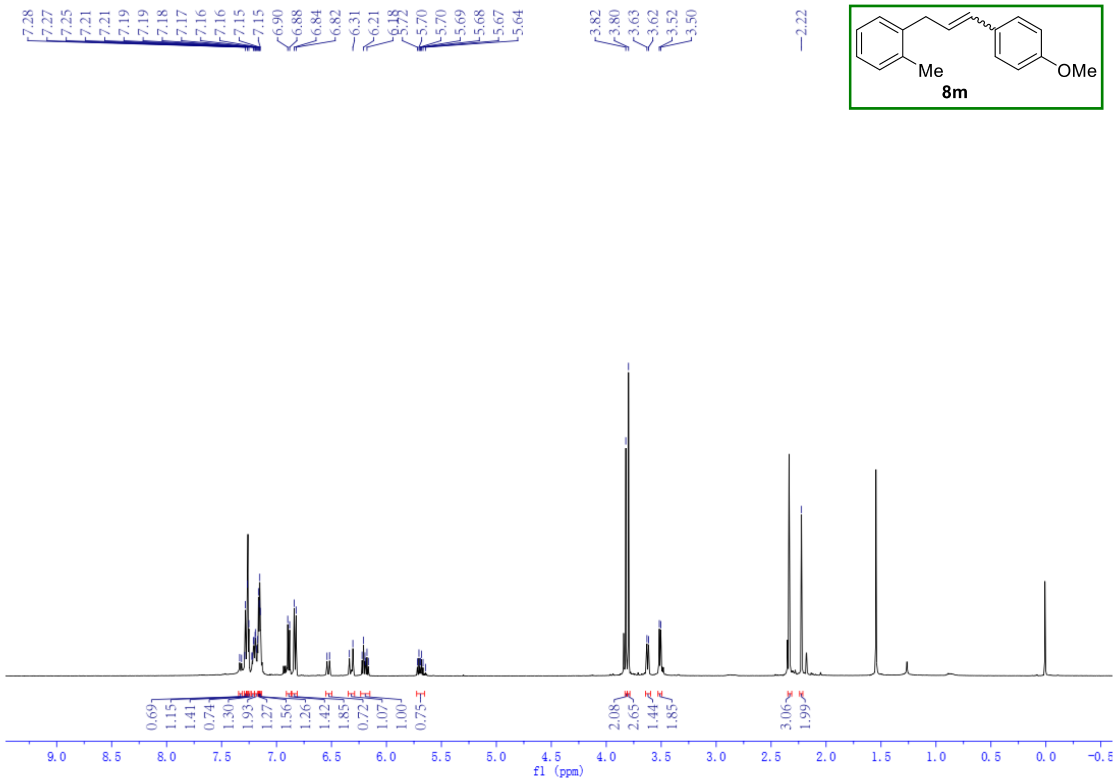
Me
OMe
8m
f1 (ppm)

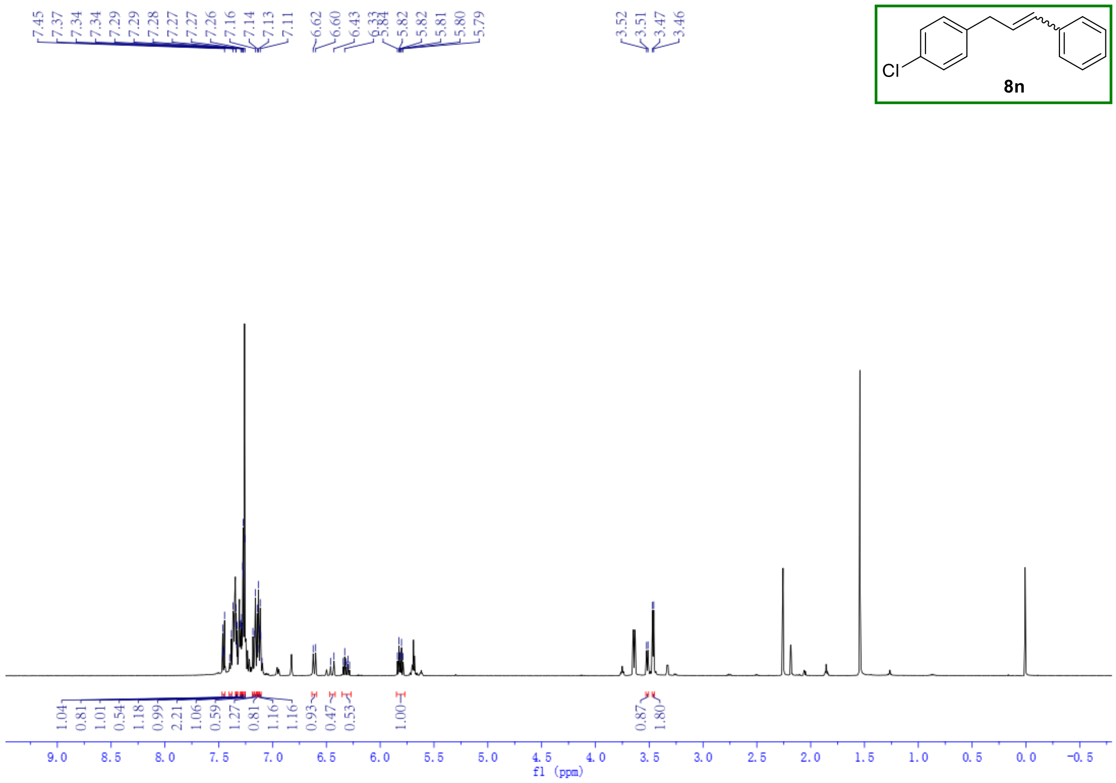
Cl
8n
f1 (ppm)

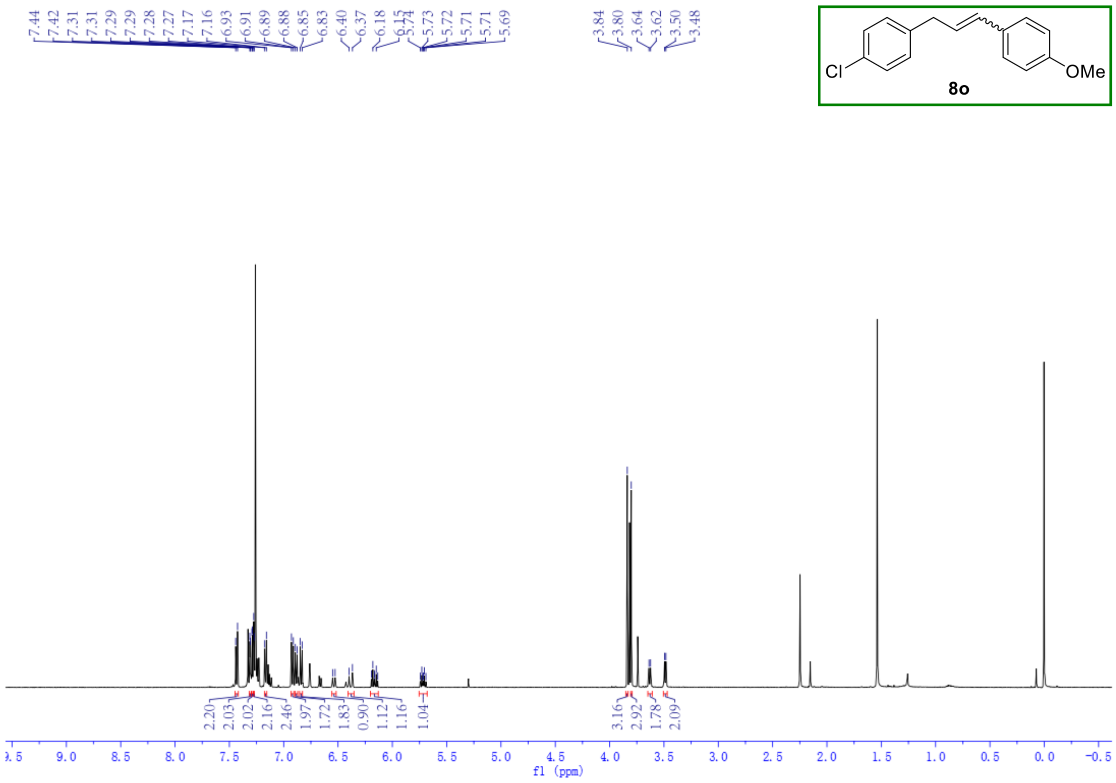
Cl
OMe
8o
f1 (ppm)

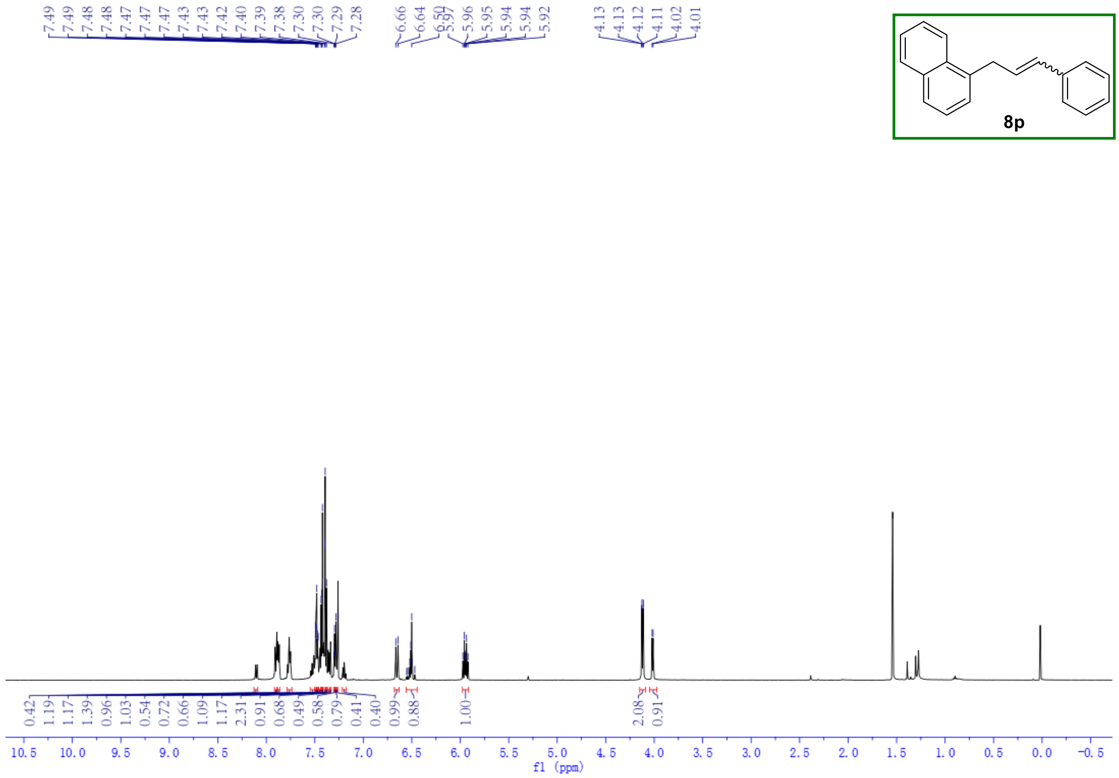
8p
f1 (ppm)

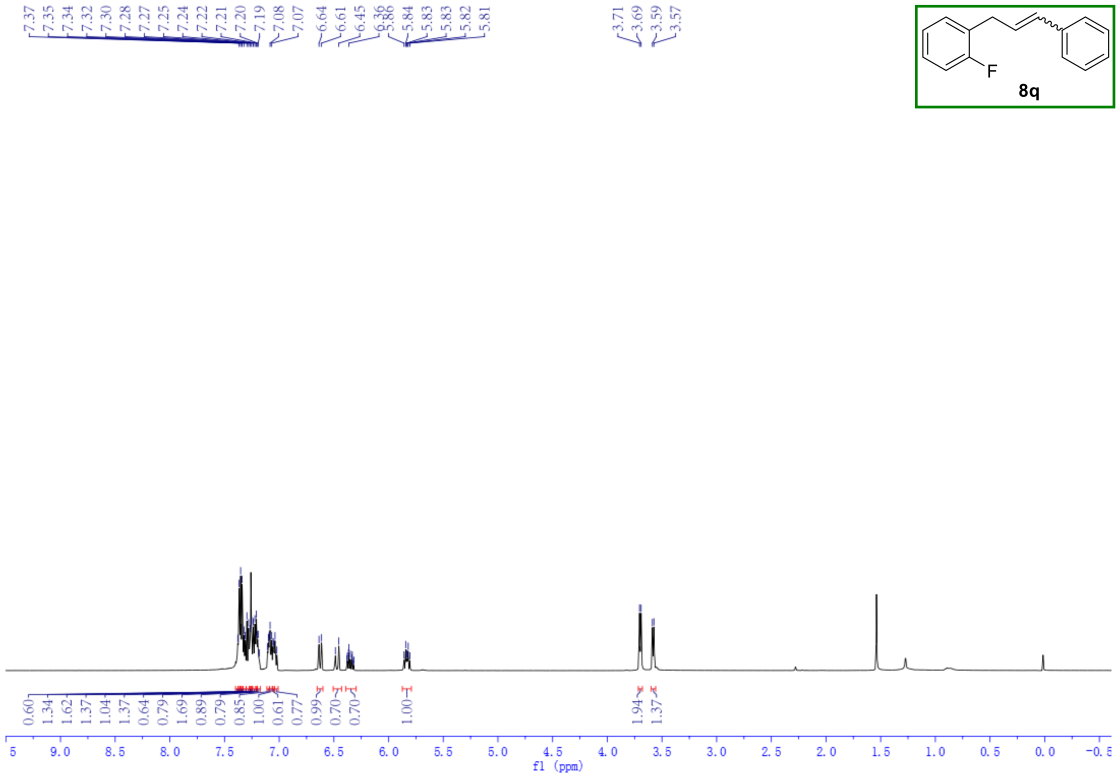
8q
f1 (ppm)

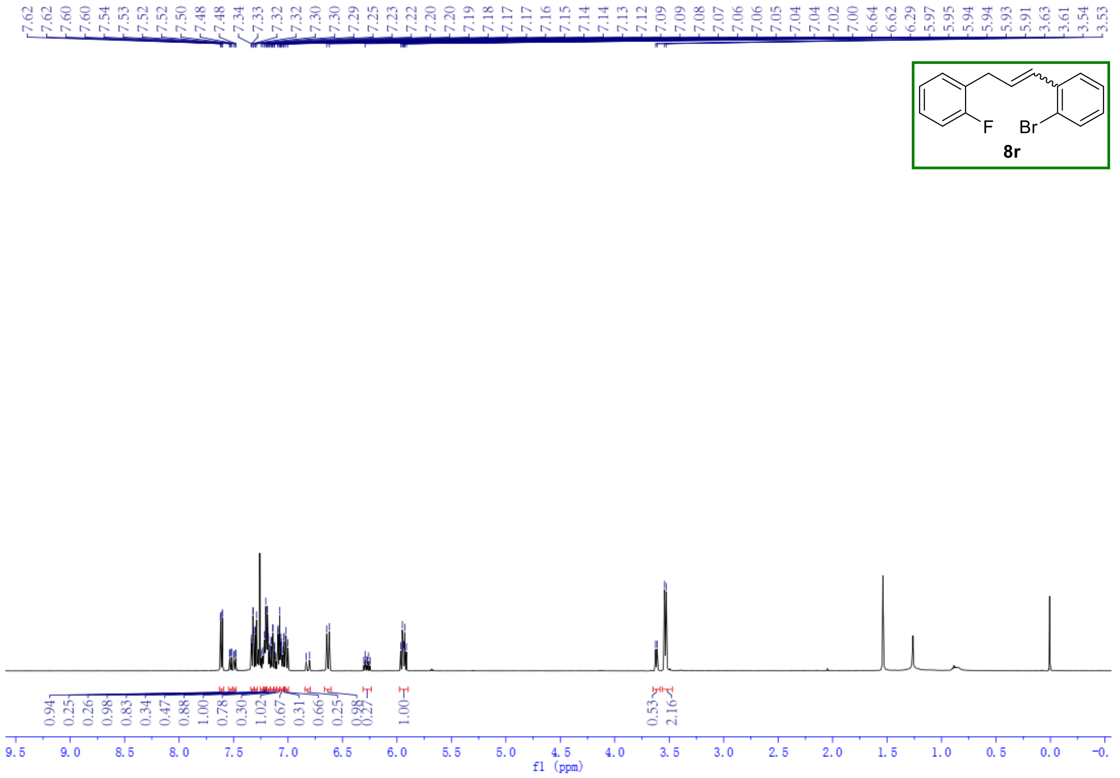
8r
f1 (ppm)

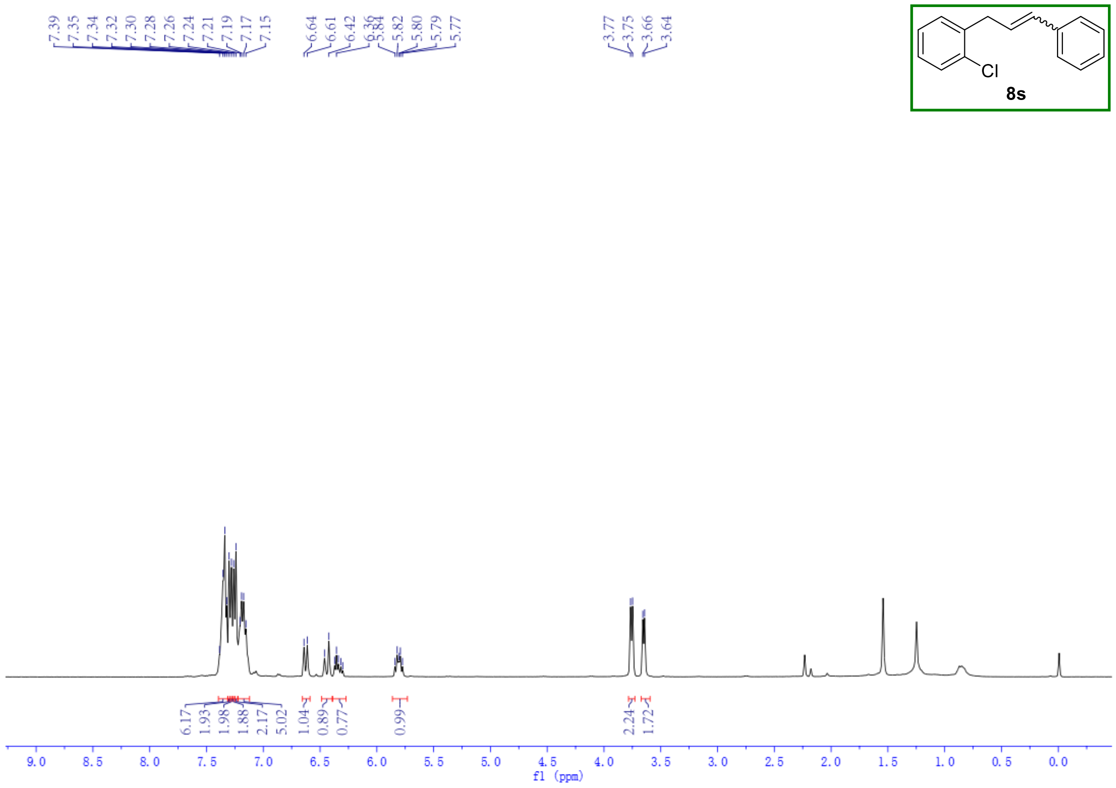
7.39 7.35 7.34 7.32 7.30 7.28 7.26 7.24 7.21 7.19 7.17 7.15
6.64 6.61 6.42 6.36 5.84 5.82 5.80 5.79 5.77
3.77 3.75 3.66 3.64
Cl
8s
6.17 1.93 1.98 1.88 2.17 5.02 1.04 0.89 0.77 0.99 2.24 1.72
9.0 8.5 8.0 7.5 7.0 6.5 6.0 5.5 5.0 4.5 4.0 3.5 3.0 2.5 2.0 1.5 1.0 0.5 0.0
f1 (ppm)

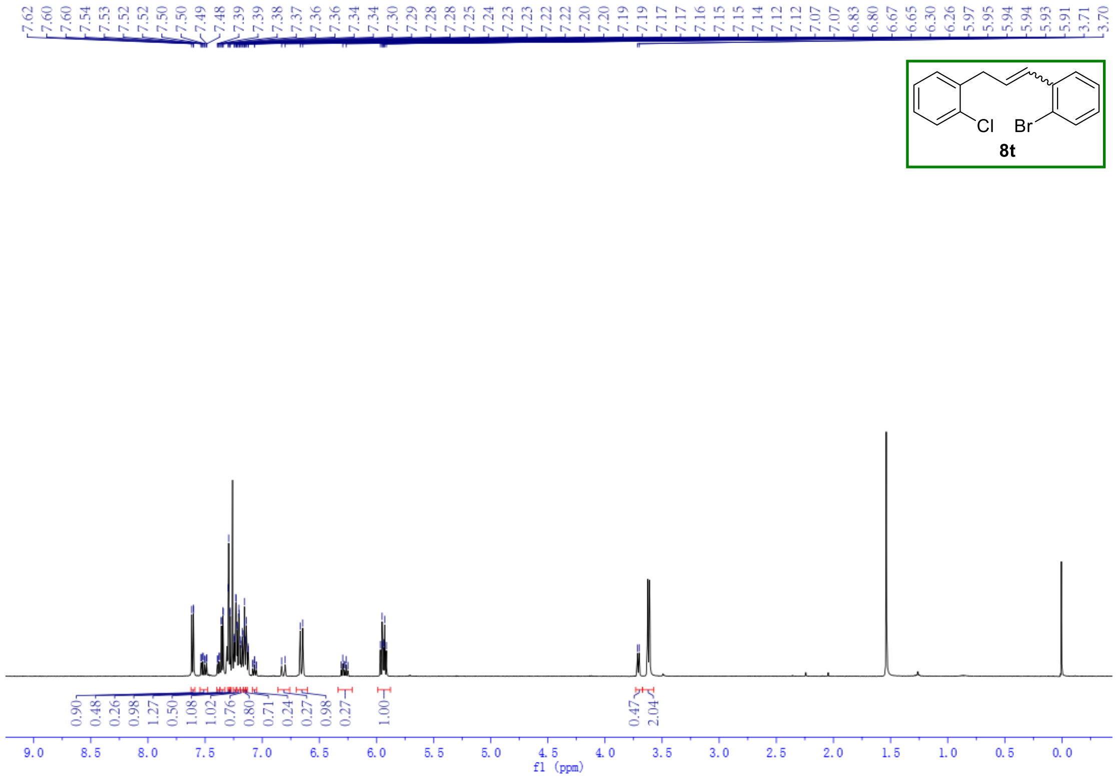
7.62 7.60 7.60 7.54 7.53 7.52 7.52 7.50 7.50 7.49 7.48 7.39 7.39 7.38 7.37 7.36 7.36 7.34 7.30 7.29 7.28 7.28 7.25 7.24 7.23 7.23 7.22 7.22 7.20 7.20 7.19 7.19 7.17 7.17 7.16 7.15 7.15 7.14 7.12 7.12 7.07 7.07 6.83 6.80 6.67 6.65 6.30 6.26 5.97 5.95 5.94 5.94 5.93 5.91 3.71 3.70
Cl
Br
8t
0.90 0.48 0.26 0.98 1.27 0.50 1.08 1.02 0.76 0.80 0.71 0.24 0.27 0.98 0.27 1.00 0.47 2.04
9.0 8.5 8.0 7.5 7.0 6.5 6.0 5.5 5.0 4.5 4.0 3.5 3.0 2.5 2.0 1.5 1.0 0.5 0.0
f1 (ppm)

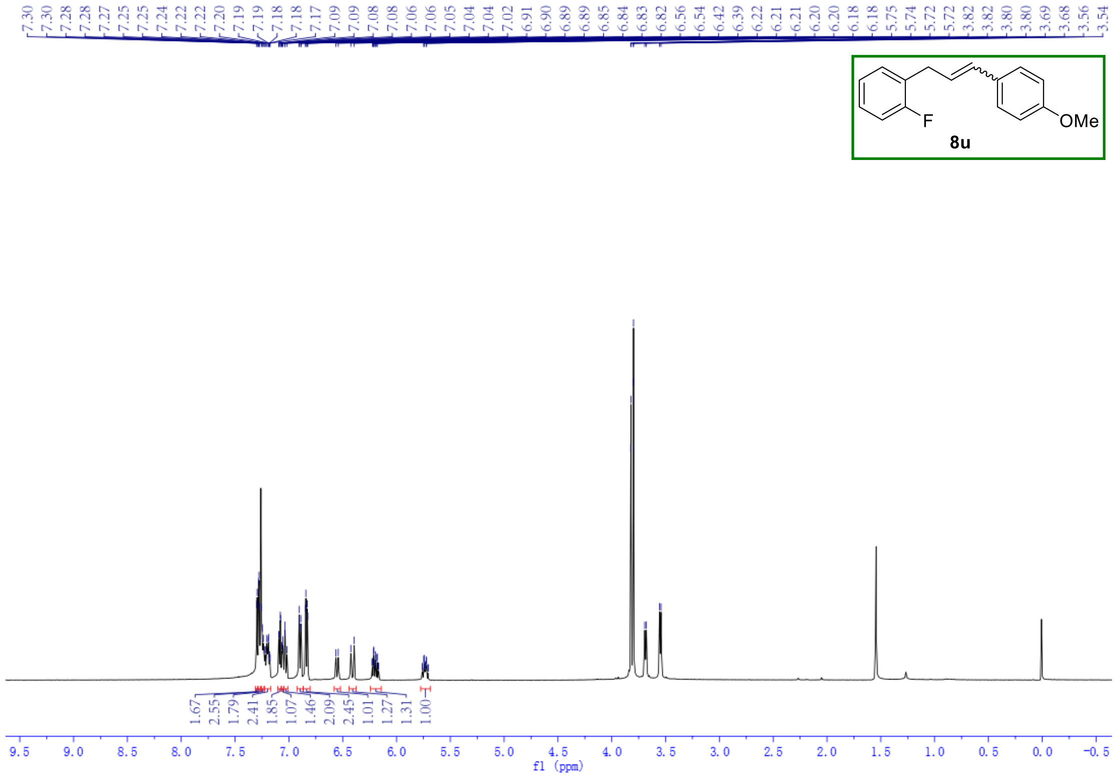
8u
OMe
F
f1 (ppm)

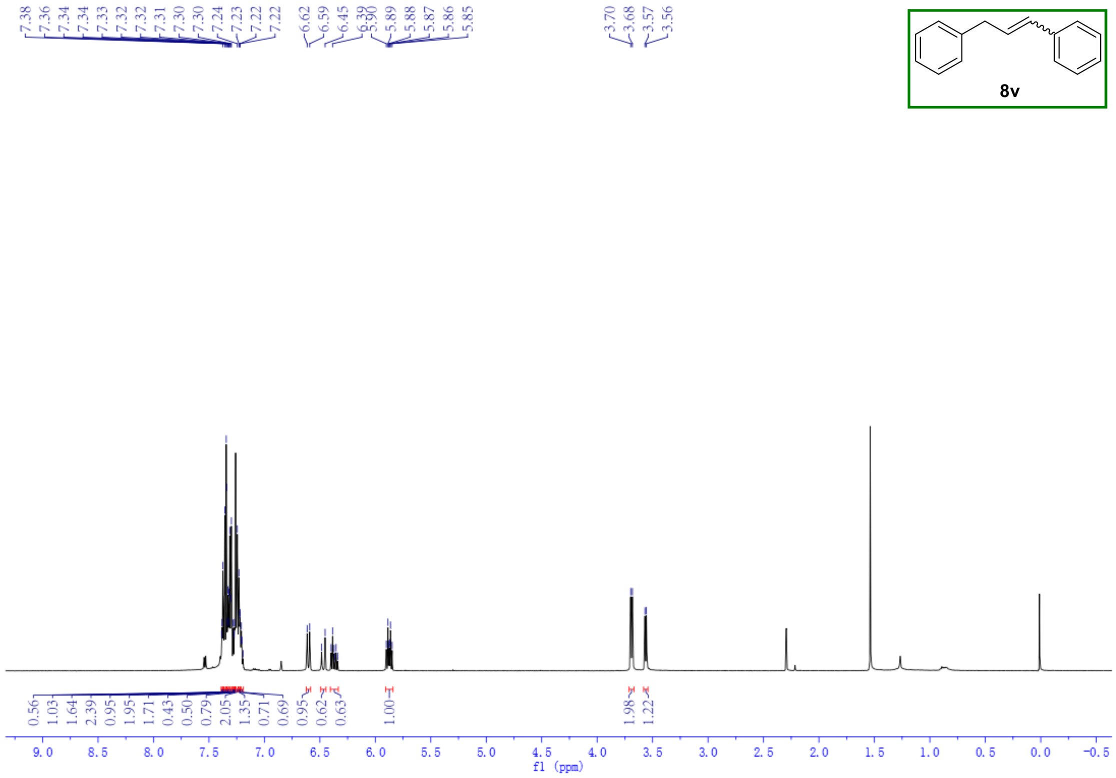
8v
f1 (ppm)

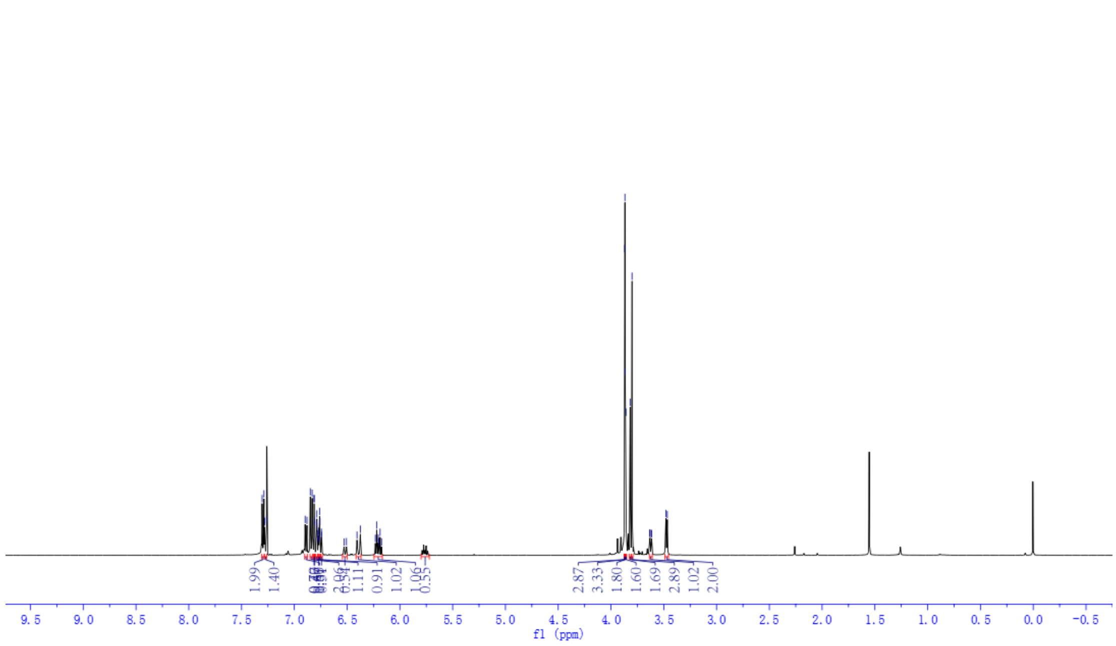

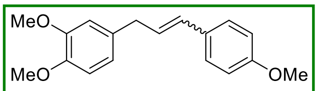

8w

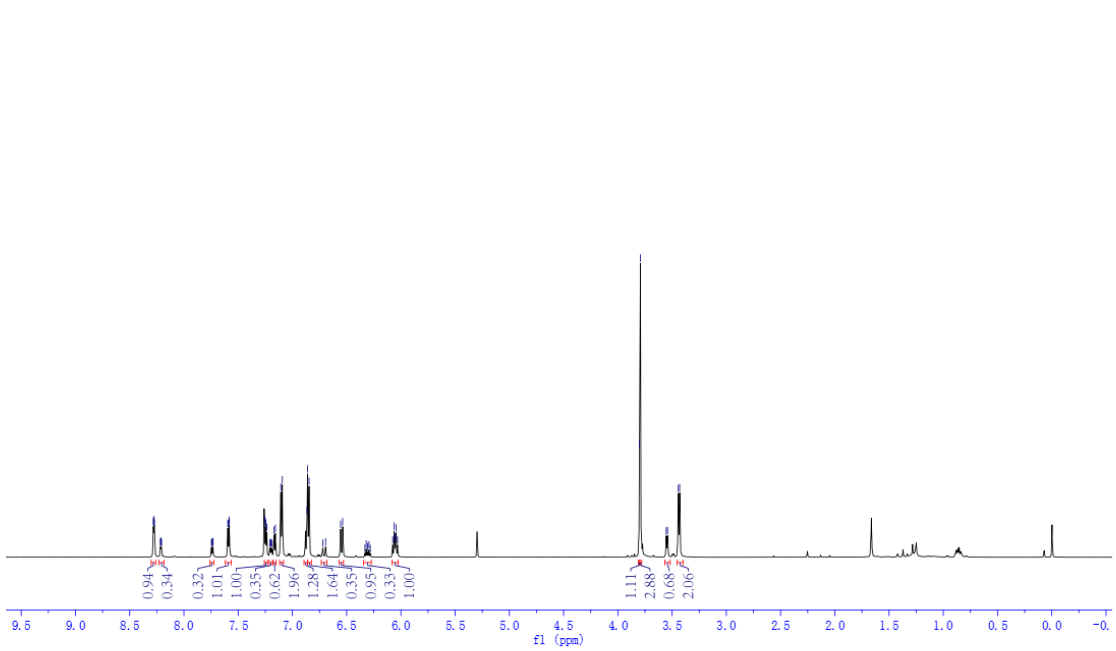

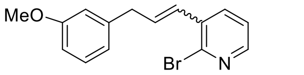

8x

NMR spectra of 7a-7v, 11a ,11a′ and 11b

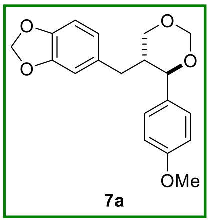

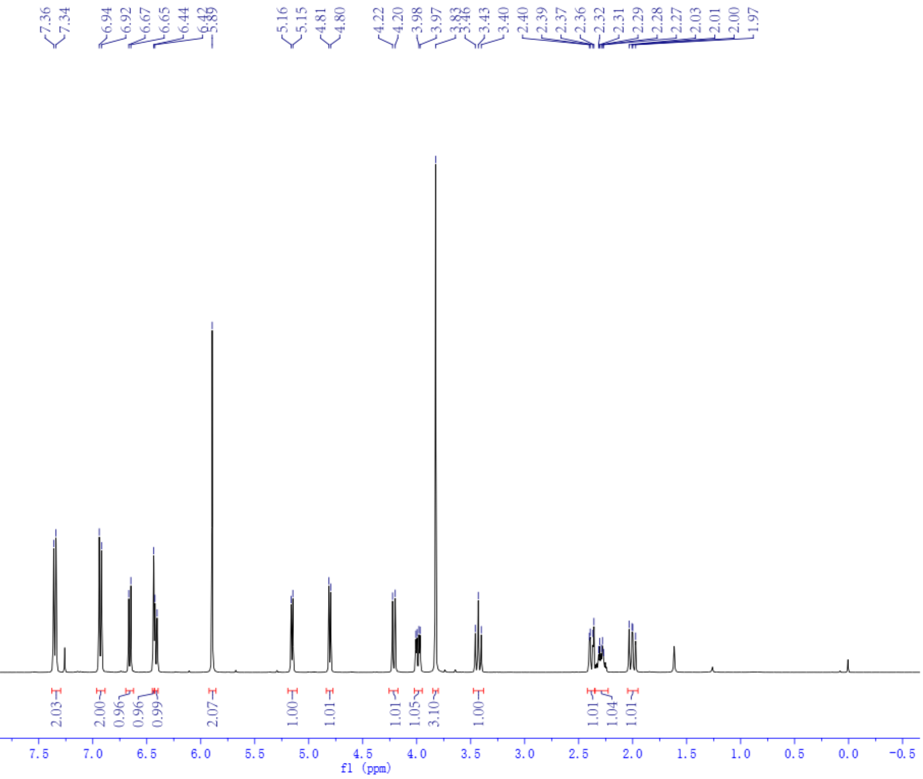

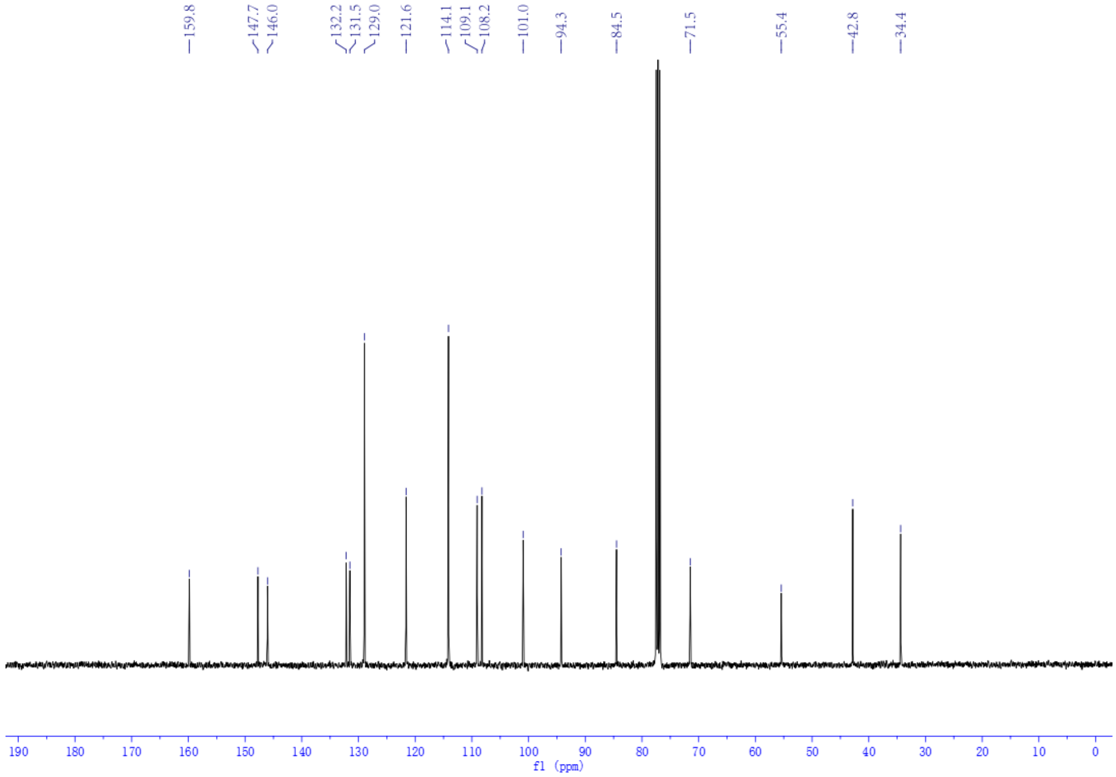

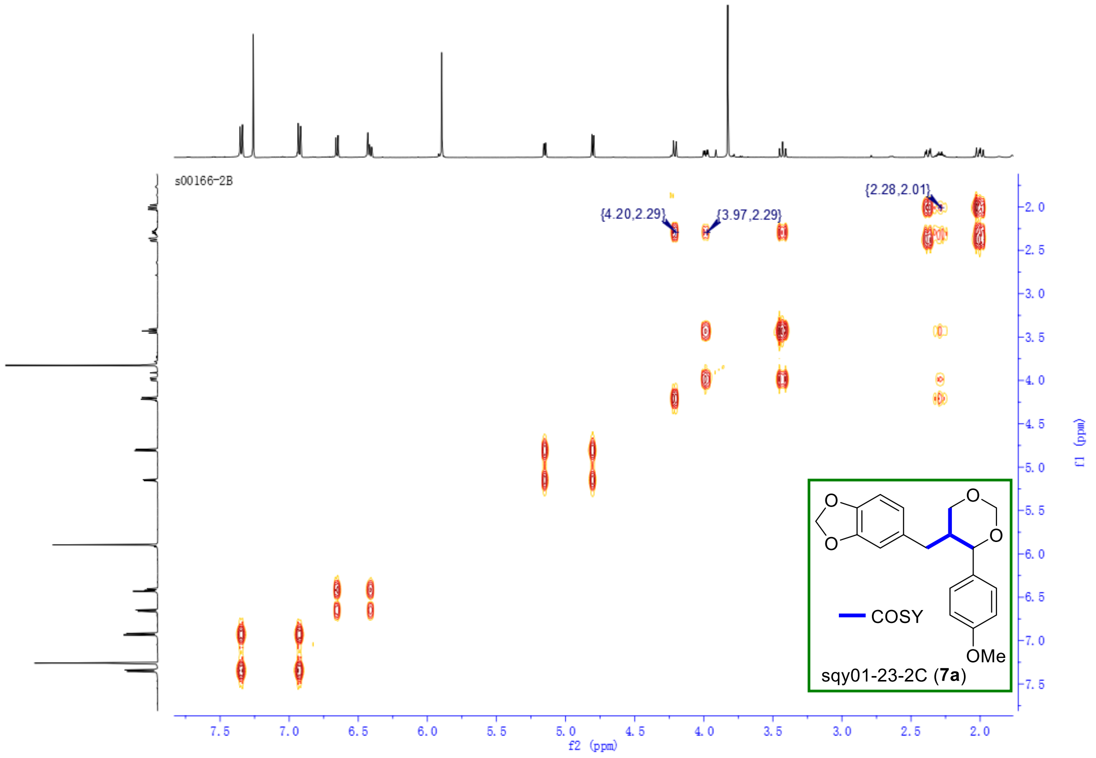
s00166-2B
{4.20,2.29}
{3.97,2.29}
{2.28,2.01}
COSY
OMe
sqy01-23-2C (7a)
f2 (ppm)
f1 (ppm)

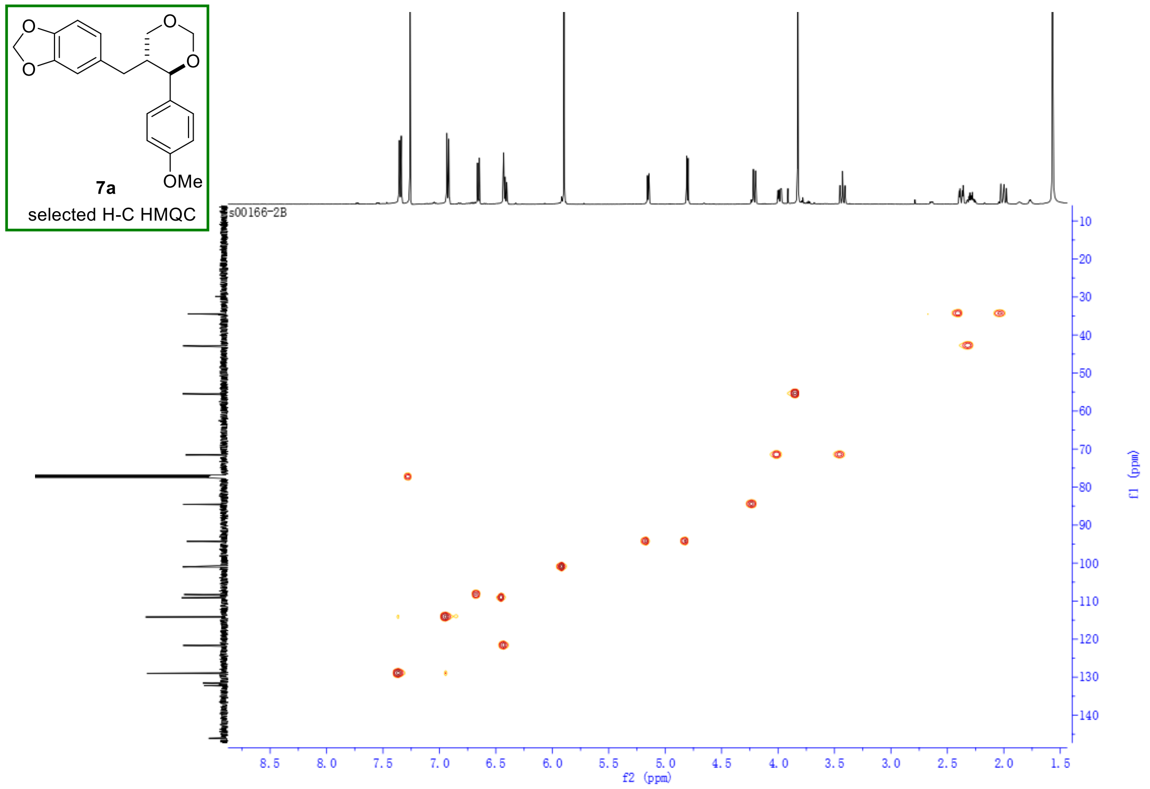
7a
OMe
selected H-C HMQC
s00166-2B
f2 (ppm)
f1 (ppm)

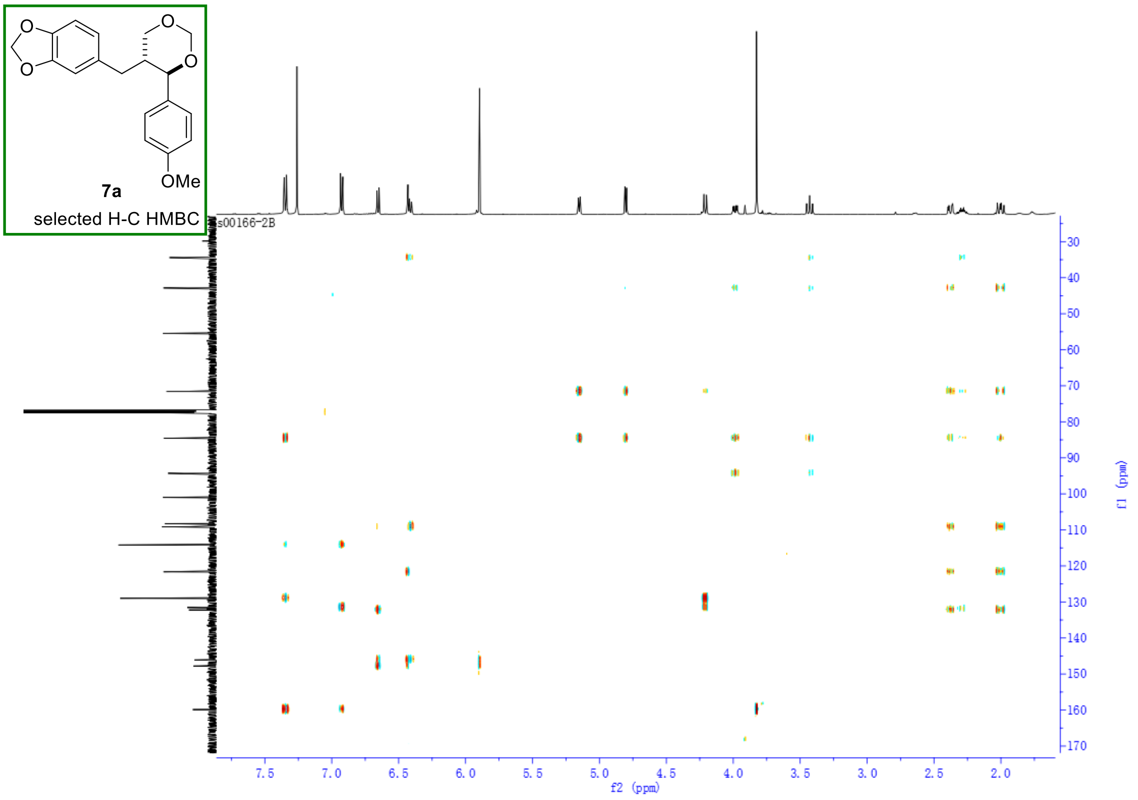
7a
OMe
selected H-C HMBC
s00166-2B
f2 (ppm)
f1 (ppm)

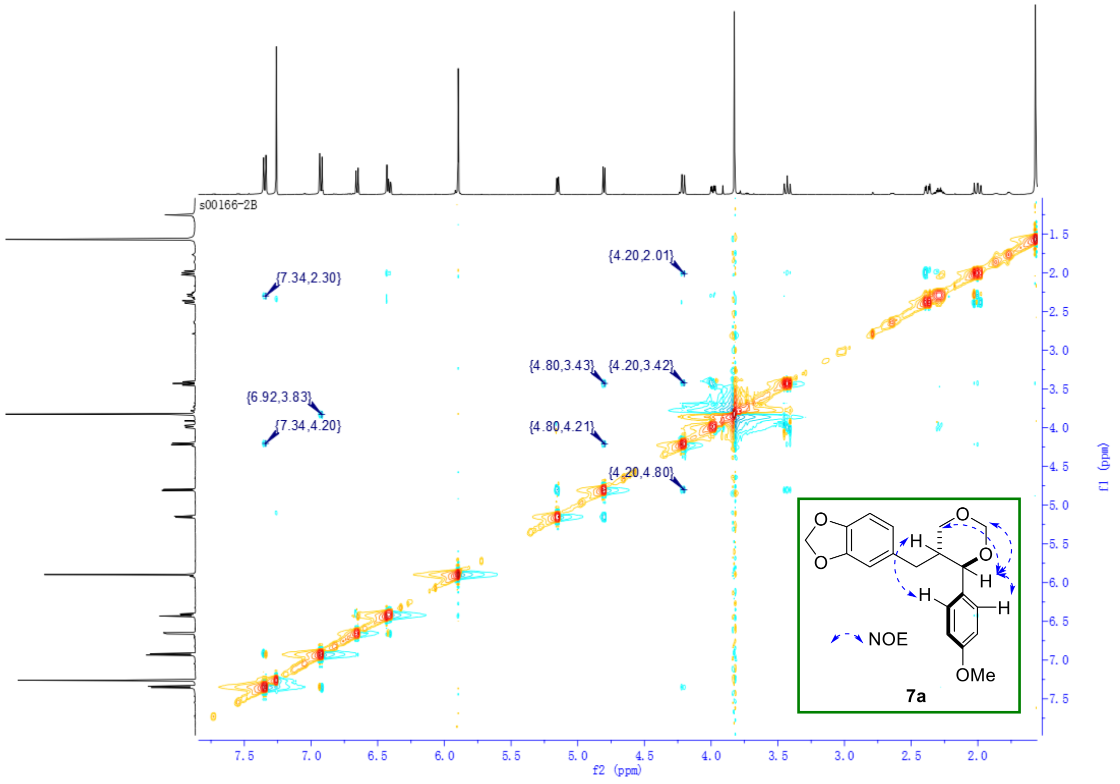
s00166-2B
{7.34,2.30}
{4.20,2.01}
{4.80,3.43}
{4.20,3.42}
{6.92,3.83}
{7.34,4.20}
{4.80,4.21}
{4.20,4.80}
NOE
OMe
7a
f2 (ppm)
f1 (ppm)

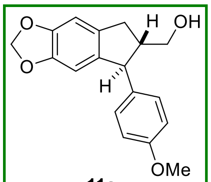
OH
H
H
OMe

11a

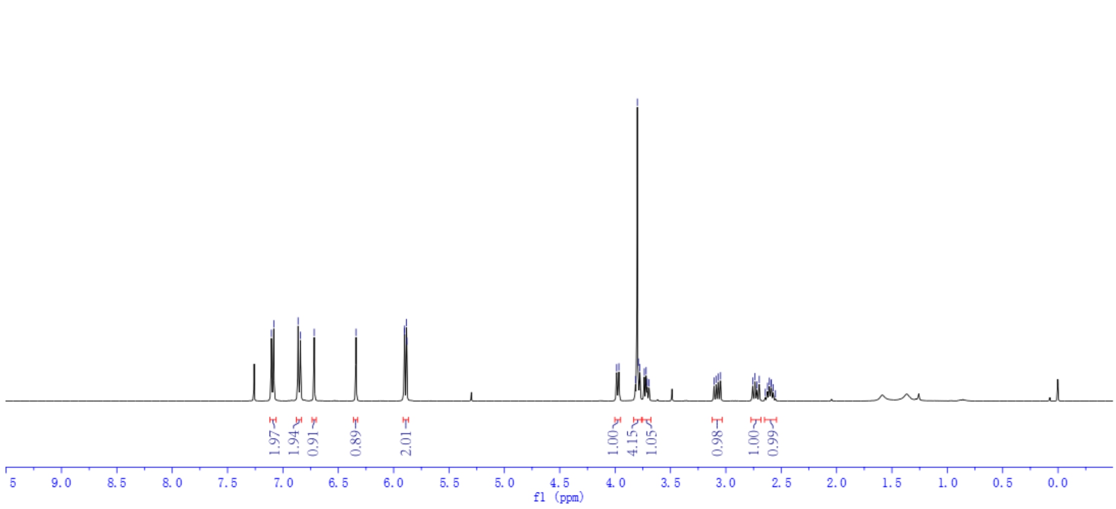
7.10
7.08
6.86
6.84
6.72
6.34
5.90
5.90
5.88
5.88
3.99
3.97
3.82
3.80
3.79
3.78
3.74
3.72
3.71
3.69
3.11
3.09
3.07
3.05
2.76
2.74
2.72
2.70
2.64
2.62
2.61
2.59
2.59
2.57
2.55
1.97
1.94
0.91
0.89
2.01
1.00
4.15
1.05
0.98
1.00
0.99
f1 (ppm)

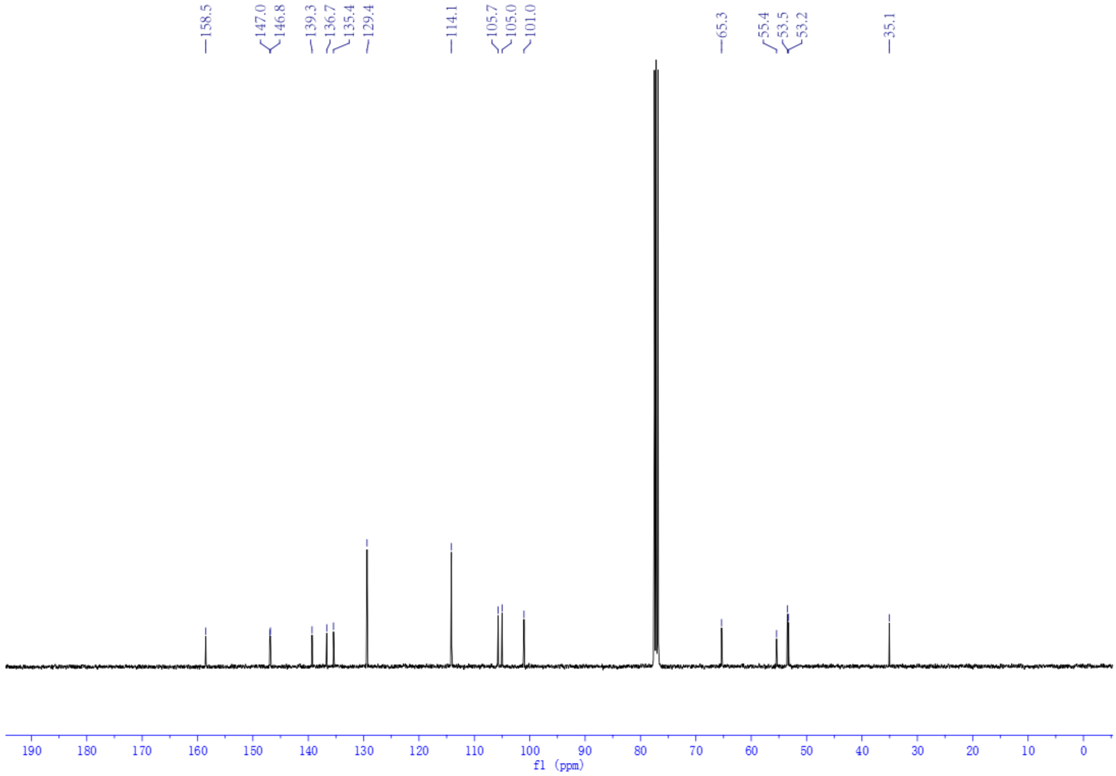
158.5
147.0
146.8
139.3
136.7
135.4
129.4
114.1
105.7
105.0
101.0
65.3
55.4
53.5
53.2
35.1
f1 (ppm)

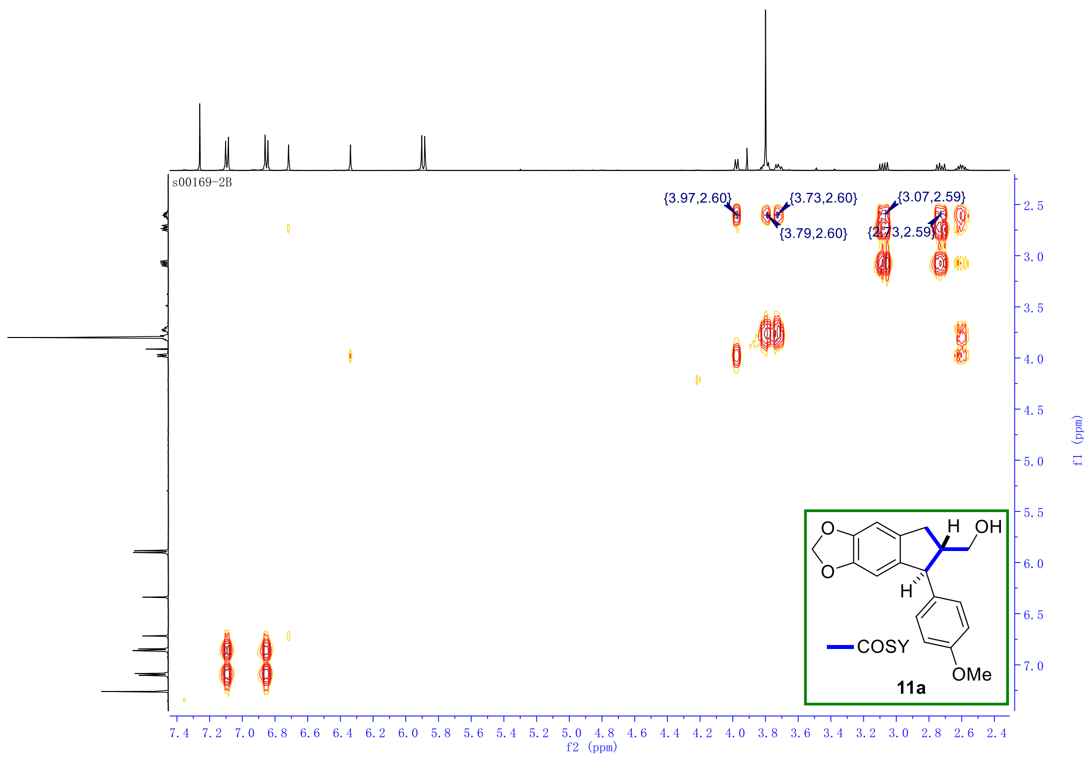
s00169-2B
{3.97,2.60}
{3.73,2.60}
{3.79,2.60}
{3.07,2.59}
{2.73,2.59}
COSY
11a
OMe
H
OH
f2 (ppm)
f1 (ppm)

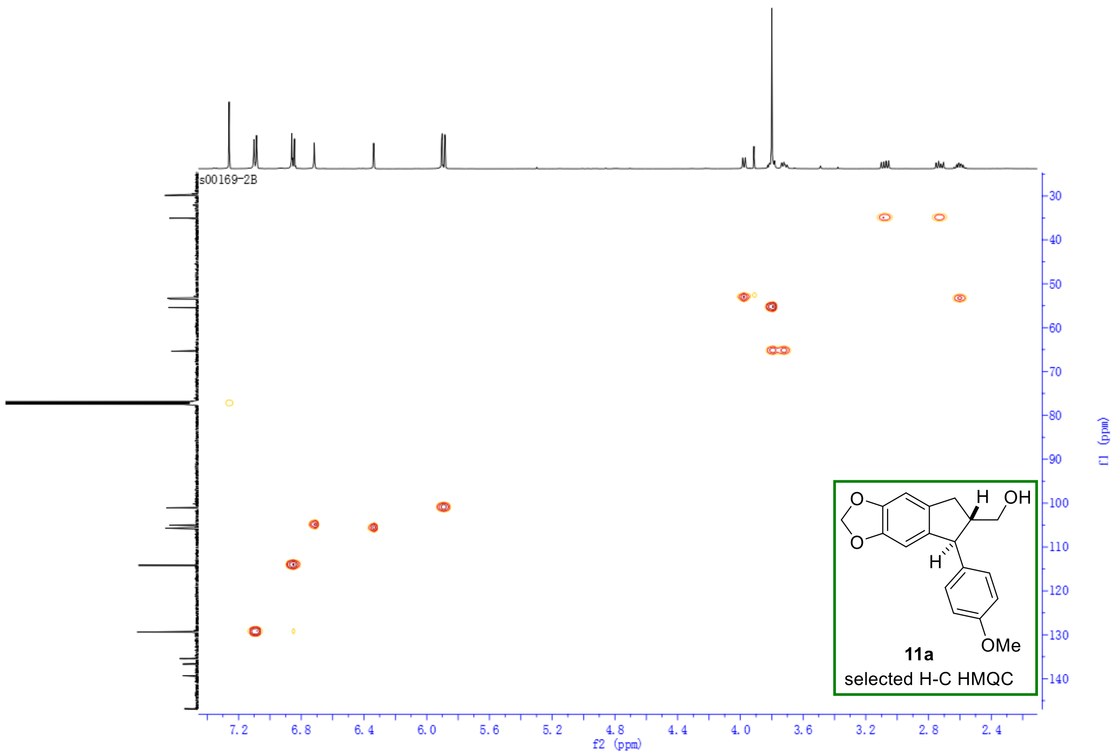
s00169-2B
11a
selected H-C HMQC
OMe
H
OH
f2 (ppm)
f1 (ppm)

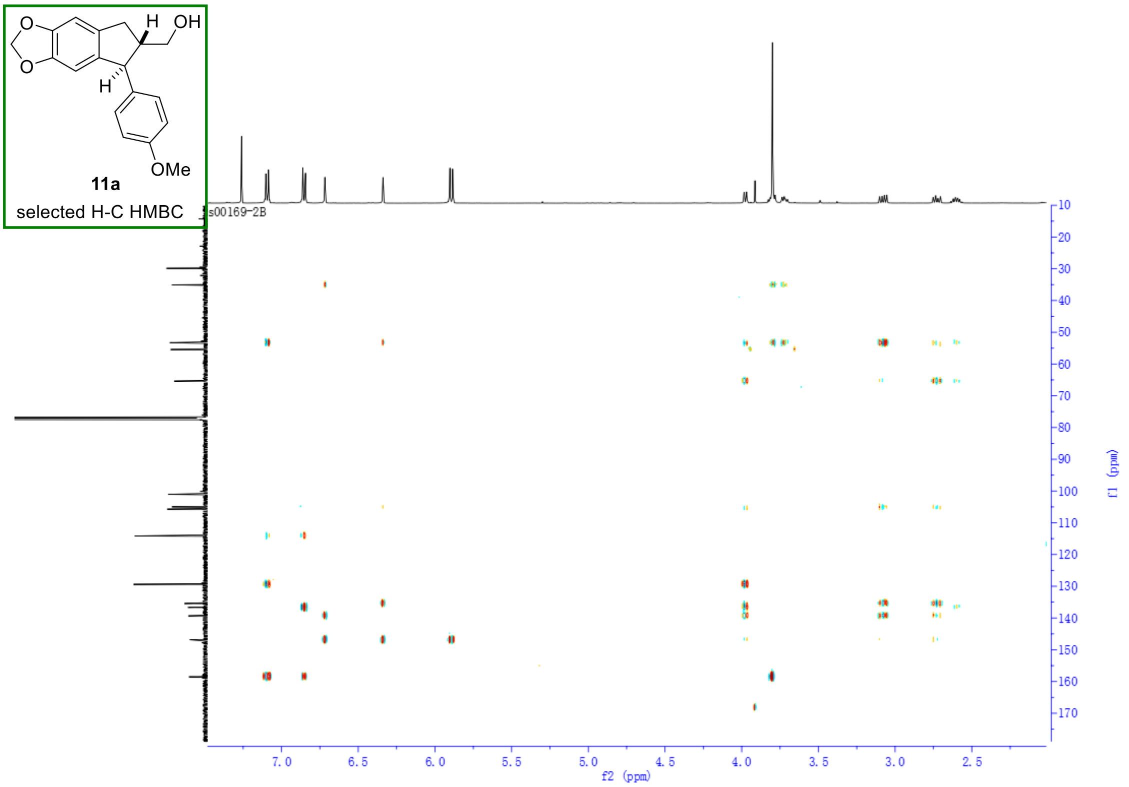
11a
selected H-C HMBC
s00169-2B
f2 (ppm)
f1 (ppm)

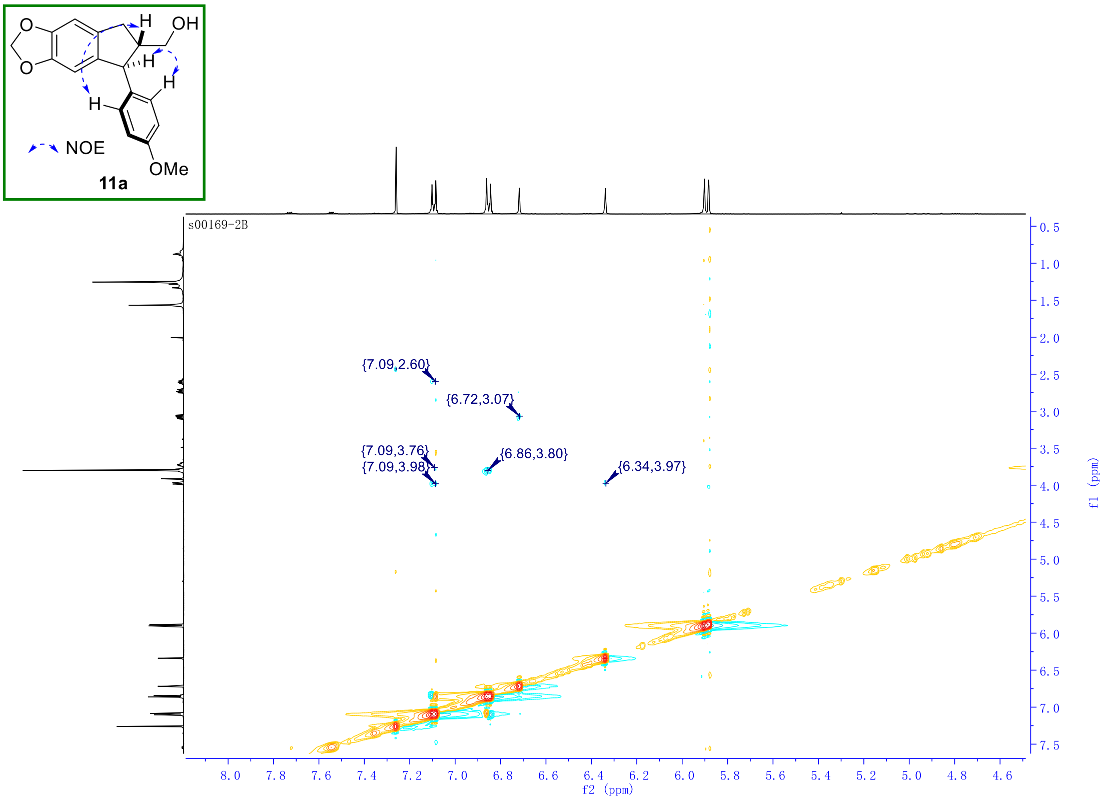
NOE
11a
s00169-2B
{7.09,2.60}
{6.72,3.07}
{7.09,3.76}
{7.09,3.98}
{6.86,3.80}
{6.34,3.97}
f2 (ppm)
f1 (ppm)

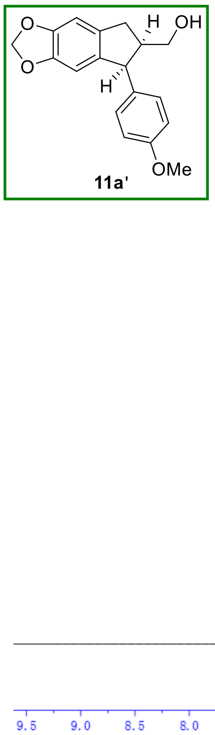

H
OH
H
OMe

11a'

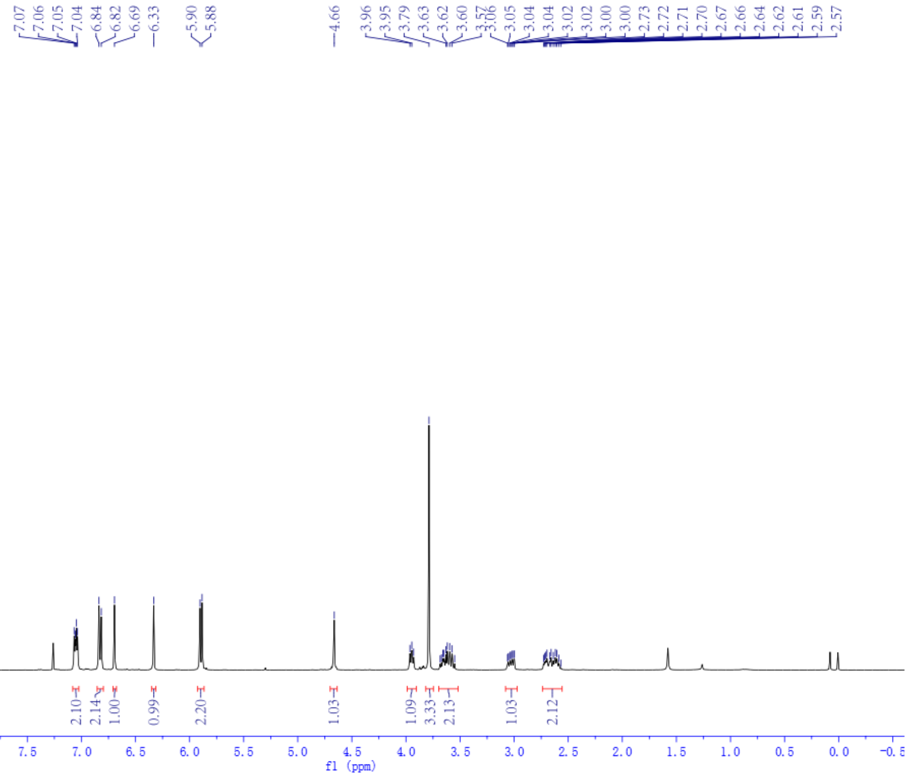

7.07 7.06 7.05 7.04 6.84 6.82 6.69 6.33 5.90 5.88 4.66 3.96 3.95 3.79 3.63 3.62 3.60 3.57 3.06 3.05 3.04 3.04 3.02 3.02 3.00 3.00 2.73 2.72 2.71 2.70 2.67 2.66 2.64 2.62 2.61 2.59 2.57
2.10 2.14 1.00 0.99 2.20 1.03 1.09 3.33 2.13 1.03 2.12
f1 (ppm)

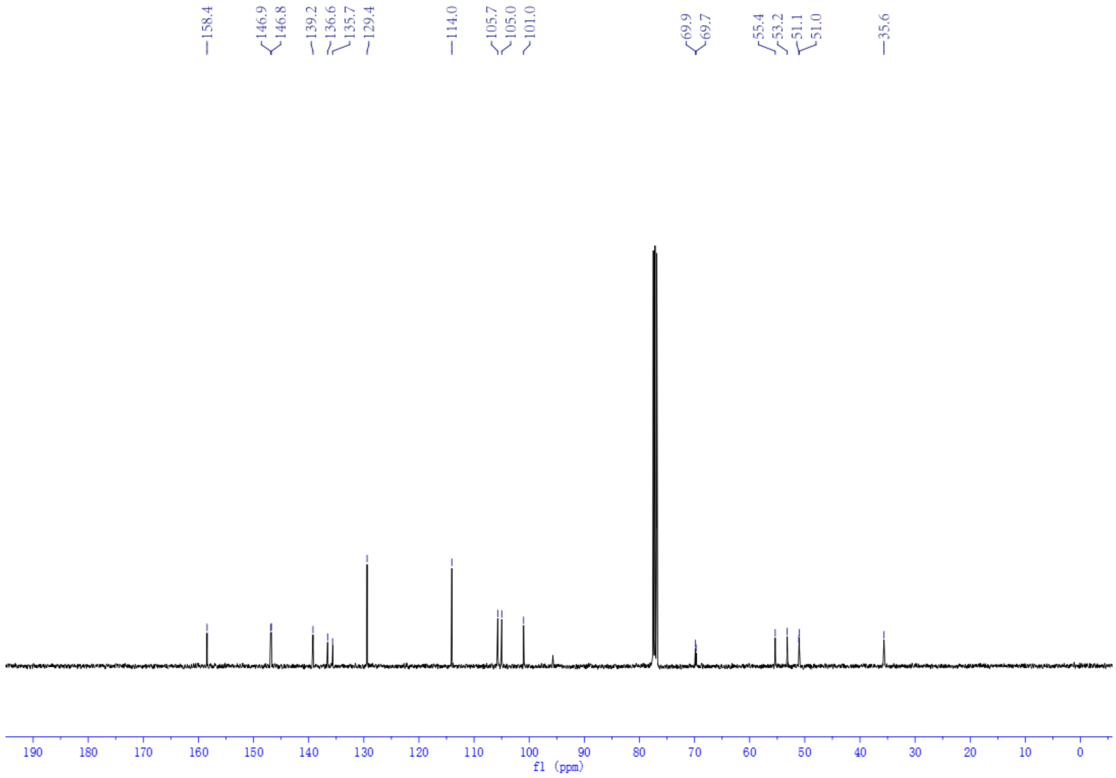

158.4 146.9 146.8 139.2 136.6 135.7 129.4 114.0 105.7 105.0 101.0 69.9 69.7 55.4 53.2 51.1 51.0 35.6
f1 (ppm)

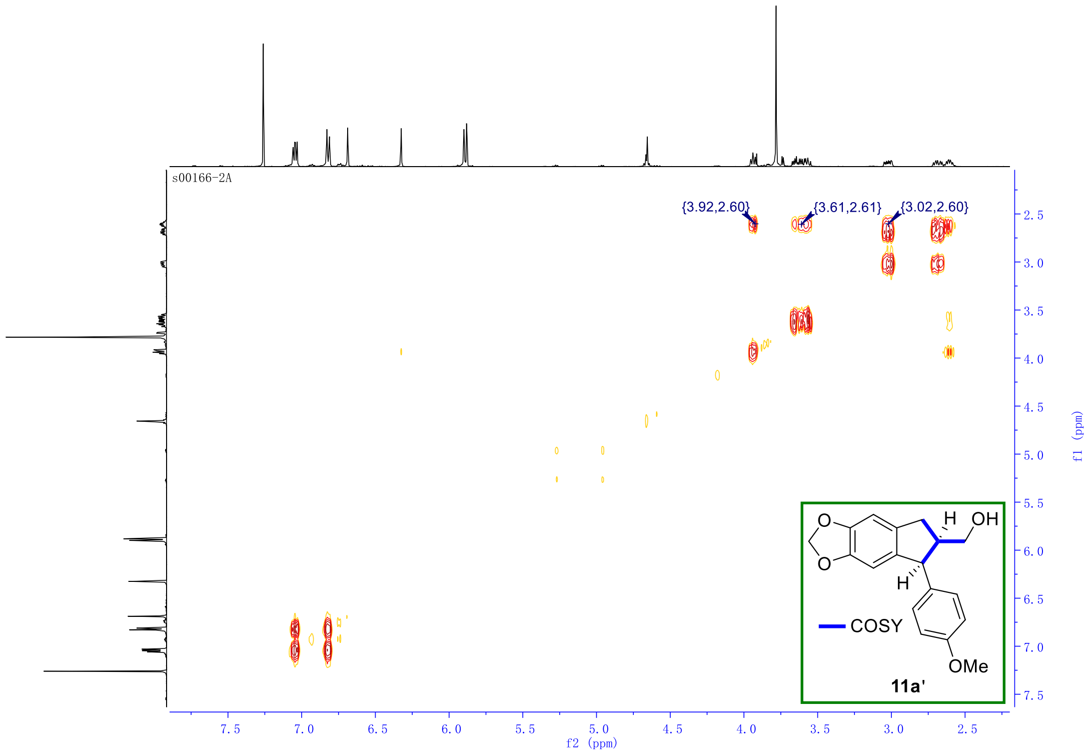
s00166-2A
{3.92,2.60}
{3.61,2.61}
{3.02,2.60}
COSY
OMe
11a'
f2 (ppm)
f1 (ppm)

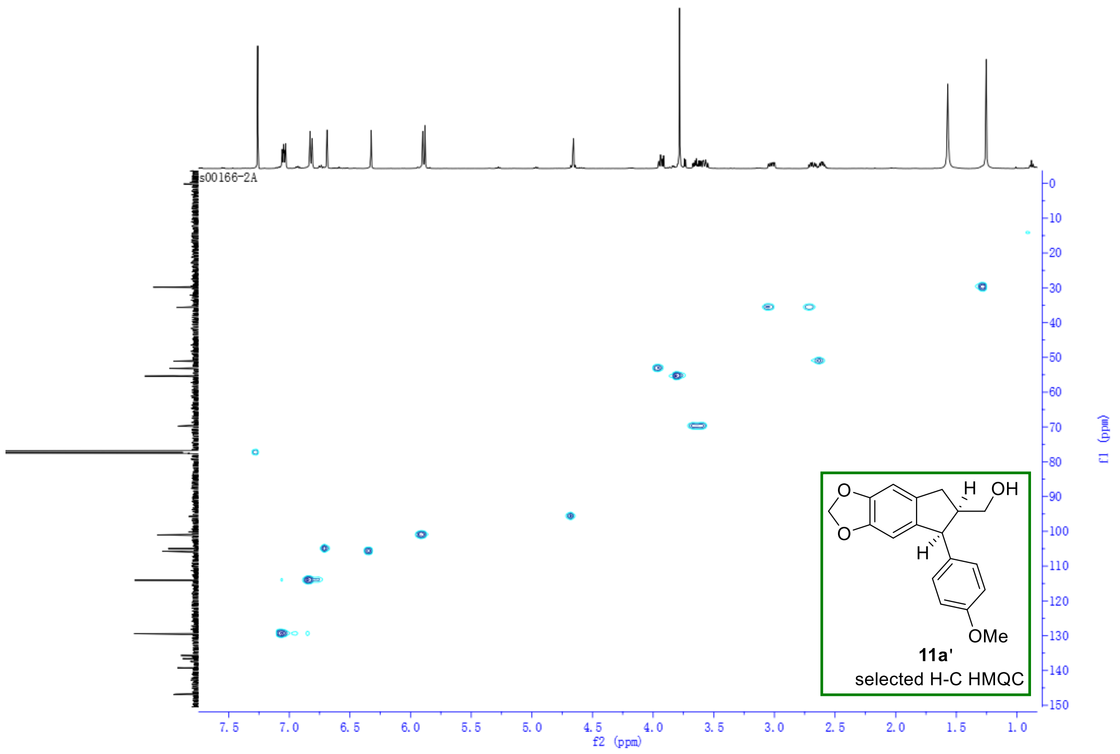
s00166-2A
OMe
11a'
selected H-C HMQC
f2 (ppm)
f1 (ppm)

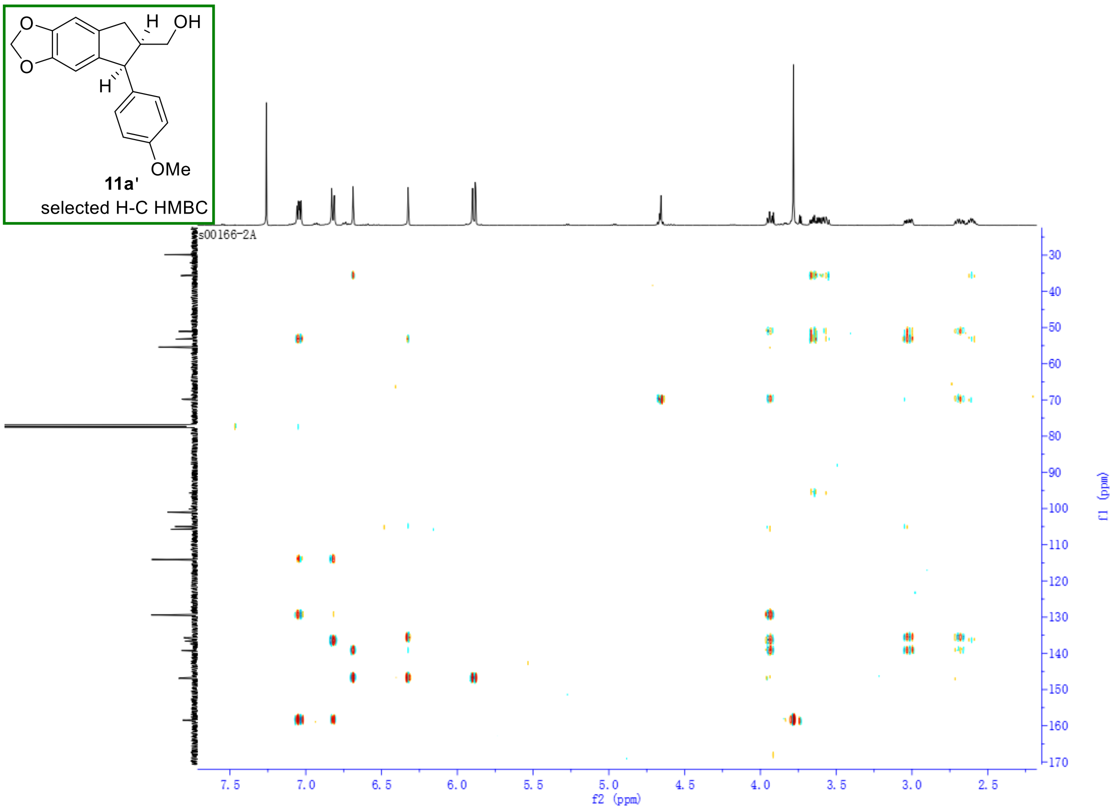

11a'
selected H-C HMBC
OH
OMe
s00166-2A
f2 (ppm)
f1 (ppm)

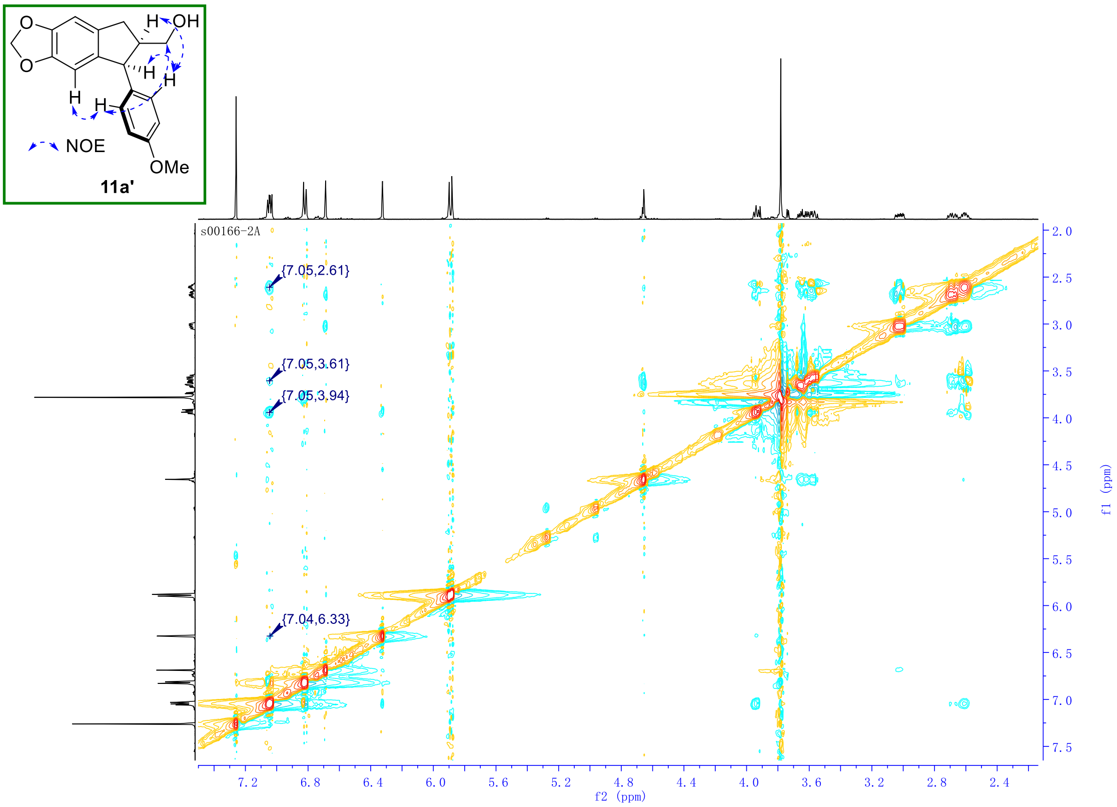

NOE
11a'
OH
OMe
s00166-2A
{7.05,2.61}
{7.05,3.61}
{7.05,3.94}
{7.04,6.33}
f2 (ppm)
f1 (ppm)

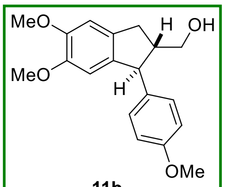
MeO
MeO
H
OH
H
OMe

11b

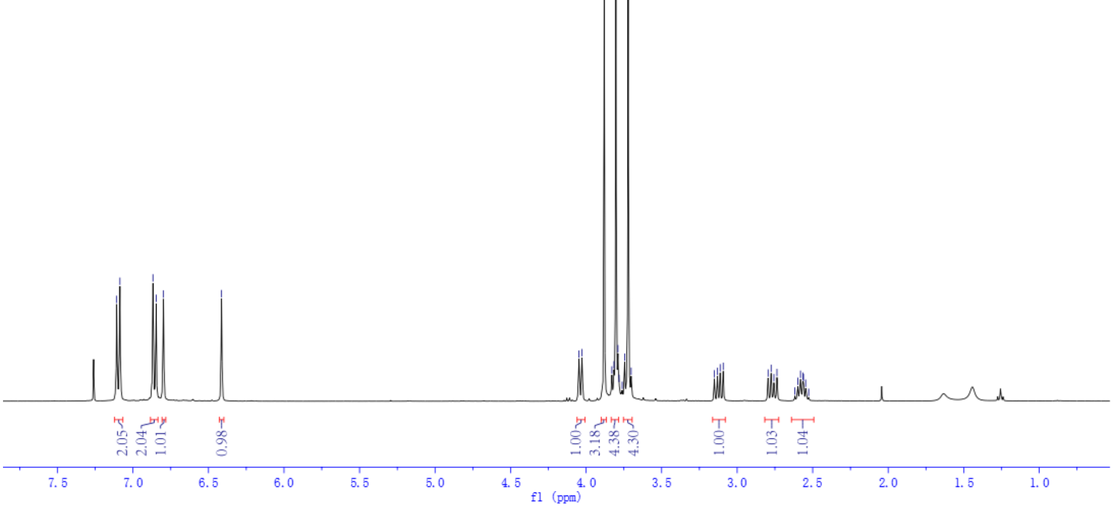
7.11
7.09
6.87
6.85
6.80
6.41
4.05
4.03
3.88
3.83
3.82
3.80
3.79
3.78
3.76
3.74
3.72
3.70
3.13
3.11
3.09
2.80
2.77
2.76
2.74
2.62
2.60
2.58
2.56
2.56
2.55
2.53
2.05
2.04
1.01
0.98
1.00
3.18
4.38
4.30
1.00
1.03
1.04
f1 (ppm)

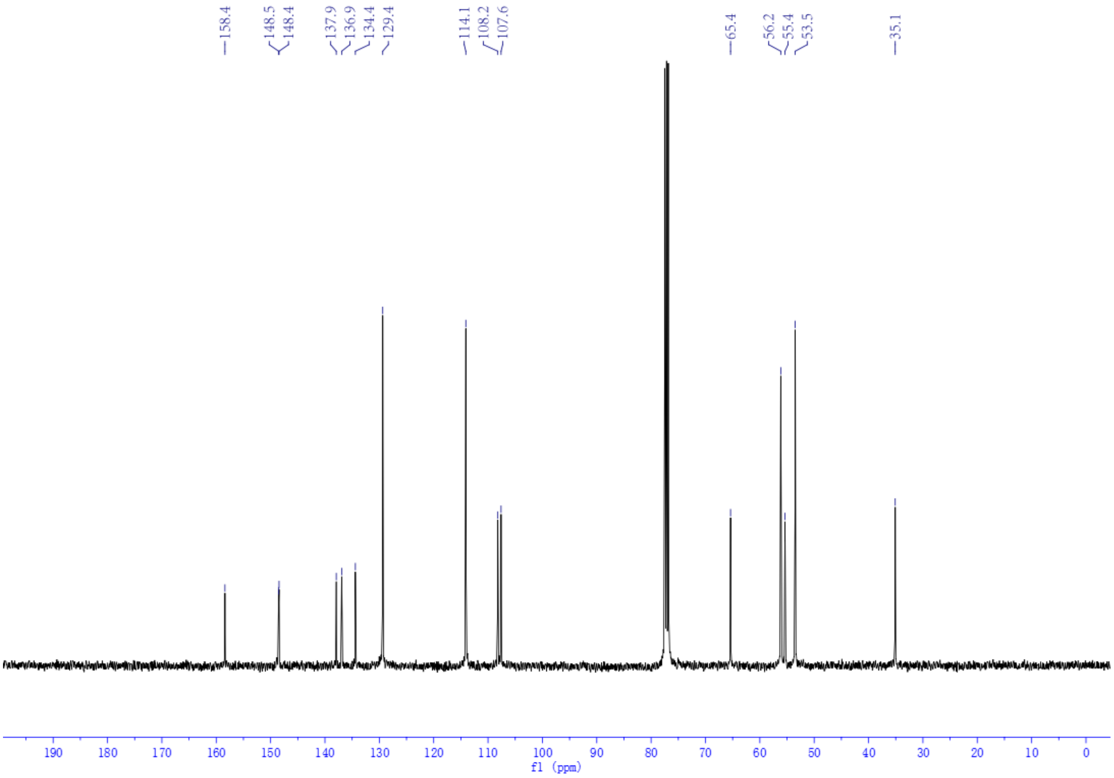
158.4
148.5
148.4
137.9
136.9
134.4
129.4
114.1
108.2
107.6
65.4
56.2
55.4
53.5
35.1
f1 (ppm)

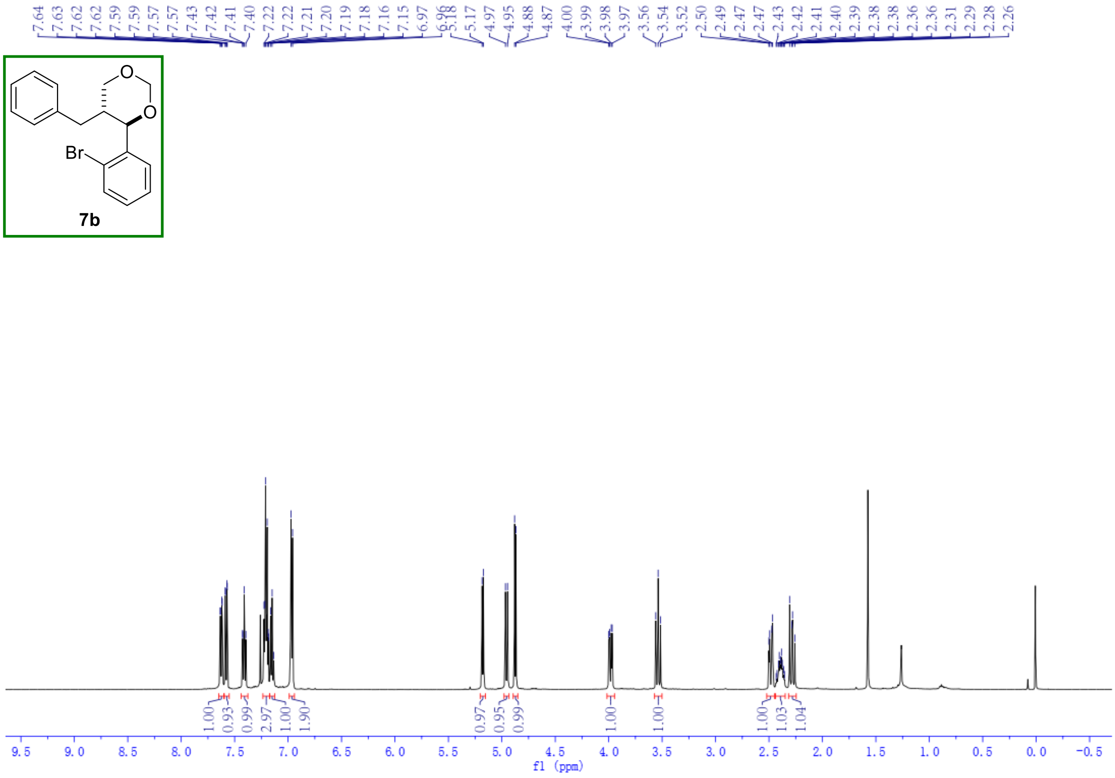
7b
7.64
7.63
7.62
7.62
7.59
7.59
7.57
7.57
7.43
7.42
7.41
7.40
7.22
7.22
7.21
7.20
7.19
7.18
7.16
7.15
6.97
6.96
5.18
5.17
4.97
4.95
4.88
4.87
4.00
3.99
3.98
3.97
3.56
3.54
3.52
2.50
2.49
2.47
2.47
2.43
2.42
2.41
2.40
2.39
2.38
2.38
2.36
2.36
2.31
2.29
2.28
2.26
1.00
0.93
0.99
2.97
1.00
1.90
0.97
0.95
0.99
1.00
1.00
1.00
1.03
1.04
f1 (ppm)

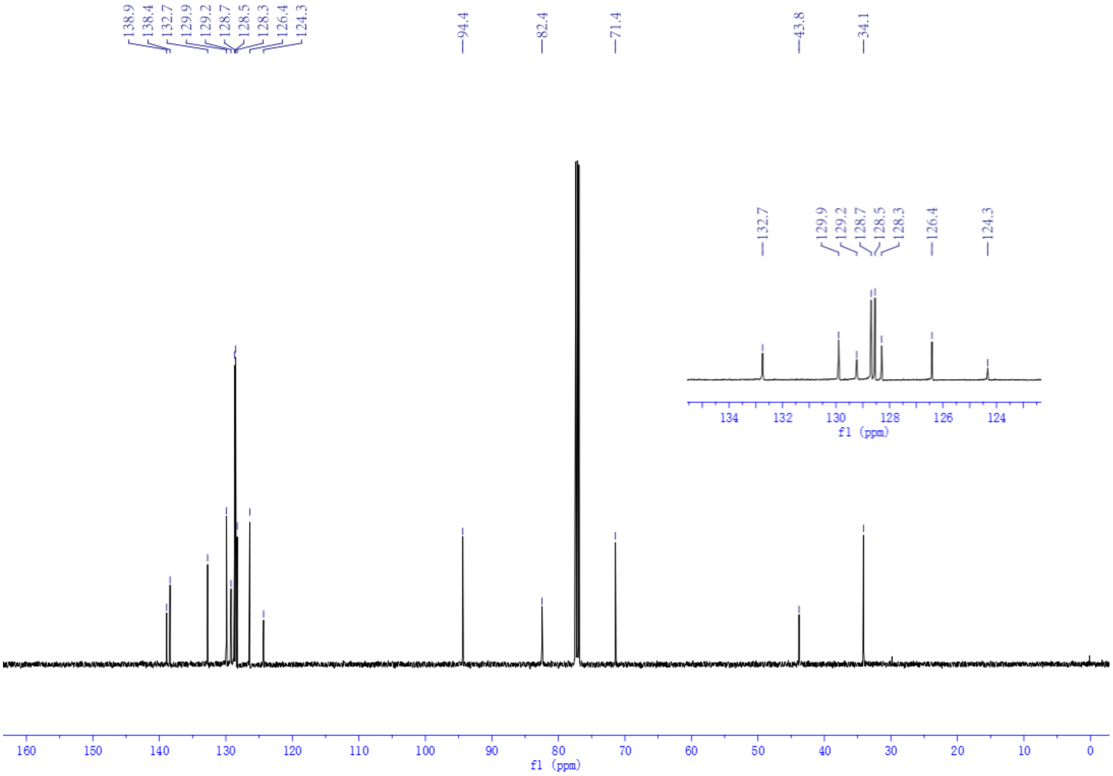
138.9
138.4
132.7
129.9
129.2
128.7
128.5
128.3
126.4
124.3
94.4
82.4
71.4
43.8
34.1
f1 (ppm)

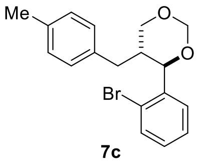
Me
O
O
Br

7c

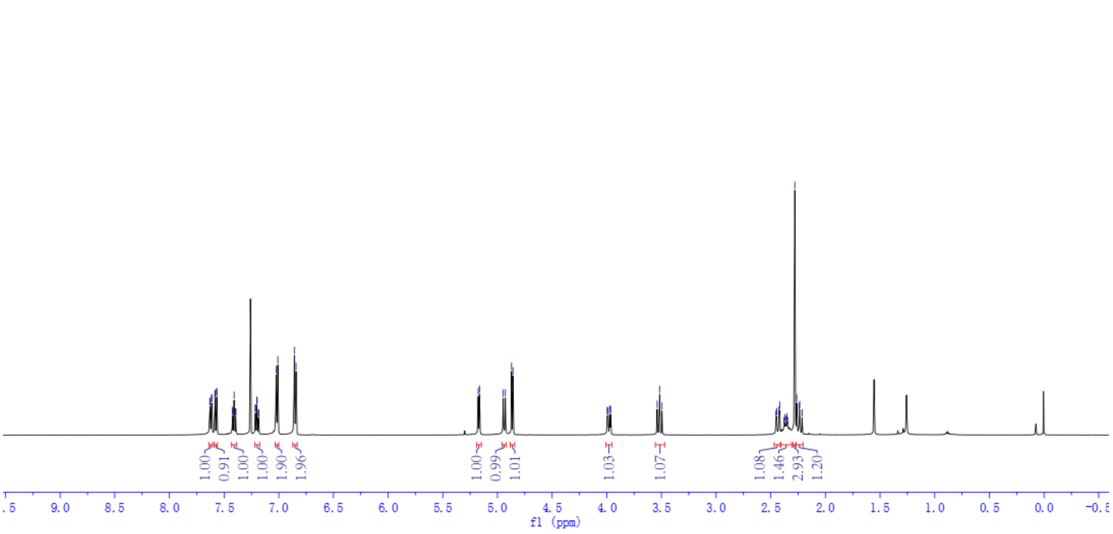
7.63 7.63 7.62 7.61 7.58 7.58 7.57 7.57 7.42 7.42 7.41 7.39 7.39 7.22 7.21 7.20 7.20 7.19 7.02 7.01 6.86 6.84 5.18 5.16 4.95 4.93 4.87 4.86 4.00 3.99 3.97 3.96 3.54 3.52 3.49 2.45 2.45 2.42 2.42 2.38 2.37 2.36 2.35 2.34 2.28 2.26 2.24 2.21
1.00 0.91 1.00 1.00 1.90 1.96 1.00 0.99 1.01 1.03 1.07 1.08 1.46 2.93 1.20
f1 (ppm)

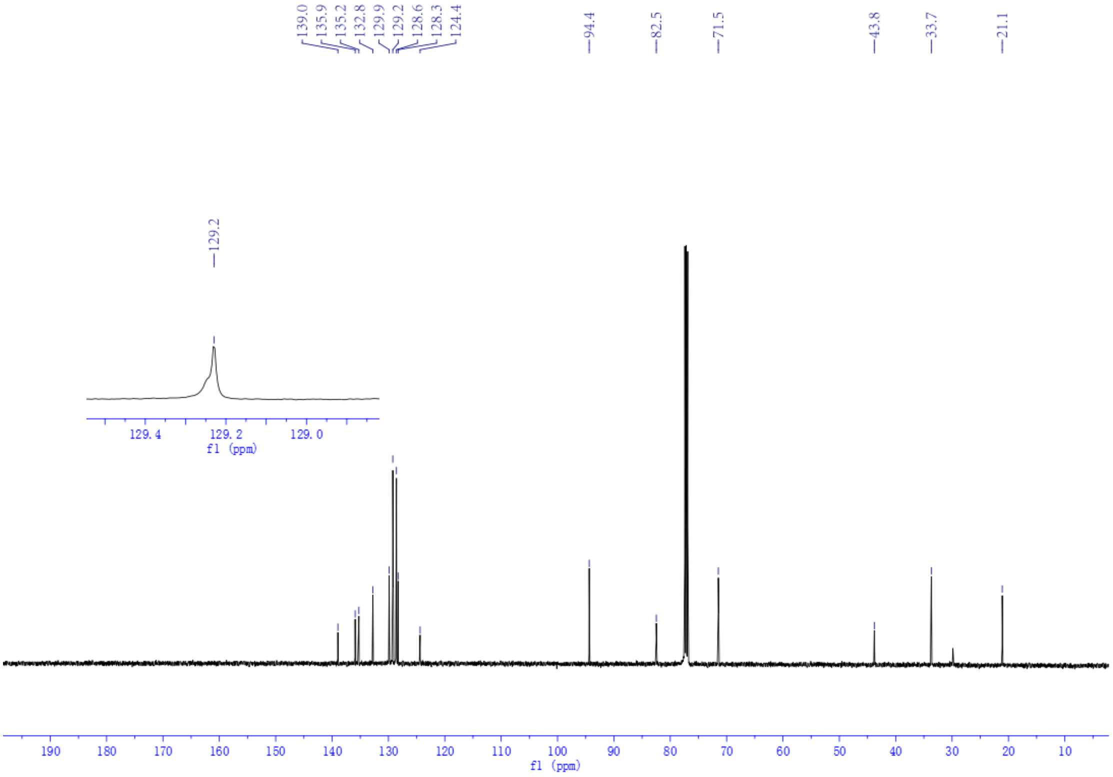
139.0 135.9 135.2 132.8 129.9 129.2 128.6 128.3 124.4
94.4
82.5
71.5
43.8
33.7
21.1
129.2
f1 (ppm)
f1 (ppm)

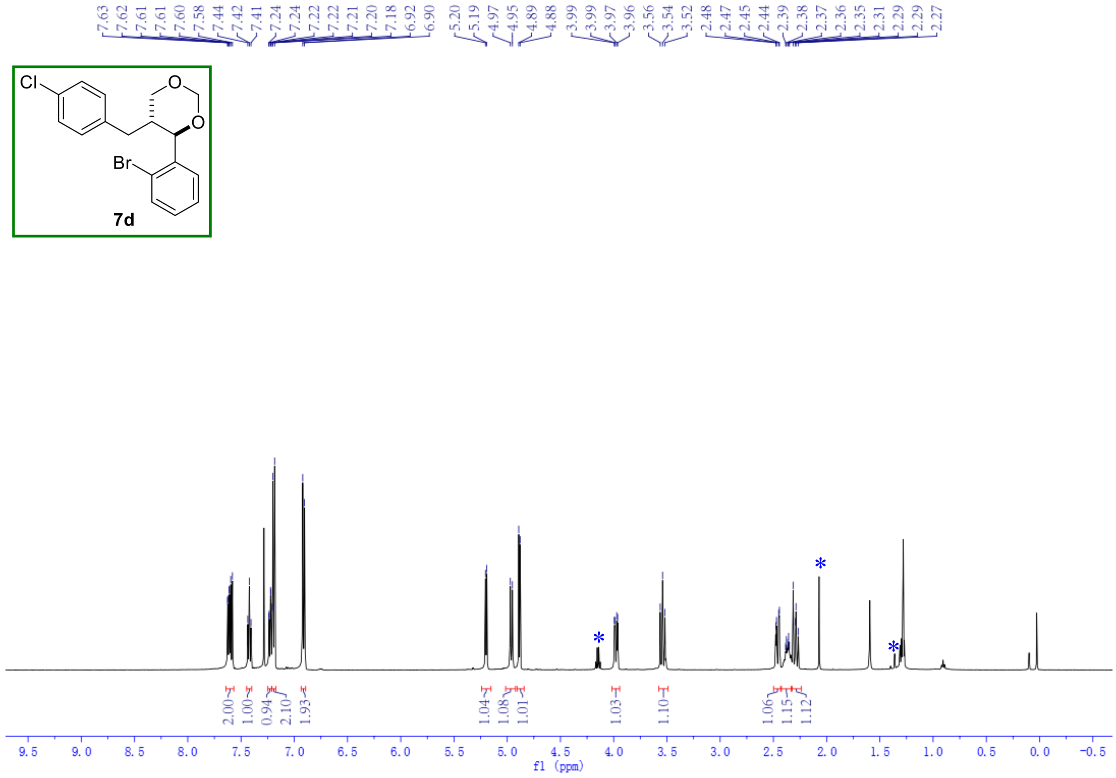
7.63 7.62 7.61 7.61 7.60 7.58 7.44 7.42 7.41 7.24 7.24 7.22 7.22 7.21 7.20 7.18 6.92 6.90 5.20 5.19 4.97 4.95 4.89 4.88 3.99 3.99 3.97 3.96 3.56 3.54 3.52 2.48 2.47 2.45 2.44 2.39 2.38 2.37 2.36 2.35 2.31 2.29 2.29 2.27
Cl
O
O
Br
7d
2.00 1.00 0.94 2.10 1.93 1.04 1.08 1.01 1.03 1.10 1.06 1.15 1.12
f1 (ppm)

* EtOAc

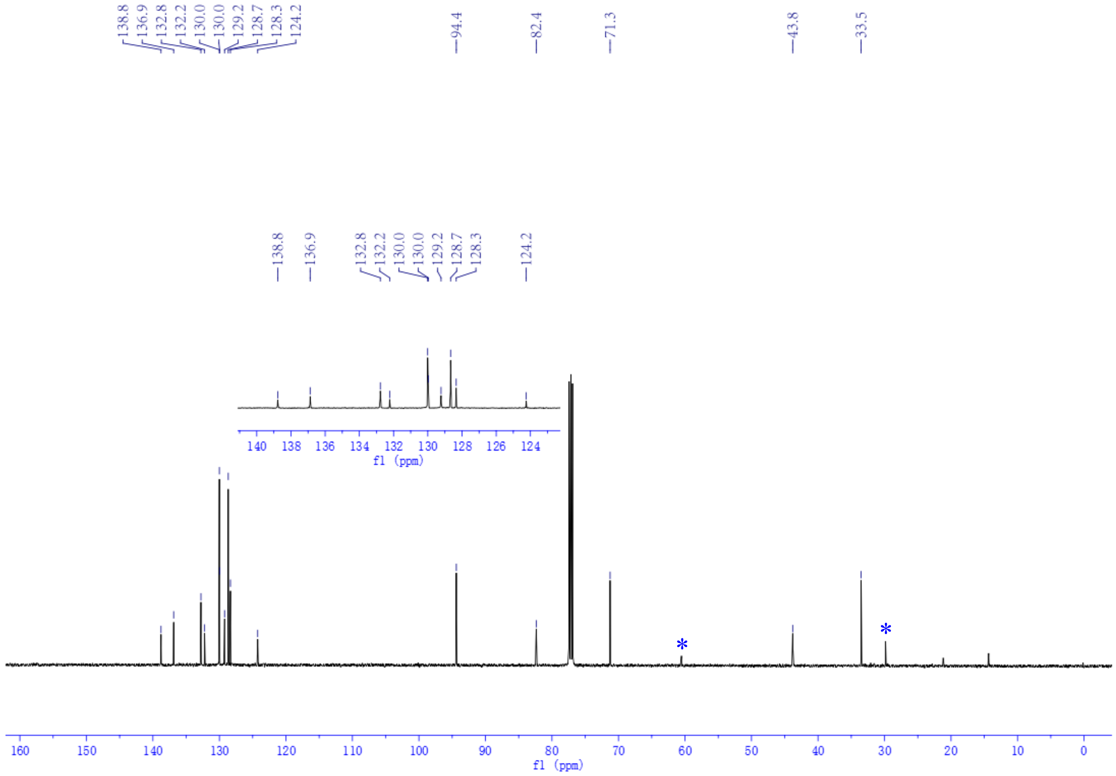
138.8 136.9 132.8 132.2 130.0 130.0 129.2 128.7 128.3 124.2
94.4
82.4
71.3
43.8
33.5
f1 (ppm)

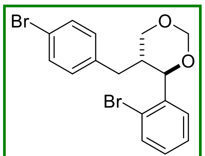

7e

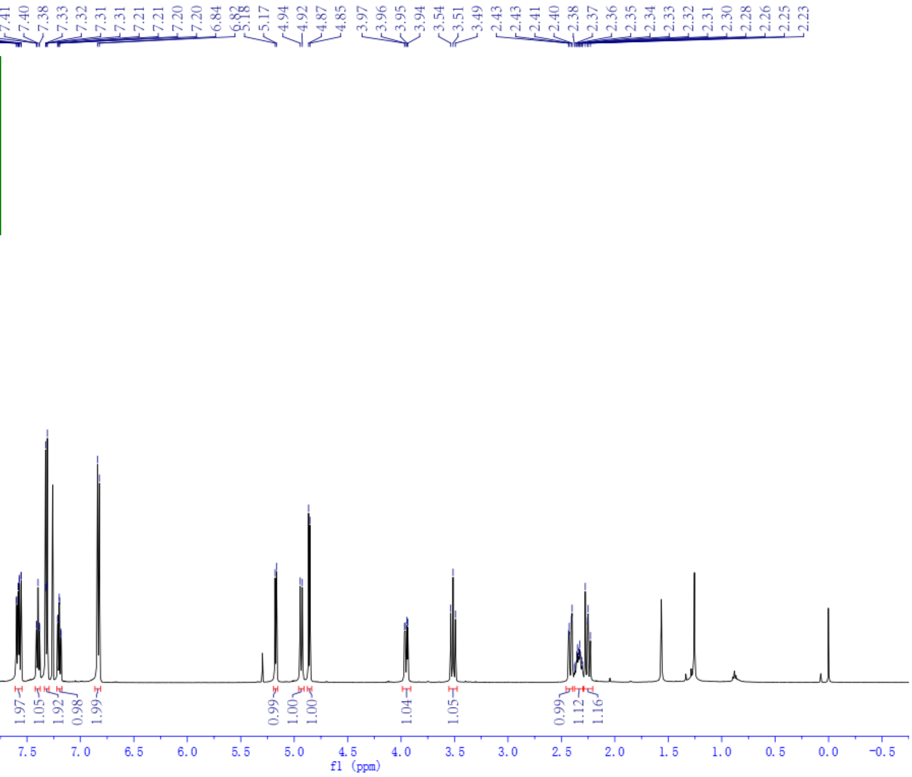

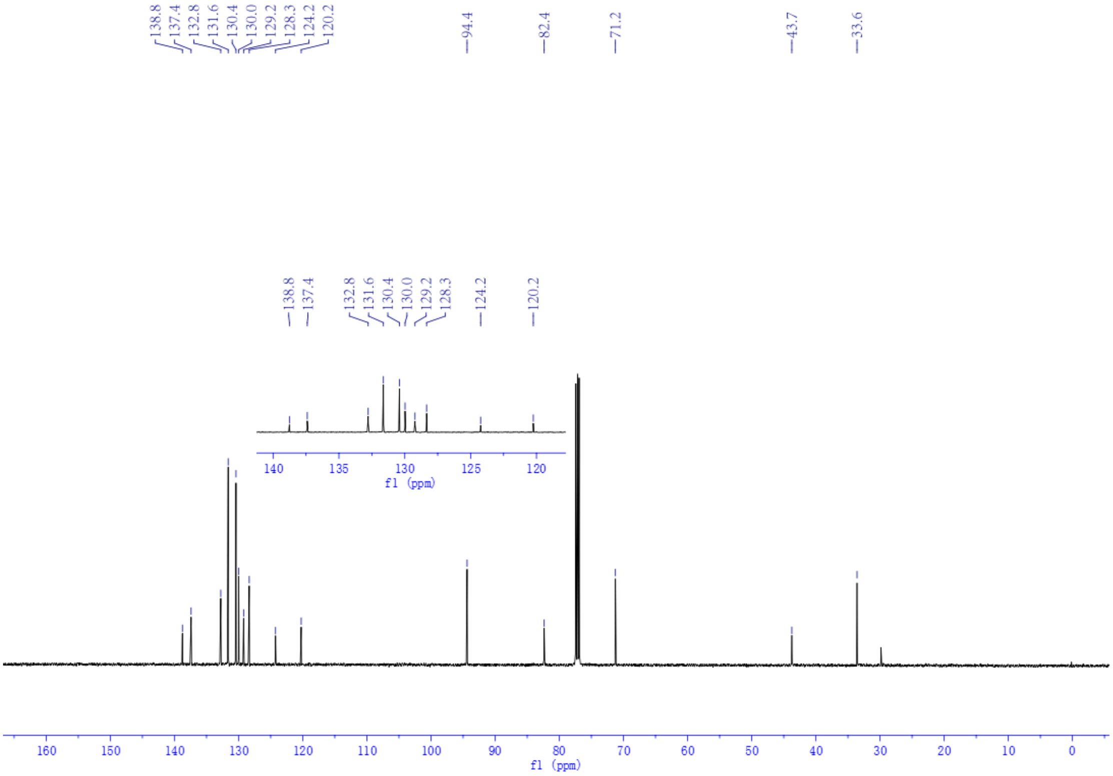

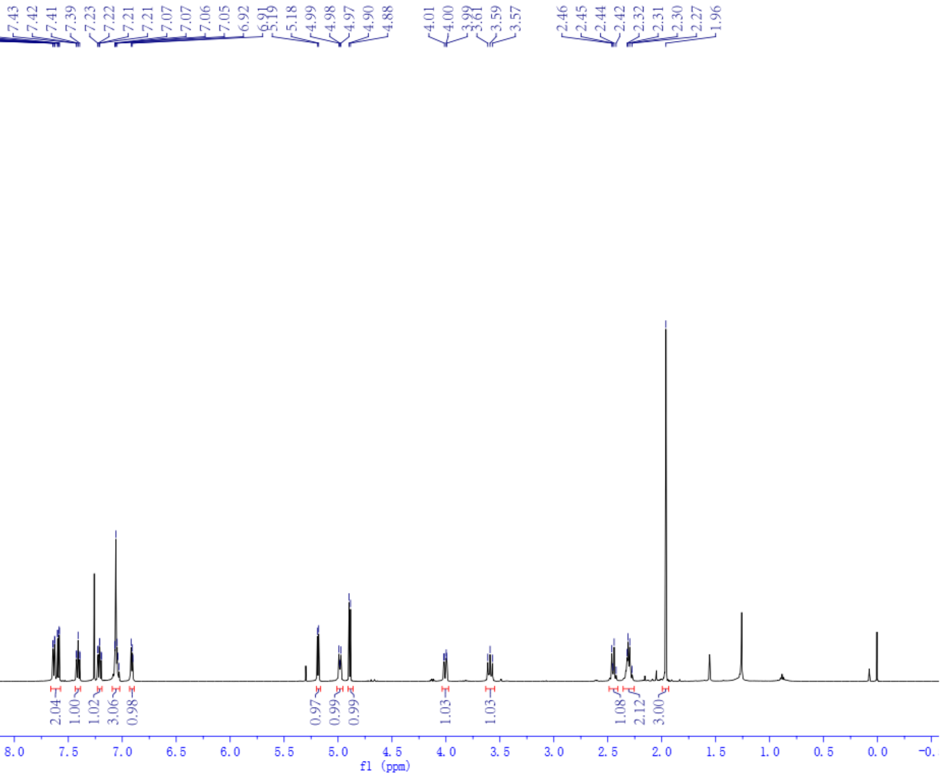
7.64 7.64 7.63 7.62 7.60 7.60 7.58 7.58 7.43 7.42 7.41 7.39 7.23 7.22 7.21 7.21 7.07 7.07 7.06 7.05 6.92 6.91 5.19 5.18 4.99 4.98 4.97 4.90 4.88 4.01 4.00 3.99 3.61 3.59 3.57 2.46 2.45 2.44 2.42 2.32 2.31 2.30 2.27 1.96
2.04 1.00 1.02 3.06 0.98 0.97 0.99 0.99 1.03 1.03 1.08 2.12 3.00
f1 (ppm)

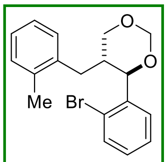
Me
Br
O
O

7f

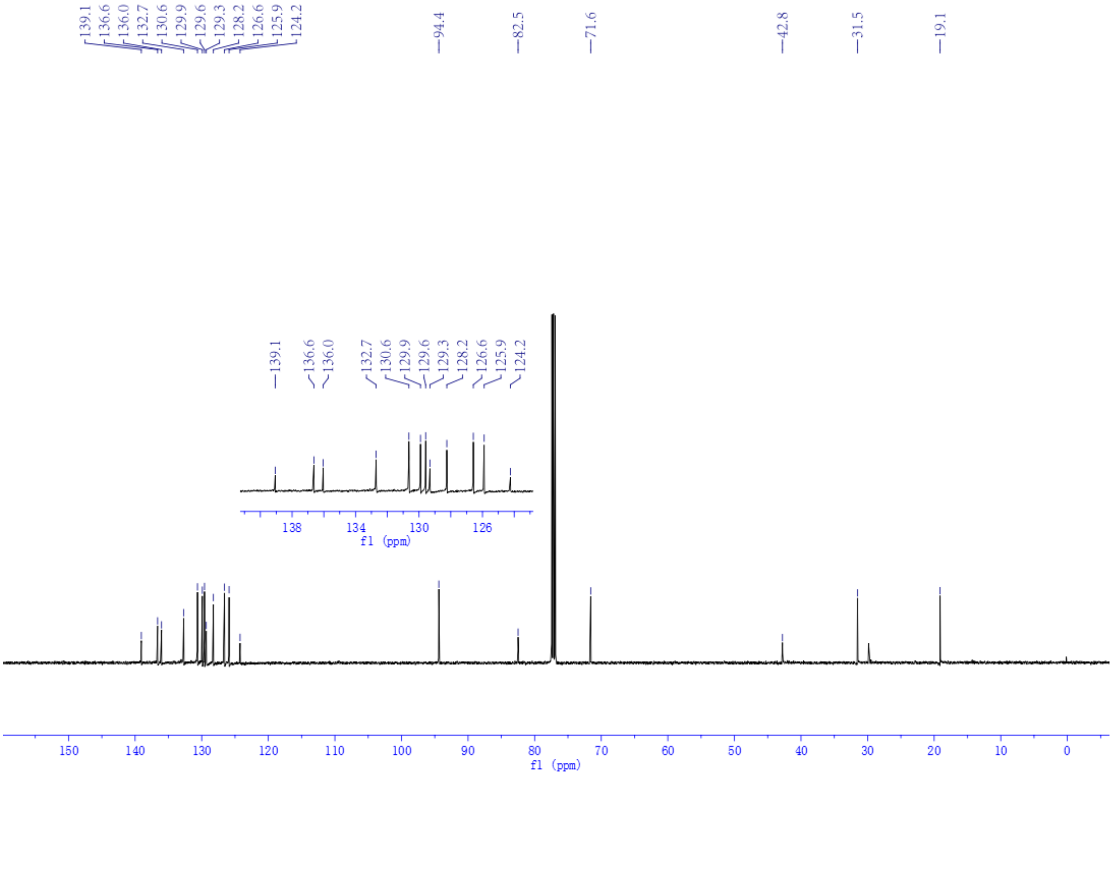
139.1 136.6 136.0 132.7 130.6 129.9 129.6 129.3 128.2 126.6 125.9 124.2 94.4 82.5 71.6 42.8 31.5 19.1
f1 (ppm)

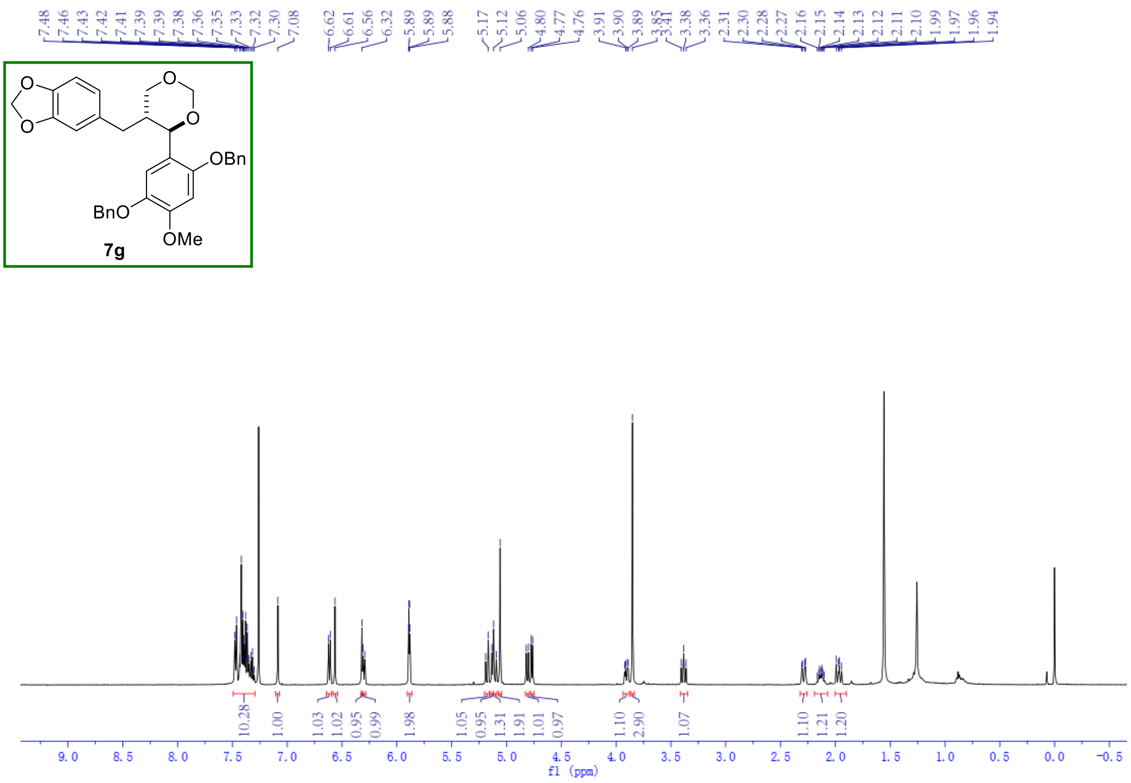
7g

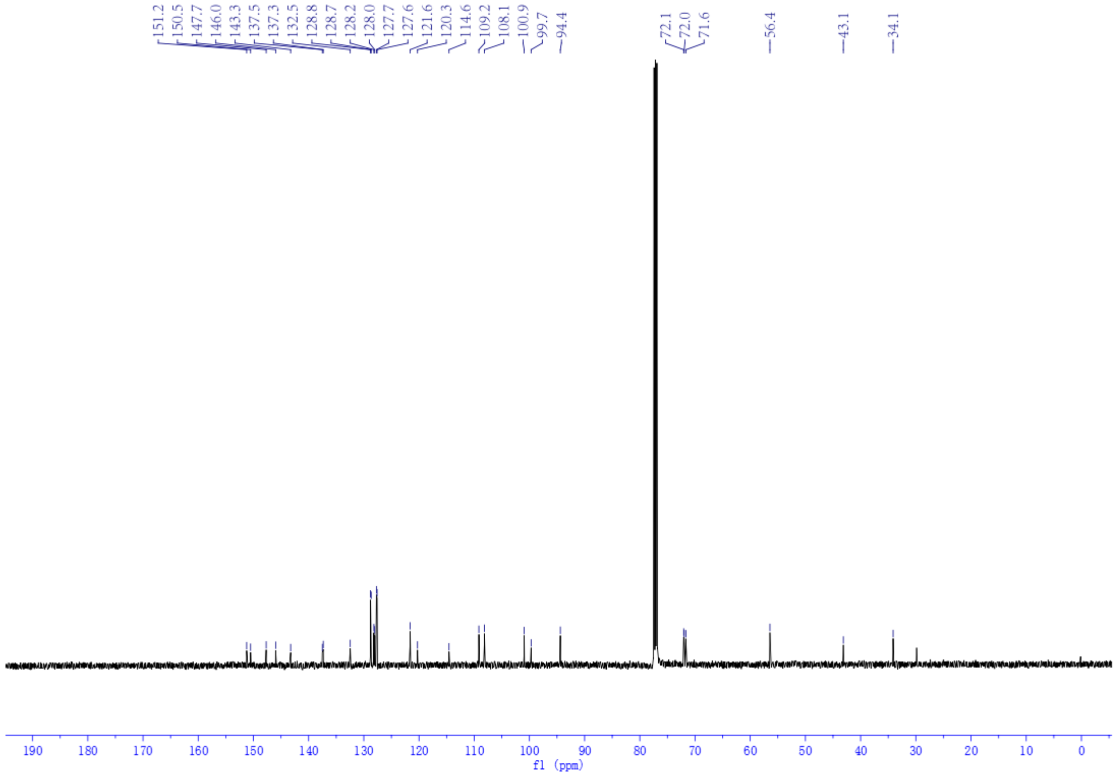

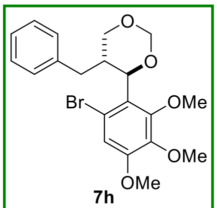
Br
OMe
OMe
OMe
O
O

7h

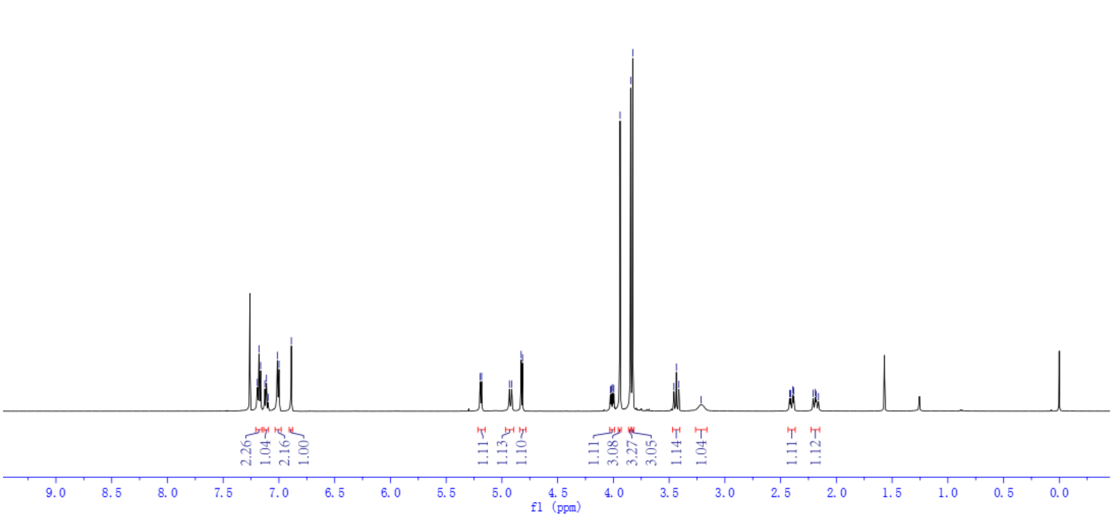
7.19 7.18 7.16 7.13 7.11 7.10 7.01 7.00 6.89
5.19 5.18 4.93 4.91 4.83 4.81
4.00 3.94 3.85 3.83 3.46 3.44 3.41 3.21
2.42 2.41 2.39 2.38 2.21 2.19 2.18 2.16
2.26 1.04 2.16 1.00 1.11 1.13 1.10 1.11 3.08 3.27 3.05 1.14 1.04 1.11 1.12
f1 (ppm)

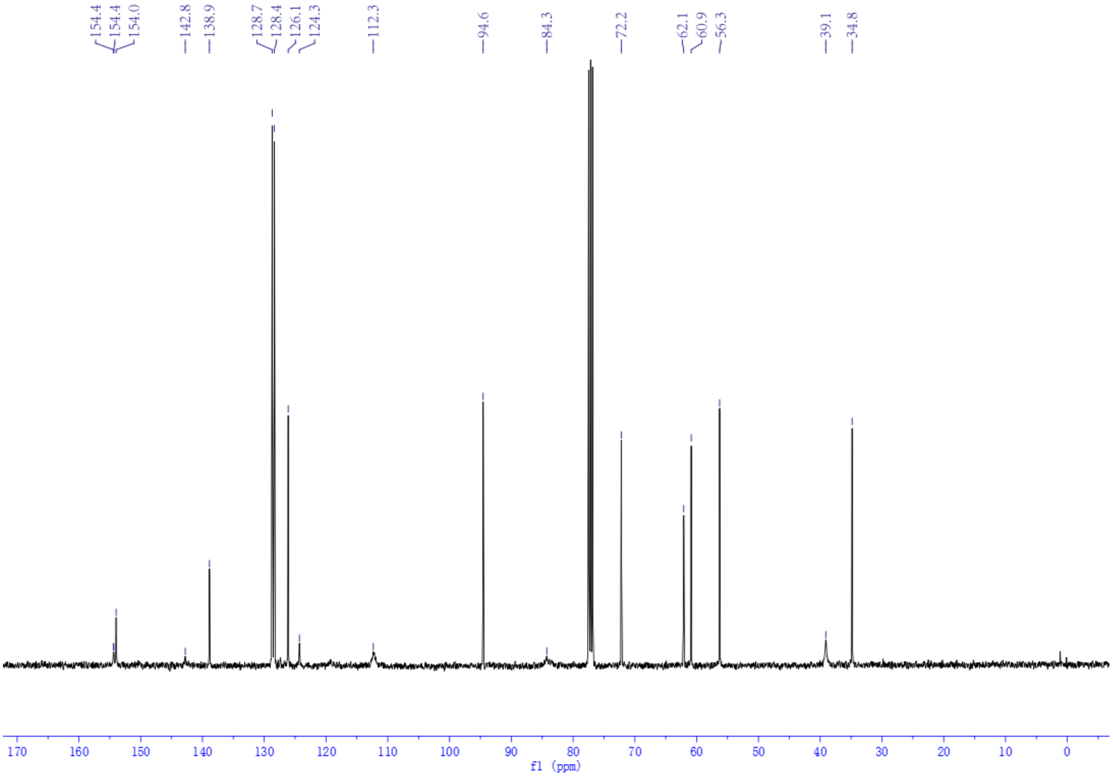
154.4 154.4 154.0 142.8 138.9 128.7 128.4 126.1 124.3 112.3 94.6 84.3 72.2 62.1 60.9 56.3 39.1 34.8
f1 (ppm)

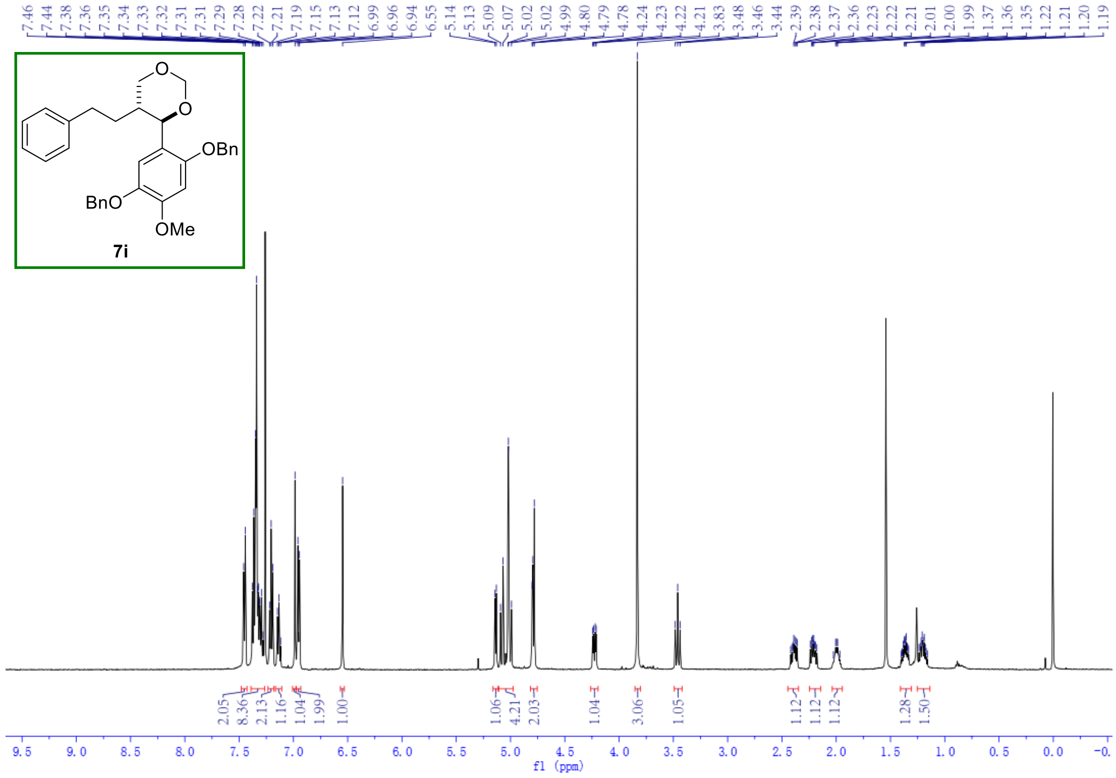

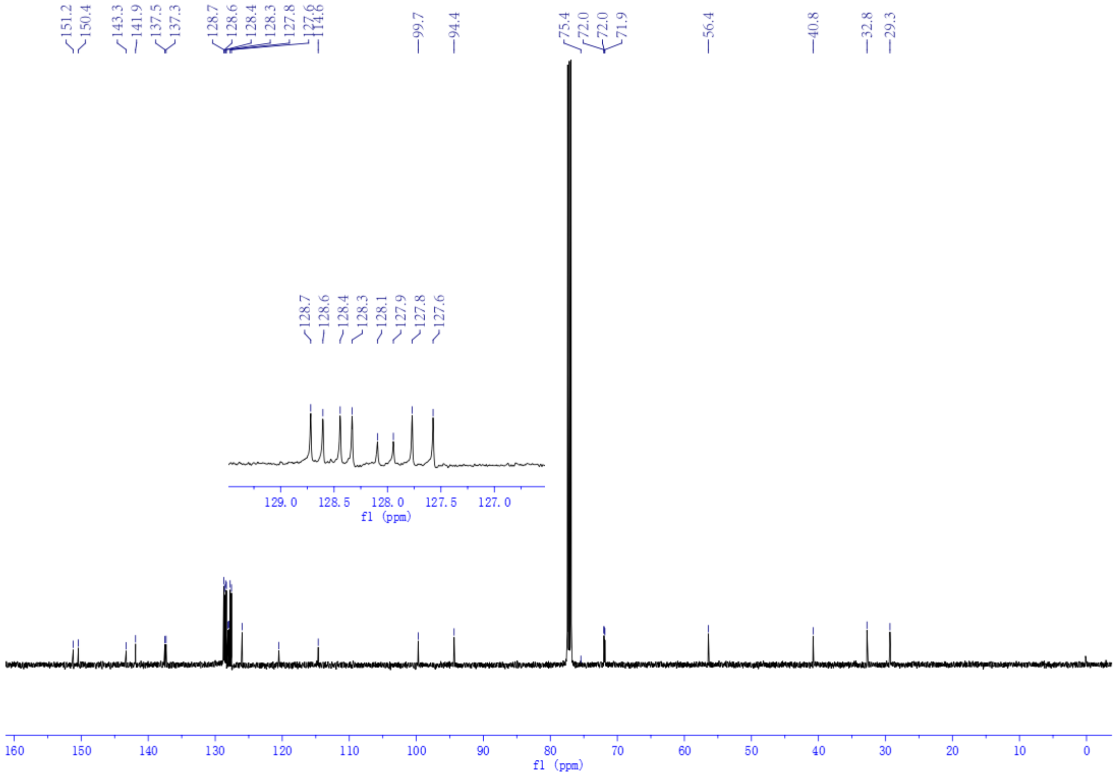

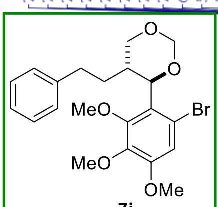

7j

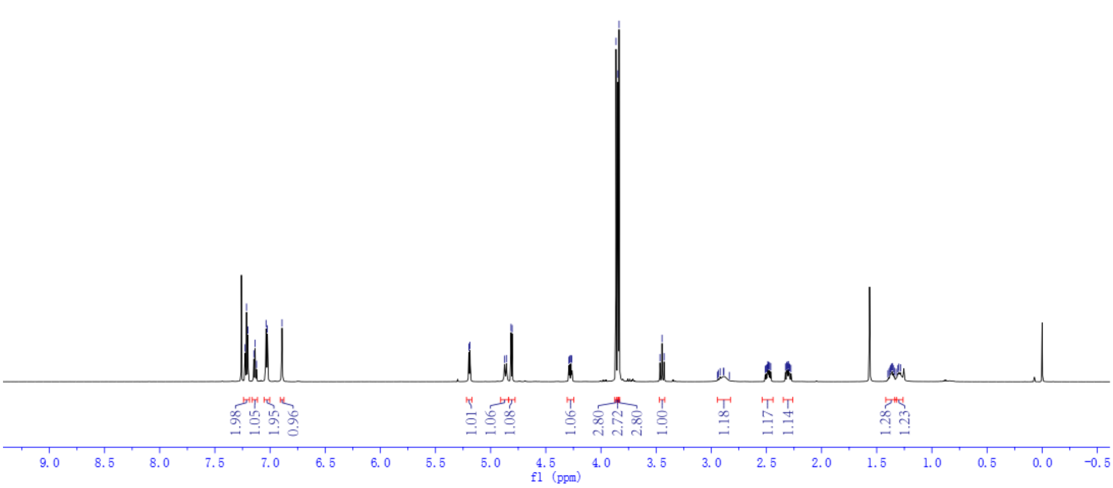

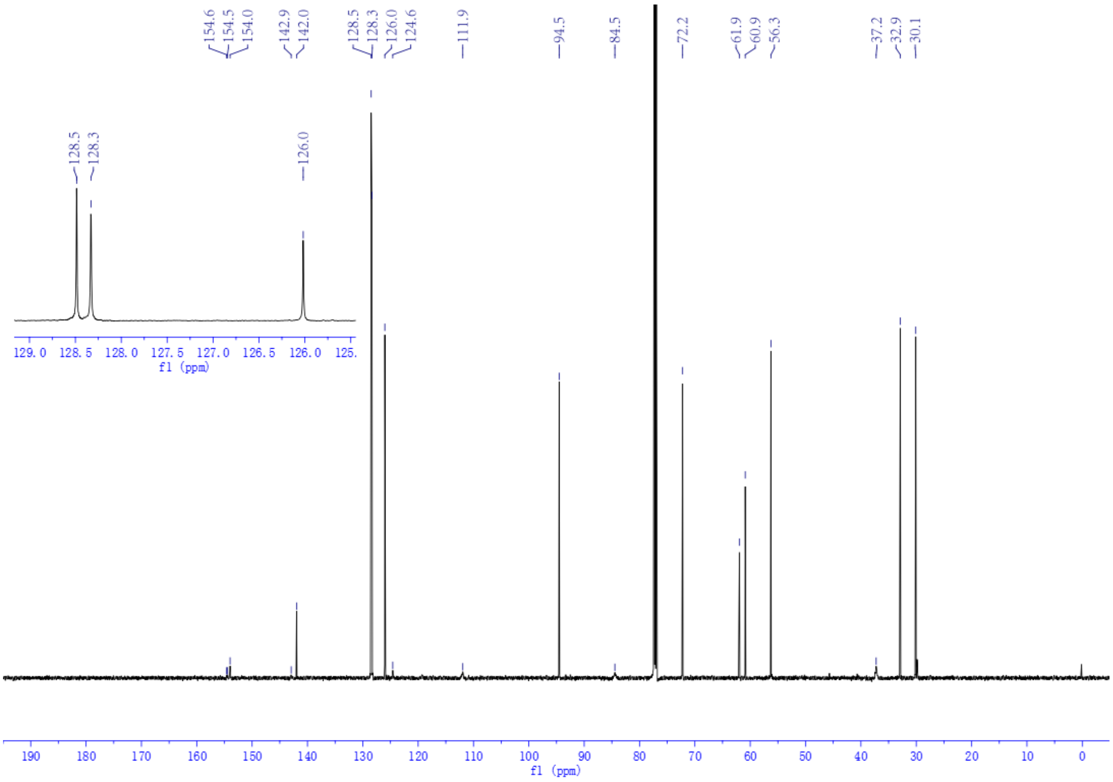

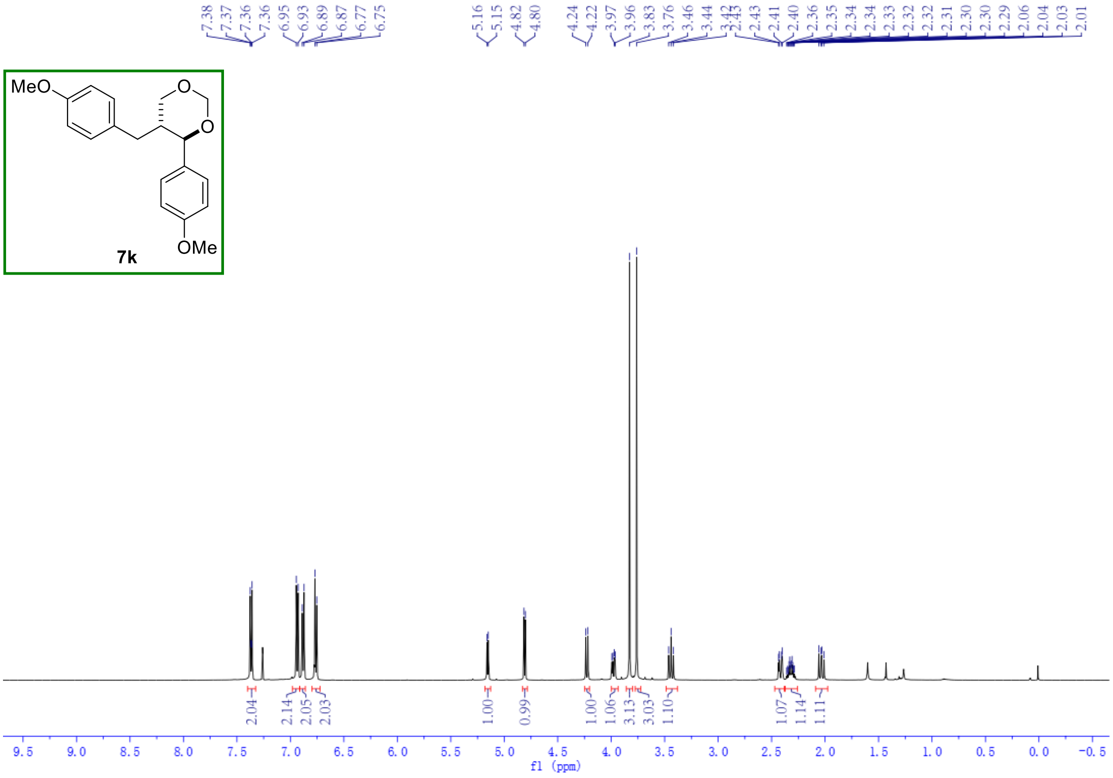
MeO
O
O
OMe
7k
7.38
7.37
7.36
7.36
6.95
6.93
6.89
6.87
6.77
6.75
5.16
5.15
4.82
4.80
4.24
4.22
3.97
3.96
3.83
3.76
3.46
3.44
2.04
2.14
2.05
2.03
1.00
0.99
1.00
1.06
3.13
3.03
1.10
1.07
1.14
1.11
f1 (ppm)

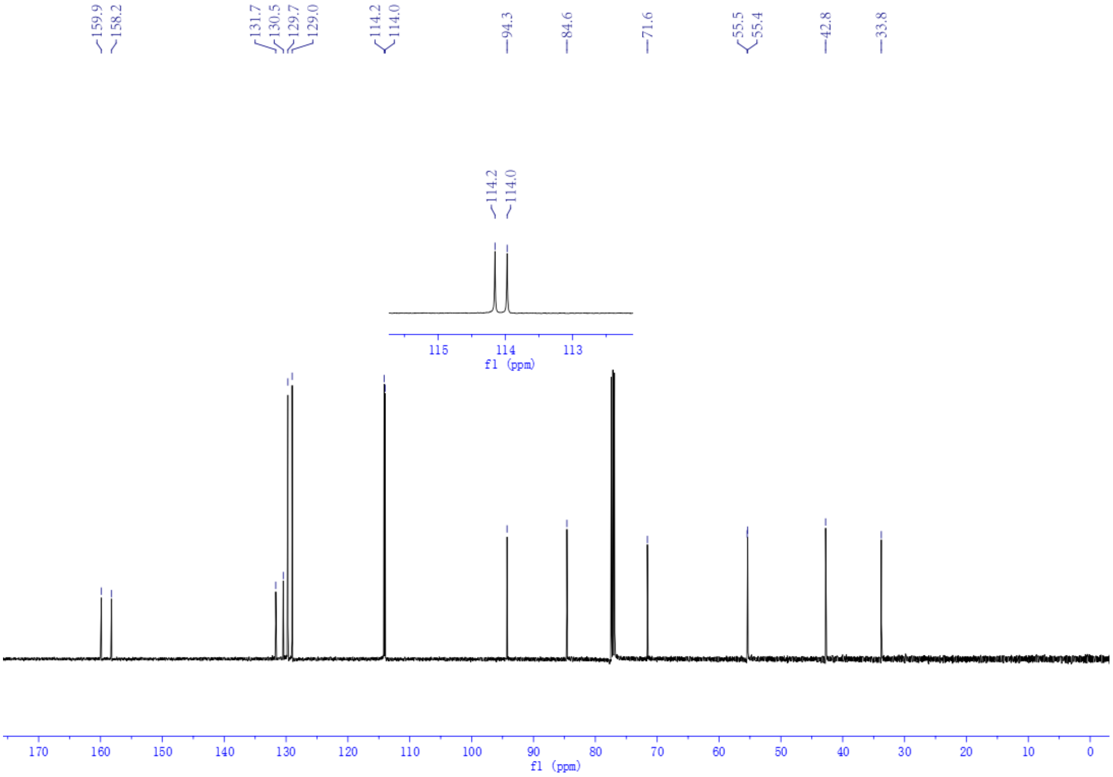
159.9
158.2
131.7
130.5
129.7
129.0
114.2
114.0
94.3
84.6
71.6
55.5
55.4
42.8
33.8
f1 (ppm)

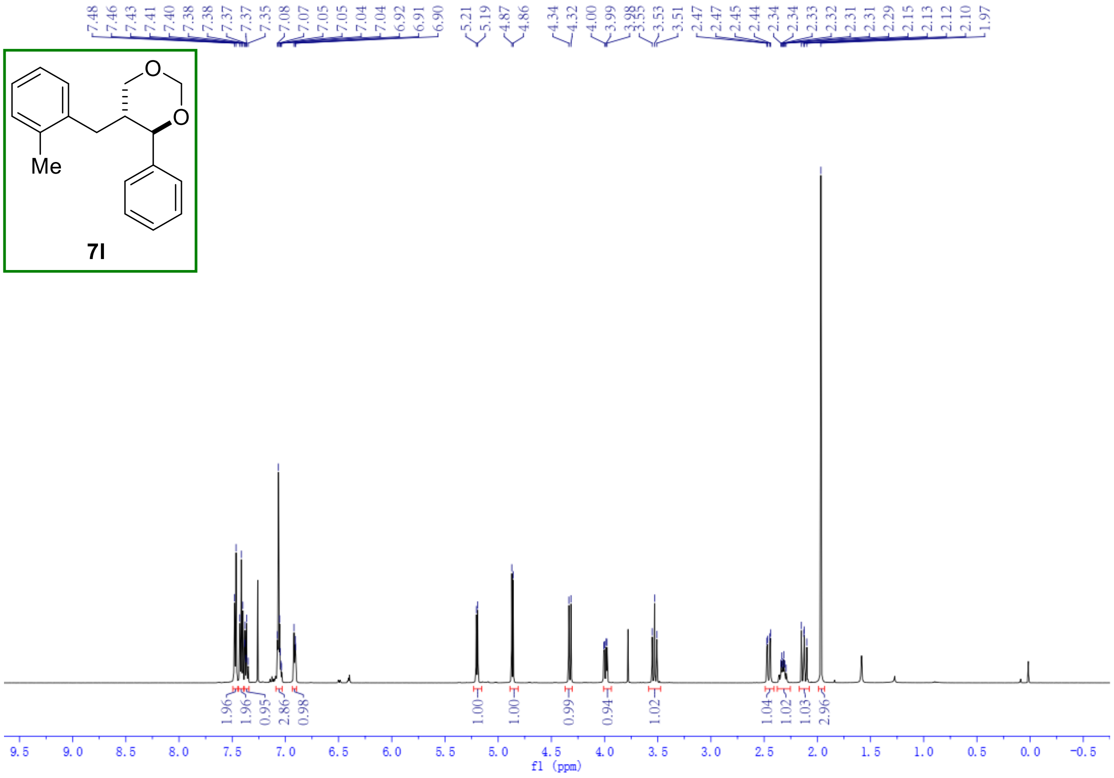

7.48 7.46 7.43 7.41 7.40 7.38 7.38 7.37 7.37 7.35 7.08 7.07 7.05 7.05 7.04 7.04 6.92 6.91 6.90
5.21 5.19 4.87 4.86 4.34 4.32 4.00 3.99 3.98 3.55 3.53 3.51
2.47 2.47 2.45 2.44 2.34 2.34 2.33 2.32 2.31 2.31 2.29 2.15 2.13 2.12 2.10 1.97
O
O
Me
7l
1.96 1.96 0.95 2.86 0.98 1.00 1.00 0.99 0.94 1.02 1.04 1.02 1.03 2.96
f1 (ppm)

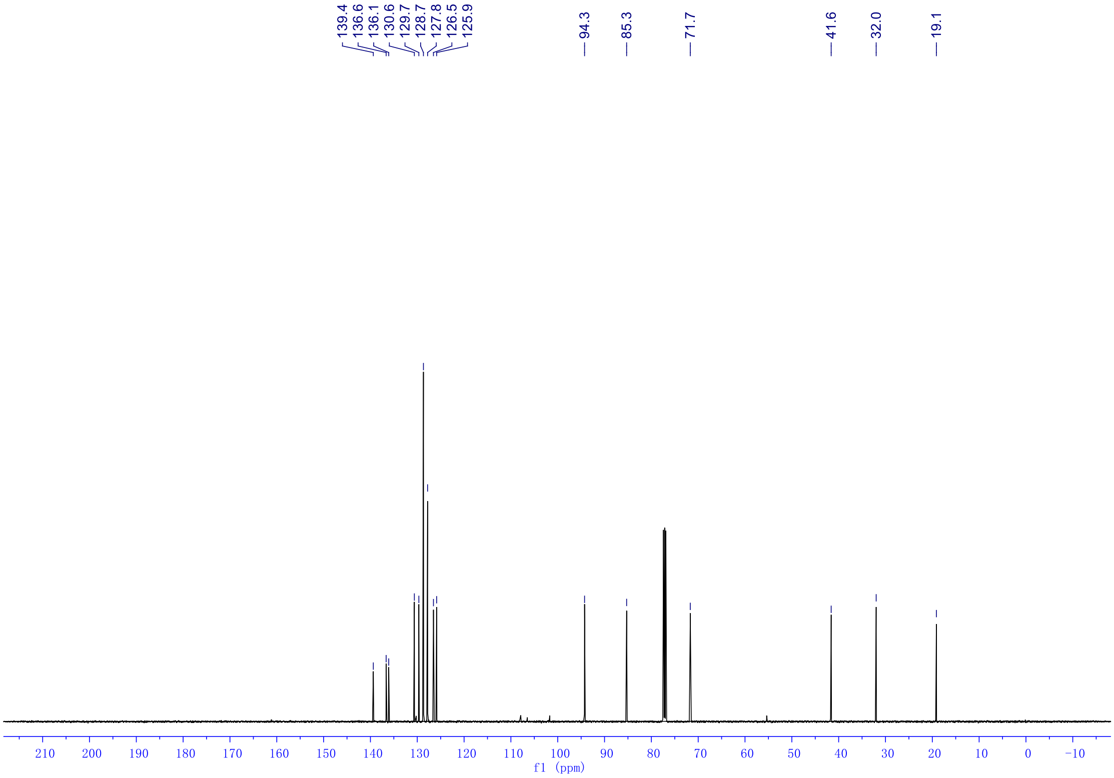

139.4 136.6 136.1 130.6 129.7 128.7 127.8 126.5 125.9
94.3
85.3
71.7
41.6
32.0
19.1
f1 (ppm)

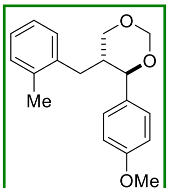

O
O
Me
OMe

7m

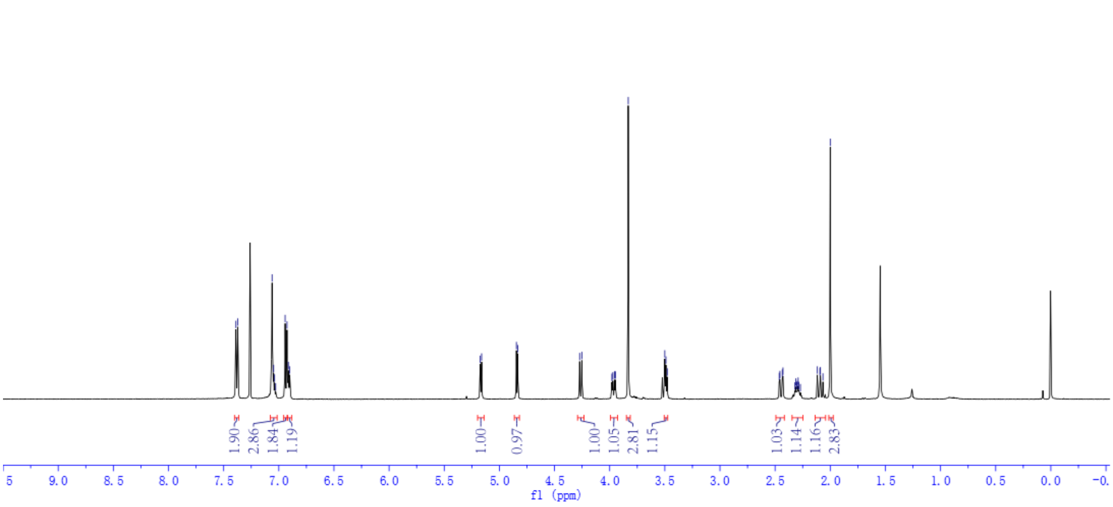

7.39
7.37
7.06
7.05
7.04
7.03
6.94
6.93
6.91
6.90
5.17
5.16
4.85
4.83
4.27
4.25
3.97
3.96
3.95
3.83
3.50
3.49
3.48
2.46
2.43
2.43
2.32
2.31
2.30
2.29
2.28
2.27
2.12
2.09
2.09
2.07
2.00
1.90
2.86
1.84
1.19
1.00
0.97
1.00
1.05
2.81
1.15
1.03
1.14
1.16
2.83
f1 (ppm)

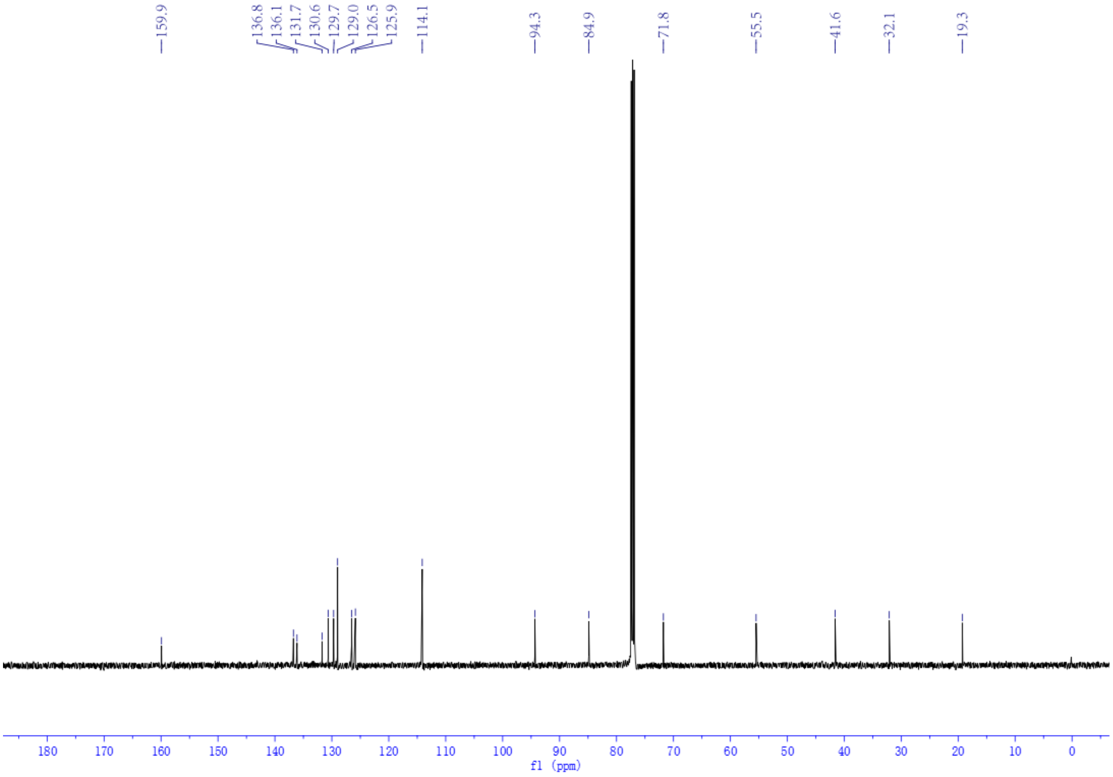

159.9
136.8
136.1
131.7
130.6
129.7
129.0
126.5
125.9
114.1
94.3
84.9
71.8
55.5
41.6
32.1
19.3
f1 (ppm)

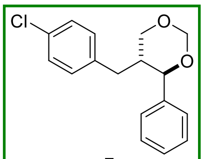
Cl
O
O

7n

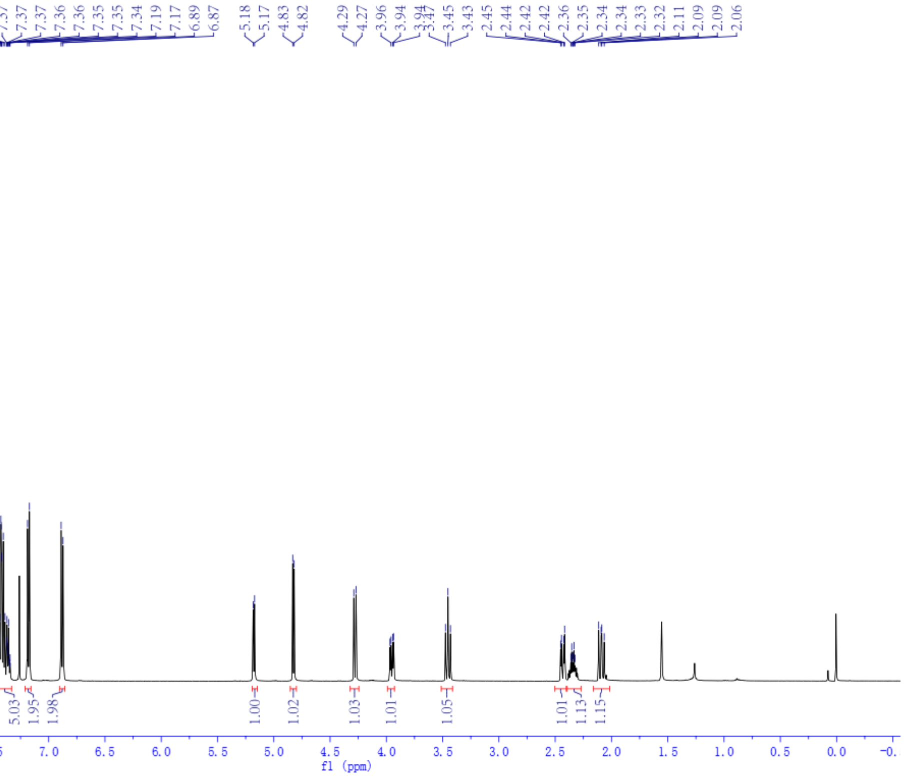
7.44
7.44
7.42
7.42
7.42
7.42
7.40
7.39
7.37
7.37
7.37
7.36
7.36
7.35
7.35
7.34
7.19
7.17
6.89
6.87
5.18
5.17
4.83
4.82
4.29
4.27
3.96
3.94
3.94
3.47
3.45
3.43
2.45
2.44
2.42
2.42
2.36
2.35
2.34
2.34
2.33
2.32
2.11
2.09
2.09
2.06
5.03
1.95
1.98
1.00
1.02
1.03
1.01
1.05
1.01
1.13
1.15
9.0
8.5
8.0
7.5
7.0
6.5
6.0
5.5
5.0
4.5
4.0
3.5
3.0
2.5
2.0
1.5
1.0
0.5
0.0
f1 (ppm)

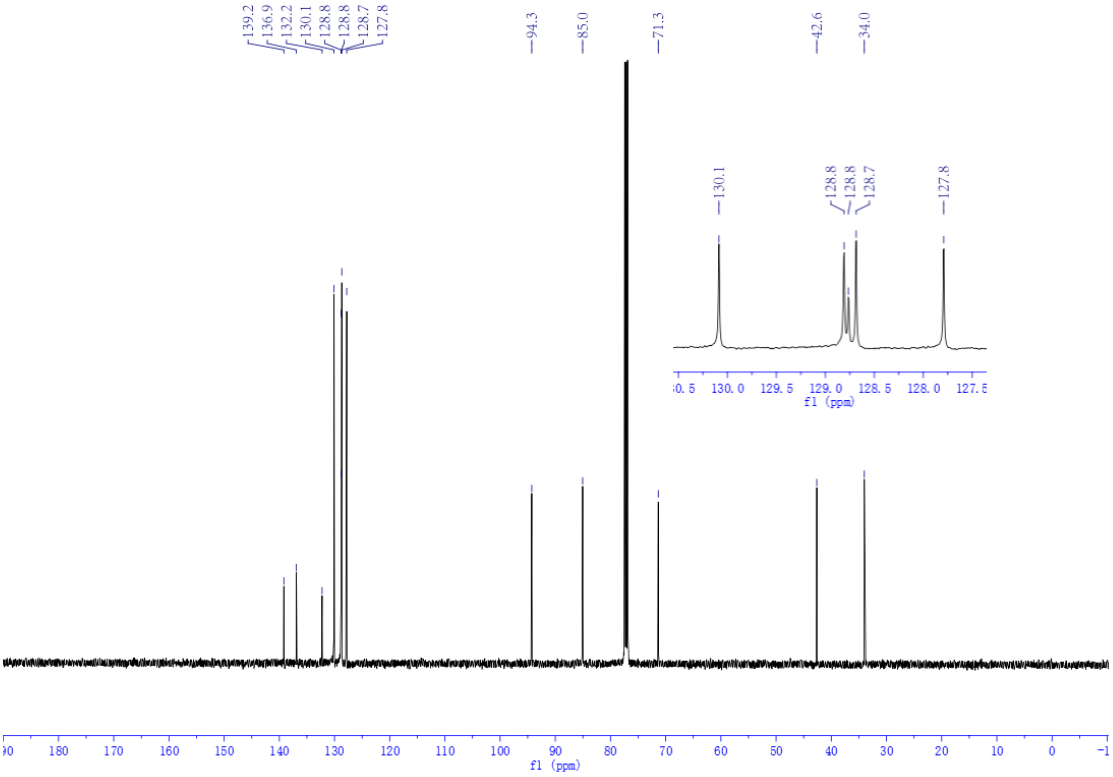
139.2
136.9
132.2
130.1
128.8
128.8
128.7
127.8
94.3
85.0
71.3
42.6
34.0
130.1
128.8
128.8
128.7
127.8
130.0
129.5
129.0
128.5
128.0
f1 (ppm)
180
170
160
150
140
130
120
110
100
90
80
70
60
50
40
30
20
10
0
f1 (ppm)

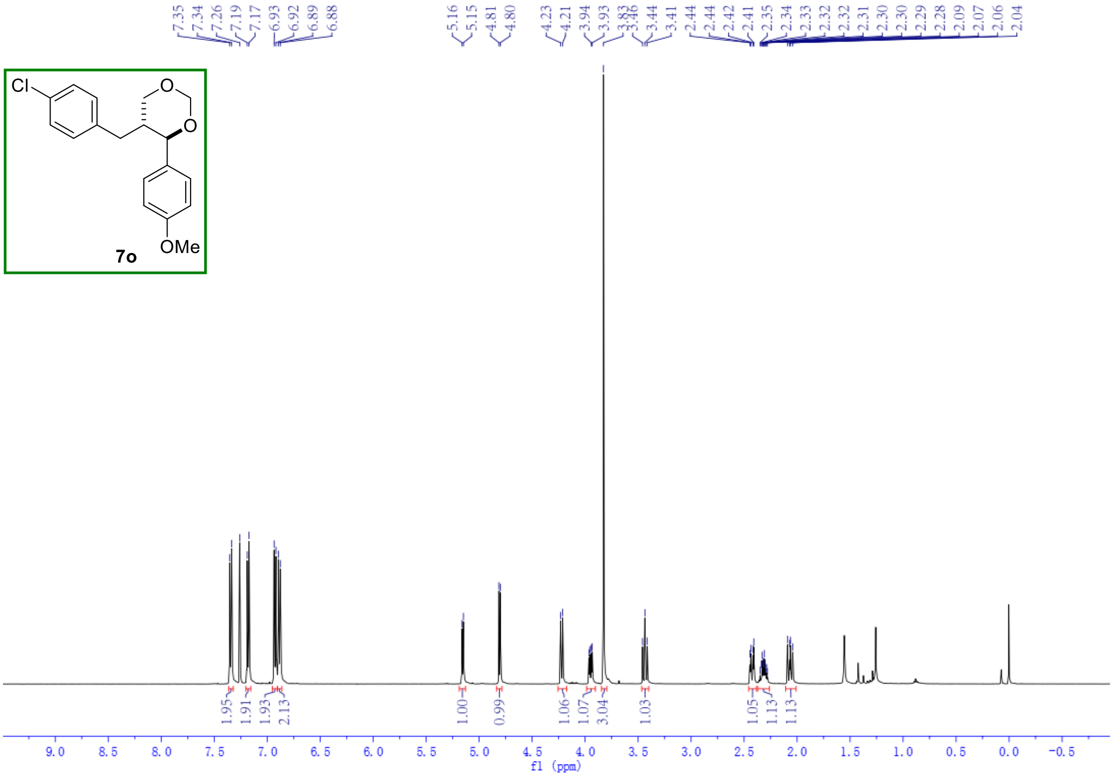
7.35 7.34 7.26 7.19 7.17 6.93 6.92 6.89 6.88
5.16 5.15 4.81 4.80 4.23 4.21 3.94 3.93 3.83 3.46 3.44 3.41
2.44 2.44 2.42 2.41 2.35 2.34 2.33 2.32 2.32 2.31 2.30 2.30 2.29 2.28 2.09 2.07 2.06 2.04
Cl
O
O
OMe
7o
1.95 1.91 1.93 2.13 1.00 0.99 1.06 1.07 3.04 1.03 1.05 1.13 1.13
9.0 8.5 8.0 7.5 7.0 6.5 6.0 5.5 5.0 4.5 4.0 3.5 3.0 2.5 2.0 1.5 1.0 0.5 0.0 -0.5
f1 (ppm)

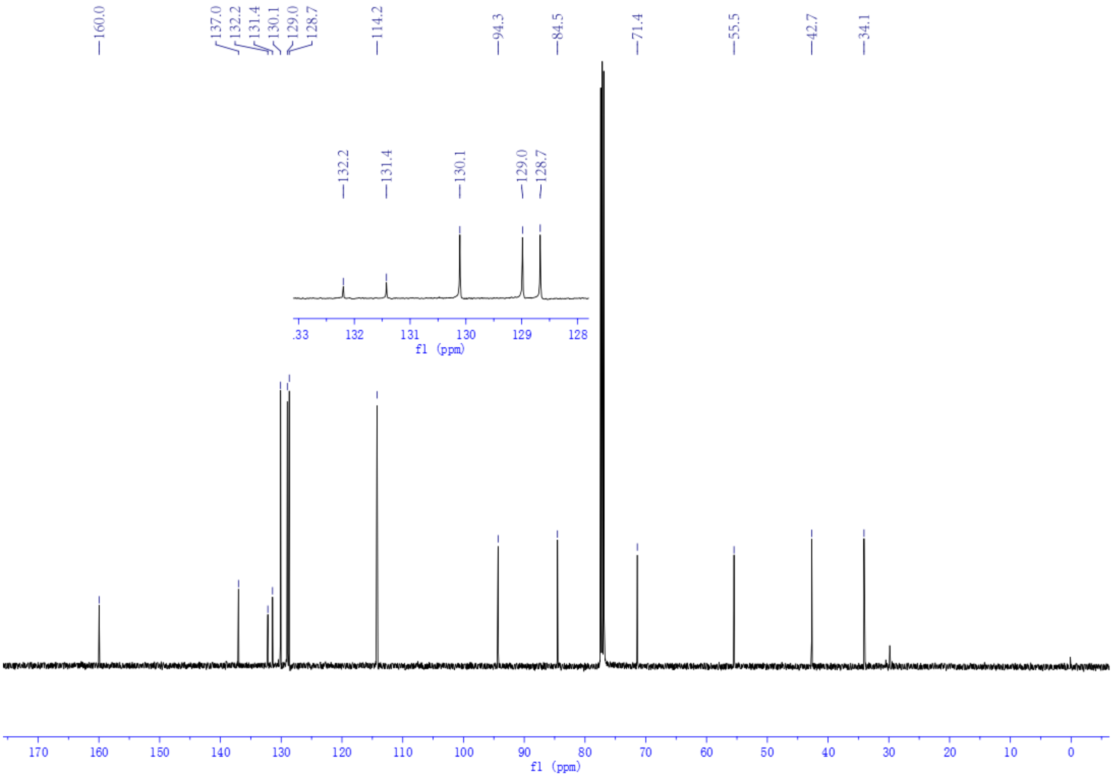
160.0 137.0 132.2 131.4 130.1 129.0 128.7 114.2 94.3 84.5 71.4 55.5 42.7 34.1
132.2 131.4 130.1 129.0 128.7
.33 132 131 130 129 128
f1 (ppm)
170 160 150 140 130 120 110 100 90 80 70 60 50 40 30 20 10 0
f1 (ppm)

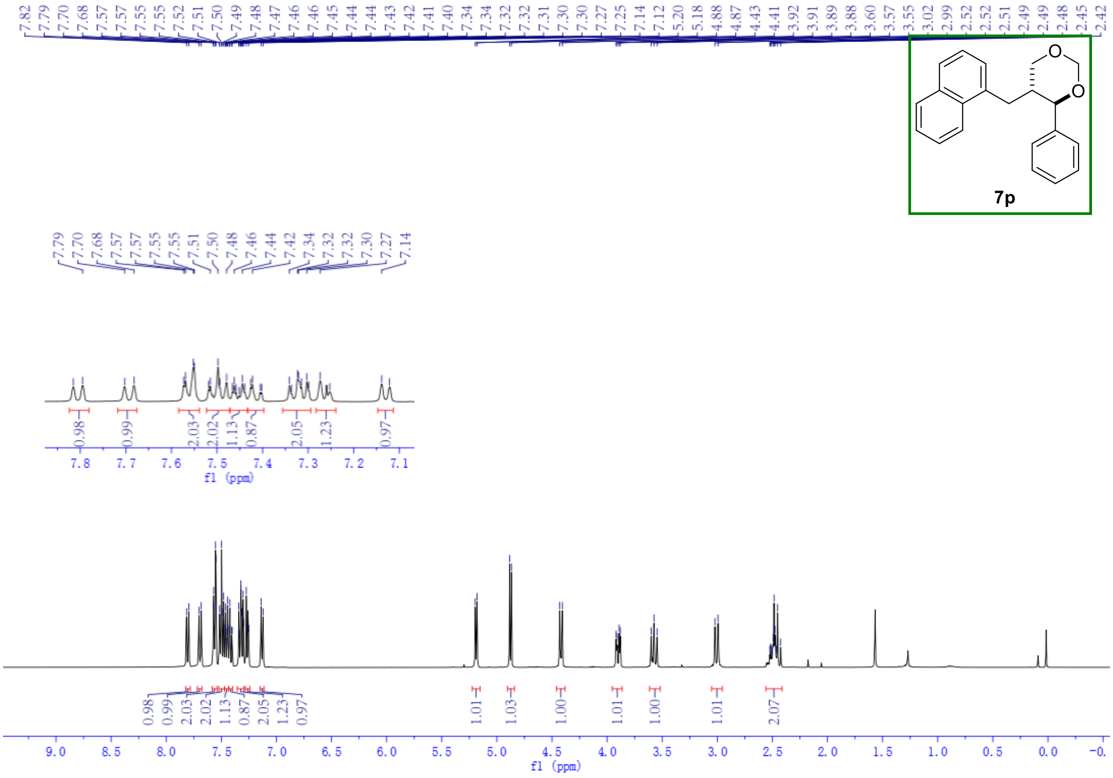
7.82 7.79 7.70 7.68 7.57 7.57 7.55 7.55 7.52 7.51 7.50 7.49 7.48 7.47 7.46 7.46 7.45 7.44 7.44 7.43 7.42 7.41 7.40 7.34 7.34 7.32 7.32 7.31 7.30 7.30 7.27 7.25 7.14 7.12 5.20 5.18 4.88 4.87 4.43 4.41 3.92 3.91 3.89 3.88 3.60 3.57 3.55 3.02 2.99 2.52 2.52 2.51 2.49 2.49 2.48 2.45 2.42
O
O
7p
7.79 7.70 7.68 7.57 7.57 7.55 7.55 7.51 7.50 7.48 7.46 7.44 7.42 7.34 7.32 7.32 7.30 7.27 7.14
0.98 0.99 2.03 2.02 1.13 0.87 2.05 1.23 0.97
7.8 7.7 7.6 7.5 7.4 7.3 7.2 7.1
f1 (ppm)
0.98 0.99 2.03 2.02 1.13 0.87 2.05 1.23 0.97 1.01 1.03 1.00 1.01 1.00 1.01 2.07
9.0 8.5 8.0 7.5 7.0 6.5 6.0 5.5 5.0 4.5 4.0 3.5 3.0 2.5 2.0 1.5 1.0 0.5 0.0
f1 (ppm)

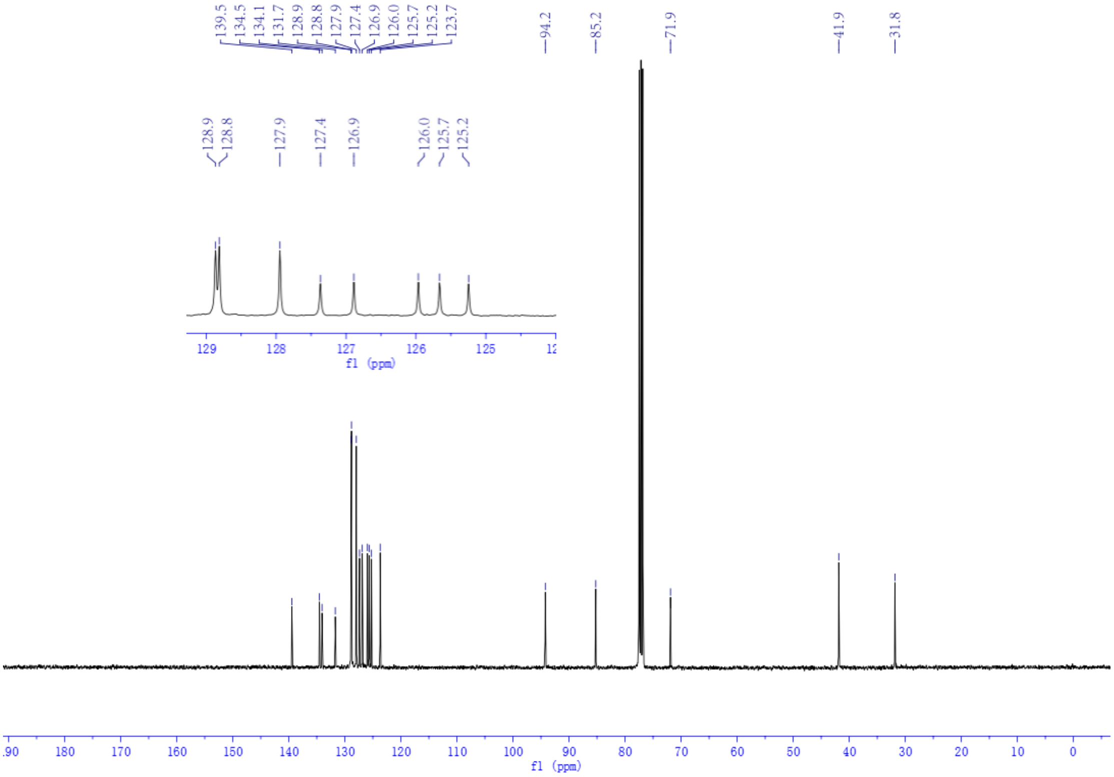
139.5 134.5 134.1 131.7 128.9 128.8 127.9 127.4 126.9 126.0 125.7 125.2 123.7
94.2
85.2
71.9
41.9
31.8
128.9 128.8 127.9 127.4 126.9 126.0 125.7 125.2
129 128 127 126 125
f1 (ppm)
180 170 160 150 140 130 120 110 100 90 80 70 60 50 40 30 20 10 0
f1 (ppm)

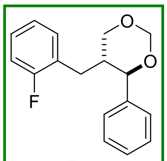

7q

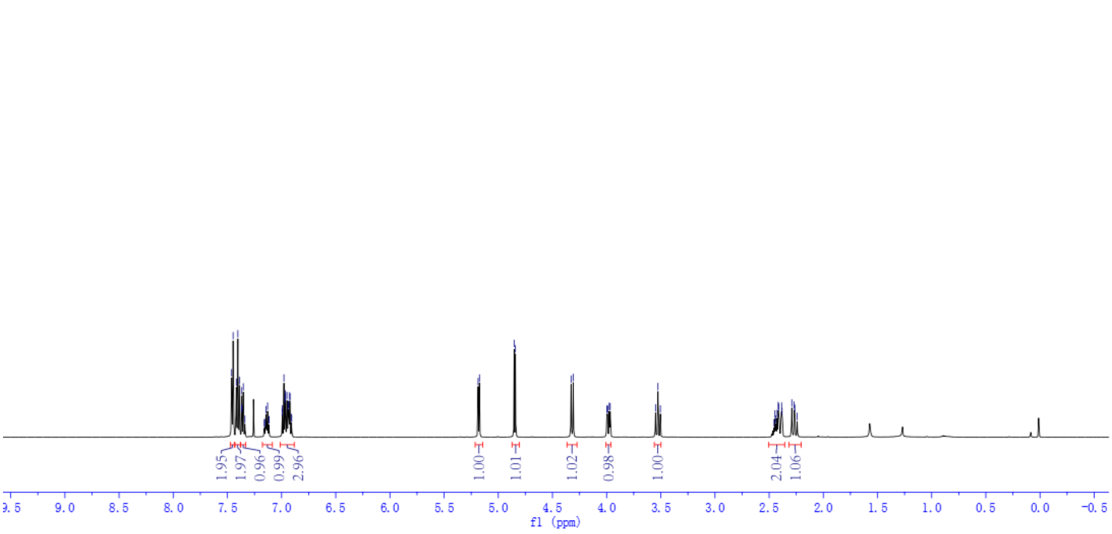

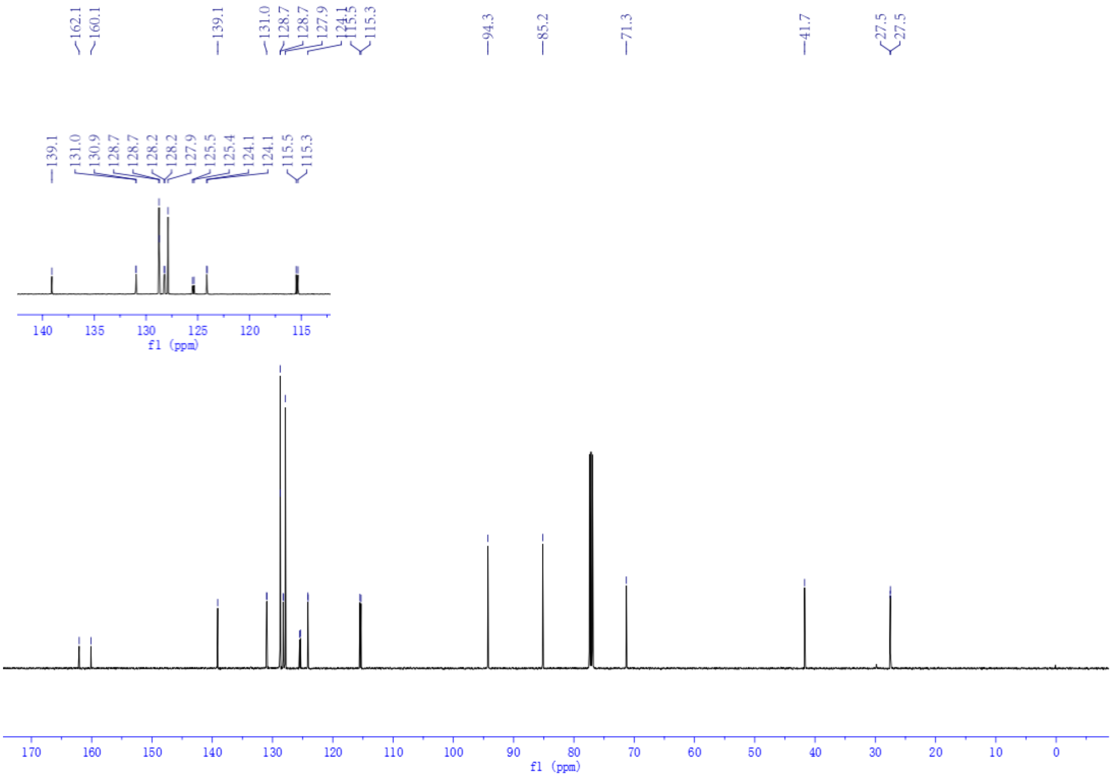

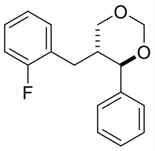

7q

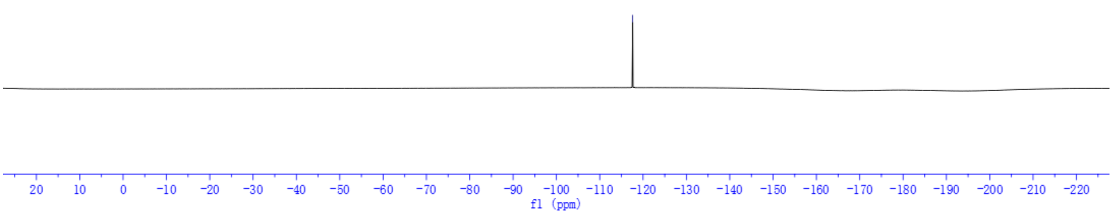

-117.6
20
10
0
-10
-20
-30
-40
-50
-60
-70
-80
-90
-100
-110
-120
-130
-140
-150
-160
-170
-180
-190
-200
-210
-220
f1 (ppm)

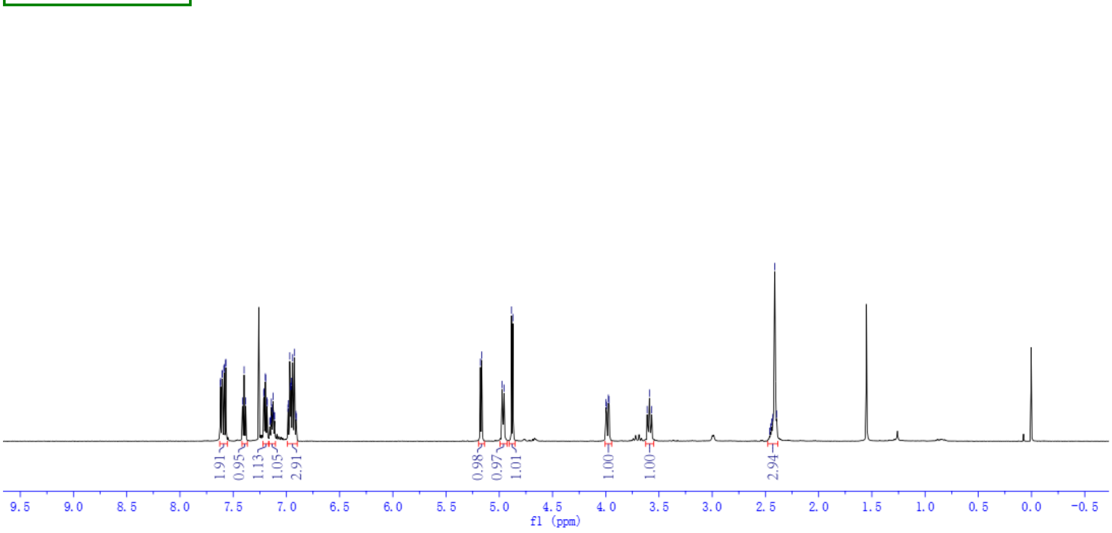
7.62 7.62 7.60 7.60 7.59 7.59 7.57 7.57 7.41 7.40 7.38 7.21 7.21 7.20 7.19 7.13 6.97 6.96 6.96 6.95 6.94 6.93 5.18 5.17 4.97 4.96 4.88 4.87 4.00 3.99 3.98 3.97 3.61 3.59 3.57 2.46 2.45 2.44 2.43 2.41 2.40
1.91 0.95 1.13 1.05 2.91 0.98 0.97 1.01 1.00 1.00 2.94
f1 (ppm)

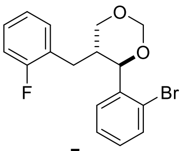
O
O
F
Br

7r

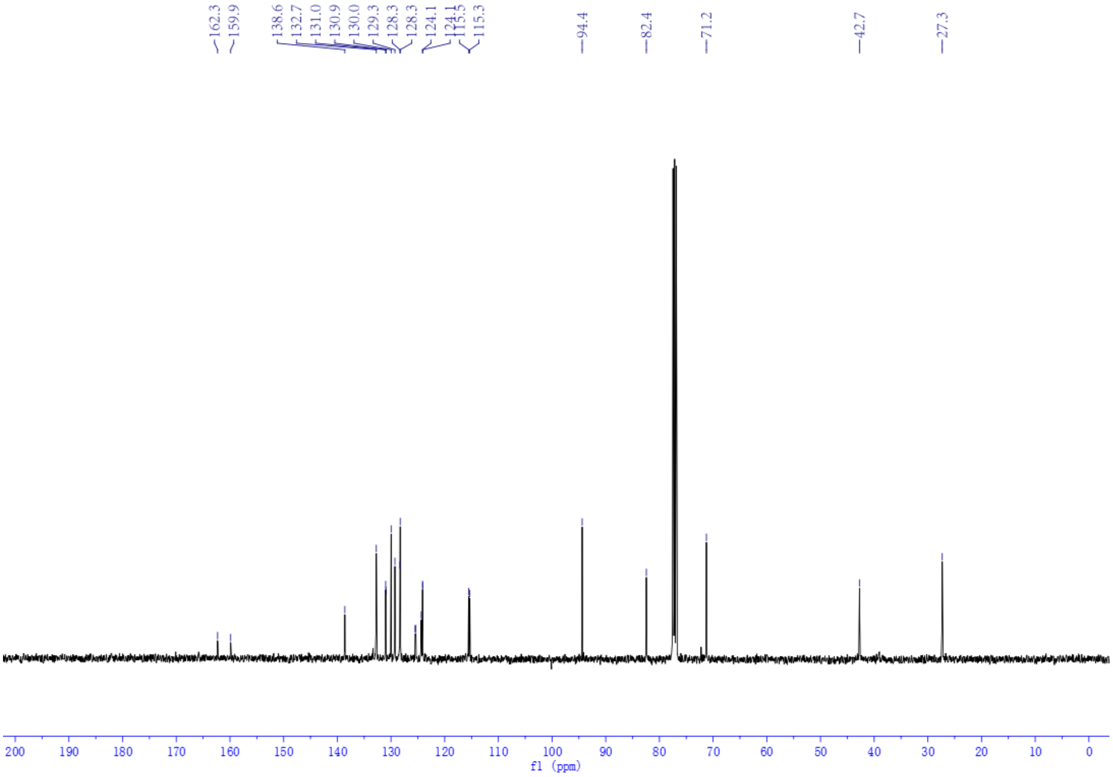
162.3 159.9 138.6 132.7 131.0 130.9 130.0 129.3 128.3 128.3 124.1 124.1 115.5 115.3 94.4 82.4 71.2 42.7 27.3
f1 (ppm)

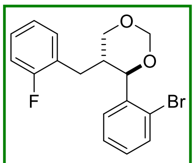

O
O
F
Br

7r

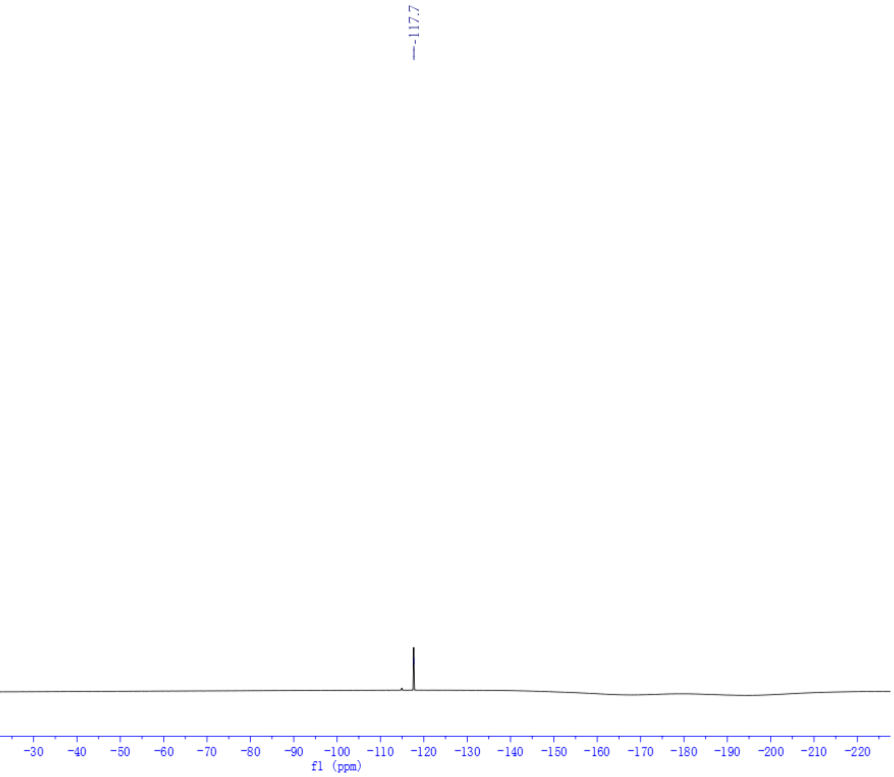

-117.7
20
10
0
-10
-20
-30
-40
-50
-60
-70
-80
-90
-100
-110
-120
-130
-140
-150
-160
-170
-180
-190
-200
-210
-220
f1 (ppm)

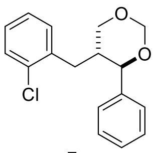
Cl
O
O

7s

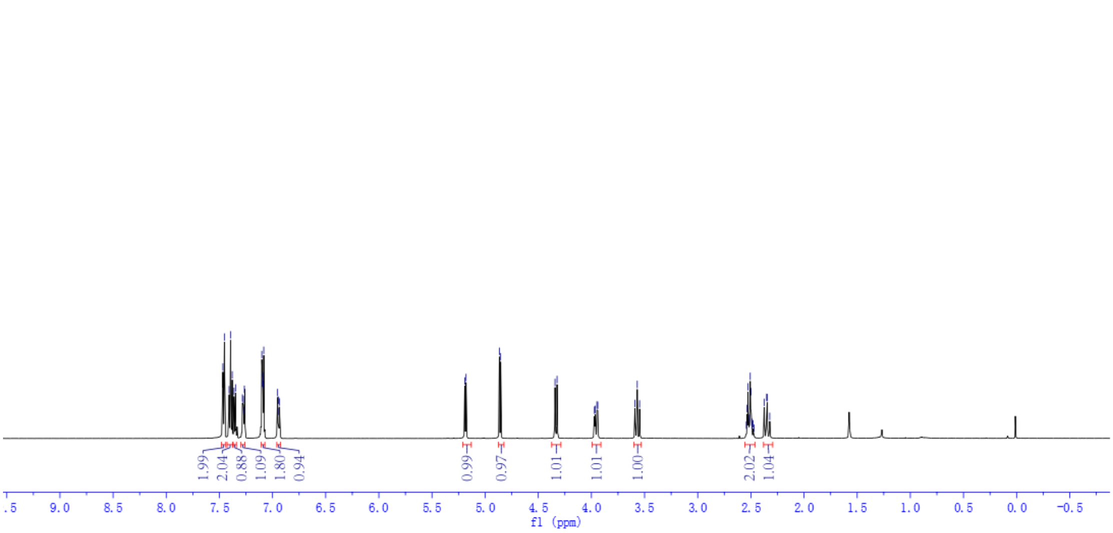
7.47 7.45 7.41 7.39 7.38 7.36 7.35 7.28 7.27 7.27 7.27 7.27 7.10 7.10 7.09 7.09 7.08 6.95 6.95 6.94 6.93
5.19 5.18 4.87 4.85
4.34 4.32 3.96 3.95 3.94 3.59 3.57 3.55 2.54 2.53 2.53 2.51 2.51 2.50 2.49 2.49 2.48 2.47 2.37 2.35 2.32
1.99 2.04 0.88 1.09 1.80 0.94 0.99 0.97 1.01 1.01 1.00 2.02 1.04
f1 (ppm)

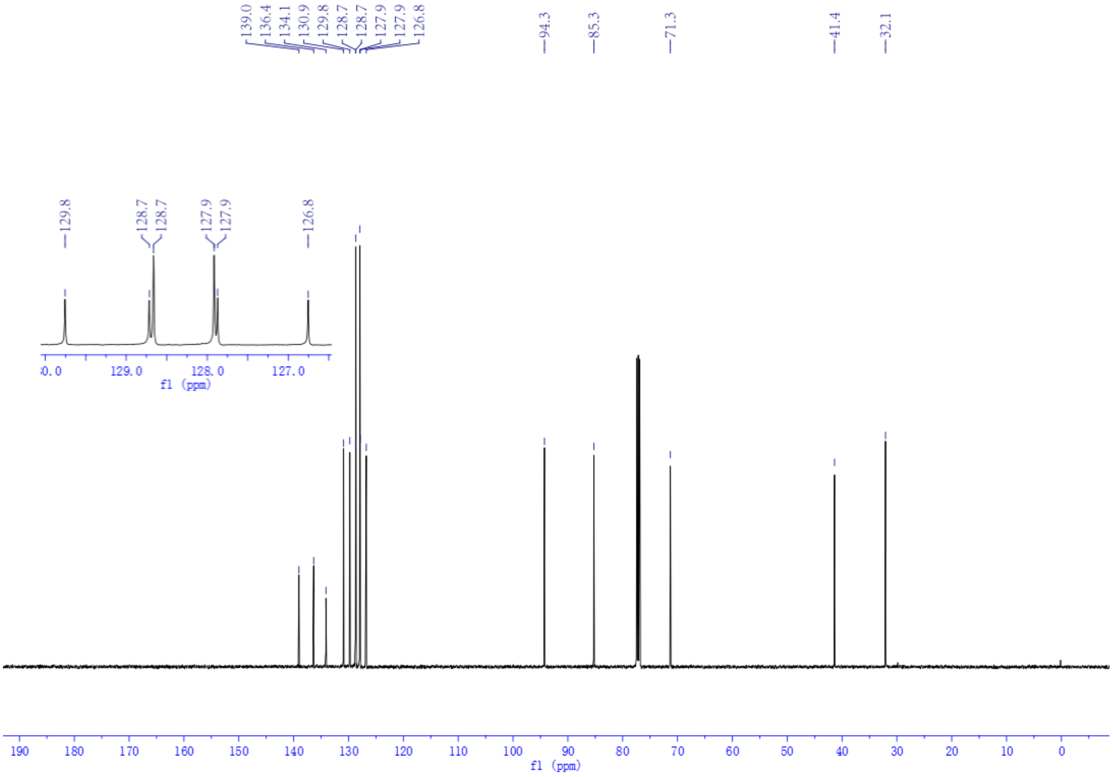
139.0 136.4 134.1 130.9 129.8 128.7 128.7 127.9 127.9 126.8
94.3
85.3
71.3
41.4
32.1
129.8 128.7 128.7 127.9 127.9 126.8
f1 (ppm)
f1 (ppm)

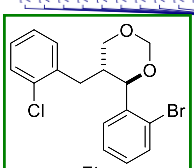
O
O
Cl
Br

7t

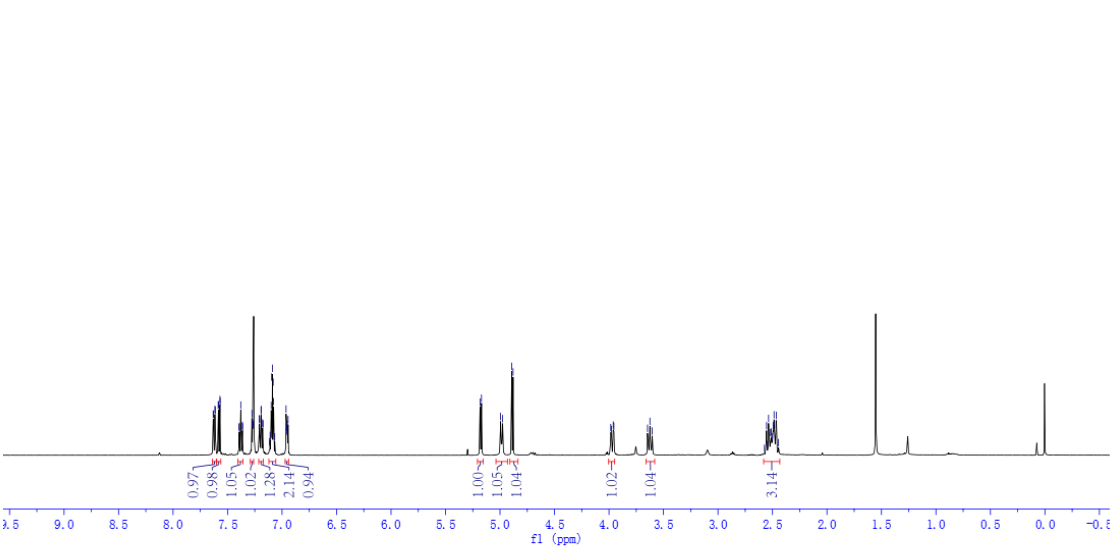
7.63 7.63 7.62 7.61 7.59 7.58 7.57 7.57 7.39 7.39 7.38 7.36 7.36 7.28 7.27 7.27 7.27 7.21 7.21 7.19 7.19 7.18 7.17 7.11 7.10 7.10 7.09 7.08 7.08 7.07 6.96 6.96 6.96 6.95 6.94 5.18 5.17 4.99 4.98 4.89 4.88 3.98 3.98 3.96 3.96 3.65 3.62 3.60 2.55 2.54 2.52 2.51 2.50 2.49 2.49 2.48 2.46
0.97 0.98 1.05 1.02 1.28 2.14 0.94 1.00 1.05 1.04 1.02 1.04 3.14
9.0 8.5 8.0 7.5 7.0 6.5 6.0 5.5 5.0 4.5 4.0 3.5 3.0 2.5 2.0 1.5 1.0 0.5 0.0
f1 (ppm)

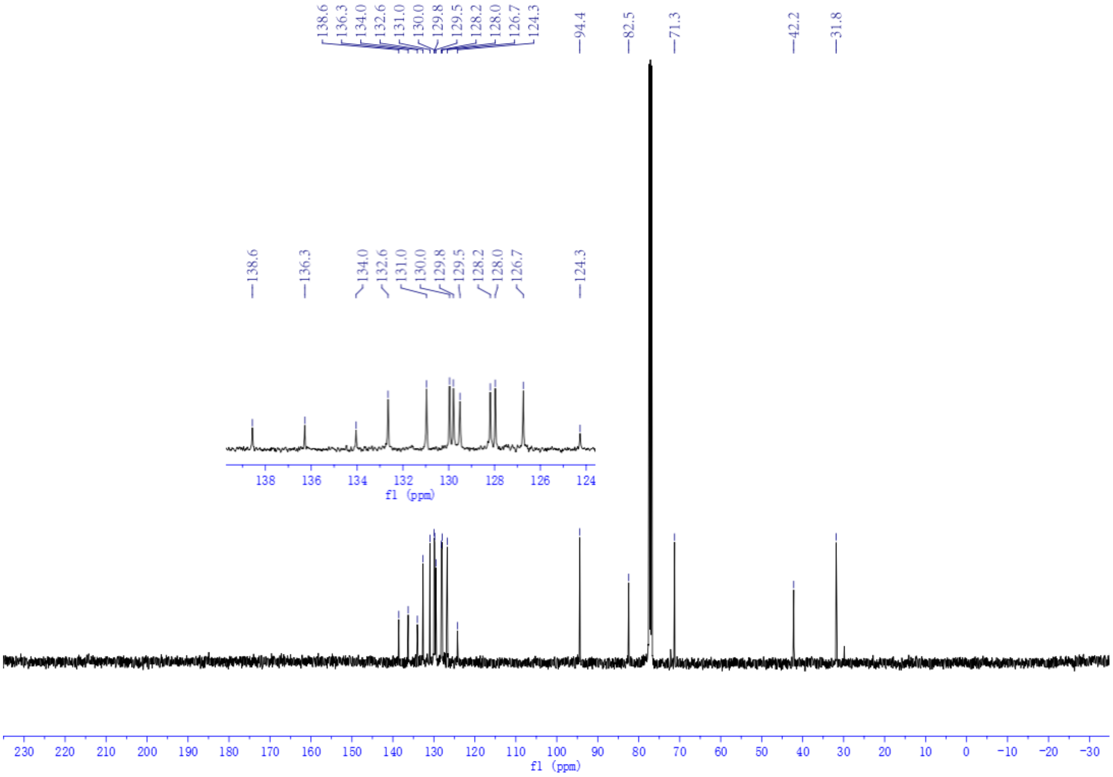
138.6 136.3 134.0 132.6 131.0 130.0 129.8 129.5 128.2 128.0 126.7 124.3
94.4
82.5
71.3
42.2
31.8
138 136 134 132 130 128 126 124
f1 (ppm)
230 220 210 200 190 180 170 160 150 140 130 120 110 100 90 80 70 60 50 40 30 20 10 0 -10 -20 -30
f1 (ppm)

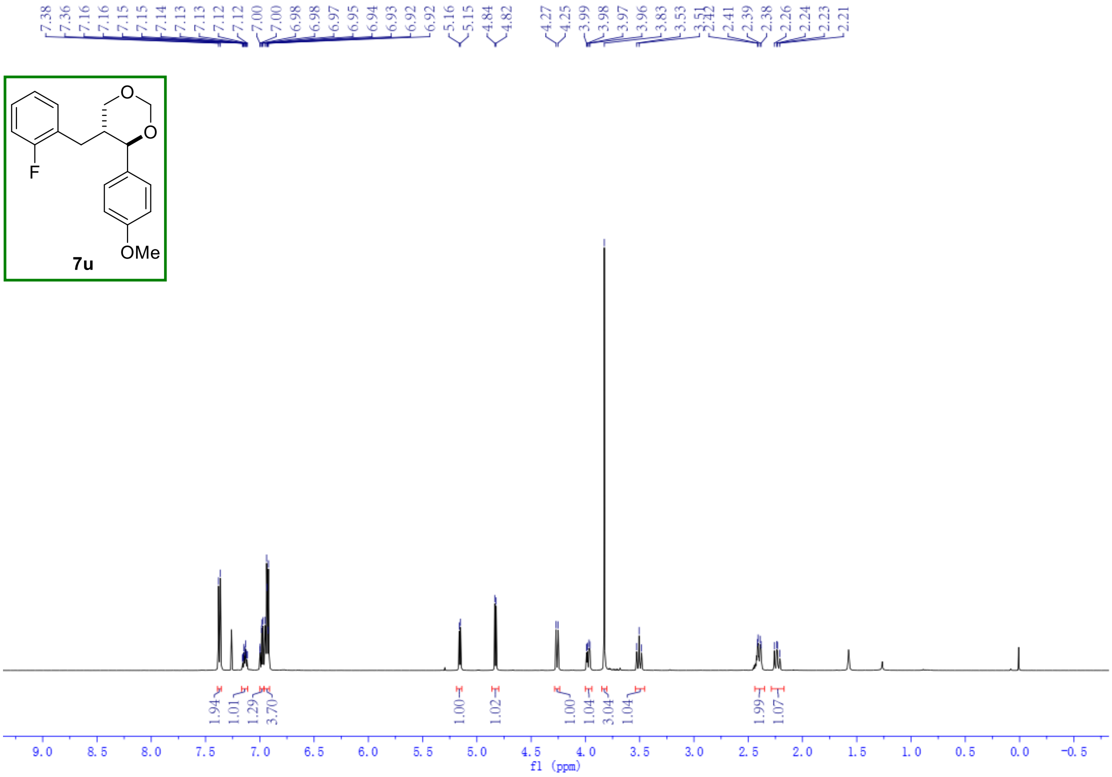
7.38 7.36 7.16 7.16 7.15 7.15 7.14 7.14 7.13 7.13 7.12 7.12 7.12 7.00 7.00 6.98 6.98 6.97 6.95 6.94 6.93 6.92 6.92 5.16 5.15 4.84 4.82 4.27 4.25 3.99 3.98 3.97 3.96 3.83 3.53 3.51 2.42 2.41 2.39 2.38 2.26 2.24 2.23 2.21
F
O
O
OMe
7u
1.94 1.01 1.29 3.70 1.00 1.02 1.00 1.04 3.04 1.04 1.99 1.07
9.0 8.5 8.0 7.5 7.0 6.5 6.0 5.5 5.0 4.5 4.0 3.5 3.0 2.5 2.0 1.5 1.0 0.5 0.0 -0.5
f1 (ppm)

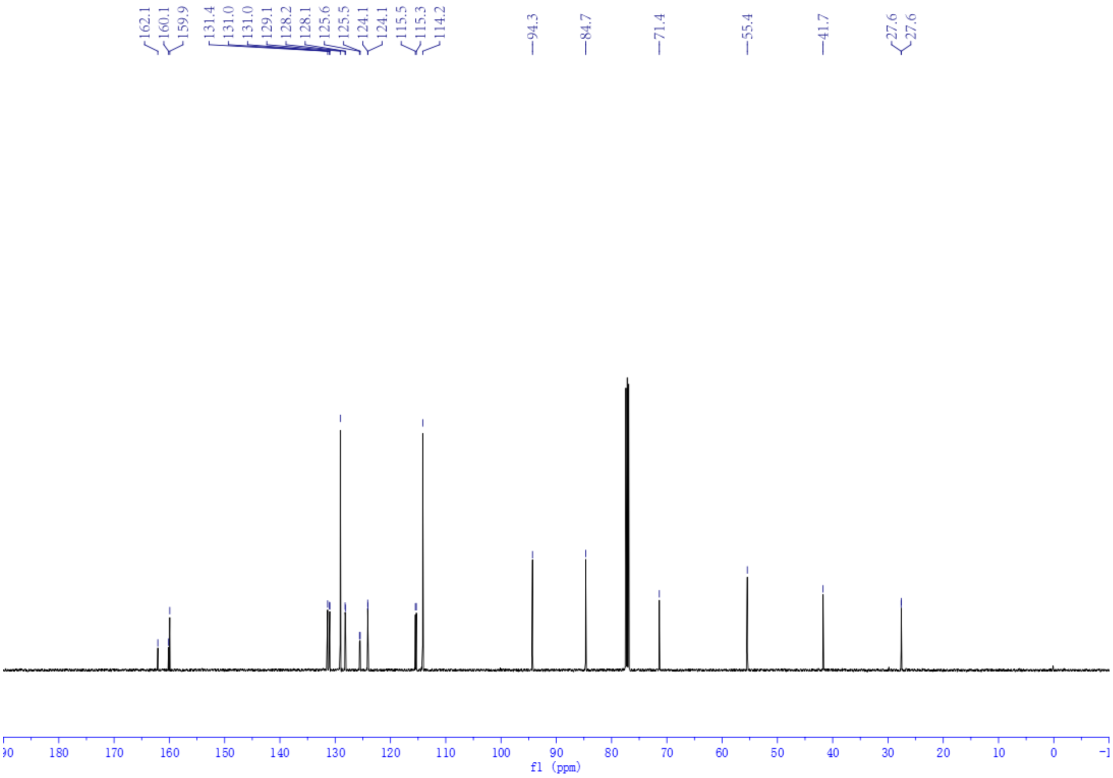
162.1 160.1 159.9 131.4 131.0 131.0 129.1 128.2 128.1 125.6 125.5 124.1 124.1 115.5 115.3 114.2 94.3 84.7 71.4 55.4 41.7 27.6 27.6
180 170 160 150 140 130 120 110 100 90 80 70 60 50 40 30 20 10 0
f1 (ppm)

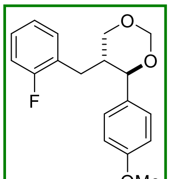
F
O
O
OMe

7u

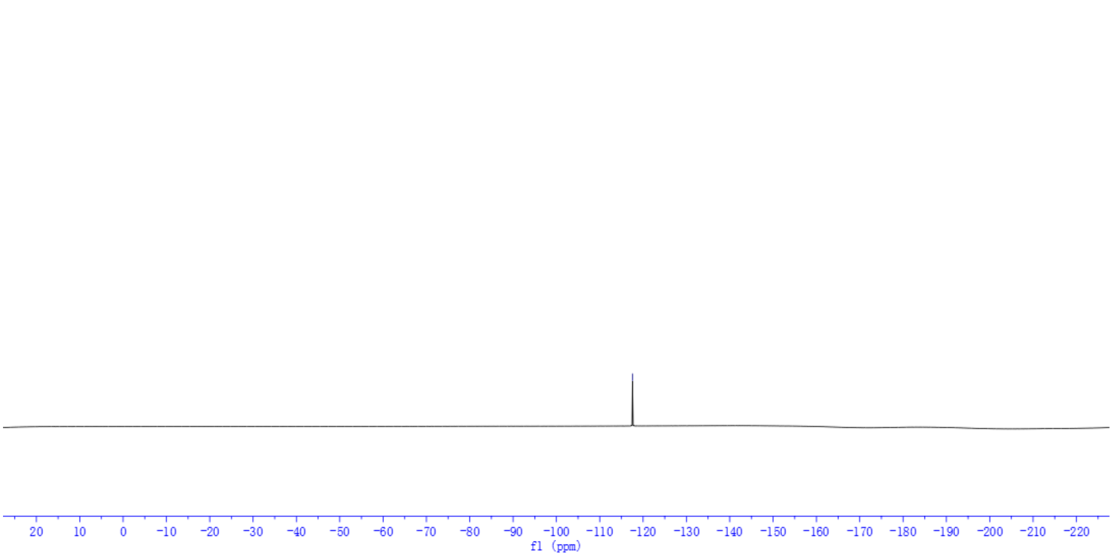
-117.5
20 10 0 -10 -20 -30 -40 -50 -60 -70 -80 -90 -100 -110 -120 -130 -140 -150 -160 -170 -180 -190 -200 -210 -220
f1 (ppm)

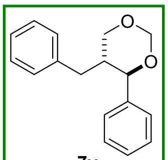

7v

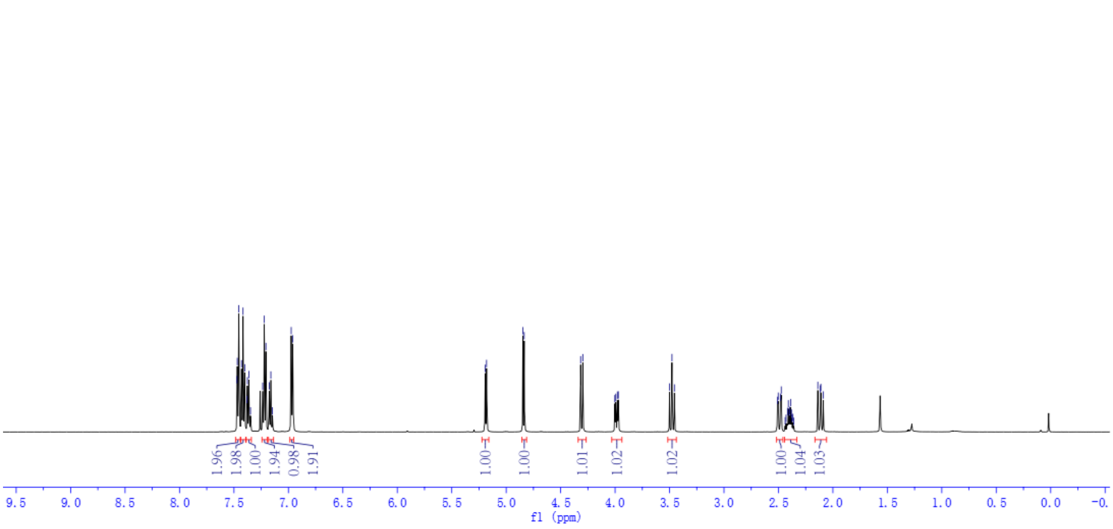
7.47
7.47
7.46
7.43
7.42
7.40
7.38
7.38
7.36
7.35
7.24
7.22
7.21
7.18
7.16
7.15
6.97
6.96
5.19
5.18
4.85
4.83
4.32
4.30
3.99
3.98
3.97
3.50
3.48
3.46
2.51
2.50
2.48
2.47
2.44
2.43
2.42
2.41
2.40
2.40
2.39
2.38
2.38
2.37
2.36
2.14
2.12
2.11
2.09
1.96
1.98
1.00
1.94
0.98
1.91
1.00
1.00
1.01
1.02
1.02
1.00
1.04
1.03
f1 (ppm)

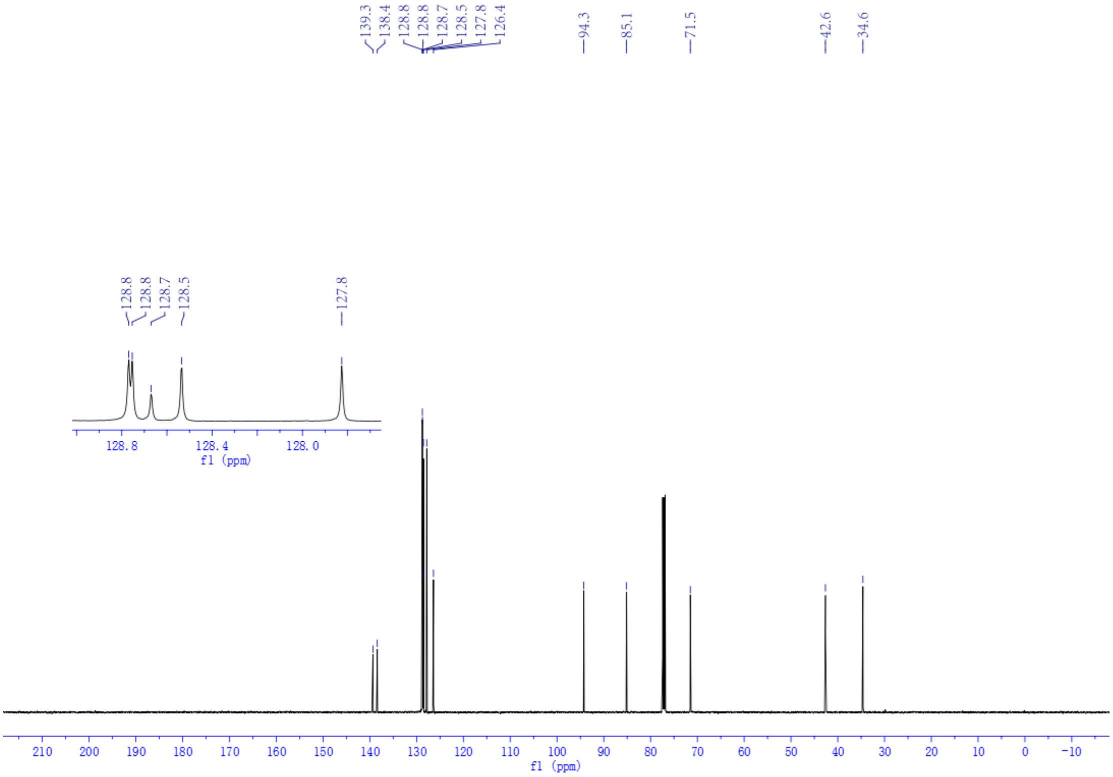
139.3
138.4
128.8
128.8
128.7
128.5
127.8
126.4
94.3
85.1
71.5
42.6
34.6
f1 (ppm)

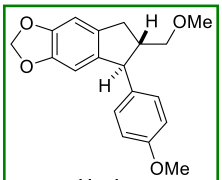

11a-1

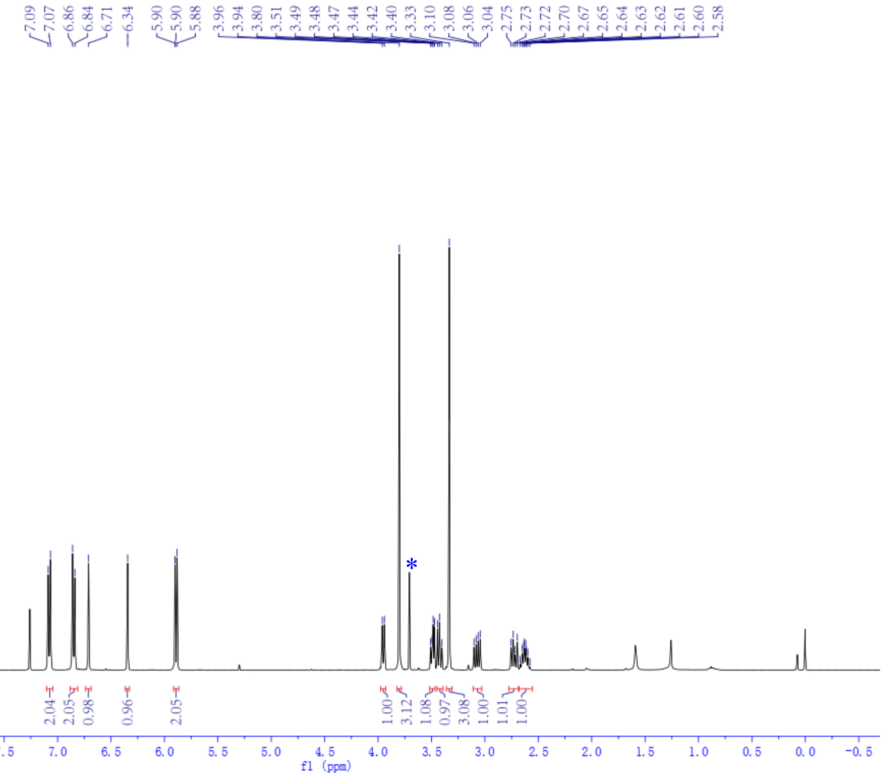

* dioxane

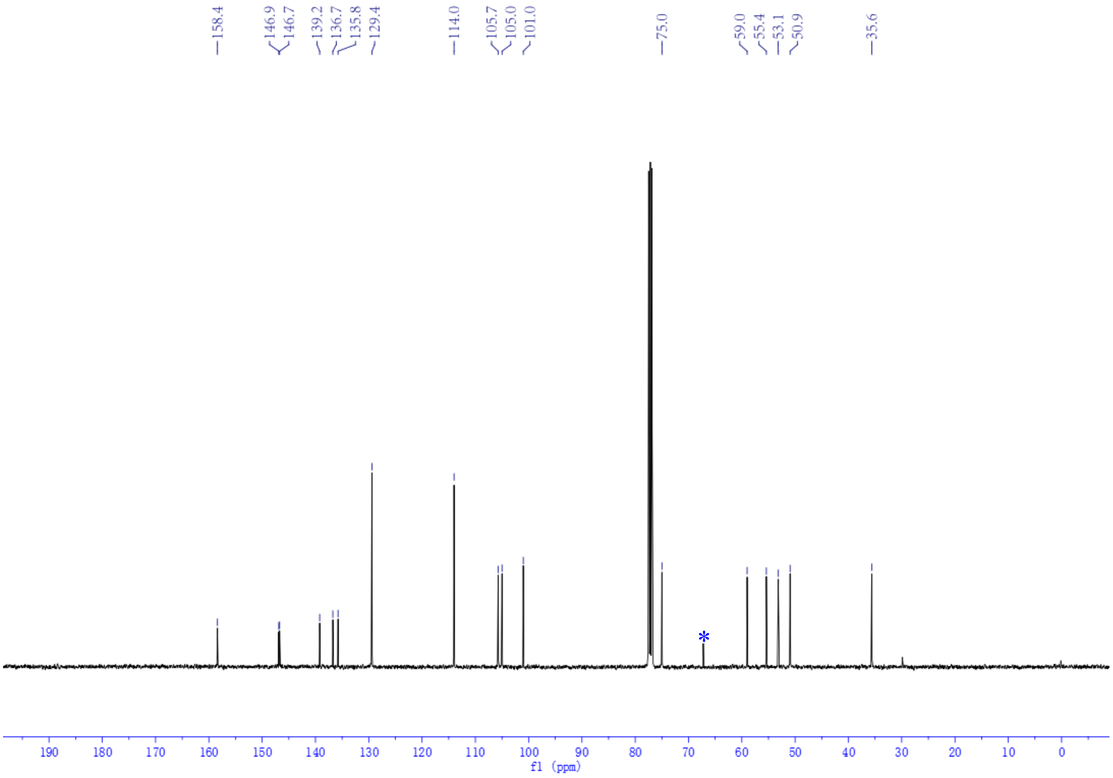

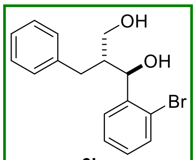

6b

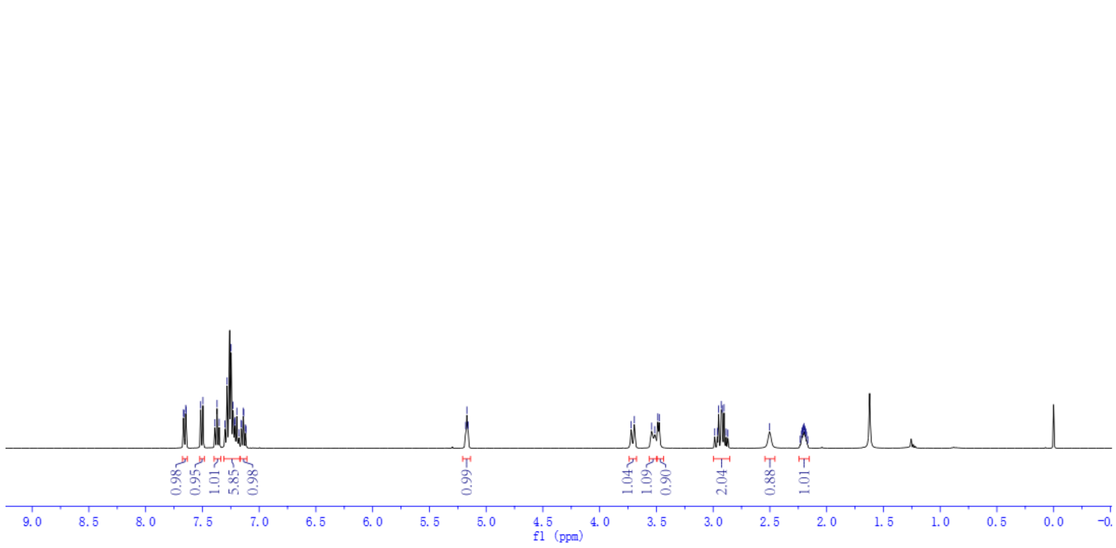

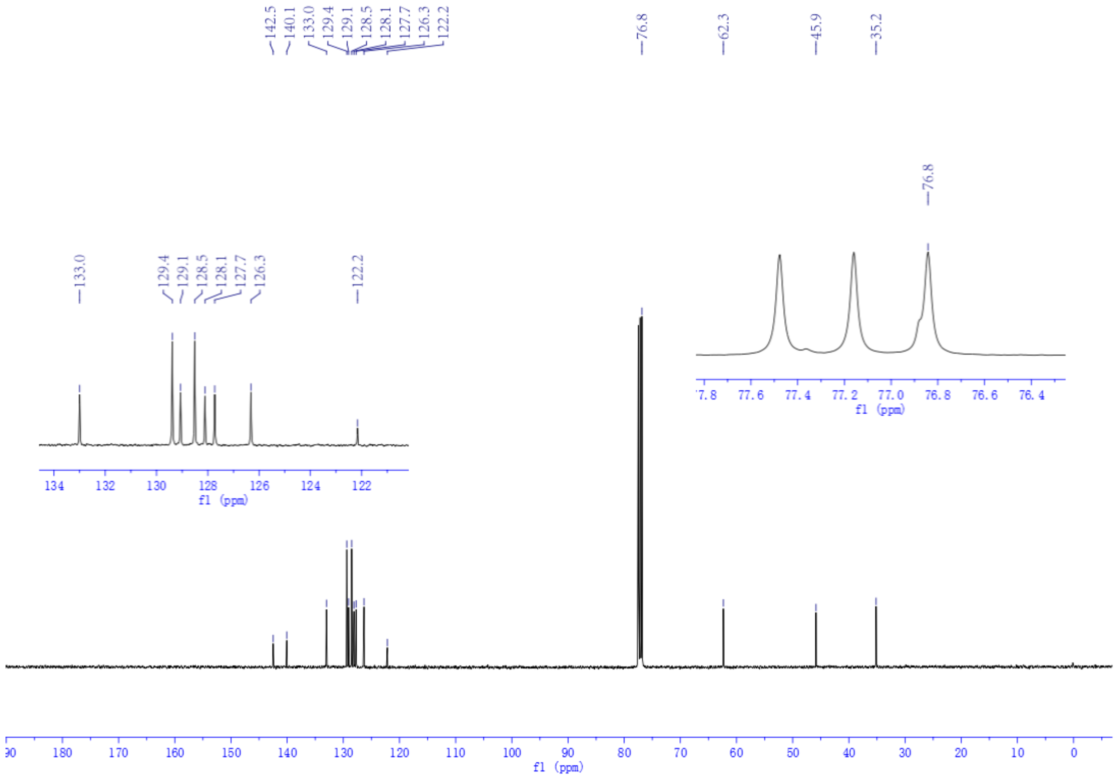

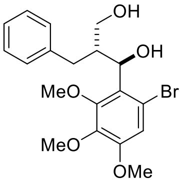
OH
OH
MeO
Br
MeO
OMe

trans-**6h**

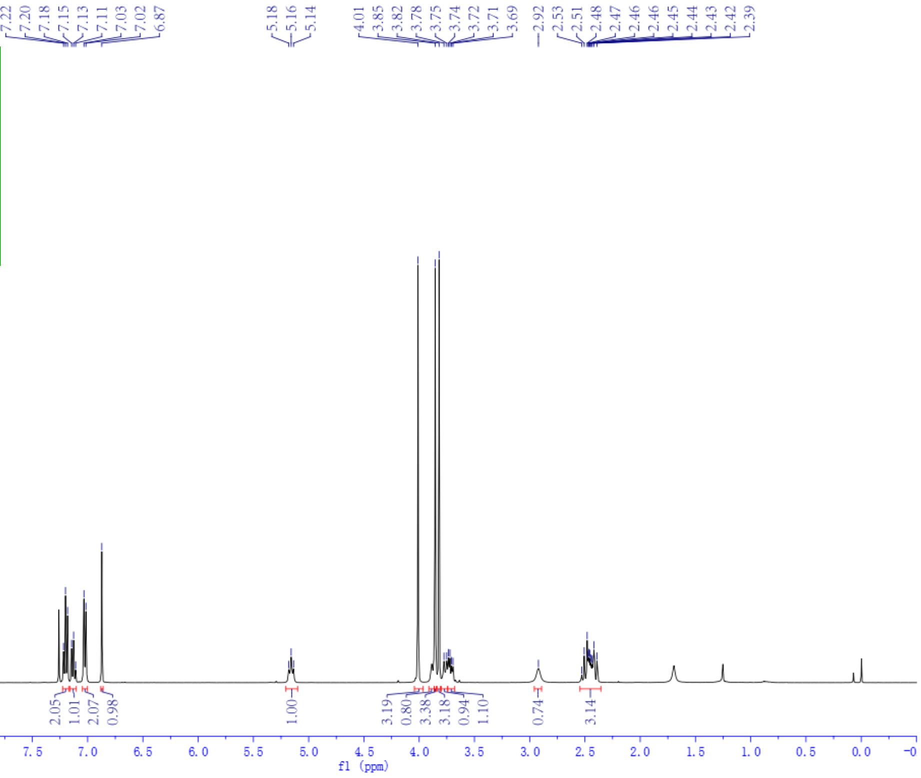
7.22
7.20
7.18
7.15
7.13
7.11
7.03
7.02
6.87
5.18
5.16
5.14
4.01
3.85
3.82
3.78
3.75
3.74
3.72
3.71
3.69
2.92
2.53
2.51
2.48
2.47
2.46
2.46
2.45
2.44
2.43
2.42
2.39
2.05
1.01
2.07
0.98
1.00
3.19
0.80
3.38
3.18
0.94
1.10
0.74
3.14
f1 (ppm)

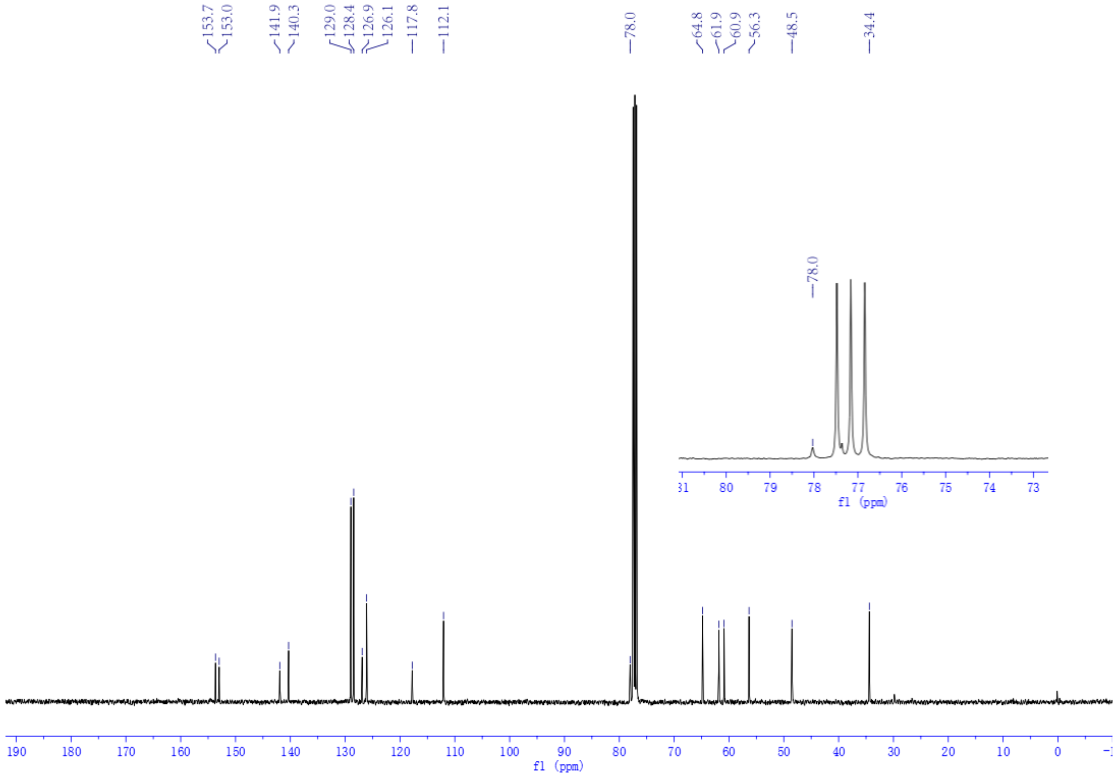
153.7
153.0
141.9
140.3
129.0
128.4
126.9
126.1
117.8
112.1
78.0
64.8
61.9
60.9
56.3
48.5
34.4
78.0
f1 (ppm)
f1 (ppm)

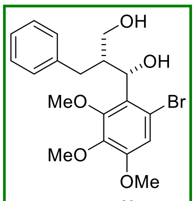
OH
OH
MeO
Br
MeO
OMe
cis-**6h**

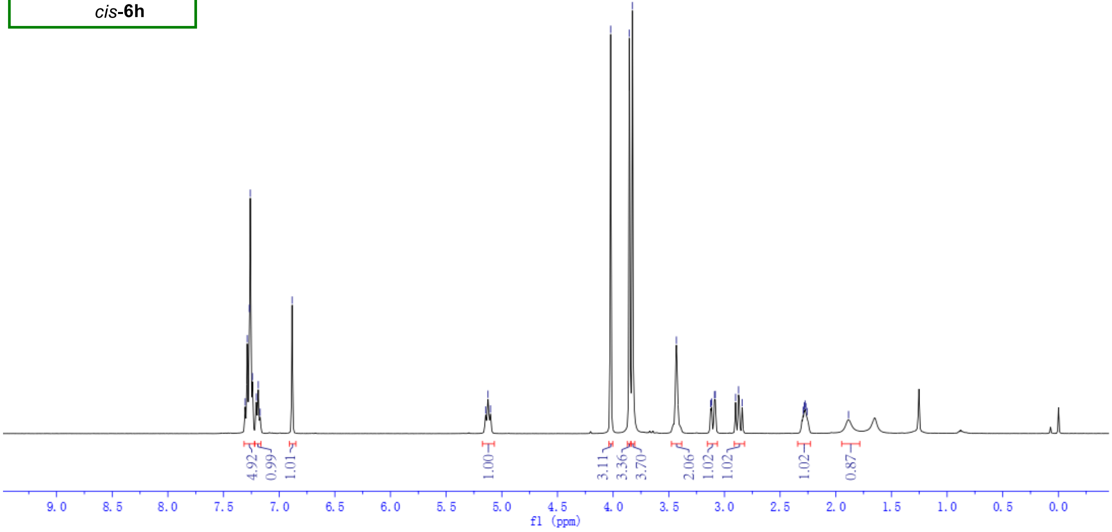
7.31
7.29
7.27
7.26
7.24
7.21
7.19
7.17
6.88
5.15
5.12
5.10
4.02
3.85
3.83
3.43
3.12
3.09
3.08
2.90
2.87
2.28
2.28
2.27
1.89
4.92
0.99
1.01
1.00
3.11
3.36
3.70
2.06
1.02
1.02
1.02
0.87
f1 (ppm)

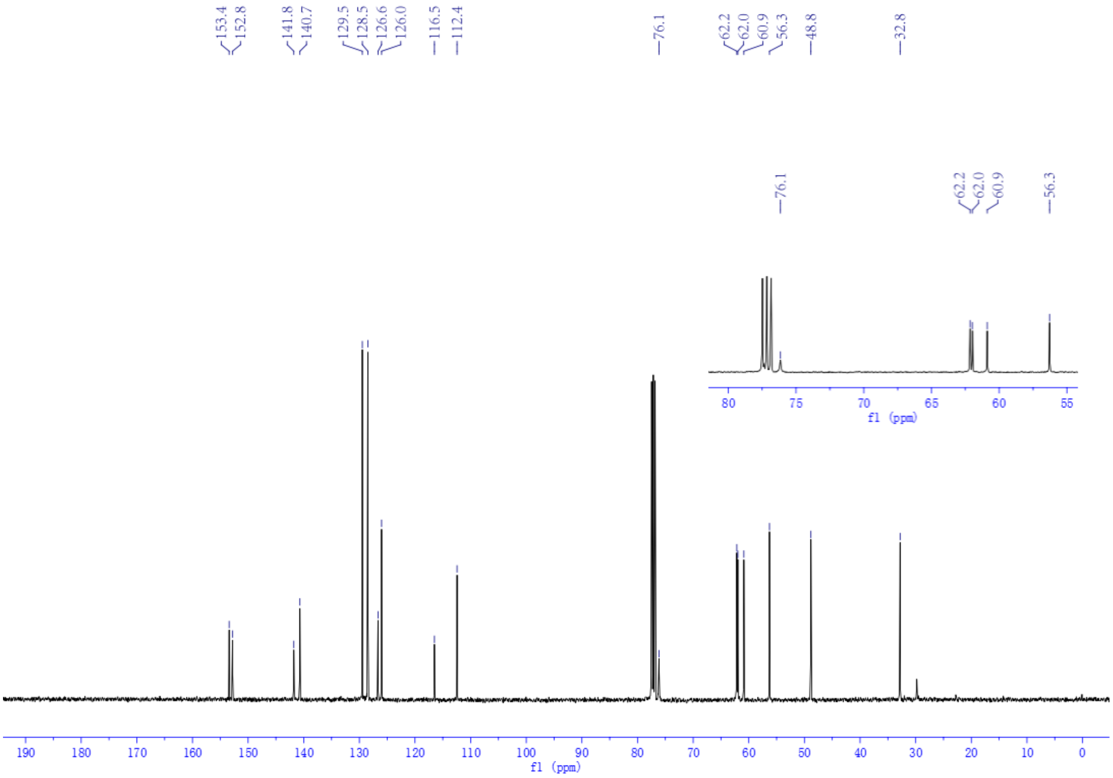
153.4
152.8
141.8
140.7
129.5
128.5
126.6
126.0
116.5
112.4
76.1
62.2
62.0
60.9
56.3
48.8
32.8
f1 (ppm)

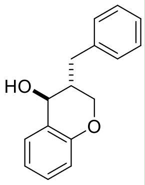

12a

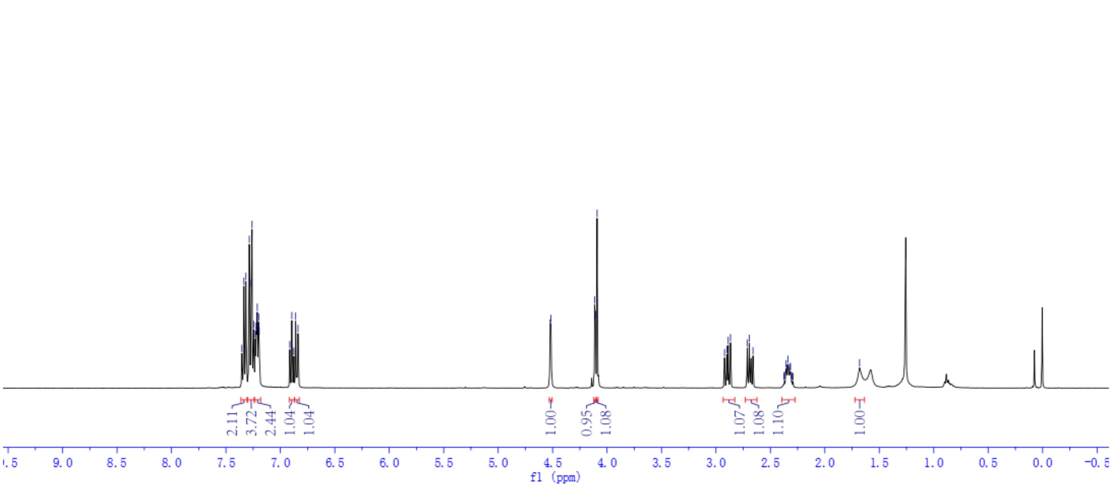
7.35 7.34 7.32 7.29 7.27 7.26 7.25 7.23 7.22 7.22 7.21 7.20 7.20 7.19 6.91 6.89 6.88 6.86 6.84
4.52 4.52 4.11 4.11 4.09
2.92 2.90 2.89 2.87 2.71 2.69 2.66 2.36 2.34 2.32 2.32 1.68
2.11 3.72 2.44 1.04 1.04 1.00 0.95 1.08 1.07 1.08 1.10 1.00
f1 (ppm)

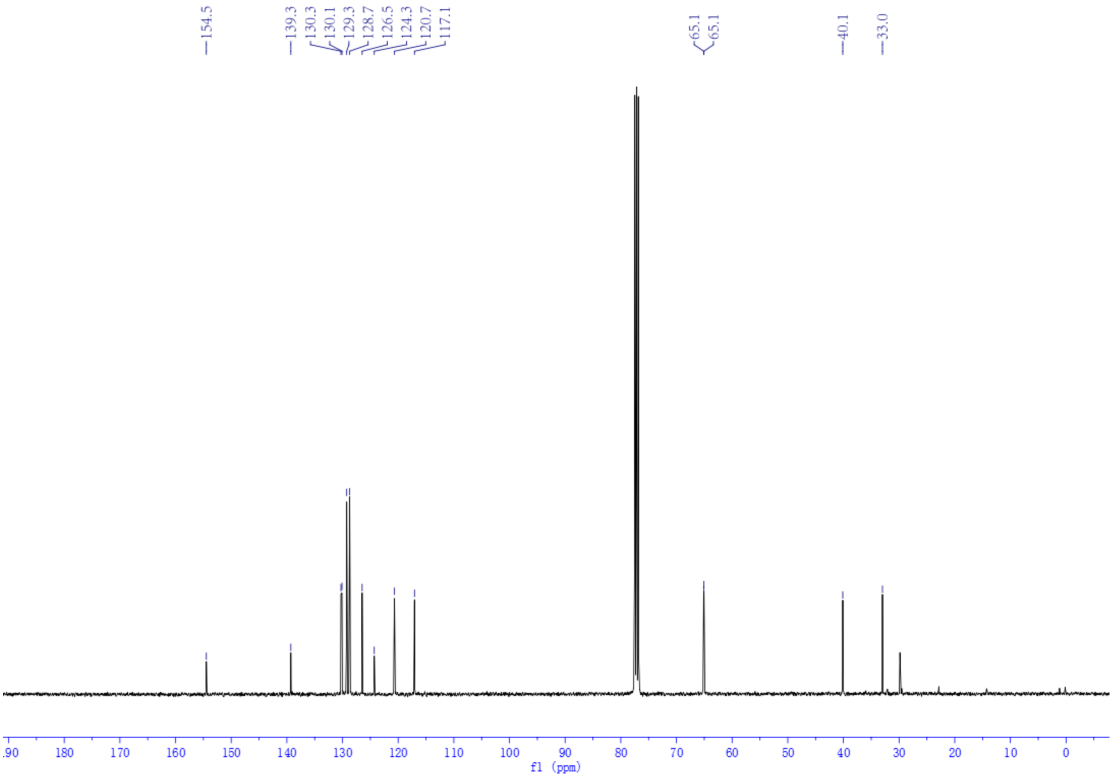
154.5
139.3 130.3 130.1 129.3 128.7 126.5 124.3 120.7 117.1
65.1 65.1
40.1
33.0
f1 (ppm)

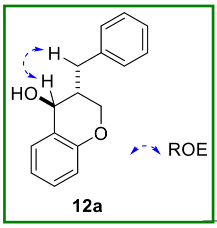
HO
H
H
O
ROE
12a

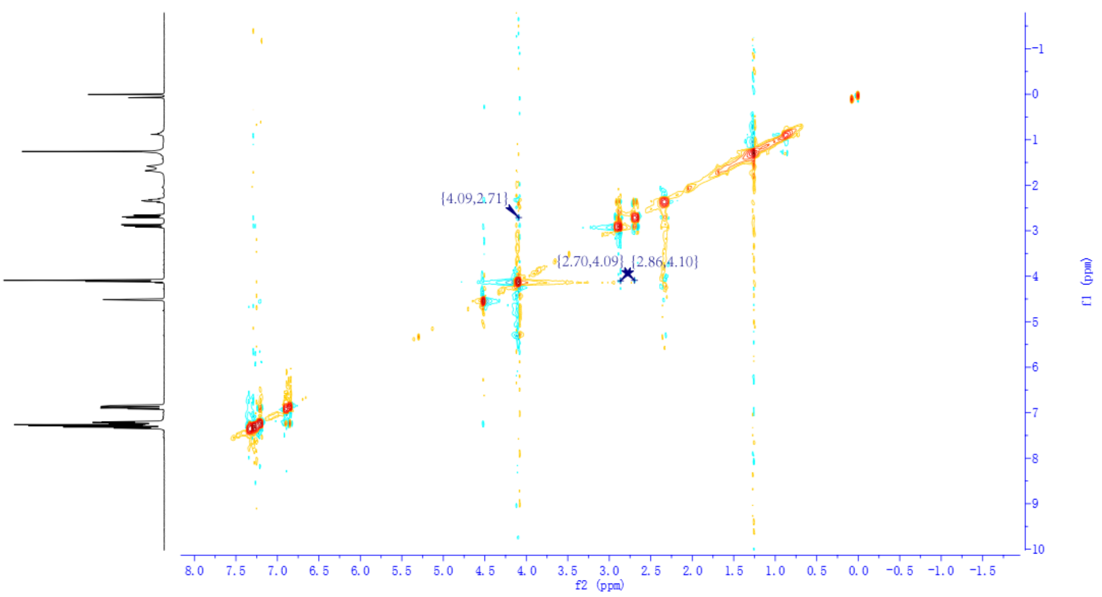
{4.09,2.71}
{2.70,4.09}
{2.86,4.10}
f2 (ppm)
f1 (ppm)

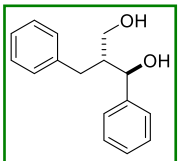
12b

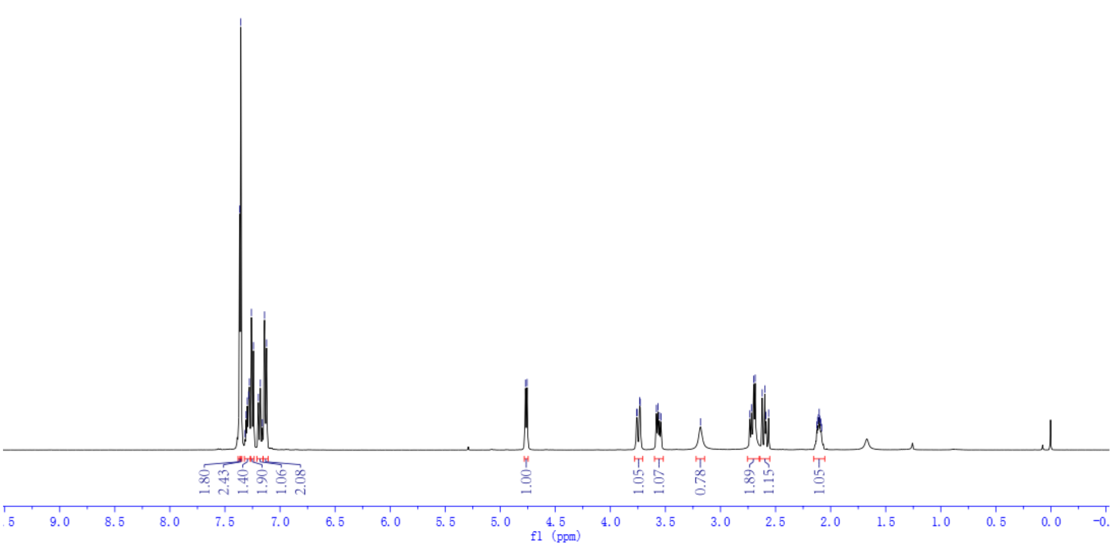
7.37 7.36 7.32 7.31 7.30 7.29 7.28 7.26 7.24 7.20 7.18 7.16 7.14 7.12
4.77 4.76
3.76 3.76 3.73 3.73 3.58 3.57 3.55 3.18
2.70 2.68 2.62 2.60 2.13 2.12 2.11 2.10 2.10 2.09 2.08
1.80 2.43 1.40 1.90 1.06 2.08 1.00 1.05 1.07 0.78 1.89 1.15 1.05
f1 (ppm)

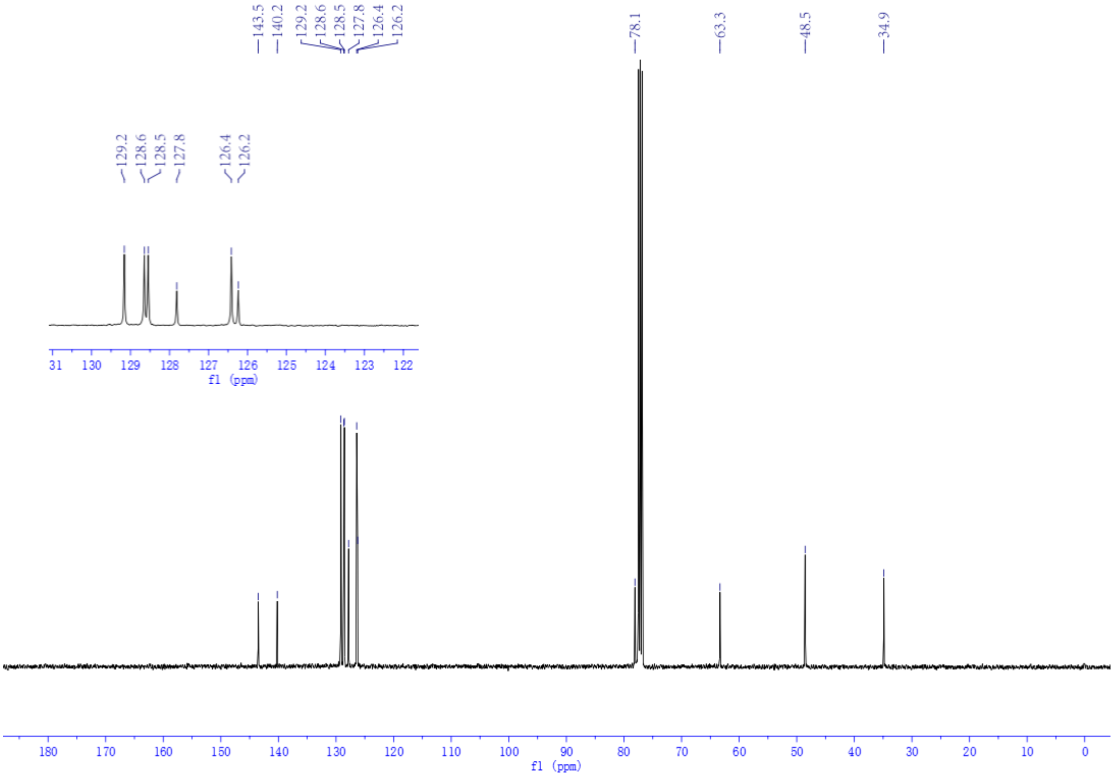
143.5 140.2 129.2 128.6 128.5 127.8 126.4 126.2
78.1
63.3
48.5
34.9
129.2 128.6 128.5 127.8 126.4 126.2
f1 (ppm)
f1 (ppm)

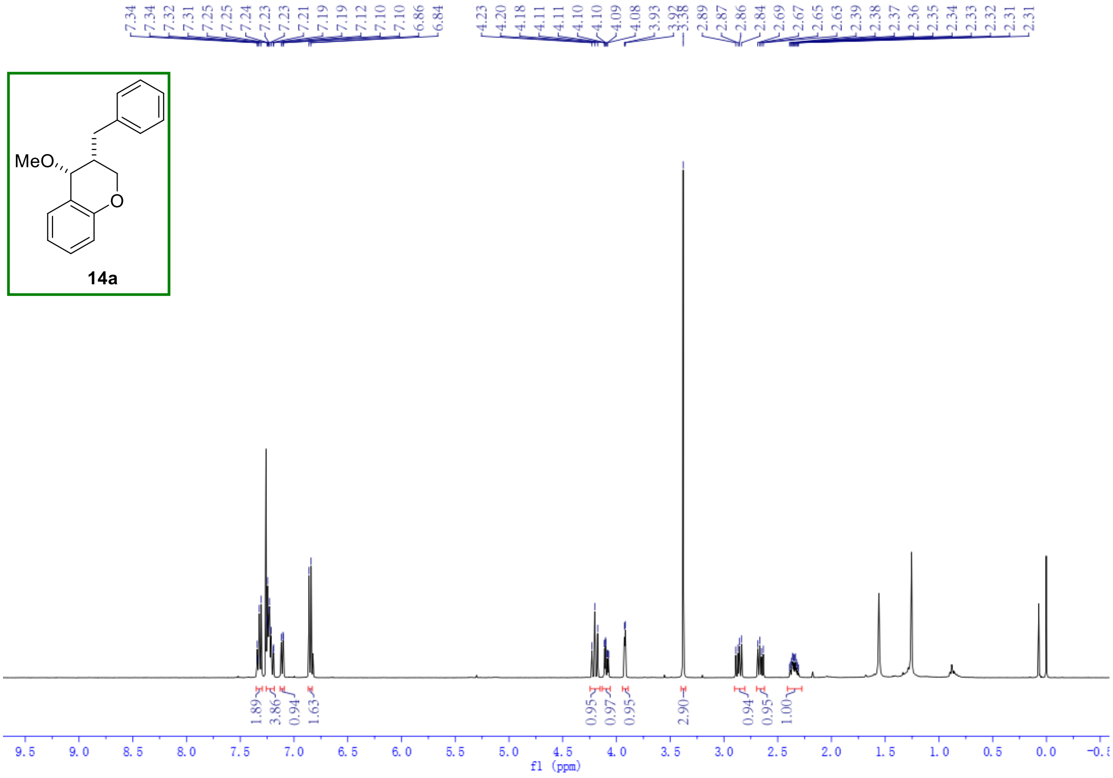

7.34 7.34 7.32 7.31 7.25 7.25 7.24 7.23 7.23 7.21 7.19 7.19 7.12 7.10 7.10 6.86 6.84
4.23 4.20 4.18 4.11 4.11 4.10 4.10 4.09 4.08 3.93 3.92 3.38 2.89 2.87 2.86 2.84 2.69 2.67 2.65 2.63 2.39 2.38 2.37 2.36 2.35 2.34 2.33 2.32 2.31 2.31
MeO
O
14a
1.89 3.86 0.94 1.63 0.95 0.97 0.95 2.90 0.94 0.95 1.00
9.5 9.0 8.5 8.0 7.5 7.0 6.5 6.0 5.5 5.0 4.5 4.0 3.5 3.0 2.5 2.0 1.5 1.0 0.5 0.0 -0.5
f1 (ppm)

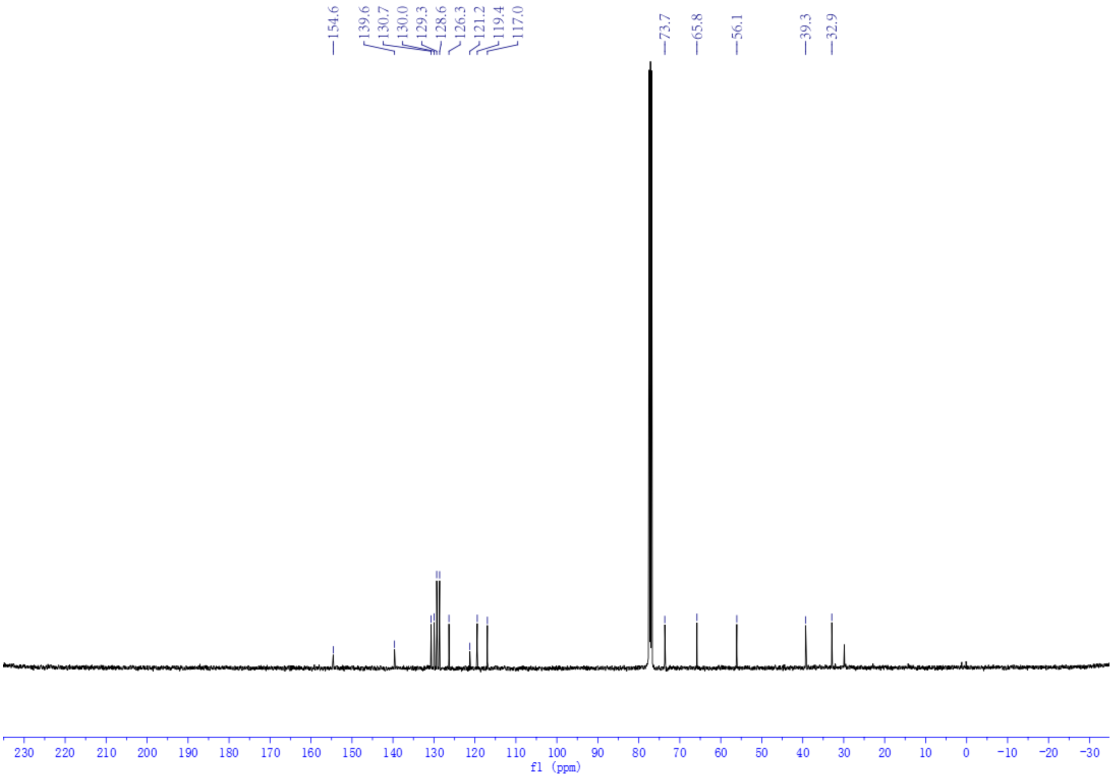

154.6 139.6 130.7 130.0 129.3 128.6 126.3 121.2 119.4 117.0 73.7 65.8 56.1 39.3 32.9
230 220 210 200 190 180 170 160 150 140 130 120 110 100 90 80 70 60 50 40 30 20 10 0 -10 -20 -30
f1 (ppm)

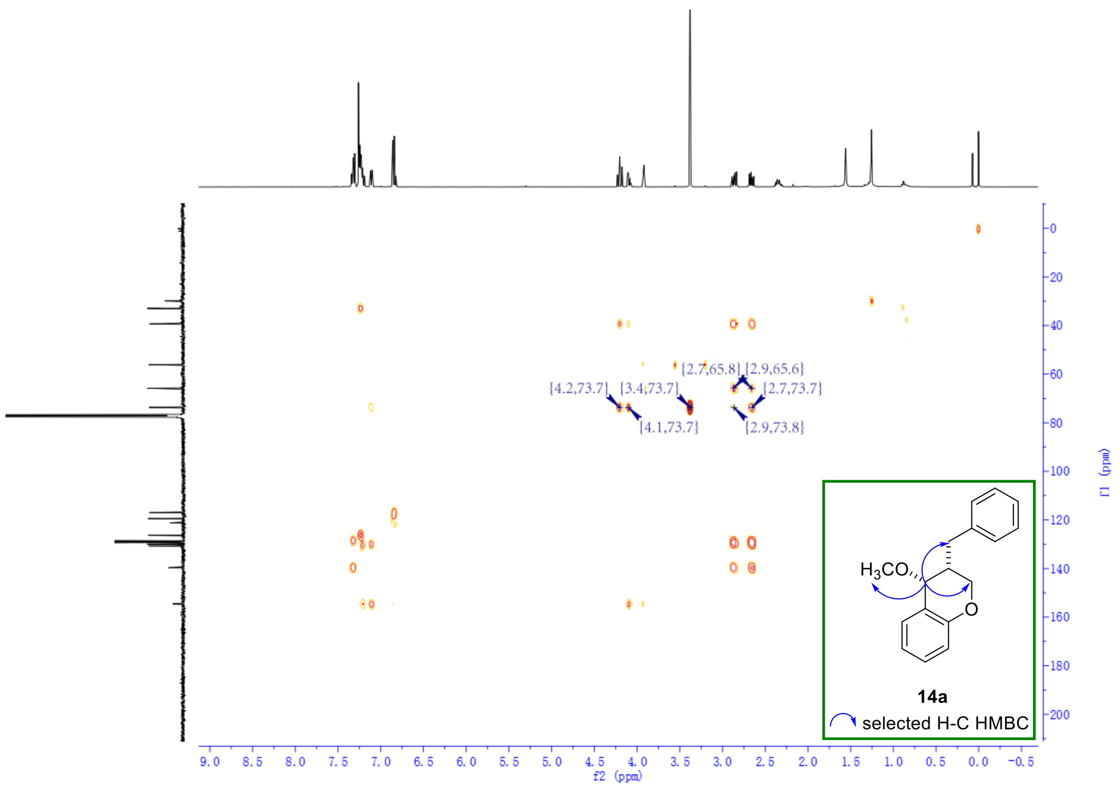

{2.7,65.8}
{2.9,65.6}
{4.2,73.7}
{3.4,73.7}
{2.7,73.7}
{4.1,73.7}
{2.9,73.8}
H_3CO
O
14a
selected H-C HMBC
f2 (ppm)
f1 (ppm)

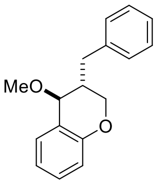
MeO
O
14b

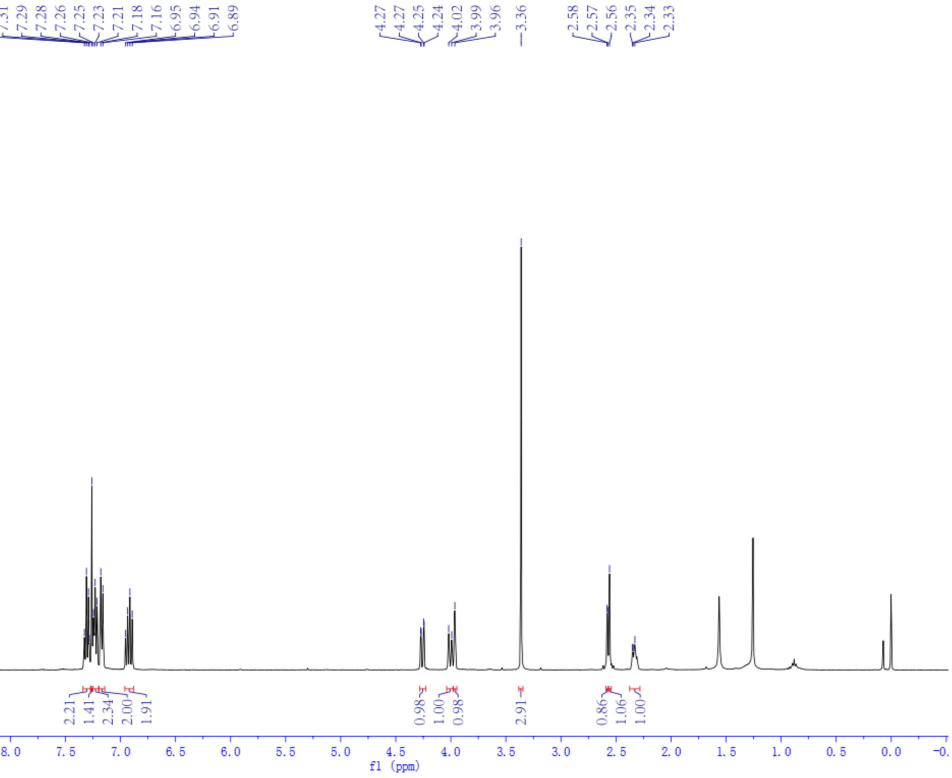
7.33 7.31 7.29 7.28 7.26 7.25 7.23 7.21 7.18 7.16 6.95 6.94 6.91 6.89
4.27 4.27 4.25 4.24 4.02 3.99 3.96
3.36
2.58 2.57 2.56 2.35 2.34 2.33
2.21 1.41 2.34 2.00 1.91
0.98 1.00 0.98
2.91
0.86 1.06 1.00
f1 (ppm)

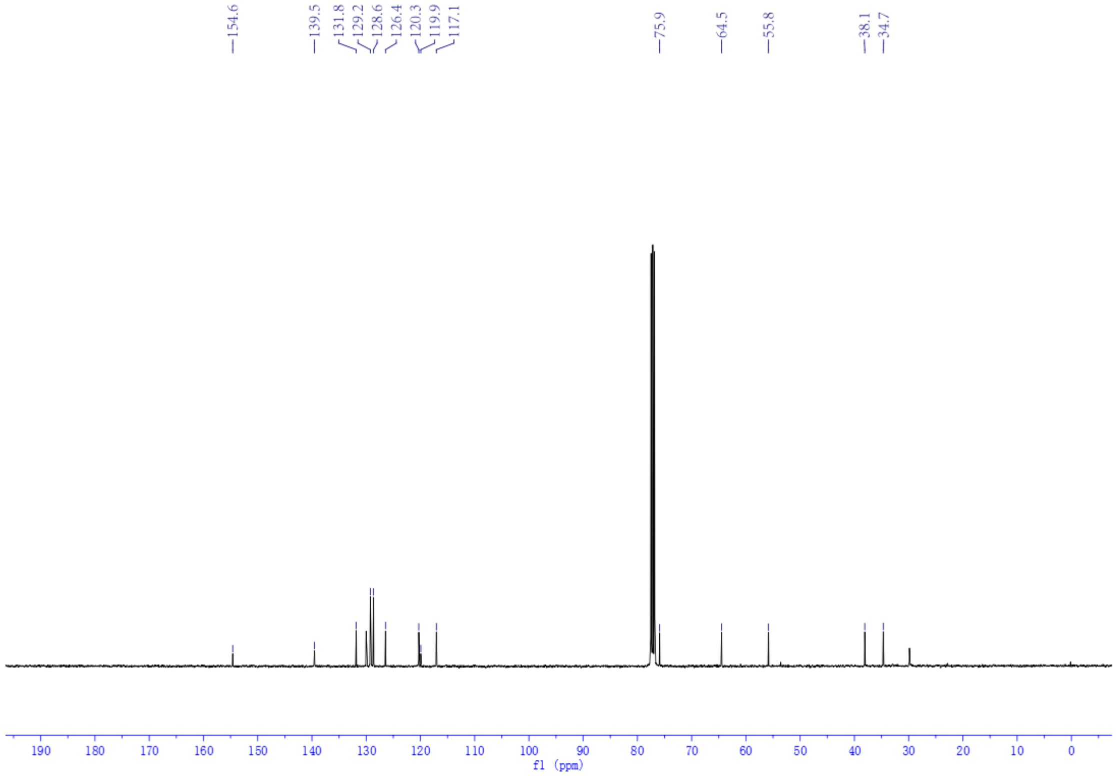
154.6
139.5 131.8 129.2 128.6 126.4 120.3 119.9 117.1
75.9
64.5
55.8
38.1 34.7
f1 (ppm)

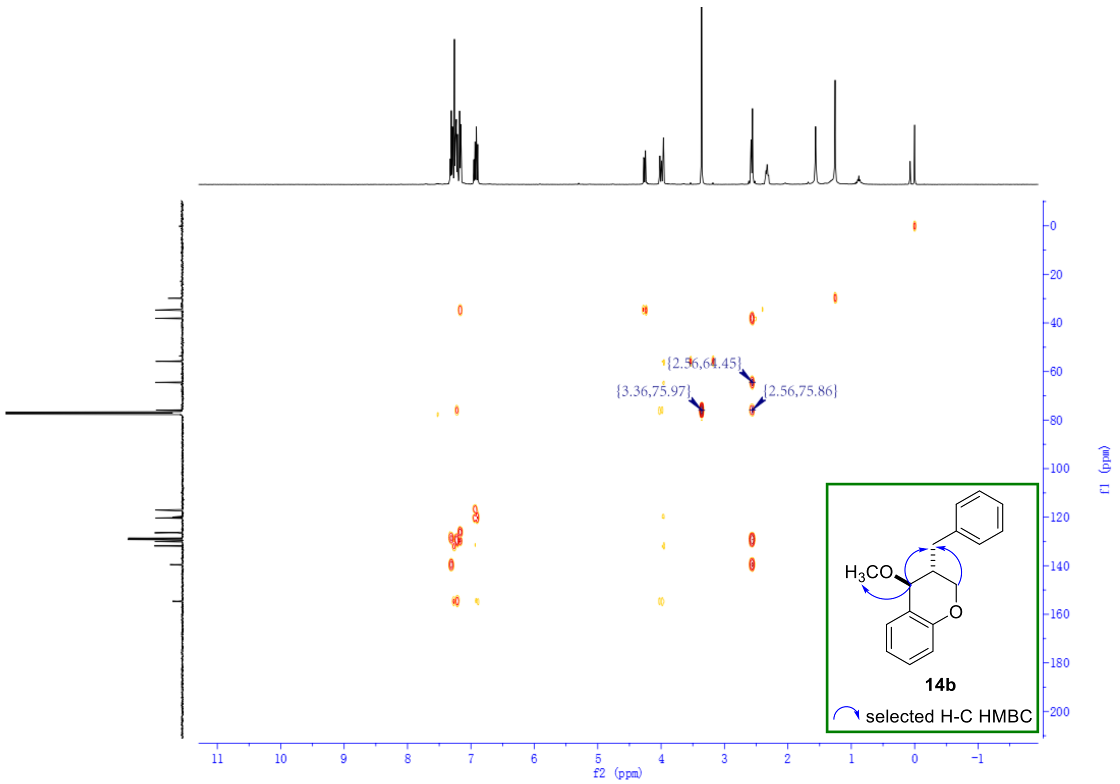

[2.56,64.45]
[3.36,75.97]
[2.56,75.86]
H3CO
O
14b
selected H-C HMBC
f2 (ppm)
f1 (ppm)

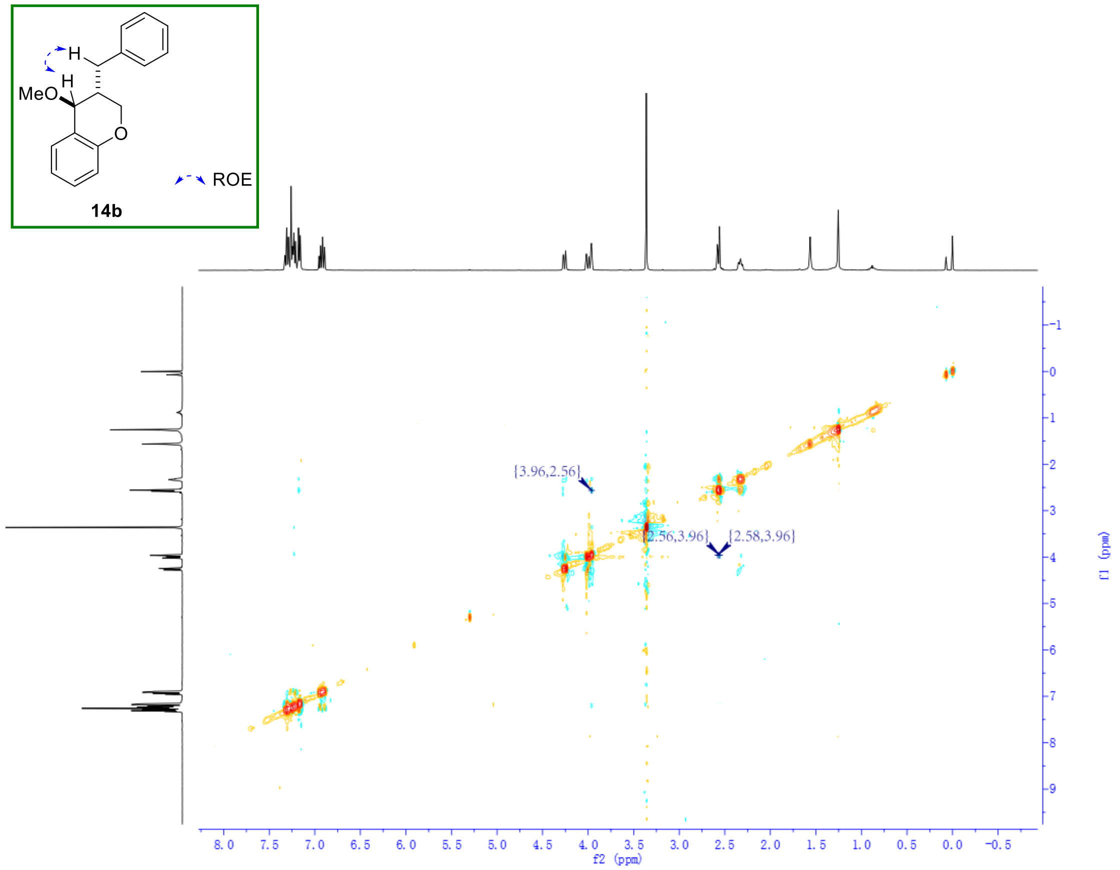

H
MeO
H
O
ROE
14b
[3.96,2.56]
[2.56,3.96]
[2.58,3.96]
f2 (ppm)
f1 (ppm)

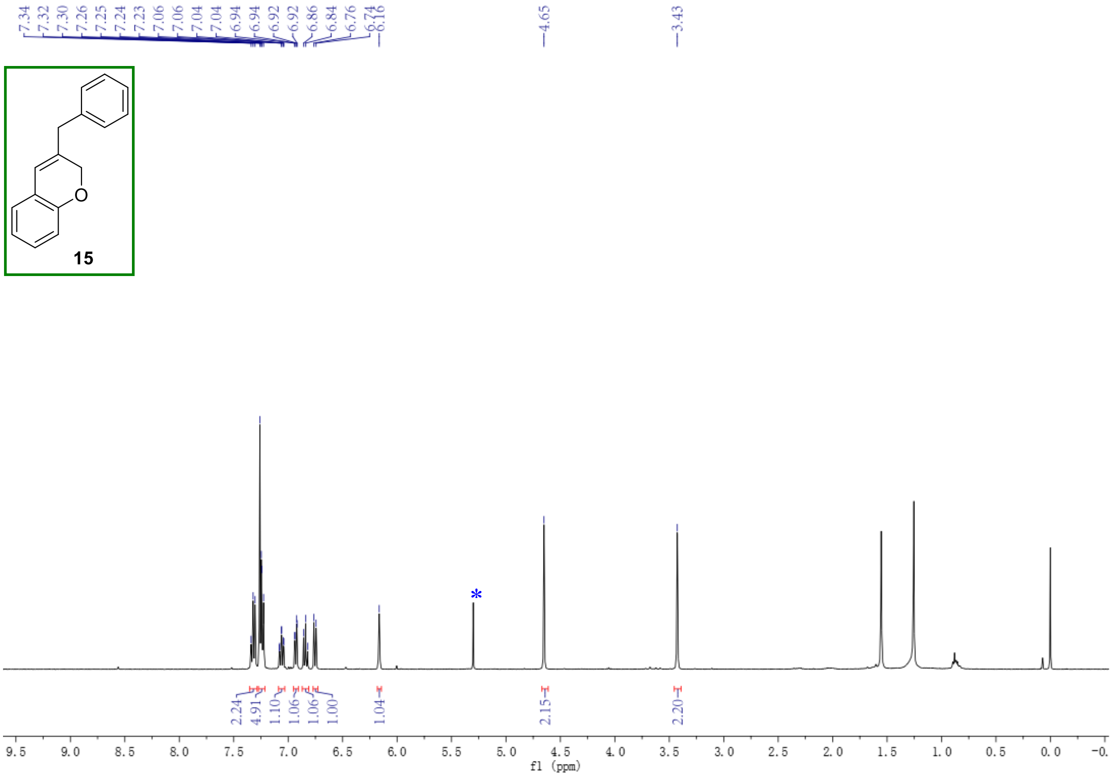

7.34
7.32
7.30
7.26
7.25
7.24
7.23
7.06
7.06
7.04
7.04
6.94
6.94
6.92
6.92
6.86
6.84
6.76
6.74
6.16
4.65
3.43
O
15
*
2.24
4.91
1.10
1.06
1.06
1.00
1.04
2.15
2.20
9.5
9.0
8.5
8.0
7.5
7.0
6.5
6.0
5.5
5.0
4.5
4.0
3.5
3.0
2.5
2.0
1.5
1.0
0.5
0.0
f1 (ppm)

* DCM

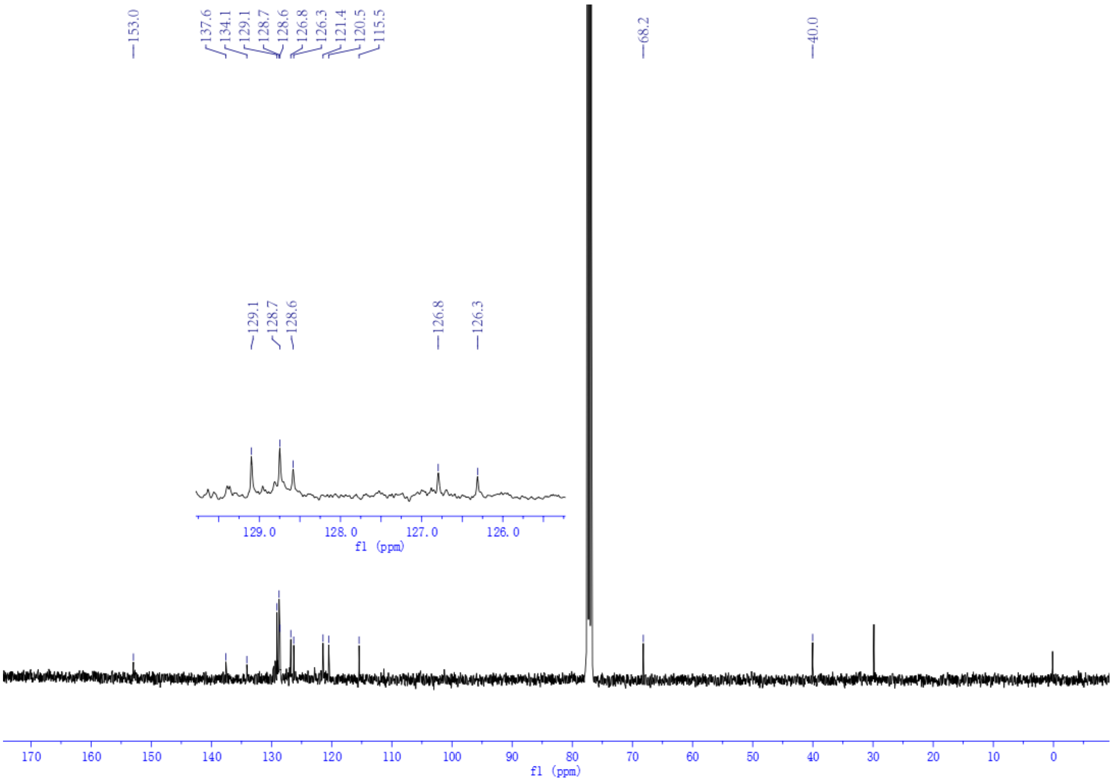

153.0
137.6
134.1
129.1
128.7
128.6
126.8
126.3
121.4
120.5
115.5
68.2
40.0
129.1
128.7
128.6
126.8
126.3
129.0
128.0
127.0
126.0
f1 (ppm)
170
160
150
140
130
120
110
100
90
80
70
60
50
40
30
20
10
0
f1 (ppm)

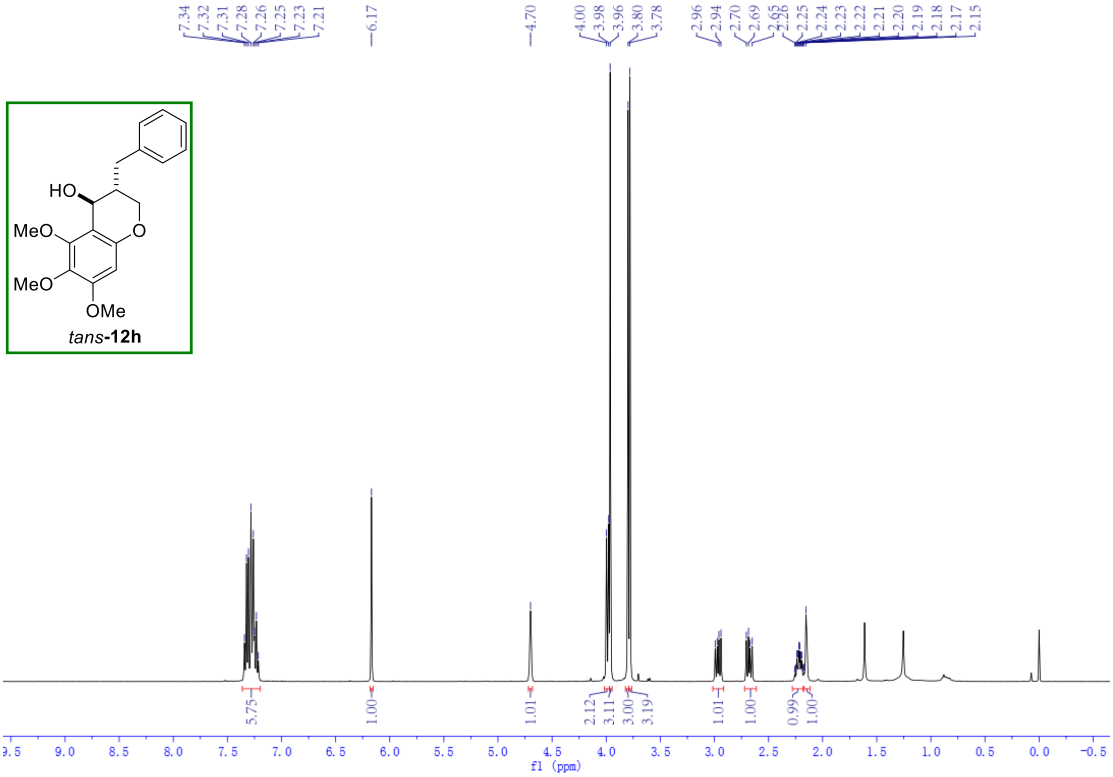

tans-**12h**

*dioxane

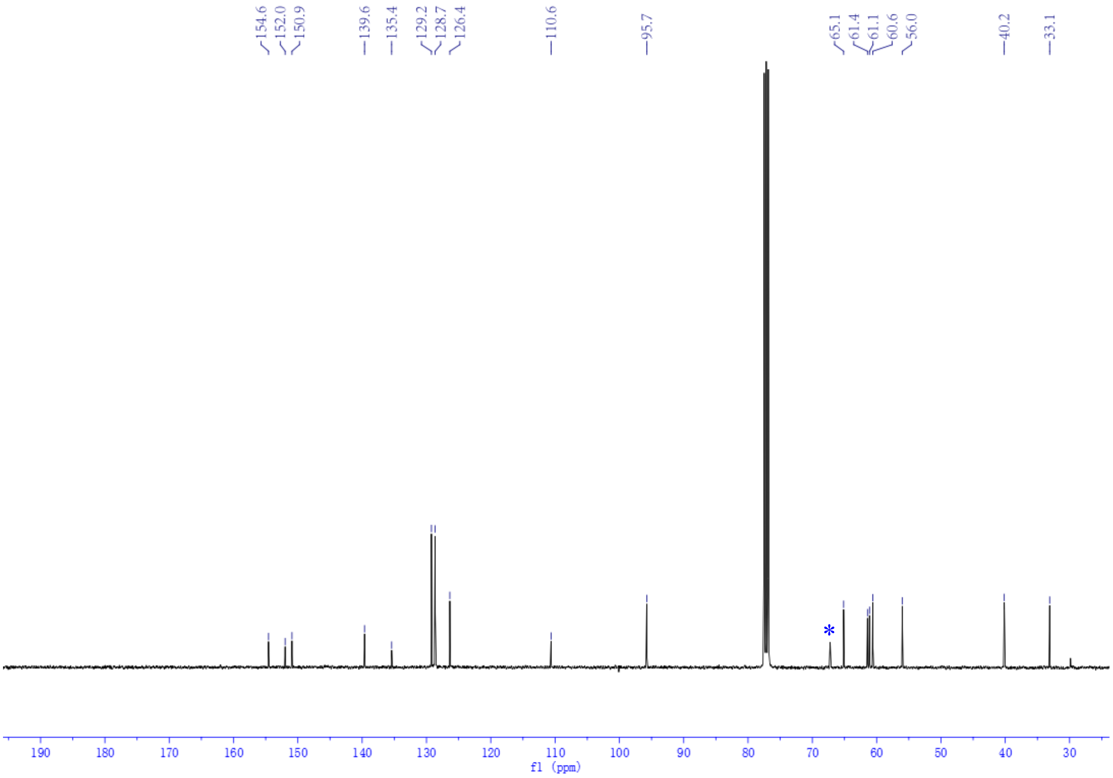

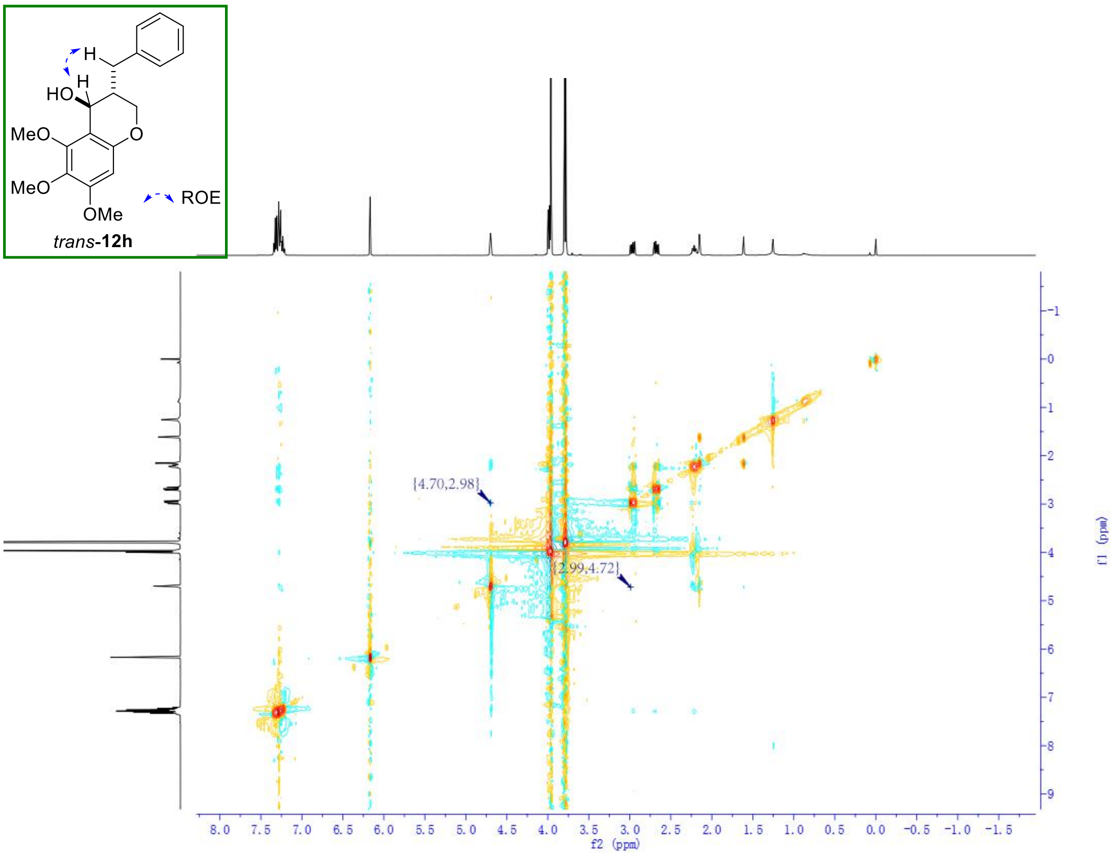

HO
H
H
MeO
MeO
OMe
O
ROE
trans-12h
{4.70,2.98}
{2.99,4.72}
f2 (ppm)
f1 (ppm)

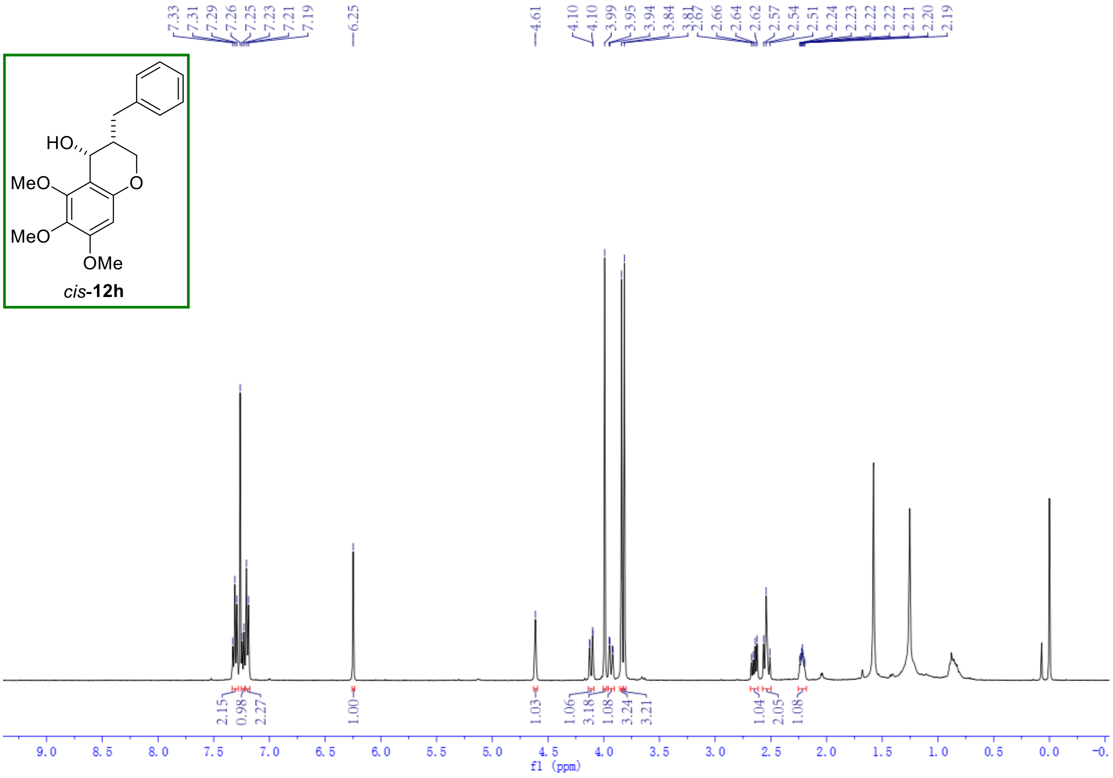
HO
MeO
O
MeO
OMe
cis-12h
7.33 7.31 7.29 7.26 7.25 7.23 7.21 7.19
6.25
4.61
4.10 4.10 3.99 3.95 3.94 3.84 3.81 2.67 2.66 2.64 2.62 2.57 2.54 2.51 2.24 2.23 2.22 2.22 2.21 2.20 2.19
2.15 0.98 2.27 1.00 1.03 1.06 3.18 1.08 3.24 3.21 1.04 2.05 1.08
9.0 8.5 8.0 7.5 7.0 6.5 6.0 5.5 5.0 4.5 4.0 3.5 3.0 2.5 2.0 1.5 1.0 0.5 0.0
f1 (ppm)

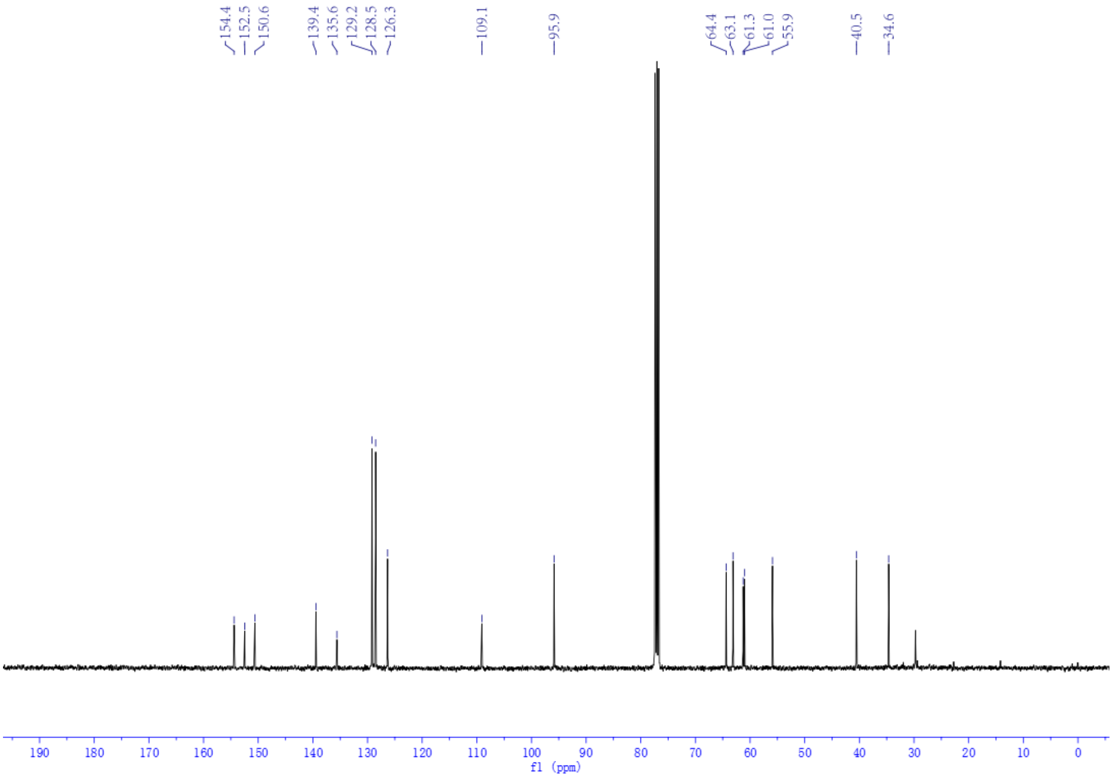
154.4 152.5 150.6
139.4 135.6 129.2 128.5 126.3
109.1
95.9
64.4 63.1 61.3 61.0 55.9
40.5
34.6
190 180 170 160 150 140 130 120 110 100 90 80 70 60 50 40 30 20 10 0
f1 (ppm)

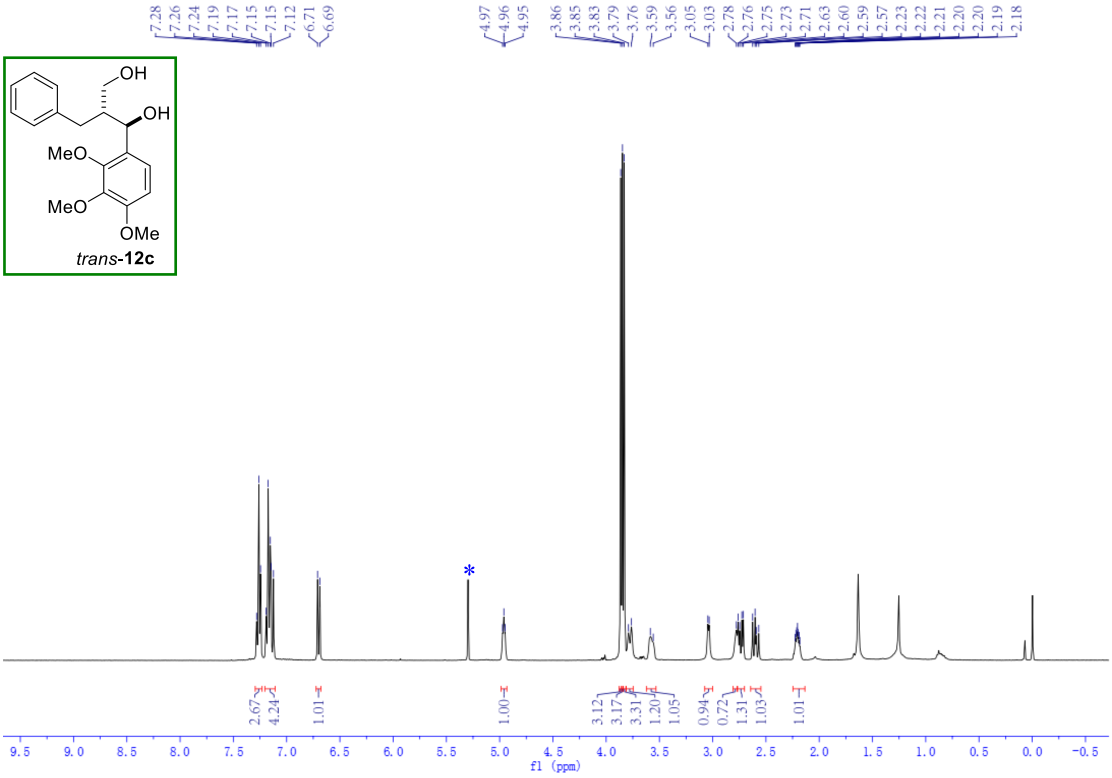

7.28
7.26
7.24
7.19
7.17
7.15
7.15
7.12
6.71
6.69
4.97
4.96
4.95
3.86
3.85
3.83
3.79
3.76
3.59
3.56
3.05
3.03
2.78
2.76
2.75
2.73
2.71
2.63
2.60
2.59
2.57
2.23
2.22
2.21
2.20
2.20
2.19
2.18
OH
OH
MeO
MeO
OMe
trans-**12c**
*
2.67
4.24
1.01
1.00
3.12
3.17
3.31
1.20
1.05
0.94
0.72
1.31
1.03
1.01
f1 (ppm)

* DCM

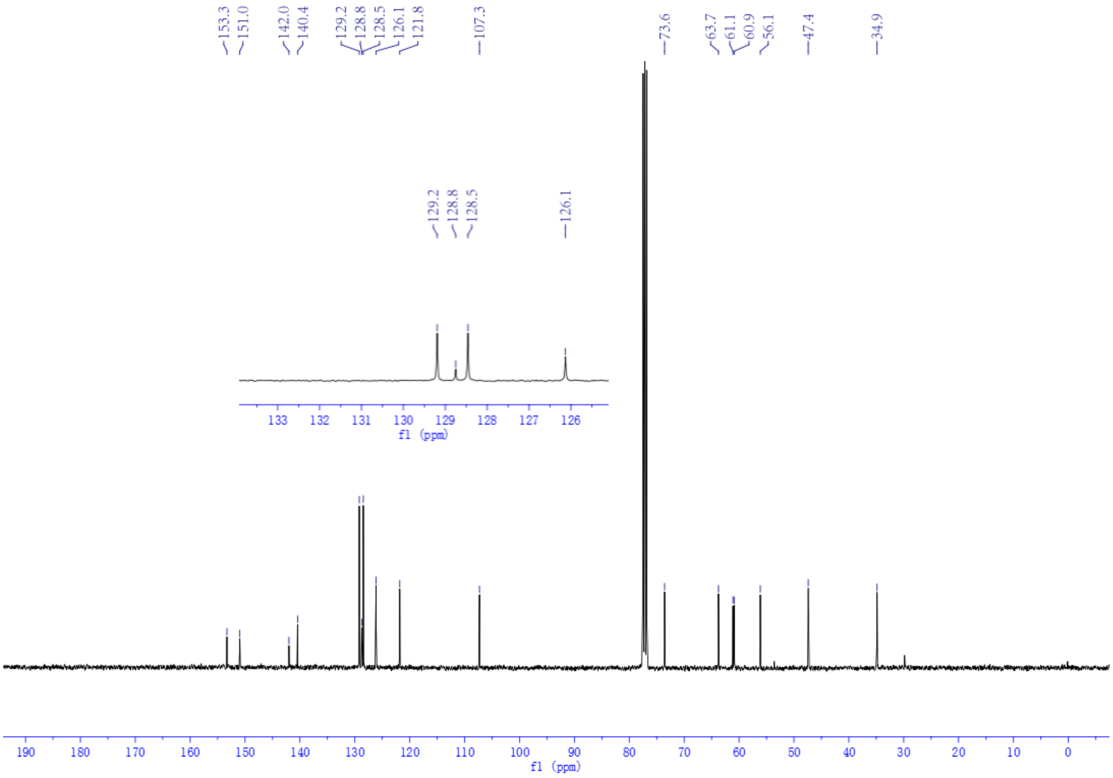

153.3
151.0
142.0
140.4
129.2
128.8
128.5
126.1
121.8
107.3
73.6
63.7
61.1
60.9
56.1
47.4
34.9
129.2
128.8
128.5
126.1
f1 (ppm)
f1 (ppm)

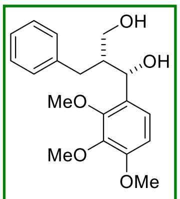
OH
OH
MeO
MeO
OMe

cis-**12c**

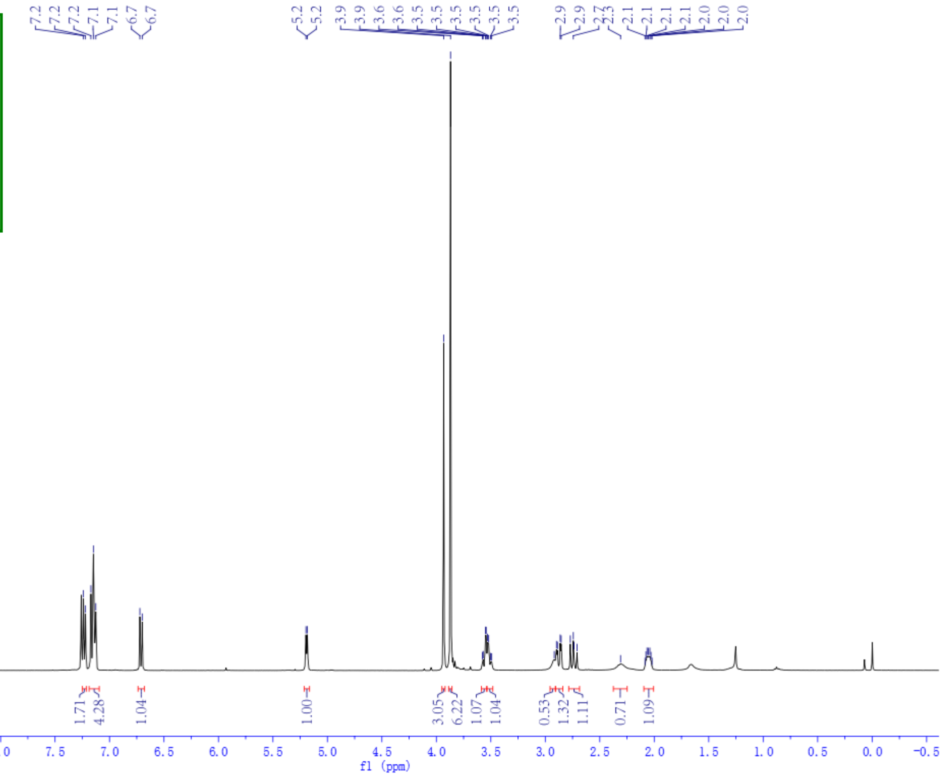
7.2
7.2
7.2
7.1
7.1
6.7
6.7
5.2
5.2
3.9
3.9
3.6
3.6
3.5
3.5
3.5
3.5
3.5
3.5
2.9
2.9
2.7
2.3
2.1
2.1
2.1
2.1
2.0
2.0
2.0
1.71
4.28
1.04
1.00
3.05
6.22
1.07
1.04
0.53
1.32
1.11
0.71
1.09
f1 (ppm)

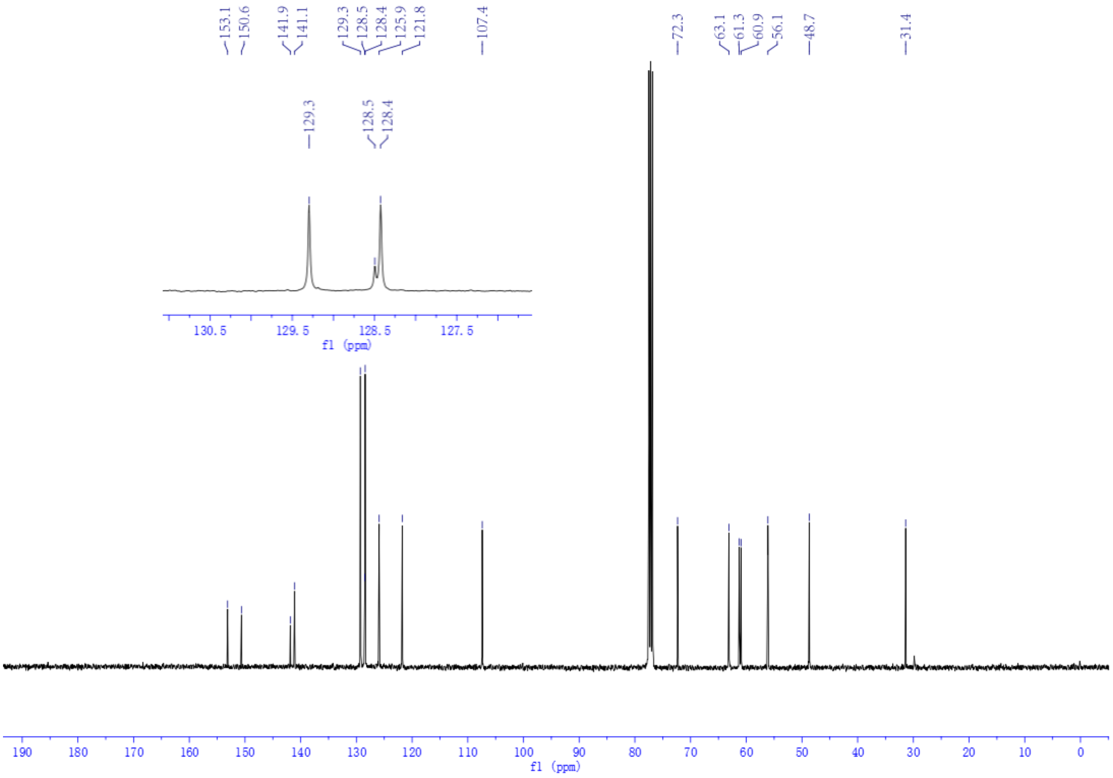
153.1
150.6
141.9
141.1
129.3
128.5
128.4
125.9
121.8
107.4
72.3
63.1
61.3
60.9
56.1
48.7
31.4
129.3
128.5
128.4
f1 (ppm)
f1 (ppm)

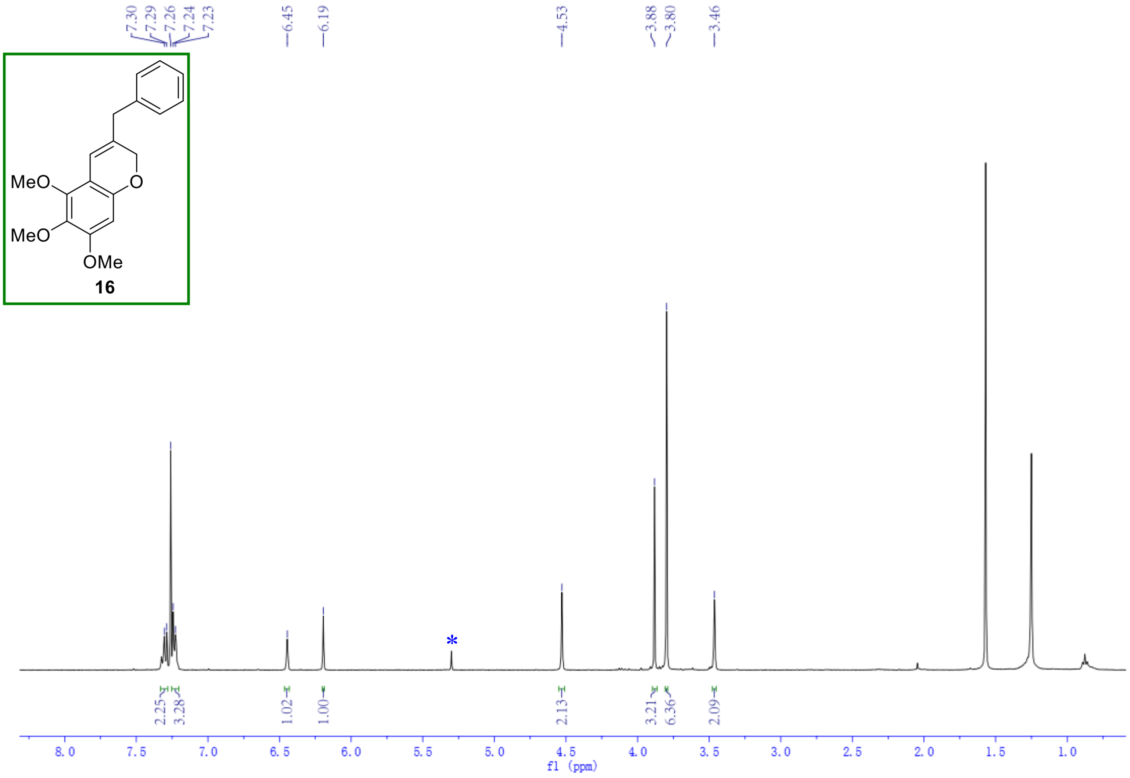
MeO
MeO
O
OMe
16
7.30
7.29
7.26
7.24
7.23
6.45
6.19
4.53
3.88
3.80
3.46
*
2.25
3.28
1.02
1.00
2.13
3.21
6.36
2.09
8.0
7.5
7.0
6.5
6.0
5.5
5.0
4.5
4.0
3.5
3.0
2.5
2.0
1.5
1.0
f1 (ppm)

* DCM

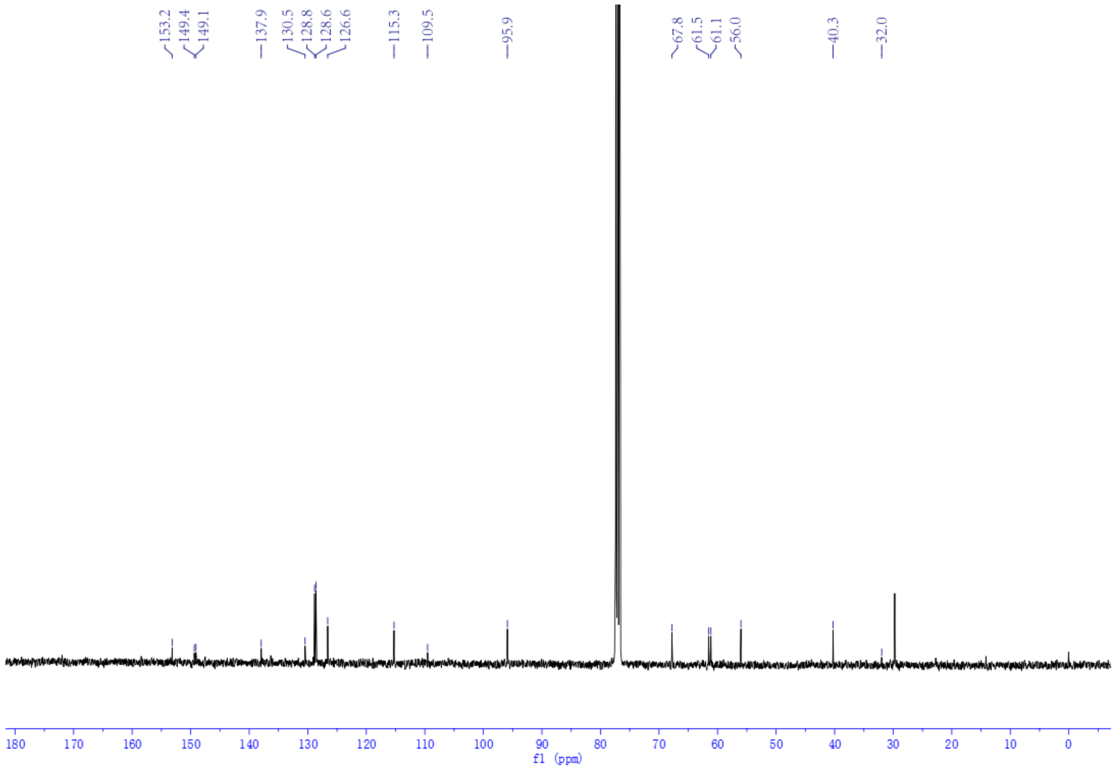
153.2
149.4
149.1
137.9
130.5
128.8
128.6
126.6
115.3
109.5
95.9
67.8
61.5
61.1
56.0
40.3
32.0
180
170
160
150
140
130
120
110
100
90
80
70
60
50
40
30
20
10
0
f1 (ppm)

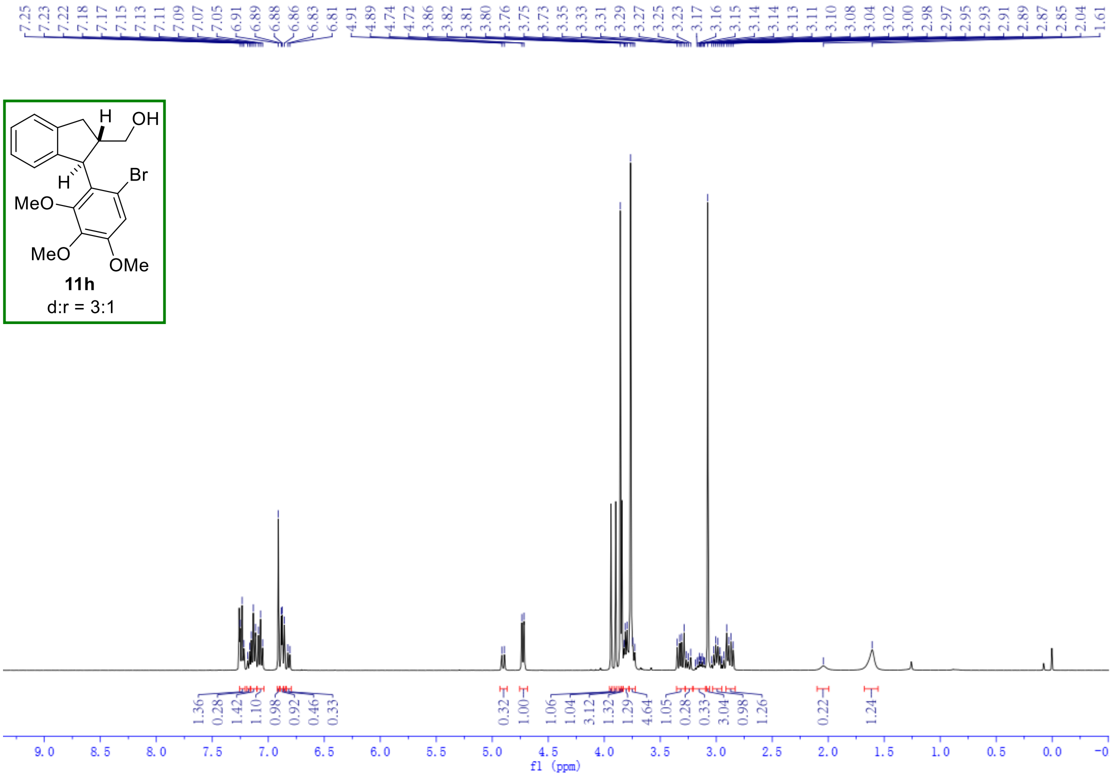
7.25 7.23 7.22 7.18 7.17 7.15 7.13 7.11 7.09 7.07 7.05 6.91 6.89 6.88 6.86 6.83 6.81 4.91 4.89 4.74 4.72 3.86 3.82 3.81 3.80 3.76 3.75 3.73 3.35 3.33 3.31 3.29 3.27 3.25 3.23 3.17 3.16 3.15 3.14 3.14 3.13 3.11 3.10 3.08 3.04 3.02 3.00 2.98 2.97 2.95 2.93 2.91 2.89 2.87 2.85 2.04 1.61
H
OH
H
Br
MeO
MeO
OMe
11h
d:r = 3:1
1.36 0.28 1.42 1.10 0.98 0.92 0.46 0.33 0.32 1.00 1.06 1.04 3.12 1.32 1.29 4.64 1.05 0.28 0.33 3.04 0.98 1.26 0.22 1.24
f1 (ppm)

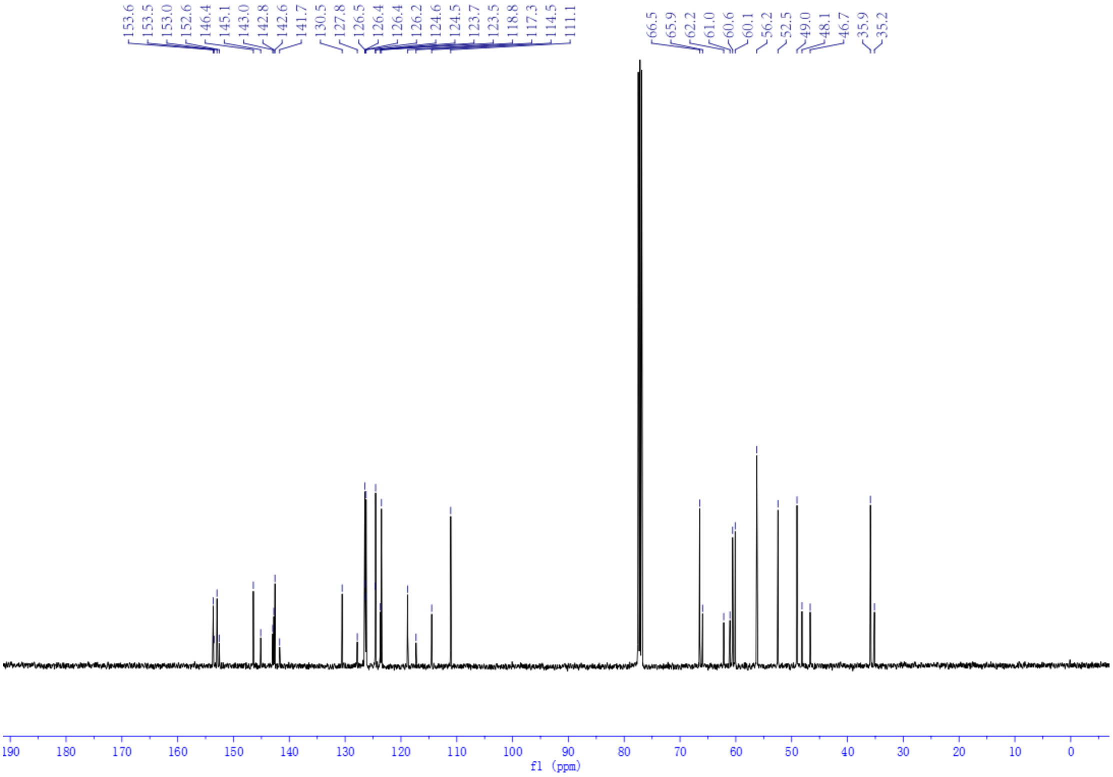
153.6 153.5 153.0 152.6 146.4 145.1 143.0 142.8 142.6 141.7 130.5 127.8 126.5 126.4 126.2 124.6 124.5 123.7 123.5 118.8 117.3 114.5 111.1 66.5 65.9 62.2 61.0 60.6 60.1 56.2 52.5 49.0 48.1 46.7 35.9 35.2
f1 (ppm)

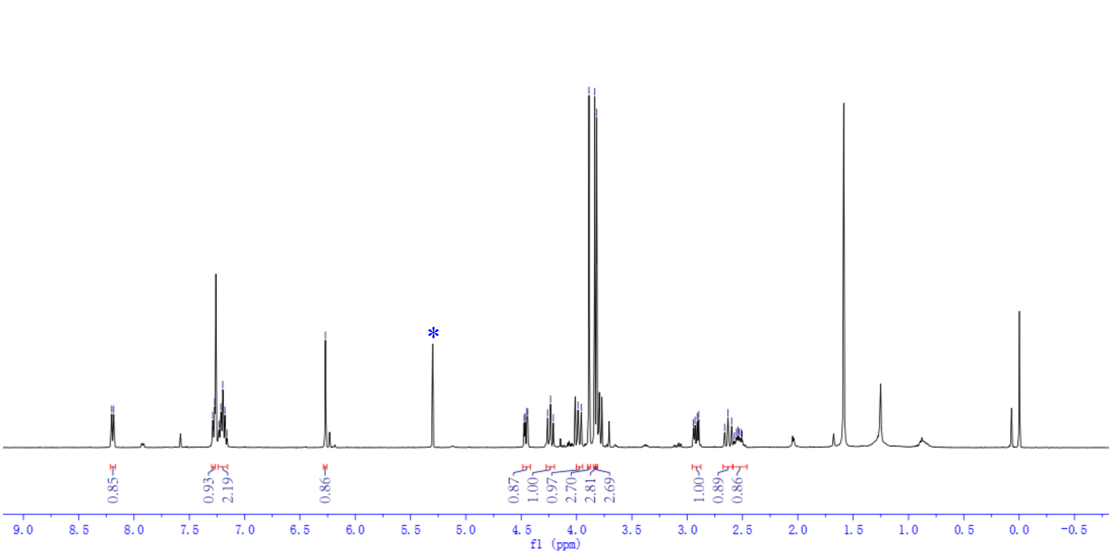
8.20
8.18
7.29
7.27
7.23
7.21
7.20
7.18
7.16
6.27
4.47
4.47
4.45
4.44
4.26
4.24
4.21
3.99
3.96
3.89
3.84
3.82
2.94
2.93
2.91
2.90
2.66
2.63
2.60
2.57
2.55
2.54
2.53
2.51
2.50
*
0.85
0.93
2.19
0.86
0.87
1.00
0.97
2.70
2.81
2.69
1.00
0.89
0.86
f1 (ppm)

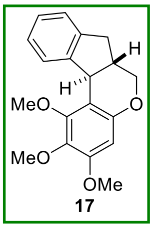
H
H
MeO
MeO
O
OMe
17

* DCM

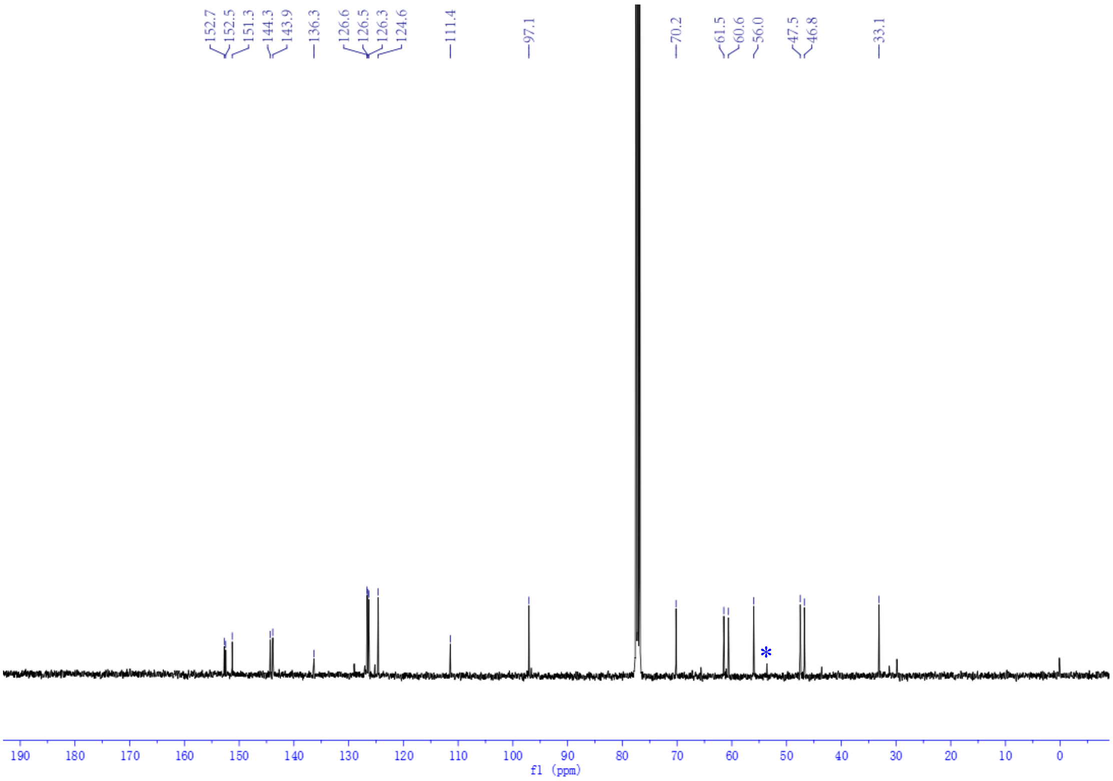
152.7
152.5
151.3
144.3
143.9
136.3
126.6
126.5
126.3
124.6
111.4
97.1
70.2
61.5
60.6
56.0
47.5
46.8
33.1
*
f1 (ppm)

X-ray crystallography data of compounds 11a-1 and 12b.

Crystal data for **11a-1** (**CCDC: 2339844**): $C_{19}H_{20}O_4 \cdot H_2O$, M = 330.36, a = 11.8151(4) Å, b = 5.6355(2) Å, c = 12.9829(5) Å, α = 90°, β = 95.508(2)°, γ = 90°, V = 860.46(5) Å^3, T = 150.(2) K, space group P1211, Z = 2, μ(Cu Kα) = 0.754 mm^{-1}, 6069 reflections measured, 2864 independent reflections (R_{int} = 0.0638). The final R_1 values were 0.0893 ($I > 2\sigma(I)$). The final $wR(F^2)$ values were 0.2369 ($I > 2\sigma(I)$). The final R_1 values were 0.0920 (all data). The final $wR(F^2)$ values were 0.2411 (all data). The goodness of fit on F^2 was 1.055. Flack parameter = 0.2(2).

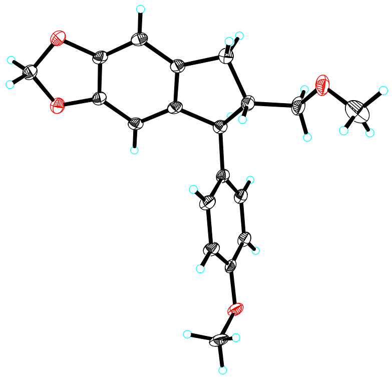

View of a molecule of **11a-1**.

Displacement ellipsoids are drawn at the 30% probability level.

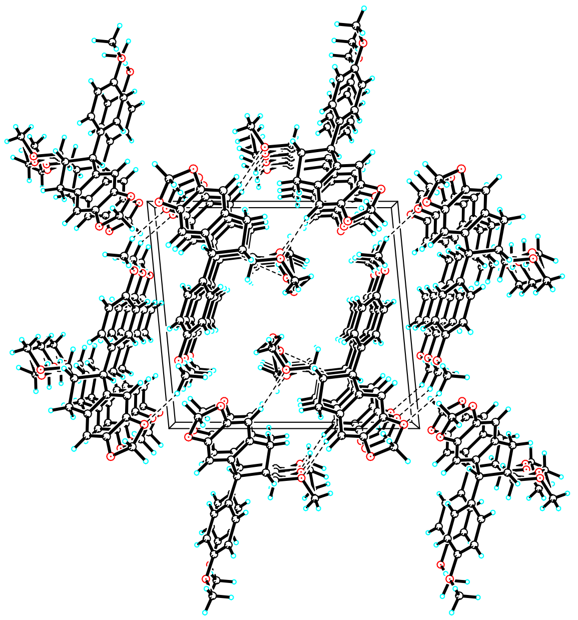

View of the pack drawing of **11a-1**.

Hydrogen-bonds are shown as dashed lines.

Crystal data for **12b** (**CCDC: 2339848**): $C_{16}H_{18}O_2$, M = 242.30, a = 5.4782(2) Å, b = 9.6101(3) Å, c = 25.0037(9) Å, α = 90°, β = 92.6360(10)°, γ = 90°, V = 1314.95(8) Å^3, T = 150.(2) K, space group $P121/c1$, Z = 4, μ(Cu Kα) = 0.626 mm^{-1}, 12271 reflections measured, 2425 independent reflections (R_{int} = 0.0497). The final R_1 values were 0.0374 ($I > 2\sigma(I)$). The final $wR(F^2)$ values were 0.0917 ($I > 2\sigma(I)$). The final R_1 values were 0.0424 (all data). The final $wR(F^2)$ values were 0.0937 (all data). The goodness of fit on F^2 was 1.032.

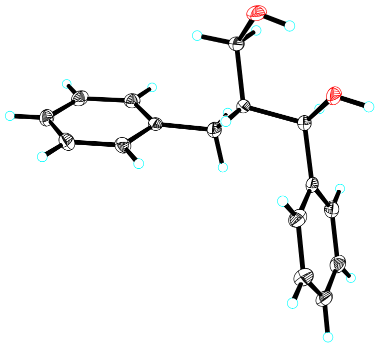

View of a molecule of **12b** with the atom-labelling scheme.

Displacement ellipsoids are drawn at the 30% probability level.

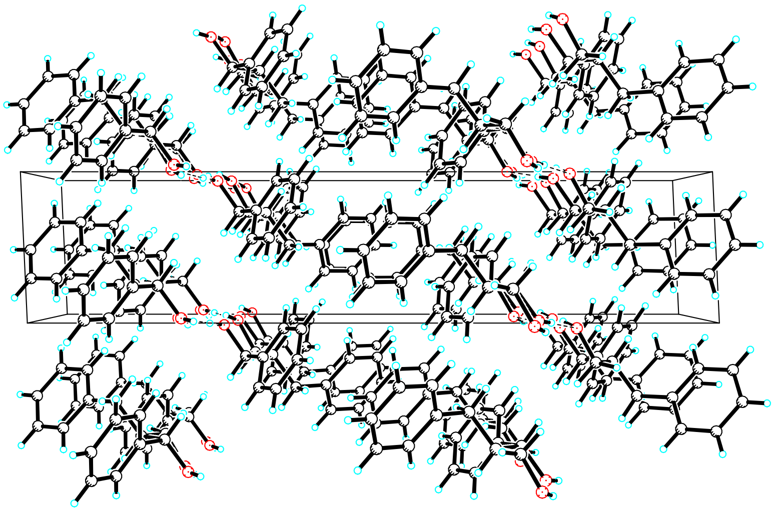

View of the pack drawing of **12b**.

Hydrogen-bonds are shown as dashed lines.
